# Biochemical Characterization of a Novel Redox-Regulated Metacaspase in a Marine Diatom

**DOI:** 10.3389/fmicb.2021.688199

**Published:** 2021-09-08

**Authors:** Shiri Graff van Creveld, Shifra Ben-Dor, Avia Mizrachi, Uria Alcolombri, Amanda Hopes, Thomas Mock, Shilo Rosenwasser, Assaf Vardi

**Affiliations:** ^1^Department of Plant and Environmental Sciences, Weizmann Institute of Science, Rehovot, Israel; ^2^School of Oceanography, University of Washington, Seattle, WA, United States; ^3^Department of Life Sciences Core Facilities, Weizmann Institute of Science, Rehovot, Israel; ^4^Department of Civil, Environmental and Geomatic Engineering, Institute for Environmental Engineering, Swiss Federal Institute of Technology, Zurich, Switzerland; ^5^School of Environmental Sciences, University of East Anglia, Norwich, United Kingdom; ^6^Robert H. Smith Faculty of Plant Sciences and Genetics in Agriculture, The Hebrew University of Jerusalem, Jerusalem, Israel

**Keywords:** diatom, metacaspase, *Phaeodactylum tricornutum*, redox-regulation, reactive oxygen species, infochemicals, programmed cell death, phytoplankton

## Abstract

Programmed cell death (PCD) in marine microalgae was suggested to be one of the mechanisms that facilitates bloom demise, yet its molecular components in phytoplankton are unknown. Phytoplankton are completely lacking any of the canonical components of PCD, such as caspases, but possess metacaspases. Metacaspases were shown to regulate PCD in plants and some protists, but their roles in algae and other organisms are still elusive. Here, we identified and biochemically characterized a type III metacaspase from the model diatom *Phaeodactylum tricornutum*, termed PtMCA-IIIc. Through expression of recombinant PtMCA-IIIc in *E. coli*, we revealed that PtMCA-IIIc exhibits a calcium-dependent protease activity, including auto-processing and cleavage after arginine. Similar metacaspase activity was detected in *P. tricornutum* cell extracts. PtMCA-IIIc overexpressing cells exhibited higher metacaspase activity, while CRISPR/Cas9-mediated knockout cells had decreased metacaspase activity compared to WT cells. Site-directed mutagenesis of cysteines that were predicted to form a disulfide bond decreased recombinant PtMCA-IIIc activity, suggesting its enhancement under oxidizing conditions. One of those cysteines was oxidized, detected in redox proteomics, specifically in response to lethal concentrations of hydrogen peroxide and a diatom derived aldehyde. Phylogenetic analysis revealed that this cysteine-pair is unique and widespread among diatom type III metacaspases. The characterization of a cell death associated protein in diatoms provides insights into the evolutionary origins of PCD and its ecological significance in algal bloom dynamics.

## Introduction

Diatoms are an important phytoplankton group that is responsible for about half of marine photosynthesis, playing a significant role in global biogeochemical cycles and in carbon sequestration (Nelson et al., 1995; Rousseaux and Gregg, 2013). Their evolutionary and ecological success in contemporary oceans suggests that diatoms possess sophisticated mechanisms for adaptation to diverse environmental conditions (Falciatore and Bowler, 2002). Diatoms can form massive blooms that are controlled by abiotic factors such as the availability of nutrients and light, and by biotic interactions with grazers, bacteria and viruses (Nagasaki et al., 2004; Vanelslander et al., 2012; Assmy et al., 2013; Kimura and Tomaru, 2014; Bertrand et al., 2015). Bloom termination and the rapid turnover of phytoplankton were suggested to involve programmed cell death (PCD) as an important mortality mechanism (Bidle, 2016).

Diverse biotic and abiotic stress conditions can lead to the production of an array of bioactive compounds (infochemicals) that can regulate cell fate and shape population dynamics (Vanelslander et al., 2012; Gillard et al., 2013; Poulson-ellestad et al., 2014; Gallo et al., 2017). Grazing or nutrient stress, can rapidly induce the biosynthesis of diatom-derived oxylipins such as (*E,E*)-2,4-Decadienal (DD; Pohnert, 2000; Ribalet et al., 2007). DD may act as a chemical defense against grazing (Miralto et al., 1999; Ianora et al., 2004, 2006; Marrone et al., 2012), and as a signaling molecule that enables cell–cell communication within diatom populations (Casotti et al., 2005; Ianora et al., 2006; Vardi et al., 2006). Lethal doses of DD can initiate a signaling pathway which includes Ca^2+^ transients, nitric oxide production and redox-dependent PCD in the model diatom *Phaeodactylum tricornutum* (Vardi et al., 2006, 2008; Graff van Creveld et al., 2015). Reactive oxygen species (ROS) are known to play an important role in stress sensing and cell fate regulation across kingdoms, from bacteria to plants and animals (Vardi et al., 1999; D’Autréaux and Toledano, 2007; Mittler et al., 2011; Suzuki et al., 2012; Dietz et al., 2016). However, the actual redox-sensitive proteins, and the specific oxidation events that regulate cell fate are under-explored in well-established model-systems, and unknown in diatoms.

**Figure 1:**
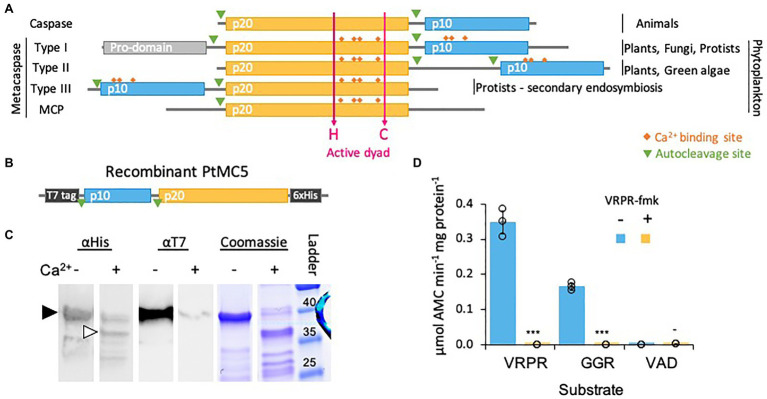
PtMCA-IIIc exhibits Ca^2+^ dependent autocleavage and MC-typical protease activity. **(A)** Domain organization of caspases, MCs, and MC-like proteases (MCPs), as previously described (Vercammen et al., 2007; Choi and Berges, 2013). The p20 and p10 domains are marked in orange and blue, respectively. Ca^2+^ binding sites are marked in orange rhombuses, the active His-Cys dyad in magenta arrows. **(B)** Schematic representation of the recombinant tagged PtMCA-IIIc expressed in *E. coli*, putative autocleavage sites are marked with green triangles. **(C)** Coomassie stained SDS-PAGE gel and immunoblot with antibodies against His or T7 tags. About 2.5μg purified protein extracts per lane were incubated with (+) or without (−) 10mM CaCl_2_ for 30min prior to gel loading. The black arrowhead represents full length PtMCA-IIIc, white arrowhead represents auto-cleaved PtMCA-IIIc as detected following Ca^2+^ addition. Protein ladder (size in kD) is presented to the right. **(D)** Protease activity of purified recombinant PtMCA-IIIc, measured by the release of AMC from peptidyl substrates, VRPR-AMC, GGR-AMC and VAD-AMC, with (+) or without (−) 25μM of the MC inhibitor VRPR-fmk. Standard curve was used to convert the relative fluorescence units into μmol of free AMC released per min per mg of total protein. Single measurements are indicated in circles, bars are means ± s.d. of triplicates, compared to no inhibitor ^***^p=1.4×10^−5^, ^**^p=1.1×10^−4^, −*p*=0.013.

Despite accumulated evidence of PCD in diatoms and in phytoplankton in general, the genes and proteins that regulate and execute PCD in diatoms are yet unknown. Phytoplankton lack most canonical PCD related proteins, such as Bcl2, p53, and caspases. Caspases are a family of cysteine-dependent aspartate-directed proteases that coordinate and execute various PCD pathways in animals (Kumar, 2007). While caspases are unique to metazoans, other organisms and microorganisms express structural homologues that share the active cysteine-histidine dyad, known as metacaspases (MCs; Uren et al., 2000). In contrast to caspases, MCs act in monomers and cleave their targets after arginine or lysine (Watanabe and Lam, 2005; Tsiatsiani et al., 2011). MCs are functionally diverse and exhibit different roles in autophagy and cell fate regulation, stress response and development in various organisms including plants, fungi and pathogenic protozoan (Escamez et al., 2016; Kabbage et al., 2017; Balakireva and Zamyatnin, 2019). MCs are divided into four subgroups defined by the arrangement of the short p10 domain, and the catalytic p20 domain (Figure 1A). A bioinformatics analysis in algal genomes identified type III MCs, the only type in which the p10 domain precedes the p20 domain (Choi and Berges, 2013; Figure 1A). Type III MCs are absent in plants and green algal lineages, but are prevalent in algae that originated from secondary endosymbiosis, including diatoms (Choi and Berges, 2013; Klemenčič and Funk, 2018a). Expression levels of some MCs in diatoms were induced during nutrients limitations that led to the induction of PCD (Bidle and Bender, 2008; Thamatrakoln et al., 2012; Orefice et al., 2015; Wang et al., 2017), but biochemical characterization and functional roles of diatom MCs in PCD and stress acclimation are yet to be described.

In this study, we combined biochemical characterization of a recombinant type III MC from the model diatom *P. tricornutum* (PtMCA-IIIc), with functional characterization of genetically modified *P. tricornutum* cells, in order to unveil the function and role of MCs in diatoms cell fate regulation. We demonstrate that *PtMCA-IIIc* encodes an active Ca^2+^ dependent Cys-protease; and identified a unique redox regulation of MC activity by oxidation of two regulatory Cys. This regulatory Cys pair is specific to diatom type III MCs, forming a novel subfamily of type III MCs.

## Materials and Methods

### Culture Growth

*P. tricornutum*, accession Pt1 8.6 (CCMP2561 in the Provasoli-Guillard National Center for Culture of Marine Phytoplankton) was purchased from the National Center of Marine Algae and Microbiota (NCMA, formerly known as CCMP). Cultures were grown in f/2 media in filtered seawater (FSW) at 18°C with 16:8h light:dark cycles and light intensity of 80μmol photons·m^−2^·sec^−1^ supplied by cool-white LED lights. Unless specified otherwise, experiments were initiated with exponentially growing cultures at ~5·10^5^cellsml^−1^.

### Cell Death

Cell death was determined by positive Sytox Green (Invitrogen) staining, used at a final concentration of 1μM. Samples were incubated in the dark for 30min prior to measurement. Positive gating was based according to untreated cells and unstained cells.

### Infochemical Preparation

(*E,E*)-2,4-decadienal (DD; 95%, Acros Organics) solutions were prepared by diluting the stock in absolute methanol on ice. DD was added to the cells at a dilution of at least 1:200. Control cultures were treated by the addition of methanol to the same dilution as the treatment culture.

### Flow Cytometry

Flow cytometry measurements (cell abundance and Sytox staining) were obtained using Eclipse iCyt flow cytometer (Sony Biotechnology Inc., Champaign, IL, United States), equipped with a 488nm solid state air cooled 25mW laser with a standard filter setup. Cells were identified by plotting chlorophyll fluorescence in the red channel (737–663nm) vs. green fluorescence (500–550nm) or forward scatter. At least 5,000 cells were analyzed per sample, with at least three biological replicates.

### Identification of Redox Sensitive Cysteines

25μM DD treatment was applied to *P. tricornutum* cells that were either pre-treated with 5μM DD 2.5h before (non-lethal condition, “5+25μM DD”) or without pre-treatment (lethal condition, “25μM DD”). These conditions were chosen because Vardi et al. (2006) demonstrated that pre-treatment with a low dose of DD alters the DD-induced calcium signaling and prevents cell death. The prevention of DD induced PCD by pre-treatment with a non-lethal dose was further recapitulated (Sabharwal et al., 2017). After 2h, cells were sampled by centrifugation of 200ml per sample. Proteins were extracted and cysteine oxidation was assessed as previously described by Rosenwasser et al. (2014). To summarize, proteins were extracted by sonication and the pellet was dried under nitrogen flow to avoid cysteine oxidation. Subsequent to extraction, proteins were dissolved in denaturing buffer (50mM Tris, pH=8.5 and 0.1% SDS) and subjected to thiol trapping according to the OxICAT methodology (Leichert, 2010) using the cleavable ICAT reagent kit for protein labeling (AB Sciex, Foster City, CA, United States). Downstream proteomics analysis including peptide liquid chromatography, mass spectrometry and data processing were carried out exactly as previously described (Rosenwasser et al., 2014). The full list of H_2_O_2_ sensitive proteins is present in Rosenwasser et al. (2014) and Supplementary Table 1. The list of all MCs oxidation degree in all treatments examined is present here in Supplementary Table 1.

### PtMCA-IIIc Gene and Protein Modeling

The gene sequence and amino acid sequence of PtMCA-IIIc were obtained from the JGI genome portal (synonym: PtMC5, protein ID: 54873, transcript ID: estExt_Phatr1_ua_kg.C_chr_160041), and corrected manually using ESTs (the prediction extended the sequences artificially by two exons on the 5' end which were removed in the final sequence). Conserved domain prediction [CDD; https://www.ncbi.nlm.nih.gov/Structure/bwrpsb/bwrpsb.cgi, and (Choi and Berges, 2013)] were used to obtain protein domains. Modeling of the 3D structure of PtMCA-IIIc was performed online using the SwissModel server^1^ based on the structure of *S. cerevisiae* MC (ScMCA-I, 4F6O; Wong et al., 2012) as a template. Molecular graphics were prepared using PyMOL software (http://www.pymol.org/, Schröedinger).

### Bacterial Cloning

cDNA of PtMCA-IIIc was ordered from GENWIZ (in pUC57), ligated into the bacterial expression vector pET-21a using EcoRI and XhoI restriction sites. This construct was subsequently used as a template for the preparation of PtMCA-IIIc mutants (C202S, C259S or C264S), using site directed mutagenesis (SDM). This was carried out using mutagenesis primers 1–6 (listed in Supplementary Table 3) with either KAPA polymerase PCR followed by DpnI digestion or by using a Q5 SDM kit (New England Biolabs, E00554S). Correct ligation and incorporation of mutations was verified by DNA sequencing using primers 7, 8 (Supplementary Table 3).

### PtMCA-IIIc Expression and Purification

MC expression, purification and activity assays were adapted from Bozhkov and Salvesen (2014) and McLuskey et al. (2014). *E. coli* Rosetta cells were transformed with the expression plasmids and grown in LB containing ampicillin in a shaker at 37°C. When O.D. reached 0.6, 1mM IPTG was added for overnight shacking at 16°C. Cell pellets collected from 100ml of bacterial culture were resuspended in 3.5ml of lysis buffer (150mM NaCl, 25mM HEPES, 10% glycerol, 0.2% triton, 1mgml^−1^ lysozyme, 1μl benzonase, 0.5mM DTT, pH 7.8) and sonicated 10×10 sec on ice. Following centrifugation at 14,000g for 5min to remove insoluble debris, the supernatant was applied to Ni-NTA resin (Ni-NTA His•Bind® Resin, Milipore, 70,666-3). After washing with base buffer (150mM NaCl, 25mM HEPES, 10% glycerol), and with base buffer containing 20 and 30mM imidazole, the bound proteins were eluted in base buffer, containing 200mM imidazole. The elution was concentrated and washed with base buffer using an Amicon Centrifugal Filter Units (Millipore) equipped with a 10kDa exclusion membrane.

Protein concentration was determined using the BCA method and the samples were diluted to the lowest concentration in base buffer. Samples were then incubated with or without 10mM Ca^2+^. Samples were incubated at 95° C for 5min and loaded on Tris-Glycine eXtended gels (Criterion TGX Gels Any kD, BioRad) and subjected to protein gel analysis using Coomassie brilliant blue, or blotted onto a poly(vinylidene difluoride; PVDF) membrane and analyzed using HRP-anti-6xHis or HRP-anti-T7 antibodies (Zotal). ECL-Prime western blotting detection reagent (GE Healthcare) was used for detection.

### Kinetic Assays

Purified PtMCA-IIIc or *P. tricornutum* cell lysate (10^8^ cells were harvested, resuspended in 250μl lysis buffer, sonicated, and centrifuged to remove insoluble debris) was used for kinetic measurements. In each experiment the protein concentration was calculated by the BCA method and all samples were diluted in base buffer. Purified PtMCA-IIIc was used at about 60ng per well and *P. tricornutum* protein extracts were used at about 30μg per well. Protein extracts were incubated in activity buffer (base buffer with 0.1% CHAPS, 10mM DTT, 10mM CaCl_2_, pH 7.8) for 30min in 18°C prior to addition of the substrate. MC-typical activity, cleavage after arginine/lysine, was assessed using the short peptides Val-Arg-Pro-Arg (VRPR) and Gly-Gly-Arg (GGR) conjugated to the fluorophore 7-Amino-4-methylcoumarin (AMC). Following proteolytic activity, the fluorophore was released to the media and its fluorescence was detected over time with 360nm excitation and 460nm emission using a plate reader (Infinite 200 pro, Tecan). A calibration curve with 12, 6, 3, 1.5, 0.75, 0μM AMC and initial slopes were used to calculate the activity (μmol AMC·min^−1^·mg protein^−1^) from the fluorescence measurements as previously described (McLuskey et al., 2014). Assays were performed in activity buffer unless otherwise stated, at 20°C in black 96 well plates (TAMAR). All assays were performed with 50μM substrate (z-GGR-AMC, Ac-VRPR-AMC, z-VAD-AMC and AMC, all from Bachem). Protease inhibitors, in which the uncleavable fluoromethylketone (fmk) group is conjugated to the short peptide, z-VRPR-fmk (25μM, MC inhibitor) and z-VAD-fmk (100μM, pan-caspase inhibitor; both from abcam) were incubated for 30min before the addition of the substrate. Importantly, the measurement of protease activity in cell extracts, in adequate activity buffer, does not represent the actual *in vivo* activity as MCs are often inactive zymogens, but the potential of MC typical activity upon activation signal.

### gRNA Design for PtMCA-IIIc Knockout

In order to inactivate PtMCA-IIIc we adapted for *P. tricornutum* the method established by Hopes et al. (2016, 2017) for the diatom *Thalassiosira pseudonana*. Two single guide RNA (sgRNA) were designed to cut 115 nucleotides, which includes the catalytic Cys and the 3^rd^ intron. Selection of CRISPR/Cas9 targets and estimating on-target score: 20bp targets with an NGG PAM were identified and scored for on-target efficiency using the Broad Institute sgRNA design program,^2^ which utilizes the on-target scoring algorithm (Doench et al., 2014). The sgRNAs that were chosen had no predicted off-targets: The full 20nt target sequences and their 3' 12nt seed sequences were subjected to a nucleotide BLAST search against the *P. tricornutum* genome. Resulting homologous sequences were checked for presence of an adjacent NGG PAM sequence at the 3' end. The 8nt sequence outside of the seed sequence was manually checked for complementarity to the target sequence. In order for a site to be considered a potential off-target, the seed sequence had to match, a PAM had to be present at the 3' end of the sequence and a maximum of three mismatches between the target and sequences from the BLAST search were allowed outside of the seed sequence.

### Plasmid Construction Using Golden Gate Cloning

Golden Gate cloning was carried out as previously described (Weber et al., 2011), using a design similar to Hopes et al. (2016; Supplementary Figure 6A). BsaI sites and specific 4nt overhangs for Level 1 (L1) assembly were added through PCR primers. Golden Gate reactions for L1 and Level 2 (L2) assembly were carried out, 40 fmol of each component were included in a 20μl reaction with 10units of BsaI or BpiI and 10units of T4 DNA ligase in ligation buffer. The reaction was incubated at 37°C for 5h, 50°C for 5min and 80°C for 10min. Then, 5μl of the reaction was transformed into 50μl of NEB 5α chemically competent *E. coli*.

Level 0 assembly: The endogenous FCP promoter and terminator and the Ble resistance gene were amplified from the PH4-pPhat plasmid, and the U6 promoter (Nymark et al., 2016) was amplified from gDNA, using primers 9–14 and 19–20 (Supplementary Table 3). Both promoters are associated with high expression levels. Products were cloned into a pCR8/GW/TOPO vector (ThermoFisher). FCP promoter and terminator were “domesticated” to remove the BpiI sites using a Q5 SDM kit in L0 vectors using primers 15–18 (Supplementary Table 3). L0 Cas9YFP was a gift from Thomas Mock (Hopes et al., 2016). Level 0 PtU6 promoter was deposited in Addgene (#104895).

Level 1 assembly: FCP promoter, Ble and FCP terminator L0 modules were assembled into L1 pICH47732. FCP promoter, Cas9 and FCP terminator L0 modules were assembled into L1 pICH47742. Level 1 Ble and Cas9 under *P. tricornutum* FCP promoter and terminator were deposited in Addgene (#104893 and #104894 respectively). The sgRNA scaffold was amplified from pICH86966_AtU6p_sgRNA_NbPDS (Nekrasov et al., 2013) with sgRNA sequences integrated through forward primers 21–23 (Supplementary Table 3). Together with L0 U6 promoter, sgRNA_1 and sgRNA_2 were assembled into L1 destination vectors pICH47751 and pICH47761, respectively.

Level 2 assembly: L1 modules pICH47732:FCP:Ble, pICH47742:FCP:Cas9YFP, pICH47751:U6:sgRNA_PtMCA-IIIc 1, pICH47761: U6:sgRNA_PtMCA-IIIc 2 and the L4E linker pICH41780 were assembled into the L2 destination vector pAGM4723. Constructs were screened by digestion with EcoRV or EcoRI and by PCR. See Supplementary Figures 6A for an overview of the Golden Gate assembly procedure and the final construct.

### Transformations of *P. tricornutum*

Cells were transformed as previously described (Apt et al., 2002) using the Bio-Rad Biolistic PDS-1000/He Particle Delivery System fitted with 1,550psi rupture discs. Tungsten particles M17 (1.1mm diameter) were coated with 5μg circular plasmid DNA in the presence of 2.5M CaCl_2_ and 0.1M spermidine. Approximately 2·10^6^ cells were spread in the center of a plate of a solid medium (50% FSW+f/2, 1.5% agar) 2days before bombardment. For transformation, the plate was positioned at the second level within the Biolistic chamber. Bombarded cells were set to recover for 1day prior to suspension in 1ml sterile FSW+f/2. Cell suspension was plated onto solid medium containing 100μg·ml^−1^ Phleomycin. After 2–3weeks, resistant colonies were re-streaked onto fresh solid medium containing 100μg·ml^−1^ Zeocin.

### Selection of Knockout Lines

Resistant colonies were scanned for the presence of Cas9 by colony PCR. Cas9 positive colonies were scanned for the size of *PtMCA-IIIc* amplicon (primers 24–25 and 26–27, Supplementary Table 3), colonies exhibiting double-bands, representing both WT (714bp) and edited (~590bp) *PtMCA-IIIc* (probably heterozygotes or mosaic colonies) were re-streaked onto fresh solid medium containing 100μg·ml^−1^ Zeocin. Daughter colonies were scanned for the size of *PtMCA-IIIc* amplicon, colonies exhibiting a single band representing bi-allelic edited *PtMCA-IIIc* (~590bp) were selected and the *PtMCA-IIIc* gene was sequenced to determine the exact deletion (primers 26–28, Supplementary Table 3).

### RNA Isolation and RT-PCR Analysis

RNA was isolated from 50ml cultures with the Direct-zol RNA miniprep kit (Zymo research) according to the manufacturer’s instructions, followed by DNase treatment with Turbo DNase (Ambion). Equal amounts of RNA were used for cDNA synthesis with the ThermoScript RT-PCR system (Invitrogen). For transcript abundance analysis, Platinum SYBR Green qPCR SuperMix-UDG with ROX (Invitrogen) was used as described by the manufacturer. Reactions were performed on QuantStudio5 Real-Time PCR Systems (ThermoFisher) as follows: 50°C for 2min, 95°C for 2min, 40cycles of 95°C for 15s, 60°C for 30s. The primers for the *PtMCA-IIIc* gene capture the 1^st^ exon-intron junction and exon 2, detecting wild type (WT), overexpression (OE) and knockout (KO) *PtMCA-IIIc* (primers 33–34, Supplementary Table 3). Transcript abundance of PtMCA-IIIc was calculated by normalizing to expression of TBP (Siaut et al., 2007; primers 35–36, Supplementary Table 3) in each sample and to the expression of the WT sample.

### Identifying MC Genes From Various Species

Initial lists of genes were taken from the pico-Plaza^3^ gene family HOM000388. For the diatoms, sequences were taken from the Moore collection MMETSP (Keeling et al., 2014). Sequences from *Skeletonema costatum* (Skcos) and an additional isolate of *Thalassiosira rotula* (Throt) were provided by Harriet Alexander and Sonya T. Dyhrman (Alexander et al., 2015). For species with multiple isolates, the isolate with the most complete MCs was chosen. Full protein sequences are presented in Supplementary Data 1. Additional details can be found in the Supplementary Methods.

### Identifying p20 and p10 Domains

The putative protein sequences of the various MCs were run against the CDD database at NCBI to find the p20 domain. For many sequences, the p10 domain definition as available in the public domain databases (Pfam, CDD, InterPro) did not result in hits. Based on an alignment of our sequences and the supplemental alignment (Choi and Berges, 2013), we built new patterns to search for the p10 domain in various diatom sequences. The basis of the p10 pattern was a sequence of [QE]TSAD at the beginning and GAX[ST]XXXXXX[IVLA] in the middle. This was refined to Dx[QE]TSAD at the beginning, GGAX[ST] in the middle and QxPQL at the end of the putative p10. The patterns were the basis for the search which was performed manually on all of the defined diatom MCs. Putative p10 and p20 domains are marked on the full MCs protein sequences in Supplementary Data 1.

### Phylogenetic Tree Preparation

Alignments were performed on protein sequences of the p20 domains. The p20 domains were trimmed manually from the full-length sequences based on alignment to the CDD database, and further refined manually. Alignments were performed using ClustalW 2.1 (Supplementary Data 2). Unrooted phylogenetic trees were built using the Neighbor-joining algorithm in ClustalW (1,000 bootstraps and a seed of 111) and with Maximum likelihood (ProML) in the Phylip 3.697 package. Trees were visualized using the iTol server.^4^ In the subbranches the clustering was essentially the same using both algorithms, the tree built using the Neighbor-joining algorithm is presented.

### Statistical Analysis

All reported *p*-values were determined using a two-tailed unpaired Student’s t-test. In all figures, error bars represent SEM. and “n” represents the number of unrelated replicas in each treatment.

## Results

### *In vitro* Characterization of PtMCA-IIIc Biochemical Activity

MCs are promising cell fate regulation candidates in diatoms, as they were shown to be involved in stress response and PCD in other organisms (Escamez et al., 2016; Kabbage et al., 2017; Balakireva and Zamyatnin, 2019). Since they were not previously characterized in diatoms, we aimed to bridge the gap by investigating their function in the model diatom *P. tricornutum*. The *P. tricornutum* genome encodes five MCs that are divided into two types: PtMCA-a and PtMCA-b are MC-like protease (MCPs), and PtMCA-IIIa-c are type III MCs (see Figure 1A). The MCPs relative expression levels were very low under various conditions (data obtained from published transcriptomes; Smith et al., 2016; Matthijs et al., 2017; Supplementary Figure 1), and the proteins were not detected in previous experiments (data obtained from published proteomes; Rosenwasser et al., 2014; Graff van Creveld et al., 2016; Remmers et al., 2018). In contrast, the type III MCs, PtMCA-IIIa, PtMCA-IIIb, and PtMCA-IIIc proteins were detected in proteomics datasets (Rosenwasser et al., 2014; Graff van Creveld et al., 2016; Remmers et al., 2018), and had higher gene expression levels in all the examined conditions (data obtained from published transcriptomes; Smith et al., 2016; Matthijs et al., 2017; Supplementary Figure 1). Under steady state conditions the gene expression levels and protein abundances of the three type III MCs were similar.

We chose to focus on PtMCA-IIIc, which was highly expressed in three independent transcriptomes under diverse growth phases and that was induced under stress conditions, including transition to the dark, nitrogen limitation and phosphate limitation (Smith et al., 2016; Matthijs et al., 2017; McCarthy et al., 2017; Supplementary Figure 1). Based on protein sequence alignment, we detected conserved Ca^2+^ binding sites in PtMCA-IIIc p20 and p10 domains, and presumed auto-cleavage sites between the p10 and p20 domains and before the p10 domain (Supplementary Figure 2). To characterize its biochemical function, we heterologously expressed a recombinant T7 and 6xHis tagged PtMCA-IIIc in *E. coli* cells (Figure 1B). A full-length PtMCA-IIIc protein was detected by a distinct ~40 kD band on SDS-PAGE and immunoblots with antibodies either against the N-terminus T7 tag or the C-terminus 6xHis tag (Figure 1C, black arrowhead). Incubation of PtMCA-IIIc with 10mM CaCl_2_ for 30min led to autoprocessing and revealed several new shorter bands. A~37 kD band (Figure 1C, white arrowhead) may represent cleavage before the p10 domain (positions 5–6), this corresponding band of T7-taged N-terminus is <1kD, below the detection limit. Whilst cleavage between the p10 and p20 domains (positions 122–123) may lead to a ~25kD fragment, the possible cleavage of the T7-tag can explain the absence of a corresponding ~15kD band with the T7 antibody (Figure 1C; Supplementary Figures 2, 3). All the above suggested autocleavage sites are after arginine or lysin residues, as MCs typically cleave after those amino-acids, and often between the p20 and p10 domains (Moss et al., 2007; Watanabe and Lam, 2011). Cleavage after the putative cleavage sites (shown in Supplementary Figure 2) can explain the fragments detected following Ca^2+^ addition (Figure 1C; Supplementary Figure 3).

Previous studied showed that in vitro activation of MCs requires millimolar concentrations of Ca^2+^, which binds to the Ca^2+^ binding site, and dithiothreitol (DTT), a reductant essential for the reactivity of the active-site Cys (Martin et al., 2014; Minina et al., 2014). Under reduced conditions, PtMCA-IIIc exhibited MC-typical activity, i.e., calcium-dependent cleavage after arginine, monitored by GGRase activity (cleavage after the short peptide Gly-Gly-Arg). This reached saturation at 10mM Ca^2+^ and was dependent on DTT concentration (Supplementary Figure 4). PtMCA-IIIc displayed preferential VRPRase activity, which was 2-fold higher than its GGRase activity. In contrast, PtMCA-IIIc exhibited no caspase-typical activity, as cleavage of the pan-caspase substrate z-VAD-AMC was ~3 orders of magnitude lower than cleavage of VRPR-AMC (0.0007±0.0002 and 0.3475±0.0320μmol AMC·min^−1^·mg protein^−1^ respectively, Figure 1D). PtMCA-IIIc activity was completely abolished by the MC inhibitor z-VRPR-fmk (25μM), but was unaffected by the pan-caspase inhibitor z-VAD-fmk (100μM, *p*=0.43; Figure 1D; Supplementary Figure 5A). Together, these results demonstrate that PtMCA-IIIc exhibits a Ca^2+^ dependent MC-typical activity and does not exhibit caspase-typical activity.

**Figure 2:**
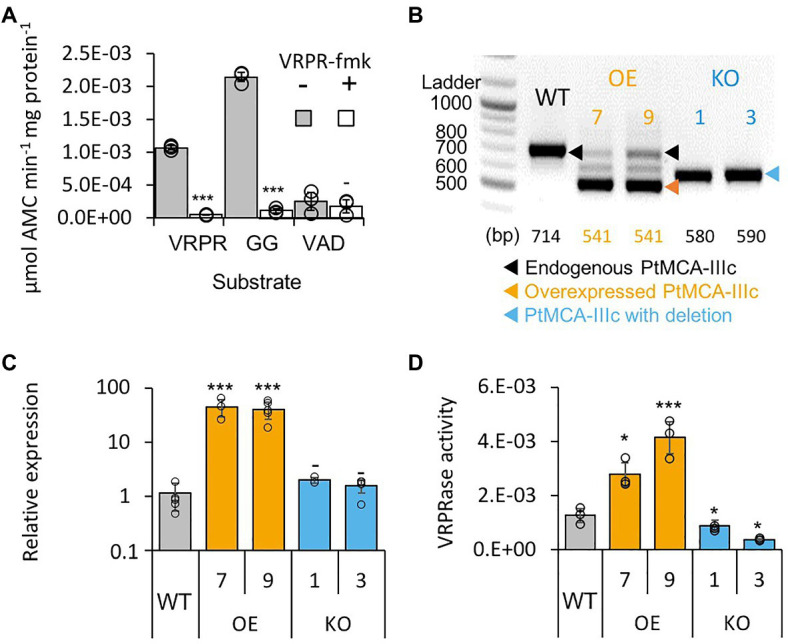
PtMCA-IIIc exhibit MC-typical activity in *P. tricornutum* cell extracts. **(A)** Protease activity of protein extracts from exponential *P. tricornutum* cells, measured by the release of AMC from peptidyl substrates, VRPR-AMC, GGR-AMC, and VAD-AMC, with (+) or without (−) 25μM of the MC inhibitor VRPR-fmk. Standard curve was used to convert the relative fluorescence units into μmol of free AMC released per min per mg of total protein. Single measurements are indicated in circles, bars are means ± s.d. of triplicates, compared to no inhibitor −*p*>0.05, ^**^p<0.005, ^***^p<0.001. **(B)** PCR of the *PtMCA-IIIc* gene in WT, overexpression (OE) and knockout (KO) lines. DNA ladder (size in bp) is present in the left lane, the predicted band sizes are indicated below. OE lines were generated using a cDNA construct without the introns, hence the shorter band, in addition to the endogenous *PtMCA-IIIc*. Homozygous deletion events, induced by CRISPR/Cas9 directly evidenced by the presence of a single, shorter PCR product (KO1, KO3), compared to WT cells. **(C)** Expression levels of PtMCA-IIIc normalized to TATA box Binding Protein (TBP), and to WT cells, measured by RT-qPCR, in WT, OE and KO lines. Single measurements are indicated in circles, bars are means ± s.d of biological triplicates. **(D)** PtMCA-IIIc VRPRase activity (μmol AMC min^−1^ mg protein^−1^) in protein extracts of WT, OE, and KO *P. tricornutum* lines. Single measurements are indicated in circles, bars are means ± s.d. of triplicates. Each transformant line was compared to WT −*p*>0.05, ^*^p<0.05, ^***^p<0.005.

Following the *in vitro* biochemical characterization of recombinant PtMCA-IIIc, we examined whether its typical activity could also be detected in cell extracts of *P. tricornutum*. Similar to the recombinant PtMCA-IIIc, *P. tricornutum* cell extracts exhibited typical MC activity, showing cleavage after arginine, albeit with preference to GGRase over VRPRase activity. This MC-typical activity was an order of magnitude higher than caspase-typical activity (VADase; Figure 2A). In accordance, MC-typical activity, but not the VADase activity, was inhibited by the MC inhibitor z-VRPR-fmk (25μM, Figure 2A). The caspase inhibitor z-VAD-fmk (100μM) inhibited VRPRase activity by ~20% (*p*=0.004), but did not affect the VADase activity (*p*=0.430; Supplementary Figure 5B). These results demonstrate MC-typical activity in *P. tricornutum* cell extracts, which is likely derived from the combined activity of PtMCAs and additional proteases.

Since PtMCA-IIIc transcription was shown to be induced along the growth curve (data from Matthijs et al., 2017; Supplementary Figure 1A), we monitored MC-typical activity during 8days of growth (Supplementary Figure 5C). On day 3 (exponential phase), GGRase and VRPRase activity exhibited a 2.3 and 2.5-fold increase respectively, compared to day 0 (*p*=0.00014 for both, Supplementary Figure 5C). MC-typical activity reached a maximum in early stationary phase, with 1.7 and 1.5 fold increases in day 6 compared to day 3 of GGRase (*p*=0.0050) and VRPRase (*p*=0.0049) activity, respectively. This might suggest a physiological role for PtMCA-IIIc in early stationary phase.

### Functional Characterization of PtMCA-IIIc in *P. tricornutum* Cells

To verify that PtMCA-IIIc is responsible for MC-typical activity in *P. tricornutum* cell lysate we either overexpressed PtMCA-IIIc, or used CRISPR/Cas9 to delete the active site (Supplementary Figures 6A,B). Two independent overexpression (OE7, OE9) and knockout (KO1, KO3) transformant lines were selected after verification using PCR screening of the *PtMCA-IIIc* gene. In the OE lines, overexpressed *PtMCA-IIIc* had a shorter band, as expected due to the lack of introns, in addition to the endogenous *PtMCA-IIIc* (Figure 2B). Higher expression of *PtMCA-IIIc* in OE lines compared to WT was verified by RT-qPCR (Figure 2C). In the KO lines, an edited *PtMCA-IIIc* was detected, indicating a bi-allelic deletion of ~100bp (Figure 2B). Exact deletions were assessed by DNA sequencing (Supplementary Figure 6C). Importantly, in the two KO lines, PtMCA-IIIc lacked the putative catalytic Cys (C264), and the deletion led to a frame-shift and an early stop codon (Supplementary Figure 6D). VRPRase activity in the *P. tricornutum* cell extracts of the transformant lines was 2.2–3.3 fold higher in OE lines compared to WT, and 0.3–0.7 fold lower in KO lines compared to WT (Figure 2D), indicating that PtMCA-IIIc is responsible for at least part of the VRPRase activity detected in cell extracts. The growth rate of all transformant lines were comparable with WT, but KO lines reached lower cell concentrations in stationary phase compared to WT (day 7: KO1, *p*=0.0001; KO3, *p*=0.0055, Supplementary Figure 7).

### C264 Is Essential for PtMCA-IIIc Catalytic Activity, While C202 Is a Regulatory Cys

**Figure 3:**
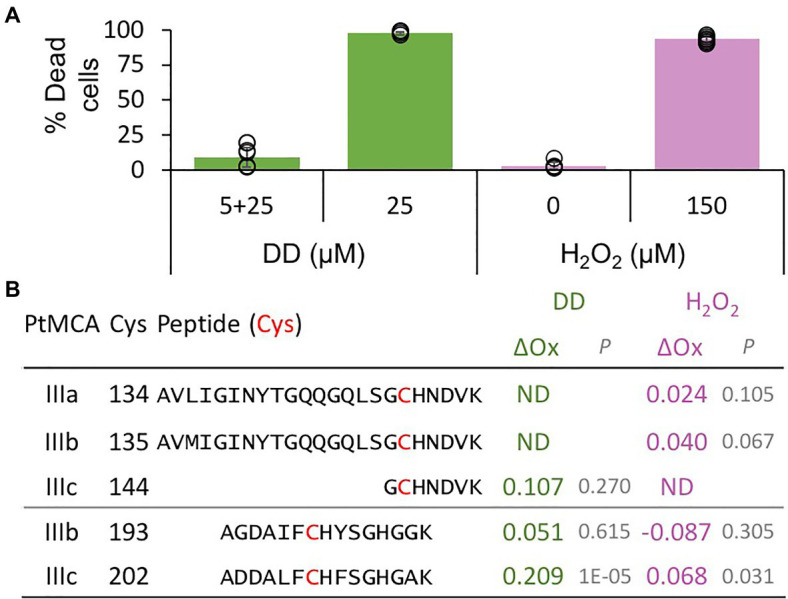
PtMCs peptides detected by redox proteomics in response to lethal treatments. *P. tricornutum* cells were treated with 5μM DD, after 2.5h 25μM DD was added to treated (5+25, non-lethal) and untreated cells (25, lethal). In addition, *P. tricornutum* cells were treated with 0, 150μM H_2_O_2_. **(A)** Cell death was measured as Sytox positive cells 24h after treatment. Single measurements are indicated in circles, bars are means ± s.d. of triplicates. **(B)** DD-treated cells were sampled for redox proteomics 2h after 25μM DD (Graff van Creveld, 2018), while H_2_O_2_ treated cells were sampled 20min after H_2_O_2_ addition (Rosenwasser et al., 2014). Degree of oxidation of cysteines in detected peptides was measured and the oxidation degree of the non-lethal treatments (5+25μM DD, and 0μM H_2_O_2_) was subtracted from the oxidation degree of the lethal treatments (25μM DD, and 150μM H_2_O_2_), to calculate the delta oxidation (ΔOx). PtMCA number, Cys number, and detected peptide (detected Cys marked in bold red) are shown. ΔOx of detected Cys between lethal and non-lethal treatments (average), and corresponding *p* value are shown. ND – not detected.

**Figure 4:**
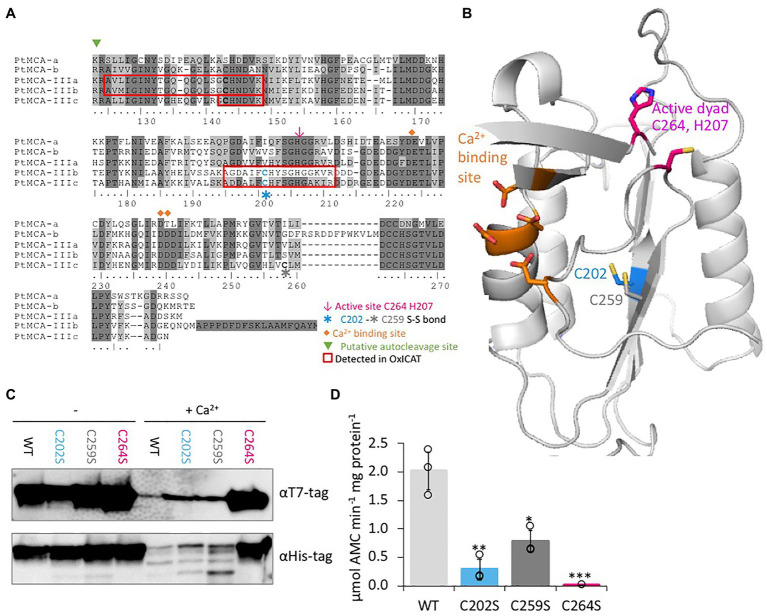
C264 is essential for PtMCA-IIIc activity, C202 and C259 are regulatory cysteines. **(A)** Protein sequence alignment of PtMCAs p20 domains, amino-acids numbered by PtMCA-IIIc sequence. Identical residues have dark gray background and similar amino acids have light gray background 70% threshold for coloring. Active dyad, C202, C259, Ca^2+^ binding site, and autocleavage sites are marked. Peptides detected in redox proteomics are framed in red. **(B)** PtMCA-IIIc 3D structure model based on ScMCA-I structure (Wong et al., 2012), cartoon representation by PyMOL. Key amino acids are presented in stick view, sulfur, oxygen and nitrogen atoms marked conventionally in yellow, blue and red, respectively. Aspartates of the Ca^2+^ binding site are marked in orange, the active Cys histidine dyad is marked in magenta, C202 which was detected in redox proteomics is marked in blue, neighboring C259 is marked in dark gray. Alignment of PtMCA-IIIc sequence and structure to ScMCA-I is presented in Supplementary Figure 8. **(C)** Immunoblot with (HRP) αHis or αT7 tags of PtMCA-IIIc, PtMCA-IIIc^C202S^, PtMCA-IIIc^C259S^, and PtMCA-IIIc^C264S^. About 0.88μg protein extracts per lane were incubated in activity buffer without / with 10mM CaCl_2_ for 10min prior to gel loading. **(D)** VRPRase activity of recombinant PtMCA-IIIc, PtMCA-IIIc^C202S^, PtMCA-IIIc^C259S^, and PtMCA-IIIc^C264S^. Single measurements are indicated in circles, bars are means ± s.d. of triplicates. Mutants lines were compared to WT, ^*^p=0.010, ^**^p=0.003, ^***^p=0.001.

The activity of proteins involved in executing PCD requires tight regulation, especially when the proteins are basally expressed as PtMCA-IIIc. Therefore, PCD executers are frequently present as inactive zymogens at steady state conditions, and can be rapidly activated by post-translational modifications or translocation. Protein activity is often regulated by reversible Cys oxidation, where the oxidation can induce or inhibit the enzymatic activity. Based on our previous work exposing the redox proteome of *P. tricornutum* (Rosenwasser et al., 2014), we could detect redox-sensitive cysteines in PtMCA-IIIc that showed significant oxidation upon treatment with lethal doses of DD (25μM) and H_2_O_2_ (150μM), in comparison to non-lethal treatments (5μM DD 2h prior to 25μM DD, and no H_2_O_2_; Figure 3; Supplementary Table 1; Rosenwasser et al., 2014). Degree of cysteine oxidation was measured for each detected peptide, and delta oxidation was calculated by subtraction of the oxidation degree of the non-lethal treatments from the lethal treatments. *P. tricornutum* MCPs (PtMCA-a, PtMCA-b) were not detected (Supplementary Table 1), in accordance with their low RNA expression levels, while peptides representing all type III MCs were detected (Figure 3B; Supplementary Table 1). Cys 144 in PtMCA-IIIc and its homologues in PtMCA-IIIa and PtMCA-IIIb were detected, but did not undergo significant oxidation due to lethal treatments (Figures 3, 4A, red frames and Supplementary Table 1). An additional cysteine in PtMCA-IIIc, C202, and its homologues in PtMCA-IIIb were detected in the two redox proteomes. In PtMCA-IIIc, the oxidation of C202 was significantly higher in response to H_2_O_2_ and even higher in response to DD, exhibiting 20.9% more oxidation in the lethal DD treatment (25μM) compared to the non-lethal treatment (5+25μM; Figure 3; Supplementary Table 1). Out of 5 detected cysteines in 3 MCs, only C202 in PtMCA-IIIc was significantly oxidized in response to lethal treatments, suggesting that its oxidation is specific, and has a possible involvement in regulating PtMCA-IIIc activity.

Since C202 is not conserved in other organisms (data from Woehle et al., 2017), and there is no known redox regulation of MC activity in any organism, we performed *in silico* structural based analysis to examine the potential function of the C202. We used the *Saccharomyces cerevisiae* MCA1 (ScMCA-I, a type I MC) structure (Wong et al., 2012) as a basis for the PtMCA-IIIc structural model. The model captured the p20 domain structure (full alignments of the sequences and structures shown in Supplementary Figure 8) and indicated that C202 is distant from the active site, located in the core of the protein 4.1Å apart from another cysteine, C259. This distance is within the range of a reversible disulfide bond (Rubinstein and Fiser, 2008; Sanchez et al., 2008), which upon oxidation may form between the two β-sheets, hence stabilizing the protein (Figure 4B). Notably, this cysteine pair is unique to PtMCA-IIIc, and is absent from the other PtMCAs (Figure 4A) and ScMCA-I (Supplementary Figure 8).

Following these results, we wanted to examine the roles of C202-C259 potential disulfide bond in regulating PtMCA-IIIc activity. Investigation of the suggested disulfide bond between C202 and C259 cannot be done by addition of an oxidant to the protein, as oxidation of the active-site cysteine eliminates MCs activity. It is not uncommon that different cysteines in the same proteins can have different oxidative state at a given time (Rosenwasser et al., 2014; Topf et al., 2018), thus we decided to mutate the putative disulfide-bond forming cysteines. Mutants PtMCA-IIIc^C202S^ and PtMCA-IIIc^C259S^ were generated, in which Cys 202 or 259 were substituted with Ser, thus eliminating the potential formation of the disulfide bond. These mutants were overexpressed in *E. coli* and tested for MC typical activity. In addition, we tested PtMCA-IIIc^C264S^, in which the putative catalytic Cys was mutated. The mutant in the active-site, PtMCA-IIIc^C264S^ exhibited loss of activity as expected, with no apparent autocleavage activity and 3 orders of magnitude lower VRPRase activity compared to the WT (Figures 4C,D). The mutants PtMCA-IIIc^C202S^ and PtMCA-IIIc^C259S^, were still active and were able to undergo autoprocessing (Figure 4C). However, the autocleavage rate was slower, as the ~40 kD band was still apparent after 10min activation and disappeared only after 30min activation (Figure 4C; Supplementary Figure 9). Furthermore, PtMCA-IIIc^C202S^ and PtMCA-IIIc^C259S^ recombinant proteins exhibited lower VRPRase activity compared to the WT (15 and 40%, *p*=0.003, *p*=0.010 respectively, Figure 4D). Thus, this data supports an enhancement of PtMCA-IIIc activity by the suggested disulfide bond between C202 and C259 (herein 2-Cys), while C264 is essential for PtMCA-IIIc proteolytic activity.

### 2-Cys Type III MCs Are Prevalent and Specific to Diatoms

**Figure 5:**
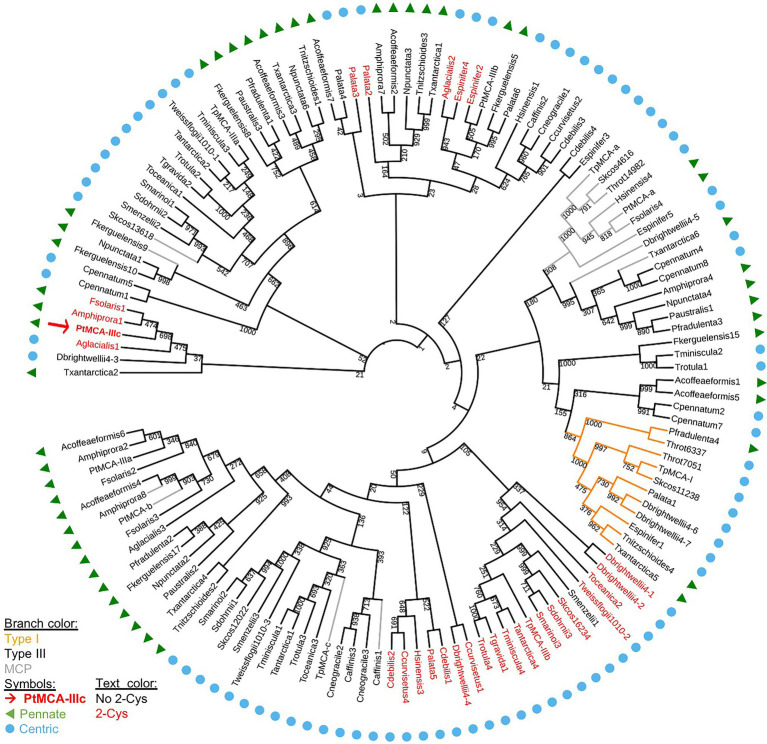
A phylogenetic tree of diatom MCs. A phylogenetic tree of diatom MCs based on protein sequence alignment of the p20 domain retrieved from the MMETSP dataset (Keeling et al., 2014). Type I MCs are marked in orange lines, MCPs are marked in gray lines, and type III MCs are marked in black lines, bootstrap values are indicated. 2-Cys MCs are marked with red text, PtMCA-IIIc is marked with a red arrow and bold text. Pennate diatoms are marked with green triangles, centric diatoms with blue circles. Numbers indicate the MC number, *P. tricornutum* and *T. pseudonana* MCs are numbered according to (Minina et al., 2020).

We aligned the p20 domain across diverse photosynthetic organisms and protist species in order to map the abundance of 2-Cys MCs (Table 1). The 2-Cys were absent in land plants, green and red algae, glaucophytes, cryptophytes, haptophytes and alveolates. In the group of stramenopiles, only diatoms were found to encode for 2-Cys MCs. Importantly, several diatom species with wide global distribution, such as the centric bloom-forming *Skeletonema marinoi* and *Thalassiosira pseudonana* possess 2-Cys MCs (Table 1). Furthermore, we identified 2-Cys MCs in an additional 19 diatom species (Supplementary Table 2) based on the Marine Microbial Eukaryote Transcriptome Sequencing Project (MMETSP; Keeling et al., 2014). The high relative abundance (in 62% of the examined species) of 2-Cys MCs in diatoms of the MMETSP dataset indicates that 2-Cys MCs are both widely distributed in diatoms and widely expressed. Remarkably, all the 2-Cys are type III MCs (Figure 5). Hence, we defined 2-Cys MCs as a novel subtype of type III MCs. Phylogenetic analysis of the 134 diatom MCs, based on the conserved p20 domain, revealed that the 2-Cys were probably acquired in a few independent events (Figure 5; Supplementary Figure 10). 2-Cys MCs seem to be spread across several different clades. The majority (19 MCs) of the 2-Cys MCs are from polar-centric diatoms, and clustered together, suggesting a common origin in this case. Based on metatranscriptome analysis of a natural diatom bloom dominated by *Skeletonema* species and *Thalassiosira rotula* in Narragansett Bay (Alexander et al., 2015), we further confirmed the expression of 2-Cys type III MCs in a natural diatom community (gene IDs 16,234 and 16,314 respectively, Supplementary Figure 11). To summarize, the newly defined 2-Cys MC subtype of type III MCs appears to be diatom specific and is expressed both in laboratory conditions and in natural diatom populations.

**Table 1:** Abundance of 2-Cys MCs across species.

Group	Genus	Species	Number of MCs	Photosynthetic	Endo-symbiosis	Type III	2-Cys
Green algae	*Chlamydomonas*	*reinhardtii*	2	+	1	−	−
	*Volvox*	*carteri*	1	+	1	−	−
	*Chlorella*	*variabilis*	1	+	1	−	−
	*Coccomyxa*	*subellipsodea*	3	+	1	−	−
	*Micromonas*	*pusilla*	1	+	1	−	−
Land plants	*Arabidopsis*	*thaliana*	9	+	1	−	−
	*Oryza*	*sativa*	8	+	1	−	−
Red algae	*Porphyridium*	*purpureum*	2	+	1	−	−
	*Chondrus*	*crispus*	4	+	1	−	−
Glaucophyte	*Cyanophora*	*paradoxa*	3	+	1	−	−
Alveolates	*Symbiodinium*	A1	12	+	2	−	−
	*Plasmodium*	*falciparum*	3	−	2	−	−
Stramenopiles	*Nannochloropsis*	*gaditana*	1	+	2	−	−
	*Aureococcus*	*anophagefferens*	1	+	2	−	−
	*Ectocarpus*	*siliculosus*	4	+	2	+	−
	*Phytophthora*	*sojae*	0	−	2	−	−
Diatoms	*Phaeodactylum*	*tricornutum*	5	+	2	+	+
	*Fragilariopsis*	*cylindrus*	5	+	2	+	−
	*Skeletonema*	*marinoi*	3	+	2	+	+
	*Thalassiosira*	*pseudonana*	6	+	2	+	+
Cryptophyte	*Guillardia*	*theta*	13	+	2	+	−
Haptophyte	*Emiliania*	*huxleyi*	7	+	2	−	−

Number of detected MCs, plastid origin, presence of type III MCs, and 2-Cys MCs are indicated.

## Discussion

In the last two decades numerous studies have reported hallmarks of PCD that are prevalent in a wide range of microorganisms, including bacteria, yeast, protozoans and diverse phytoplankton groups (Golstein et al., 2003; Bidle, 2015; Durand et al., 2016). Though the molecular pathways of PCD have not been characterized in microalgae yet, MCs were suggested as possible PCD regulators, due to their structural similarity to the canonical caspases, which are the metazoan PCD regulators and executers. Furthermore, MCs were shown to participate in PCD activation in plants at different developmental stages (Van Hautegem et al., 2015) as well as in response to biotic stress (Kabbage et al., 2017). To date, although MCs expression was detected in phytoplankton under different environmental stress conditions that can lead to PCD (Bidle and Bender, 2008; Murik and Kaplan, 2009; Thamatrakoln et al., 2012; Orefice et al., 2015; Wang et al., 2018), none of the putative MCs have been functionally characterized.

In this study, we characterized for the first time the biochemical function, regulation and ecophysiological significance of a diatom type III MC that is unique to algae originating from secondary endosymbiosis. Our findings demonstrate that PtMCA-IIIc belongs to a novel subtype of 2-Cys type III MCs that appears to be unique to diatoms and plays a role in cell fate regulation. Recombinant PtMCA-IIIc exhibited calcium dependent MC-typical activity – autoprocessing and cleavage after arginine. In contrast to recombinant PtMCA-IIIc, *P. tricornutum* cells exhibited higher GGRase than VRPRase activity (Figures 1D, 2A), probably due to combined activity of PtMCA-IIIc with additional PtMCAs and other proteases. Accordingly, VRPRase activity, representative of PtMCA-IIIc activity, was enhanced in *P. tricornutum* cells overexpressing PtMCA-IIIc and decreased in PtMCA-IIIc knockout lines (Figure 2D), indicating that part of the MC typical activity detected in *P. tricornutum* cell extracts is indeed the result of the *PtMCA-IIIc* gene product. We also found that MC typical activity (GGRase and VRPRase) was induced during culture aging, suggesting that MCs role depends on the physiological state of the cell. In addition, PtMCA-IIIc KO lines reached lower cell abundances in stationary phase cultures compared to WT or OE lines (Supplementary Figure 7), suggesting a vital role for PtMCA-IIIc in growth phase transition or in population density capacity. These activities are supported by the pro-survival role of MCs in mild stress and aging by clearing protein aggregates in other organisms (Lee et al., 2010; Coll et al., 2014; Mukherjee et al., 2017). Together with the induction in PtMCA-IIIc gene expression (Valenzuela et al., 2012) and general MC-typical activity (Supplementary Figure 5C), these findings implicate an important role of PtMCA-IIIc activity during culture aging.

PCD related proteins require very tight regulation on their activation, and execute cell death only upon requirement. In multicellular organisms, the PCD executing caspases are translated as inactive zymogens, and are activated only by a complex biochemical activation cascade that includes dimerization and cleavage. The plant, fungi and protist homologues, MCs are typically activated by Ca^2+^ binding and autoprocessing, and are active as monomers (Minina et al., 2017). In addition, protein–protein interactions can inhibit type I MCs, and S-nitrosylation of the active site inhibit activation of a type II MC (Belenghi et al., 2007; Coll et al., 2010). Accordingly, recombinant PtMCA-IIIc exhibited Ca^2+^ dependent MC typical activity with a conserved Ca^2+^ binding site in the p20 domain, similar to GtMCA-III (GtMC2), a type III MC from the cryptophyte *Guillardia theta* (Klemenčič and Funk, 2018b). PtMCA-IIIc, as seen in other MCs (Watanabe and Lam, 2011; Fortin and Lam, 2018), may undergo autohydrolysis (see low molecular weight bands Supplementary Figures 3, 9) as a self-inactivation mechanism, that ensures that the activated PtMCA-IIIc will have a short functional half-life.

Importantly, we identified another layer of post-translational regulation, novel in MCs, through reversible oxidation of reactive regulatory cysteines. By combining data from redox proteomics with a 3D protein model and directed point mutations, we suggest that oxidation of C202, as detected in response to lethal treatments (Figure 3), forms a stabilizing disulfide bond with C259, that enhances PtMCA-IIIc activity. Mutations in either one of the 2-Cys decreased PtMCA-IIIc activity to 15–40% of WT activity, but did not abolish it completely (Figure 4D). In contrast, oxidation of the active site cysteine inactivates plant MCs (Minina et al., 2014). Under such scenario, only mild oxidative stress caused by specific environmental conditions could lead to specific oxidation of these regulatory Cys, without oxidizing the active site, thus enhancing PtMCA-IIIc activity. This tight regulation of protein activity at the post-translational level can allow basal expression with rapid activation only in the right conditions without requiring *de-novo* protein synthesis.

We suggest a that optimal activation of PtMCA-IIIc requires the combination of two signals, Ca^2+^ and mild oxidative stress. The oxidative stress can be generated as an enzymatic byproduct of Ca^2+^ signaling (Bowler and Fluhr, 2000) or as a direct result of an environmental stress (Graff van Creveld et al., 2015). Diverse environmental stresses, including diatom-derived infochemicals such as DD, are perceived by the induction of Ca^2+^ intracellular transients within seconds (Falciatore et al., 2000; Vardi et al., 2006). Subsequently, Ca^2+^ can bind to the MCs Ca^2+^ binding site, which is necessary for MC activation (McLuskey et al., 2012; Klemenčič and Funk, 2018b; Figure 1D; Supplementary Figure 4A). In addition, DD leads to Ca^2+^ transients followed by a ROS burst in the mitochondria (Vardi et al., 2008; Graff van Creveld et al., 2015). This ROS accumulation is essential for PCD induction, as addition of antioxidants can prevent subsequent cell death (Graff van Creveld et al., 2015; Volpert et al., 2018; Mizrachi et al., 2019). Sublethal ROS levels can act as a signal and oxidize the 2-Cys to form a disulfide bond, which induces PtMCA-IIIc activity. These rapid post-translational modifications lead to activation of pre-existing PtMCA-IIIc protein, and execution of a PCD pathway. Only integration of the two signals, Ca^2+^ and specific ROS levels, leads to sufficient activity of 2-Cys MCs, decreasing the chance of accidental activation, which may cause unnecessary cell death. The downstream events that follow PtMCA-IIIc activation are yet to be characterized. Identification of PtMCA-IIIc natural substrate may reveal further steps in the diatom PCD cascade.

Recent phylogenomic analysis tracked the evolutionary history of the redox-sensitive Cys residues in *P. tricornutum*, revealing its expansion during plastid evolution (Woehle et al., 2017). Interestingly, the unique presence of the 2-Cys in diatom MCs but not in closely related groups (Table 1), suggests a late Cys gain in evolution. Importantly, 2-Cys MCs were expressed in cultures as well as in a natural diatom bloom (Supplementary Figure 11). Using a redox-sensitive GFP probe, it was demonstrated *in vivo* that early oxidation of the *P. tricornutum* mitochondrial glutathione pool in response to DD led to PCD in a dose-dependent manner (Graff van Creveld et al., 2015). Redox-regulation on MCs activity *via* reactive Cys can allow diatoms to integrate various environmental signals in the marine environment and rapidly adjust cellular processes in a reversible manner until reaching the point-of-no-return in which it is involved in the activation of the PCD cascade. Future studies will help to elucidate the ecophysiological importance of PCD-dependent mortality of phytoplankton blooms and turnover of carbon in the ocean.

## Data Availability Statement

The original contributions presented in the study are included in the article/Supplementary Material, further inquiries can be directed to the corresponding author.

## Author Contributions

SGvC, AM, SR, and AV designed the research, analyzed the data, and wrote the article with contributions of all the authors. SGvC and AM conducted the experimental work. SB-D and SGvC conducted the bioinformatics analysis. UA designed and assisted with the biochemistry experiments and protein modeling. SGvC, AH, and TM designed the gene knockout. TM and AH provided the plasmids for knockout plasmids cloning. SGvC and SR conducted and analyzed the redox proteomics. All authors contributed to the article and approved the submitted version.

## Funding

This research was supported by the Israel Science Foundation (ISF; grants # 712233, # 1972/20) awarded to AV.

## Conflict of Interest

The authors declare that the research was conducted in the absence of any commercial or financial relationships that could be construed as a potential conflict of interest.

## Publisher’s Note

All claims expressed in this article are solely those of the authors and do not necessarily represent those of their affiliated organizations, or those of the publisher, the editors and the reviewers. Any product that may be evaluated in this article, or claim that may be made by its manufacturer, is not guaranteed or endorsed by the publisher.

## Footnotes

^1^ http://swissmodel.expasy.org

^2^ www.broadinstitute.org/rnai/public/analysis-tools/sgrna-design

^3^ https://bioinformatics.psb.ugent.be/plaza/versions/pico-plaza/

^4^ https://itol.embl.de/
