# Supplementary material for: Biochemical Characterization of a Novel Redox-Regulated Metacaspase in a Marine Diatom
Source: Front Microbiol. 2021 Sep 8;12:688199. doi: 10.3389/fmicb.2021.688199 (PMC8455989; doi:10.3389/fmicb.2021.688199)
Supplement: Supplementary file 1 [file Data_Sheet_1.pdf]

Supplementary Material

Article title:

Biochemical characterization of a novel redox-regulated metacaspase in a marine diatom

Authors: Shiri Graff van Creveld, Shifra Ben-Dor, Avia Mizrachi, Uria Alcolombri, Amanda Hopes, Thomas Mock, Shilo Rosenwasser, and Assaf Vardi

The following Supplementary Materials is available for this article:

Supplementary Figure 1 *P. tricornutum* MCs expression in published transcriptomes.

Supplementary Figure 2 PtMCA-IIIc protein sequence and conserved features.

Supplementary Figure 3 Full membranes and gels used to reconstruct Figure 1C.

Supplementary Figure 4 PtMCA-IIIc *in vitro* activity require mM of Ca^{2+} and DDT.

Supplementary Figure 5 PtMCA-IIIc and *P. tricornutum* protease activity in response to caspase inhibitor and during a growth curve.

Supplementary Figure 6 Schematic representation of assembly, final transformation vectors for PtMCA-IIIc knockout and overexpression, and the exact deletion in the DNA and protein sequence.

Supplementary Figure 7 Growth curve of *P. tricornutum* cells with PtMCA-IIIc overexpression or knockout.

Supplementary Figure 8 Protein sequence and structure of PtMCA-IIIc aligned to ScMCA-I.

Supplementary Figure 9 Full membranes used to reconstruct Figure 4C, and similar membranes following longer activation of PtMCA-IIIc.

Supplementary Figure 10 Protein sequence alignment of the p20 domain of diatoms 2-Cys MCs, and *S. menzelii* MC1.

Supplementary Figure 11 Expression of 2-Cys MCs in a natural diatoms bloom.

Supplementary Table 1 All PtMCAs peptides detected in redox proteomics in response to lethal treatments of DD or H_2O_2 in compare to non-lethal treatments

Supplementary Table 2 Abundance of 2-Cys MCs across diatom species.

Supplementary Table 3 A list of primers used in this study.

Supplementary Methods Identifying MC genes from various species.

Supplementary Data 1 Full protein sequences of all the MCs used in this study.

Supplementary Data 2 Alignment of the p20 domains of diatoms MCs, used to generate the phylogenetic tree in Figure 5.

Supplementary Figure 1

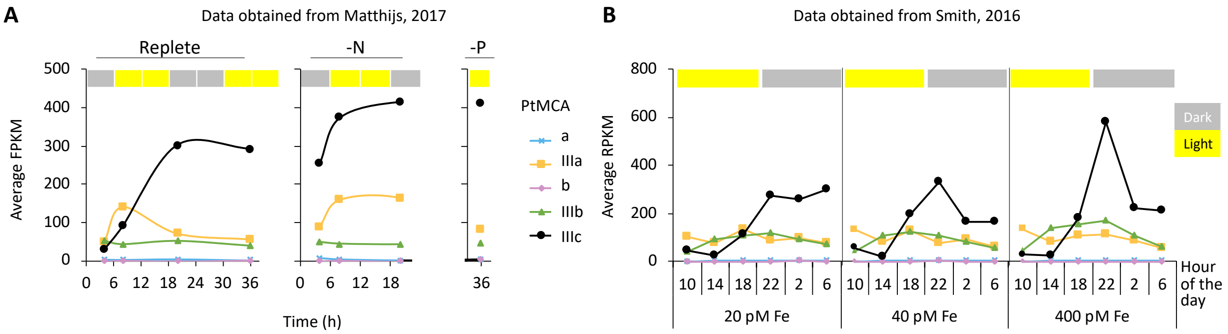

P. tricornutum MCs expression in published transcriptomes.

(A) *P. tricornutum* MCs expression, presented in fragments per kilobase of exon per million fragments mapped (FPKM) as obtained from (Matthijs et al., 2017) during 36 h growth in replete, nitrogen (-N) or phosphate (-P) deplete media. **(B)** *P. tricornutum* MCs expression, presented in reads per kilobase of transcript per million (RPKM) values as obtained from (Smith et al., 2016) after 30 generation acclimation to 20, 40 or 400 pM Fe, during 20 h growth diel cycle. Light regime is indicated in yellow (light) or gray (dark). PtMCA-IIIc is represented in black lines.

Supplementary Figure 2

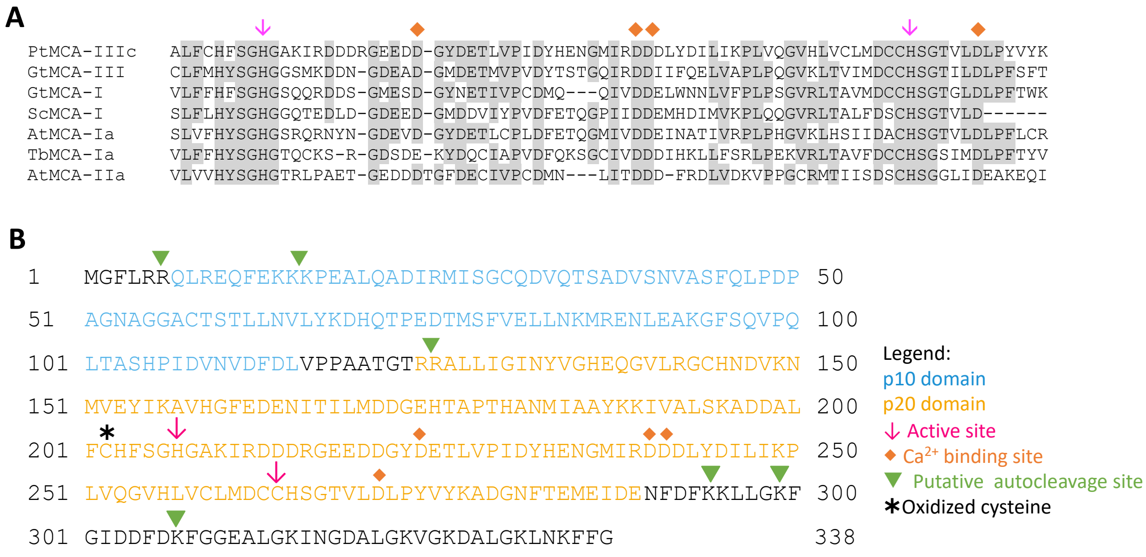

PtMCA-IIIc protein sequence and conserved features.

(**A**) Partial alignment of PtMCA-IIIc with MCs showing the conserved Ca^{2+} binding sites (orange rhombuses), and active dyad (magenta arrows). Gray background represents above 50% similarity.

(**B**) PtMCA-IIIc protein sequence and conserved features.

Supplementary Figure 3

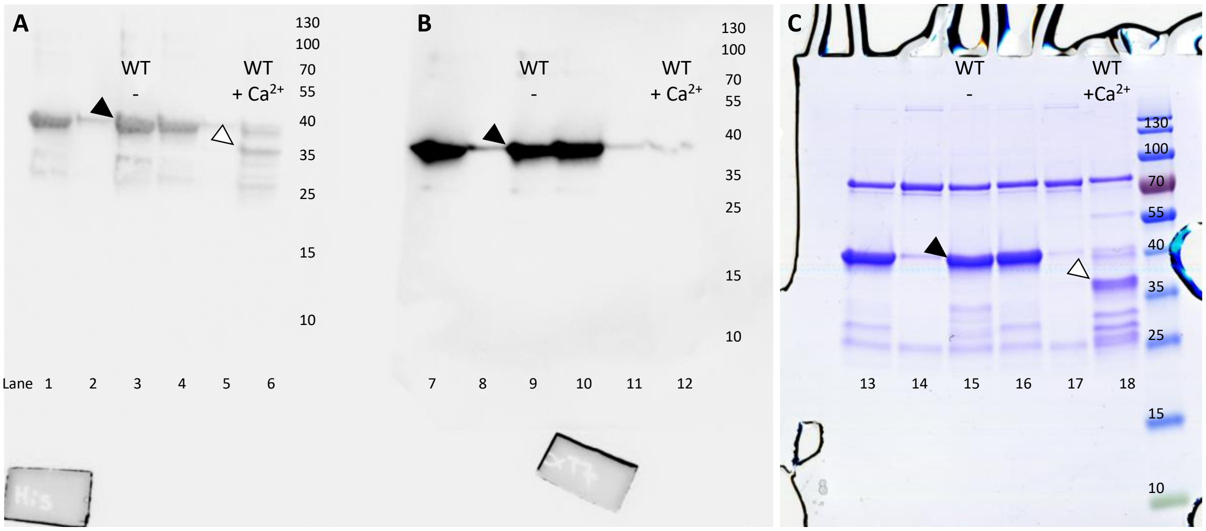

Full membranes and gels used to reconstruct Figure 1C.

Immunoblot with (HRP) αHis (**A**) or αT7 (**B**) tags, and Coomassie stained SDS-page (**C**). About 2.5 µg protein extracts per lane were incubated with activity buffer containing 10 mM DTT, 0.1% CHAPS and without / with 10 mM $CaCl_2$ for 30 min prior to gel loading. Full length PtMCA-IIIc (~40 kD), auto-cleaved PtMCA-IIIc (~37 kD) were detected. Lanes 1, 2, 4, 5, 7, 8, 10, 11, 13, 14, 16 and 17 are not discussed in this manuscript. Protein ladders are present in the right, size in kD.

Supplementary Figure 4

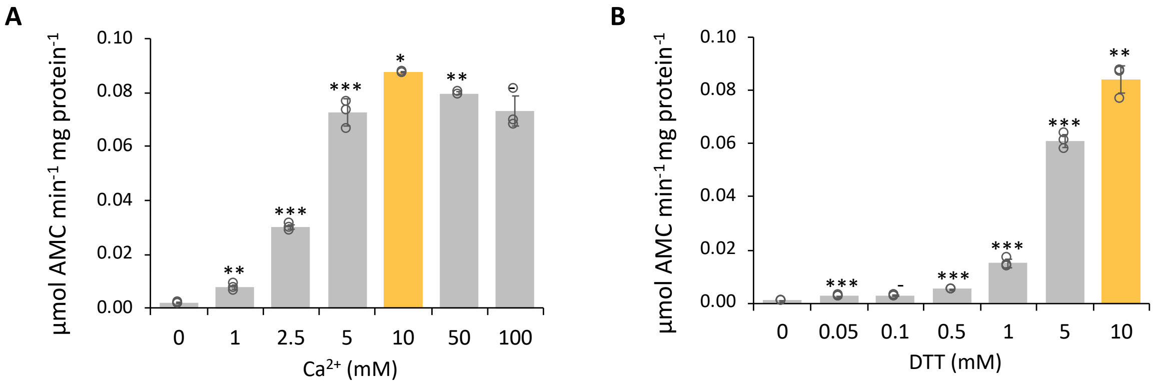

PtMCA-IIIc *in vitro* activity require mM of Ca^{2+} and DDT.

Recombinant PtMCA-IIIc protease activity, measured as release of AMC from GGR-AMC in increasing concentrations of Ca^{2+} (**A**) or DTT (**B**). In orange marked 10 mM (Ca^{2+} or DTT), as this concentration was used in the rest of the manuscript. Standard curve was used to convert the relative fluorescence units into µmole of free AMC released per min per mg of total protein. Single measurements are indicated in circles, bars are means ± s.d. of triplicates, each concentration was compared to 1 lower concentration, -P>0.05, *P<0.05, **P<0.005, ***P<0.001.

Supplementary Figure 5

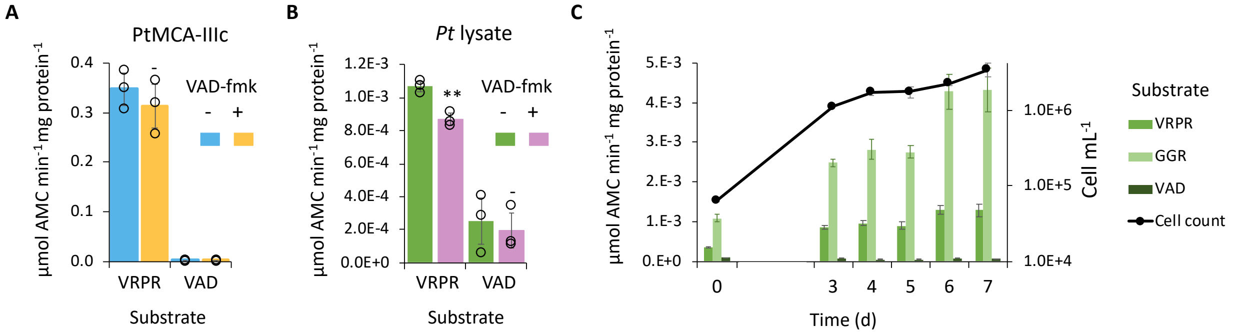

PtMCA-IIIc and *P. tricornutum* protease activity in response to caspase inhibitor and during a growth curve.

Recombinant PtMCA-IIIc (**A**), *P. tricornutum* protein extract (**B**), protease activity measured as release of AMC from peptidyl substrates, with or without 100 µM of the pan-caspase inhibitor VAD-fmk. Standard curve was used to convert the relative fluorescence units into µmole of free AMC released per min per mg of total protein. Single measurements are indicated in circles, bars are means ± s.d. of triplicates, significant relative to no inhibitor, -*P*>0.05, ***P*=0.004. (**C**) Protease activity of *P. tricornutum* protein extracts measured as in (**B**) (primary Y axis). Cell abundance (secondary Y axis) measured during 8 days of growth. Points are means ± s.d. of triplicates.

Supplementary Figure 6

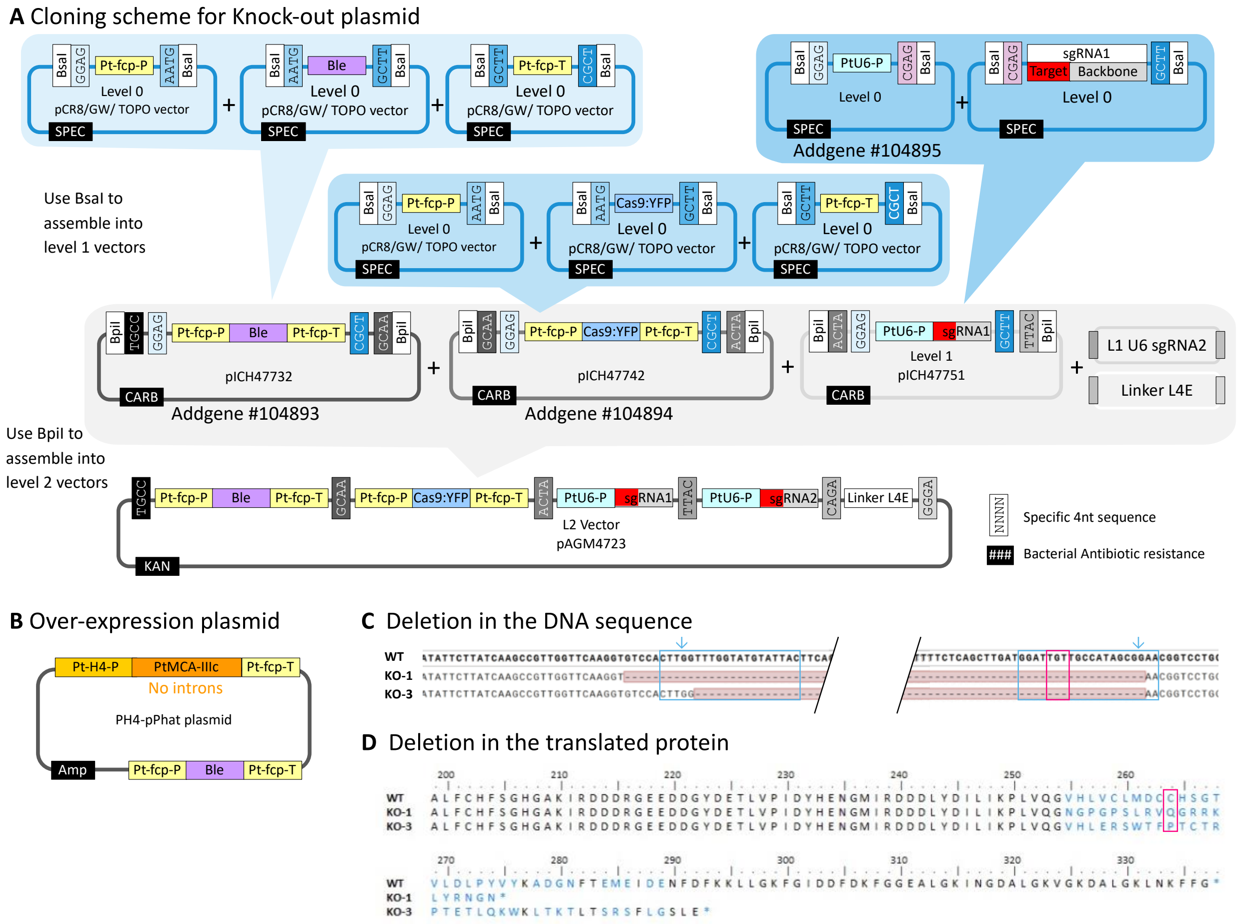

Schematic representation of assembly, final transformation vector for PtMCA-IIIc knockout and overexpression, and the exact deletion in the DNA and protein sequence.

(**A**) Schematic view of Golden Gate assembly for CRISPR/Cas9-mediated PtMCA-IIIc KO vector. GG 4 nt sites are marked in colored boxes, bacterial antibiotic resistance, Spec, Carb or Kan are marked in black boxes. (**B**) Schematic representation of *P. tricornutum* transformation vectors used for PtMCA-IIIc overexpression, including bacterial antibiotic resistance (ampicillin resistance), diatom antibiotic resistance (Bleomycin resistance under *P. tricornutum* Fcp promoter and terminator) and His-tagged PtMCA-IIIc cDNA under histone 4 promoter (H4) and Fcp terminator. (**C**) DNA sequence of clones KO1 and KO3 aligned to the WT *PtMCA-IIIc* gene. The sgRNAs are indicated in blue frame, the expected sgRNA cut positions are indicated by blue arrows. The active-site (C264) is marked in magenta frame. (**D**) Translated protein, based on DNA sequence, of WT and the two KO lines, starting from amino acid 199. The active-site (C264) location is marked in magenta frame, frameshifts are marked in bold blue text.

Supplementary Figure 7

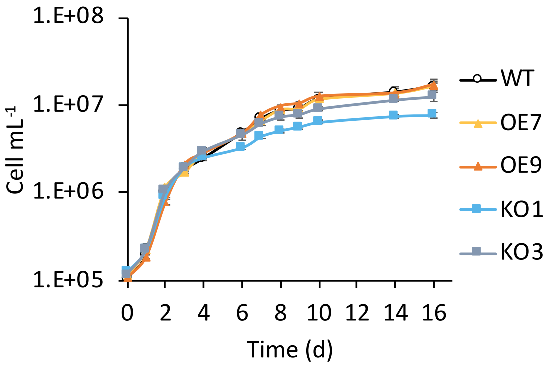

Growth curve of *P. tricornutum* cells with PtMCA-IIIc overexpression or knockout.
Growth curve of *P. tricornutum* WT, overexpression (OE7, 9) and knockout (KO1, 3) lines over 16 days, presented as means ± s.d. of triplicates. Flow cytometry, analysis is based on fluorescent measurements of at least 10,000 cells per sample.

Supplementary Figure 8

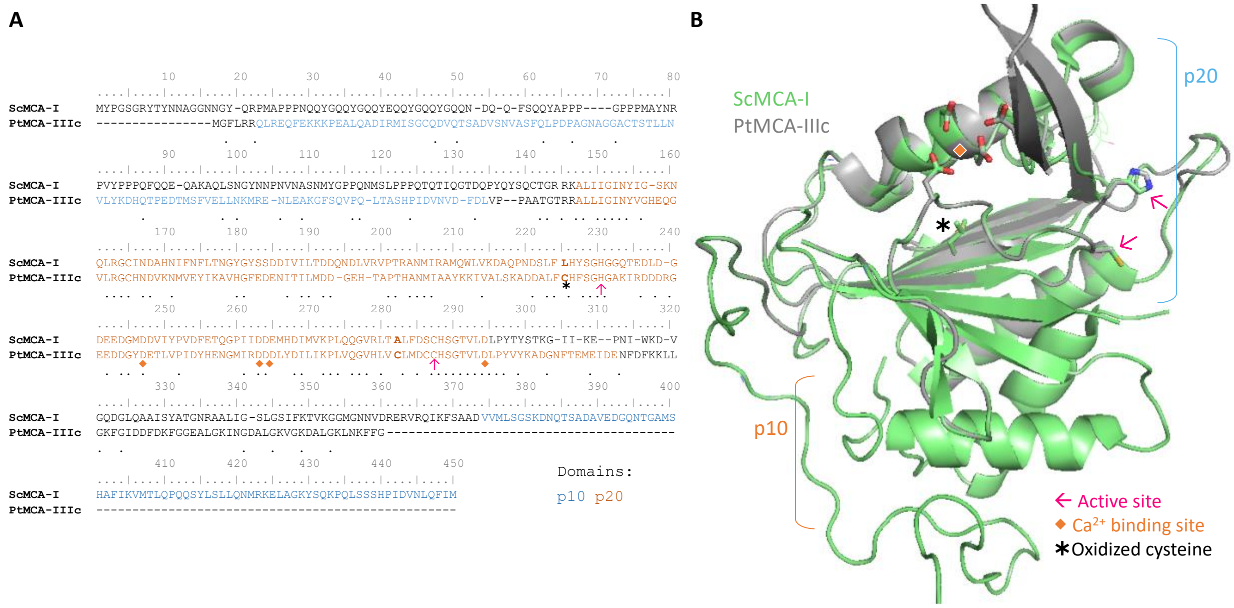

Protein sequence and structure of PtMCA-IIIc aligned to ScMCA-I.
(**A**) Protein sequence alignment of *S. cerevisiae* MC (MCA1) and PtMCA-IIIc. The p20 domains marked in blue, the p10 domains in orange. identical amino acids are marked with dots. (**B**) Cartoon representation of ScMCA-I structure (Wong et al., 2012) (green) aligned with PtMCA-IIIc (gray), practically only the p20 domain is modeled. Key amino-acids side chains presented as sticks with conventional coloring, oxygen in red, nitrate in blue and sulphur in yellow. The active site is marked in magenta arrows, Ca^{2+} binding site is marked with orange rhombus, and C202 in PtMCA-IIIc, detected as oxidized in lethal stresses marked in black asterisk.

Supplementary Figure 9

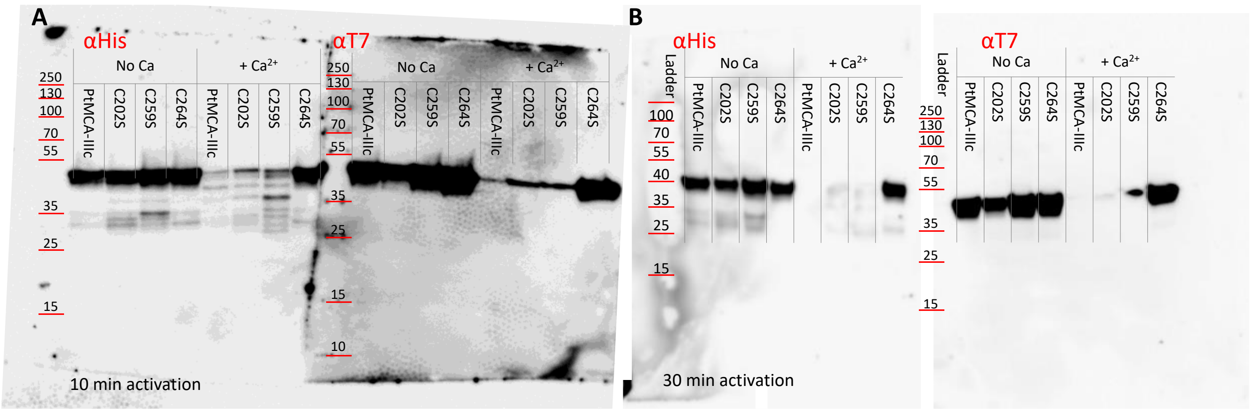

Full membranes used to reconstruct Figure 4C, and similar membranes following longer activation of PtMCA-IIIc.

Immunoblot with (HRP) αHis or αT7 tags of PtMCA-IIIc, PtMCA-IIIc^{C202S}, PtMCA-IIIc^{C259S}, and PtMCA-IIIc^{C264S}. Similar amounts of protein extracts per lane were incubated with activity buffer containing 10 mM DTT, 0.1% CHAPS and without / with 10 mM $CaCl_2$ prior to gel loading. (**A**) Full membranes used to reconstruct Figure 4C, ~0.88 µg protein per lane were incubated for 10 min in activity buffer. (**B**) About 4 µg protein per lane were incubated for 30 min in activity buffer. Protein ladders are presented in the left of each membrane, size in kD.

Supplementary Figure 10

```
C.debilis1              KRAVLIGINYVGQ-EGELSGCHNDVIRIKDYLINFQGFEERHITLLMDDGININPTKGRIIRAYRRIVKVSKAGDTVF
P.alata5                RRAVLIGINYVGQ-SGELSGCHNDVFNVKDYLVNVLGFENRHITLLLDDGLNTSPTRENITYAYRDLVNRSAPGDVAF
C.curvisetus4           KRAVLIGINYVGQ-NGELSGCHNDVKNMKEYLMNVHGFPESNMTVLMDDGYHLNPTRYNITNAYQNLVRQSMPGDSVF
C.debilis2              KRAVLIGINYTGQ-QGELSGCHNDVKNMKEYLITVHGFEEQNMLILMDDNYHPNPTRMNVLNAYRNLVRESKPGDTAF
H.sinensis3             RRAVLIGINYVGQ-QGQLSGCHNDVKNMKKYLMQVHGFEEHNMTILMDDGYHREPTYRNIVNAYKDVVRQSLPGDTVF
C.curvisetus1           KRAVLVGINYVGL-PGQLSACHYDVKKVKEYLITELGFEEKNMIILMDDDHHTRPTKYAILTAYRRLVEESKAGDTVY
D.brightwellii4-4       KRALLIGINYVGQ-NGELSGCHNDVLNIKEYLMDVLGFEEDNIMVLMDDGIHHEPTRDSILSGYRRLVAESVAGDTVF
S.dohrnii3              QRAVLIGINYKGQ-KGQLSGCHNDVHNVTQYLKEVQGFKDENITILMDDGMHKPPTKSAIISAFKRLVKQTKEGDVVF
S.marinoi3              QRAVLIGINYKGQ-KGQLSGCHNDVHNVTQYLKEVQGFKDENITILMDDGMHKPPTKSAIISAFKRLVKQTKEGDVVF
Skcos16234              QRAVLIGINYKGQ-KGXLSGCHNDVHNVTXYLKEVQGFKDENITILMDDGMHKPPTKXAIISAFKRLVKQTKEGDVVF
T.antarctica4           QRAVLIGINYTGQ-SGELSGCHNDVHNVAKYLMEVQGFKKENVTILMDDGAHKPPTKAGIVNAYKRLVKESKEGDVAF
T.miniscula4            QRAVLIGINYTGQ-SGELSGCHNDVHNVARYLTEVQGFRKENVTILMDDGMHKAPTKSAILGAYKRLVKESREGDVVF
T.gravida1              KRAVLIGINYTGQ-KGELSGCHNDVKNVARYLSEVQGFKKENVTILMDDGNHKPPTKTAIISAYKKLVKESKKGDVVF
T.rotula4               KRAVLIGINYTGQ-KGELSGCHNDVNNVARYLSEVQGFKKENVTILMDDGNHKPPTKSAIISAYKKLVKESKKGDVVF
TpMCA-IIIb              QRAVLIGINYVGQ-KGQLSGCHNDVLNVAKYLKEVQGFRKENMTILMDDGNHKSPTKSAILSAYKKIVKESKEGDVVF
T.weissflogii1010-2     KRAVLIGINYTGQ-SGELSGCHNDVKNISKYLREVQGFKKENVTILMDDGQHKEPTKANIIASYKKLVKDCKSGDVVF
T.oceanica2             KRAVLIGINYTGT-PGELRGCHNDCLNVARFLRE-QGFRDENVTMLLDDNKHRSPTKAAILSAYKKLVRESKPGDVVF
D.brightwellii4-1       KRALLIGINYTGMQPGELTGCHNDVKNVKEYLMNEHSFKEENITVMLDDNVHESPTKANIISGYKKLTKESQPGDAVF
D.brightwellii4-2       RRAVLIGINYTGMNPGALTGCHNDVHNIKHYIMDVWGFKEEHITVLLDDGKHKPPTRANILGAYAALAAKCQPGDVAF
PtMCA-IIIc              RRALLIGINYVGHEQGVLRGCHNDVKNMVEYIKAVHGFEDENITILMDDGEHTAPTHANMIAAYKKIVALSKADDALF
A.mphiprora1            RRALLIGINYIGHSQGVLSGCHNDVLNMVEYIKEVHGFEEDSITVLLDDGEHDAPTKENILAAYKKMVAEAEPGDSLF
F.solaris1              KRAVLVGINYIGHSQGVLSGCHNDVKNMVDYIKDVHGFEEQNITVIMDDGEHPEPTKANIVAAYKKLIEESQPGDCLF
A.glacialis1            RRALLIGINYVGHESGVLSGCHNDALNMKEYIMDVHGFEEENITLLLDDGEHDPPTKENILNAYKKLVEESEKGDSVF
A.glacialis2            KRALLIGINYIGQ-QGELSGCHNDVLNMVEYLKDVWGFEEHNITILLDDGEHTNPTRSNILSAYQQIVAEAESGDAVY
E.spinifer4             KRALLIGINYVGQ-QGQLSGCHNDVLNMVEYLKDVWGFPPQNITVLLDDGSHVNPTRSNILNAYRTLVAESQPGDAVY
E.spinifer2             RRALLIGINYIGQ-QGELSGCHNDVMNIKEYIIDVHGFPEENITILLDDGEHRDPTRENILDAYKALVSASKAGDACF
P.alata2                KRALLIAINYVGQ-KGELTGCINDALNMKKYIMDVWGFEEENITMIMDDGEHMEPTRENILNAYNDLVIQSEAGDSIF
P.alata3                KHALLIGINYVGQ-SGELSGCHNDVWNIRSYLIDVHGFEERYITILMDDGSHTSPTRENIMSAFRMLVKSSVGGDSAF
S.menzelii1             QRALLIGINYKGQ-TGQLSGCHNDVHNVSQYLQEVQGFKEDNITMLMDDGMHKPPTKSAIISAFKRLVKQTKEGDVVF
                        ::*:*:.*** *   * * .*  *  *:  ::    .*    : :::**. :  **    :  .:  :      .*  :

C.debilis1              CHYSGHGGRTKDLDG-DESDGLDETLIPMDYQSAGQIIDDDLFKELVQPMSEGVHATCLMDCCHSGTVLDLPYHFTAE
P.alata5                CHYSGHGGFVDDTSG-DEEDGRDETLIPVDFQSAGQITDDELLKDLVHPMKAGVLTTCLMDCCHSGTVMDLPYRFTAD
C.curvisetus4           CHYSGHGGRVRDTSG-DEDDGFDETLIPVDFQSSGQIIDDDLFKNLVQPLPKGVLMTCLMDCCHSGTVLDLPYRFTAD
C.debilis2              CHYSGHGGSVKDYSG-DEEDGFDETLIPVDFQRSGQIIDDDLFKELVRPMSTGVLMTCLMDCCHSGTVLDLPYRFTAD
H.sinensis3             CHYSGHGGSVRDASG-DEEDGYDETLIPVDFQTAGQILDDYLLENLVKPLSRDVLMTCLMDCCHSGTVLDLPYRFTAD
C.curvisetus1           CHFSGHGSRVVDTNG-DEDDGYDETLIPVDFYRAGQIVDDDLLKLFVKPMAKGVLMTCMFDCCHSGTVLDLPYYFNAD
D.brightwellii4-4       CHYSGHGGRLVDDDG-DEDDGYDETLIPVDFEHAGQIRDDVLFNEFVRPMAEGVTVTCLMDCCHSGTVLDLPYRFVAD
S.dohrnii3              CHYSGHGGRLPDDNG-DEDDGYDETLIPLDFQKSGHIRDDDLLKILIHPMPAKVTMTCLMDSCHSGTVLDLPYRFVGD
S.marinoi3              CQYSGHGGRLPDDNG-DEDDGYDETLIPLDFQKSGHIRDDDLLKILIHPMPAKVTMTCLMDSCHSGTVLDLPYRFVGD
Skcos16234              CQYSGHGGRXPDDNG-DEDXGYDETLIPLDFQKSGHIRDDDLLKILIHPMPAKVTMTCLMDSCHSGTVLDLPYRFVGD
T.antarctica4           CHYSGHGGRLPDDNG-DEEDGYDETLIPVDYDKKGQIRDDDLLKILVHPLQEGVTMTCLMDCCHSGTVLDLPYRFTSD
T.miniscula4            CHYSGHGGRLPDDNG-DEEDGYDETLIPVDYDKKGQIRDDDLLKILIHPMPKGVTMTCLMDCCHSGTVLDLPYRFTAD
T.gravida1              CHYSGHGGRLPDDNG-DEEDGYDETLIPVDFEKRGQIRDDDLLKILVSPMSEGVTMTCLMDCCHSGTVLDLPYRFTSD
T.rotula4               CHYSGHGGRLPDDNG-DEEDGYDETLIPVDFEKRGQIRDDDLLKILVGPMPEGVTMTCLMDCCHSGTVLDLPYRFTSD
TpMCA-IIIb              CHYSGHGGRIKDDNG-DEDDGHDETLIPVDFEKAGQIRDDDLLKILVHPMAAGVRMTCCMDCCHSGTVLDLPYRFTAD
T.weissflogii1010-2     CHYSGHGGRLPDDNG-DEDDGHDETLIPVDFEKNGQIRDDDLLNMLVNPMPKGVTMTCLMDCCHSGTVLDLPYRFTAD
T.oceanica2             CHYSGHGGRLPDDNG-DEDDGWDETLIPVDFKTAGQIRDDDLFKFLVHPMPANVTMTCLMDCCHSGTVLDLPYRFTAD
D.brightwellii4-1       CHFSGHGSQIKDDDWFEEEDGLDETLVPVDFKENGQIRDDDIFDILVVPMKEGVTLTCFFDCCHSGTVMDLPFTYRAD
D.brightwellii4-2       CHYSGHGGMLPDDNG-DEDDGFDETLIPLDFKRTGQIRDDDLFKILVVPMKAGVTLTCLFDCCHSGTVLDLPYNFQAD
PtMCA-IIIc              CHFSGHGAKIRDDDRGEEDDGYDETLVPIDYHENGMIRDDDLYDILIKPLVQGVHLVCLMDCCHSGTVLDLPYVYKAD
A.mphiprora1            CHYSGHGCKIRDDDRGEEEDGFDEALVPVDYQTAGLIRDDDLFDIIIKPMPDGANLFCLFDCCHSGTILDLPYLYKAD
F.solaris1              CHYSGHGAKIKDDEQGEEKDGYDEVLVPVDYEQAGMIRDDDLFDILIRPMQQGVSLFCLMDCCHSGTVMDLPYIFKAD
A.glacialis1            CHYSGHGGSTRDDDRGEEEDGKDETLIPVDYKTAGIIRDDDLFDTLVHPMKEGVTLTCLMDCCHSGTILDLPYTFKAD
A.glacialis2            CHYSGHGGRLRDDD-GDEEDGYDETLVPLDYQRAGQIRDDDLFNILVGPMKAGVVLTCVMDCCHSGTVLDLPYCFVAD
E.spinifer4             CHYSGHGGKLRDDD-GDEKDGYDETLVPLDYQSAGQIRDDDLFNTLVGPMKKGVQATCIMDCCHSGSILDLPYVFKAD
E.spinifer2             CHYSGHGGKLRDDD-GDEADGYDETLVPLDYQRAGQIRDDDIFNTLIGPMPKGVTLTCIMDCCHSGTVLDLPYVFKAD
P.alata2                CHYSGHGGKLRDDN-GDEEDGYDETLVPVDYARSGQIRDDDLYEILVGCMPRGVYMTCLMDCCHSGTVLDLPYKFVAP
P.alata3                CHYSGHGGKIRDNN-NDESDGYDETLVPLDHYASGQIRDDDVYDALVGPMPSNVTLTCLMDCCHSGTVLDLPYKFKPD
S.menzelii1             FHYSGHGGRLPDDN-GDEDDGFDETLIPLDFKKSGQIRDDDLLKILVHPMPANVSMTCLMDCCHSGTVLDLPYRFVGD
                        *::***     *  .  :*  * *..*:*:*.    * * ** : . ::  :    .    *  :*.****:::***: :

2 Cysteines
Active site
```

Protein sequence alignment of the p20 domain of diatoms 2-Cys MCs, and *S. menzelii* MC1
PtMCA-IIIc is marked in bold, active site marked in red, 2-Cys in blue.

Supplementary Figure 11

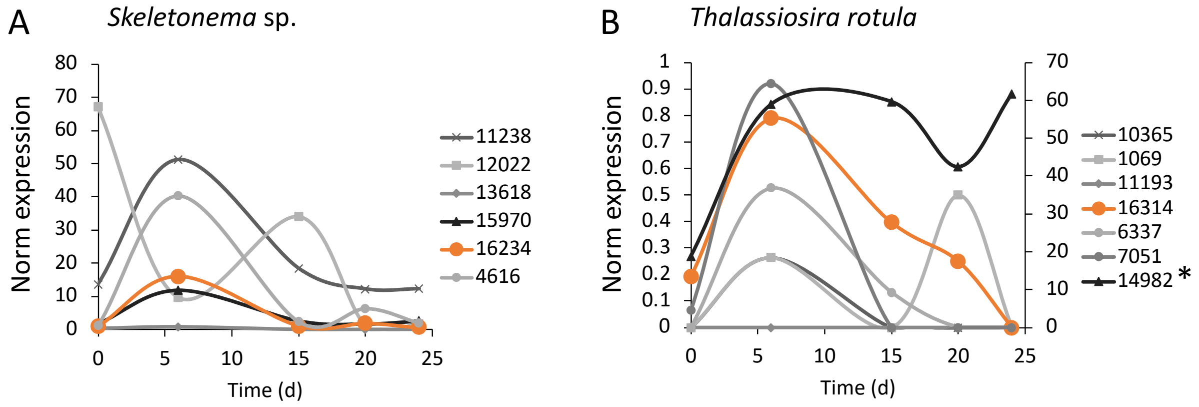

Expression of 2-Cys MCs in a natural diatoms bloom.

MCs relative expression from a metatranscriptome of *Skeletonema* (**A**) and *Thalassiosira* (**B**) natural bloom in Narragansett Bay, data obtained from (Alexander et al., 2015). Relative expression of MCs in the natural bloom that was sampled for 24 days. 2-Cys MCs are marked in orange. * 14982 is plotted on the secondary Y axis.

Supplementary Table 1 All PtMCAs peptides detected in redox proteomics in response to lethal treatments of DD or H_2O_2 in compare to non-lethal treatments.

PtMCA	Cysteine	Peptide (Cys)	H_2O_2 (µM)		DD (µM)							
			0	150	5 + 25	5 + 25	5 + 25	5+25 avg	25	25	25	25 avg
III-a	134	AVL IGINYTGQQGQLSGCHNDVK	0.082	0.106				ND				ND
III-b	135	AVMIGINYTGQQGQLSGCHNDVK	0.135	0.175				ND				ND
III-c	144	GCHNDVK	ND	ND	0.285	0.324	0.584	0.398	0.419	0.538	0.557	0.504
III-b	193	AGDAIFCHYSGHGGK	0.226	0.139	0.202	0.234	0.424	0.287	0.311	0.397	0.305	0.338
III-c	202	ADDALFCHFSGHGAK	0.272	0.340	0.214<br>0.182	0.197<br>0.215	0.142<br>0.183	0.189	0.387<br>0.361	0.424<br>0.421	0.383<br>0.413	0.398

In (Rosenwasser et al., 2014), *P. tricornutum* cells were treated with 150 µM H_2O_2 (lethal), treated and untreated cells were sampled after 20 min. In addition, *P. tricornutum* cells were sampled 2.5 h after treatment with 25 µM DD, with (non-lethal) or without (lethal) pre-treatment of 5 µM DD 4.5 h before sampling. PtMCA number, Cys number, and detected peptide (detected Cys marked in bold red) are shown. Average degree of oxidation in each H_2O_2 treatment (Rosenwasser et al., 2014), and individual and average degree of oxidation in each DD treatment are shown. ND - not detected.

Supplementary Table 2 Abundance of 2-Cys MCs across diatoms species

Genus	Species	Isolate	#detected MCs	#2-Cys MCs	Classification
Amphiprora	*sp.*	CCMP467	5	1	Raphid Pennate
Amphora	*coffeaeformis*	CCMP127	7	-	Raphid Pennate
Asterionellopsis	*glacialis*	CCMP134	3	2	Raphid Pennate
Chaetoceros	*affinis*	CCMP159	3	-	Polar Centric
	curvisetus	Unknown	3	2	
	debilis	MM31A-1	4	2	
	neogracile	CCMP1317	3	-	
Corethron	*pennatum*	L29A3	6	-	Radial Centric
Ditylum	*brightwellii*	GSO104	7	3	Polar Centric
Extubocellulus	*spinifer*	CCMP396	5	2	Polar Centric
Fistulifera	*solaris*	JPCC DA0580	4	1	Raphid Pennate
Fragilariopsis	*kerguelensis*	L26-C5	6	-	Raphid Pennate
	cylindrus	CCMP1102	5	-	
Hemiaulus	*sinensis*		3	1	Polar Centric
Nitzschia	*punctata*	CCMP561	5	-	Raphid Pennate
Phaeodactylum	*tricornutum*	CCMP2561	5	1	Raphid Pennate
Proboscia	*alata*	PI-D3	6	3	Radial Centric
Pseudo-nitzschia	*australis*	10249 10 AB	3	-	Raphid Pennate
	fraudulenta	WWA7	4	-	
Skeletonema	*costatum.*	Skcos16234	5	1	Polar Centric
	dohrnii	SkelB	3	1	
	marinoi	SkelA	3	1	
	menzelii	CCMP793	3	-	
Thalassionema	*nitzschioides*	L26-B	4	-	Araphid Pennate
Thalassiosira	*rotula*	CCMP3096 (+Throt)	7	1	Polar Centric
	antarctica	CCMP982	3	1	
	gravida	GMp14c1	2	1	
	miniscula	CCMP1093	4	1	
	oceanica	CCMP1005	3	1	
	pseudonana	CCMP1335	6	1	
	weissflogii	CCMP1010	3	1	
Thalassiothrix	*antarctica*	L6-D1	6	-	Araphid Pennate

Supplementary 3 A list of primers used in this study

#	Name	Target	Sequence (5' – 3')
**Heterologous expression of** PtMCA-IIIc			
1	SDM C202S F	PtMCA-IIIc in pET-21	GATGATGCGCTCTTTAGCCACTTTTCCGGTCAC
2	SDM C202S R	PtMCA-IIIc in pET-21	GTGACCGGAAAAGTGGCTAAAGAGCGCATCATC
3	SDM C264S F	PtMCA-IIIc in pET-21	TGCTTGATGGATTGTAGCCATAGCGGAACGGTC
4	SDM C264S R	PtMCA-IIIc in pET-21	GACCGTTCCGCTATGGCTACAATCCATCAAGCA
5	SDM C259S F	PtMCA-IIIc in pET-21	CACTTGGTTAGCTTGATGGATTG
6	SDM C259S R	PtMCA-IIIc in pET-21	GACACCTTGAACCAACGGC
7	T7 F	pET-21	GCGAAATTAATACGACTCACTATAGGG
8	Pet Rev	pET-21	GCTAGTTATTGCTCAGCGG
**Assembly of GG and CRISPR/Cas9 plasmids**			
9	GG PtFcpP F	PH4 plasmid	TGGTCTCAGGAGACATACCTTCAGCGTCGTC
10	GG PtFcpP R	PH4 plasmid	AGGTCTCACATTCTTGACATCTGGCAACCGTG
11	GG Ble F	PH4 plasmid	TGGTCTCAAATGGCCAAGTTGACCAGTG
12	GG Ble R	PH4 plasmid	AGGTCTCAAAGCTCAGTCCTGCTCCTCGGC
13	GG PtFcpT F	PH4 plasmid	AGGTCTCAGCTTCCTTCCTTAAAAATTTAATTT TCATTAGTTGC
14	GG PtFcpT R	PH4 plasmid	TGGTCTCAAGCGCTCGAGAAAACTCATCCTG
15	SDM F fcpP	Level 0 fcpP	AGCGTCGTCTACACTGTCAC
16	SDM R fcpP	Level 0 fcpP	GAAGGTATGTCTCCTGAGACC
17	SDM F fcpT	Level 0 fcpT	CACTAGCTCGACTTCACCATGG
18	SDM R fcpT	Level 0 fcpT	TTATTCCTGACTGTGAAACCAAAG
19	GG PtU6 Prom F	*Pt* Genome	TGGTCTCAGGAGGTTGGCTCGGAAGTTGGTG
20	GG PtU6 Prom R	*Pt* Genome	AGGTCTCACTCGACTTTGAAGGTGTTTTTTGAC
21	GG sgRNA1 F	Scaffold	AGGTCTCACGAGGGATTGTTGCCATAGCGGAAG TTTTAGAGCTAGAAATAGCAAG
22	GG sgRNA2 F	Scaffold	AGGTCTCACGAGGTAATACATACCAAACCAAGG TTTTAGAGCTAGAAATAGCAAG
23	GG SG R*	Scaffold	TGGTCTCAAGCGTAATGCCAACTTTGTACAAG
**Scan and sequence of transformants**			
24	Cas F*	L2 plasmid	CCGAGACAAGCAGAGTGGAAAG
25	Cas R*	L2 plasmid	AGAGCCGATTGATGTCCAGTTC
26	PtMCA-IIIc ex2 F	*Pt* Genome	GTGAACGATGACTTTGACTTGGTCC
27	scan KO R	*Pt* Genome	CGTCAATTTCCATTTCTGTAAAGTTTCCG
28	PtMCA-IIIc R seq	*Pt* Genome	CCTTAACTTTCATTCTGATGTGCAGCAC
29	PtMCA-IIIc-KpnI 5'	PtMCA-IIIc in pET-21	ATATGGTACCGGATCTCAGTAGTGGTGGT
30	PtMCA-IIIc 3'	PtMCA-IIIc in pET-21	CGGATCCGAATTCATGGGTTTCCTT
31	H4 T → gene	PH4 plasmid in *Pt*	GCAAGGATGCCCATTTGTGC
32	H4 P → gene	PH4 plasmid in *Pt*	ACAGTCAATACCGAAAACAAAC

qPCR

33	qMC5 F	*Pt* Genome	CAAGTTCCTCAGCTAACCGCG
34	qMC5 R	*Pt* Genome	TTGACGTCGTTATGGCAACCTCTC
35	TBP F**	*Pt* Genome	ACCGGAGTCAAGAGCACACAC
36	TBP R**	*Pt* Genome	CGGAATGCGCGTATACCAGT

*These are primers 6, 21, 22 from Table 1, (Hopes et al., 2016). **Primers from Table 1, (Siaut et al., 2007).

Supplementary Methods Identifying MC genes from various species

Identifying MC genes from various species: Initial lists of genes were taken from the pico-Plaza (https://bioinformatics.psb.ugent.be/plaza/versions/pico-plaza/) gene family HOM000388 for the following species: *Aureococcus anophagefferens*, *Arabidopsis thaliana*, *Chlorella sp* NC64A, *Chlamydomonas reinhardtii*, *Coccomyxa sp.* C169, *Ectocarpus siliculosus*, *Fragilariopsis cylindrus*, *Micromonas pusilla* CCMP1545, *Micromonas sp.* RCC299, *Oryza sativa, Physcomitrella patens, Phaeodactylum tricornutum*, *Thalassiosira pseudonana, Volvox carteri*. Sequences were also taken from JGI according to Table S1 from (Choi and Berges, 2013), with the exception of *Guillardia theta*, whose sequences were taken from (Klemenčič and Funk, 2018), and *Emiliania huxleyi,* where the sequences were manually curated. For the diatoms, sequences were taken from the Moore collection MMETSP (Keeling et al., 2014). The MMETSP data is currently available at: http://datacommons.cyverse.org/browse/iplant/home/shared/imicrobe/camera/camera_mmetsp_ncgr/combined_assemblies. All of the diatom datasets were downloaded, both the .pep and the .nt files, for the following species: *Amphiprora sp. Amphora coffeaeformis* CCMP127, *Asterionellopsis glacialis* CCMP134, *Chaetoceros affinis* CCMP159, *Chaetoceros curvisetus*, *Chaetoceros debilis* MM31A_1, *Chaetoceros neogracile* CCMP1317, *Corethron pennatum* L29A3, *Ditylum brightwellii* GSO103, *Ditylum brightwellii* GSO104, *Ditylum brightwellii* GSO105, *Extubocellulus spinifer* CCMP396, *Fragilariopsis kerguelensis* L2_C3, *Fragilariopsis kerguelensis* L26_C5, *Nitzschia punctata* CCMP561, *Proboscia alata* PI_D3, *Pseudonitzschia australis* 10249_10_AB, *Pseudonitzschia fradulenta* WWA7, *Skeletonema dohrnii* SkelB, *Skeletonema marinoi* SkelA, *Skeletonema menzelii* CCMP793, *Thalassionema nitzschioides* L26_B, *Thalassiosira antarctica* CCMP982, *Thalassiosira gravida* GMp14c1, *Thalassiosira miniscula* CCMP1093, *Thalassiosira oceanica* CCMP1005, *Thalassiosira rotula* CCMP3096, *Thalassiosira rotula* GSO102, *Thalassiosira weissflogii* CCMP1010, *Thalassiosira weissflogii*

CCMP1336, *Thalassiothrix antarctica* L6_D1. Sequences from *Skeletonema costatum* (Skcos) and an additional isolate of *Thalassiosira rotula* (Throt) were provided by Harriet Alexander and Sonya T. Dyhrman (Alexander et al., 2015). For species with more than one isolate (*D. brightwellii*, *F. kerguelensis, T. rotula*, *T. weissflogii*) the isolate with the most complete MCs was chosen. To identify MCs, a database was built of all the peptide files from the above species, and local Blat was run using the PtMCA-IIIc p20 domain as the query. An additional database was built of the nucleotide sequences, and a translated Blat was run to identify additional potential transcripts that were not translated. For each isolate, manual curation was performed to eliminate redundancy. Sequences from *Hemiaulus sinensis* were found by Blast search at NCBI in the TSA database, and *Fistulifera solaris* were found by Blast search at NCBI in the protein database. For non-diatom species, the input file for database searches was of the full length MCs of 6 TpMCAs, PtMCA-IIIc and 5 SmMCAs, and the searches run were translated Blasts (using the protein query against the species DNA). Red algae: The following genomes were searched at Ensembl Plants: *Chondrus crispus* (hits), *Cyanidioschyzon merolae* (none), *Galdieria sulphuraria* (none), also checked genome, local. For *Porphyridium purpureum* genome http://cyanophora.rutgers.edu/porphyridium/ website was used. *Porphyra yezoensis* (Nori) search performed at NCBI (genome, TSA). *Nannochloropsis gaditiana* (genome local, TSA at NCBI), *Cyanophora paradoxa* local searches. *Symbodinium* isolates: A1, A2, B2, C, D, *muscatinei*: Translated Blast was run at NCBI against the TSA database. For each isolate manual curation was performed to obtain a non-redundant dataset. A1 was chosen as it had the most and most complete MCs. *Plasmodium falciparum* was taken as a representative of *Plasmodium* species (Le Chat et al., 2007). *Trypanosoma brucei* sequence (TbMCA-I MCA2_TRYB2) and the domains were taken directly from Uniprot. All the full protein sequences are presented in Supplementary Dataset 1.

Supplementary references

Alexander, H., Jenkins, B. D., Rynearson, T. A., and Dyhrman, S. T. (2015). Metatranscriptome analyses indicate resource partitioning between diatoms in the field. *Proc. Natl. Acad. Sci.* 112, 201421993. doi:10.1073/pnas.1421993112.

Choi, C. J., and Berges, J. A. (2013). New types of metacaspases in phytoplankton reveal diverse origins of cell death proteases. *Cell Death Dis.* 4, e490. doi:10.1038/cddis.2013.21.

Hopes, A., Nekrasov, V., Kamoun, S., and Mock, T. (2016). Editing of the urease gene by CRISPR-Cas in the diatom *Thalassiosira pseudonana*. *Plant Methods* 12, 49. doi:10.1186/s13007-016-0148-0.

Keeling, P. J., Burki, F., Wilcox, H. M., Allam, B., Allen, E. E., Amaral-Zettler, L. A., et al. (2014). The Marine Microbial Eukaryote Transcriptome Sequencing Project (MMETSP): Illuminating the functional diversity of eukaryotic life in the Oceans through transcriptome sequencing. *PLoS Biol.* 12, e1001889. doi:10.1371/journal.pbio.1001889.

Klemenčič, M., and Funk, C. (2018). Type III metacaspases: calcium-dependent activity proposes new function for the p10 domain. *New Phytol.* 218, 1179–1191. doi:10.1111/nph.14660.

Le Chat, L., Sinden, R. E., and Dessens, J. T. (2007). The role of metacaspase 1 in *Plasmodium berghei* development and apoptosis. *Mol. Biochem. Parasitol.* 153, 41–47. doi:10.1016/j.molbiopara.2007.01.016.

Matthijs, M., Fabris, M., Obata, T., Foubert, I., Franco-Zorrilla, J. M., Solano, R., et al. (2017). The transcription factor bZIP14 regulates the TCA cycle in the diatom *Phaeodactylum tricornutum*. *EMBO J.* 36, e201696392. doi:10.15252/embj.201696392.

Rosenwasser, S., Graff van Creveld, S., Schatz, D., Malitsky, S., Tzfadia, O., Aharoni, A., et al. (2014). Mapping the diatom redox-sensitive proteome provides insight into response to nitrogen stress in the marine environment. *Proc. Natl. Acad. Sci.* 111, 2740–5. doi:10.1073/pnas.1319773111.

Siaut, M., Heijde, M., Mangogna, M., Montsant, A., Coesel, S., Allen, A., et al. (2007). Molecular toolbox for studying diatom biology in *Phaeodactylum tricornutum*. *Gene* 406, 23–35. doi:10.1016/j.gene.2007.05.022.

Smith, S. R., Gillard, J. T. F., Kustka, A. B., McCrow, J. P., Badger, J. H., Zheng, H., et al. (2016). Transcriptional orchestration of the global cellular response of a model pennate diatom to diel light cycling under iron limitation. *PLOS Genet.* 12, e1006490. doi:10.1371/journal.pgen.1006490.

Wong, A. H.-H., Yan, C., and Shi, Y. (2012). Crystal structure of the yeast metacaspase Yca1. *J. Biol. Chem.* 287, 29251–29259. doi:10.1074/jbc.M112.381806.

Supplementary Data 1 Full protein sequences of all the MCs used in this study.

Codes for all species presented in Table 1:

Pyezoensis	*Pyropia yezoensis*
Ngaditana	*Nannochloropsis gaditana*
Cparadoxa	*Cyanophora paradoxa*
Cc	*Chondrus crispus*
Ppurpureum	*Porphyridium purpureum*
Aa	*Aureococcus anophagefferens*
At	*Arabipdosis thaliana*
Cv	*Chlorella variabilis* NC64A
Cr	*Chlamydomonas reinhardtii*
Cs	*Coccomyxa subellipsodea* C-169
Es	*Ectocarpus siliculosus*
Fc	*Fragilariopsis cylindrus*
Mrc	*Micromonas pusilla* RCC299
Os	*Oryza sativa*
Pp	*Prymnesium parvum*
Pt	*Phaeodactylum tricornutum*
Tp	*Thalassiosira pseudonana*
Tb	*Trypanosoma brucei*
Vc	*Volvox carteri*
Pn	*Pseudo-nitzschia multiseries* CLN-47
Eh, Ehux	*Emiliania huxleyi*
symbA1	*Symbiodinium* A1
Pf	*Plasmodium falciparum*
Gt	*Guillardia theta*

Putative domains, marked on MCs full protein sequences, where available, or longest sequence where full ORF not available.
The putative p20 domain is marked in orange, the putative p10 domain is marked in blue.

```
>Pyezoensis1 contig1 GFKY01015919,GFKY01026864
MGKKAVLIGCNYPGTKAALEGCCNDVDIMYDVLQRYKGFDASEITILKDDGSCSDELIPTGANIRKALTKLCGEAQE
DDIIFVHFSGHGTQVPADDDDPEDDRKDEAICPTDMSLIVDDDLRAIVSTIPDGCRLTLVTDCCHSGSMLDHSEVAI
EGEKDGNSKASLLEESASLMSLLTGGTREVGGVDVKNRALPITDVASLLTQITGRSVQPGNIRSTLGEFFGGDAGRL
ALQYFLKSGKSGSGMSGGSGLAAMAGMLAASQGGGQSSSSGGGSSGLAGLAGSLLGGKKQSQSSGGGGSAALMGLAG
SLLGGKKPQQSSHSSGGGSGLTAAMGMMSMLSGSGGGGSSGGSHSTSHSLGGSLSEDKGILITGCQAHETSADVRPP
GGKAFGALTNSIQTVLKTNPDASYYDVVSGARTVLSQAKHAQNPCLECSEKNSRLPFIC

>Pyezoensis2 GFKY01000140,contig_9228_g2199
MSGAPPPDTWSSSSRGVGGSGGYGGYGAPSPGSGGYGPPPGGAYGQPPAHGPPPSGFDAEYEARIRQQNEEEARGAG
GGGQTPYGSSLGPYPPQQQDPYGQHPQQQQQHTYAGAHGGYRAQPPSYHAQPPHPPSYAHGAPPSNYGAAPPIQHVP
TPHVPFGPAHPATGYVQPPNAPAPQYGPSASGPATGWTPSGRKRALLIGINYKGTPSALAGCVNDVRYIQYLLASKF
GFRPSDFVILTDERVDVPGARTGPPTRASILAEMRALLSGAQPGDSLFLHFSGHGKQIKDVSGDEEDGLDEAILPSD
```

YLRSGHIVDDEMYHILVRPMPRGVRLTAIIDACHSATGLDLPYVHGAPGGSQYGGGAVAAQGRGGLSSLLSGGGGGN
AALASMAMAALGGGKKPMIGGGGGSGGGGGGSLGAILSAVVMGASGKPLSGKQQARMVEKRQPNPNAGEVIAFSACE
DHATAADTHGAAGGYATGACTYSFIQAIEHGGRDWHSYTFESLLVEMRRKMRAKGLKQVPQLTSSFPMSISSRFIV

>Pyezoensis3 GFKY01041200,contig_32374_g7869
MTIPTGHDTWTSSRTVDGGTGAANVLPPRGPPAYPLPRSNHHPFLTPPFAAAASATSSAGPDTWTPRRRPPTGAGNL
LPWGHPAAGESASSPWRTSAPPPAAPRAVHDALIGEEIERGARVDALMRPPLSGGLPVRPVHMPWSTAAAAPAAAAS
SSHFQPRKYALLVGINYWGNHFMPTLRGCVADVRNVRRLLTSRGGYDPADIVTLTDEPLDGIGGAWGKFPTHDAILA
EMRALVAKPKAGDSLFFHFSGHGAQEPDASGDEIDGMDETFVPADGTPTGRTILDDNVHKLLVKALPPGVRLMALID
ACHSATALDLPHLLDEPWGAGHGCNAACGTEKGCGGDRVISLGACRDDSAAADWHRAGRGTSSAGAMTSFFIDAVER
NVGGTTHRAVLNYVRRRILEEGMTQEPQLSSSSPLDPYARLEL

>Ngaditana GAGR01012758
PTNSRPYRRSLRRPPPWPSTQAPTNLYTPVGGFVANNAVATPPPSQQGRPSSSLSSFAPPSGYQPPPRPAPSFPPSS
SAAYRPPHRPPSSSPSPLPEGSTYQRPNDLHGALPFSSPCNGRRKALFVGITYAGTRAELKGCVNDVKNLHAFIQRT
YGFQPQEMRVLTDDPGFSEGTPTRQNIVQGMRWLVGGARHGDSLFFHFSGHGGSVRDQNGDEADGMDETILPLDHAR
AGQIIDDEIHDLLVKPLVQGVRLTAVFDSCHSGSVMDLPFSYKVDGSLEIVQVDNTAKVVNSLANAGLAYLSGNKAV
ALMSGLEAVGTMLFGPKTNHDARRRTEENNQSPADVIQFGGCRDDQTSADTRIDGQATGALSWALIRVLSQGPNQDY
AQVLKNMRQLLRGKYTQIPQISSSHKIDLFSPFKM

>Cparadoxa1 Contig8192
MPPPPPGYRQAPAPAPGVRPTQNLLYDPSKRRRAVHVAISYKHDEDNFLPGCWNDAKSMVDLGMKQYGFKSKDIKML
TDEDFNKNFPSFPTHLIEGARPGDILLFTFSGHGGQIKDLDGDEIDGKDETLIPVYETAGEITDDELAALFAKLPRG
VRLICFLDCCHSGTGLDLPYHHNILPAPKAGQQLRNYLPASRNKALPAPSYAPPPMAKTSMDPETPKKKHHEPFLDA
IFGPIVHPESYQDQYGNHSQQLQQARAYGVGGPMDAGHEMRPIVVQTNLRENTGILNFGKA

>Cparadoxa2 Contig7118
MSWWNPFGGAPEPPVPGVPVGFAVNQGPPPPYGAAPTVHYVPPGNVTKKALLIGINYLRTPECRLSGCINDVNCMKY
LLMTKYKYEERNILCMTDDQSDPLKQPTRHNIVQGMRWLVMGTKANDTLFFQFSGHGSQVRDHSGDEADGMDETILP
CDFKQAGQITDDEIYQLLCRPLPYGSPPTPPHSLPPHAFGSQRWRMDCCHSGTGFDLPYTHSLNFFGQLSTTTEPMQ
PNWCCQGAWIAKRAPTRAIETSFAQKTTLTYGQLVVSMLNEVQADAPTVINLALEFDCHWNSGLVL

>Cparadoxa3 Contig26116
MPTKRALIVGIKYIEWPRGTLNGCVNDAKEIITGTFGYAEENVKMIIEKPGFVQPTRANIMEGLSWLPGDQLFFGYS
GHGSQRADEGKDEVDGKDECLVPMDFQTEGYIRDDELNKLLVVQPPPGRCQLLTLGQIFDCCHSGSGIDLDYTIMTA
AGFSLATATAAKDPMGKGVDLDEDGDGVRAAAGADAEPSIDTVVVAQVANMQAKAVGSK

>CcMCP1 XP_005717184.1 metacaspase type II, MCP1 [Chondrus crispus]
MGKYAVVIGVNYTKNPEAALQGCCNDARLMVNLLHSKGFEDEDIKLLVDDDDSNDSPNHVNVKKALDWLCTGRSEGD
TIFMHFSGHGTQVPADDDDVEEDKLDEAICLEELFLMADDDLKQYFSQLPEGVRATVVMDCCHSGSMLDGQEVAIQG
AKDEDSAVPPQESDDLVNVLGGSREAVSNRSLPISTICNVMSQKLGSPVSPTGSGVNGAMAQVFGGTAGKLMMKFAL
GQMAKQDGGSSNPLVGMLGGMMGGKSTSSSGSNPLAAMMGGGSTSSGSNPLAAMMGGGGSTTSSNPLASMMGGGEST
TSSNPLASMMGGGESTTSSNPLASMMGGGGSTTSSNPLASMMGGGGSTTSSNPLASMMGGGGSTEQNGNPMGSLMGA
LGGGNASSGGSNPLASMMGGGATETETAAPQSQNPMAMLSGLMGGMGLGGQEDSGDAPAYNPAHQPMSRDVCTLITG
CQASETSADVRPAGEEAFGALTKTLTTLYEKNPETTYHDLVSNVRSELSRGGFKQNPCLECSETMAHQPFIC

>CcMCP2 XP_005713893.1 MCP2 [Chondrus crispus]

MRDRGLPLPQSLSAYQRQAEEASQVETVPDPPRLDGLNRKPSISPAAADYVPELPVGYSARMNRRKALLIGIGYRKH
KYLNVLPGCKNDVTAMFNLLTSELFGFPQDEVRVLSDELNMLGSVHVQAPTRFNILRDMLWLTEDVGEGDSAVFFFA
GHGDFIEDVSGDEIETGVDQCIMPIDCLPEPKHRTGQTFKGVPPILDDTIYERLVRGVPAGAKVTAIVDACRSGSVC
DLPVMHGDDGVKYRASGGDPPRQRSSPHKGAGGFVLFSGSADDQQSIDMTIATGRGNGEVESFGVMTRSFVDAVQEM
AMHRCPETYDGVESWTYGQLFQRVRELVWERTSSILPHYIEKQEPQMSTSHTFDTWQAPFSI

>CcMCP3 HG001672 [Chondrus crispus]
MQRHMDPNPSSPQSGLSSSRRKAPPRVSLDLCETPPGNYGARMSVENGEIRSPLAAGAFQVKPKIRPARTRYSLSDL
TYAERNMQDVPMSPLVTRGGPVKSLADSGETDVRSPYSRSLSRNRRLSNVPYNASPKNSSRKAVLIHACYINTEGAQ
RLRSQSNLASMYDLLVLSLGFSRGNVWVLTDEPKTIPGAVNFTPTRGNILNSMRWLVKGSSAKHQLLFCFSGHGCRV
LKERNPVAVFEDCILPCDYPMSSPISETEIKQILVQNLRNGATLTSLLDCQNSAQLMNLPYIHAAARGAKGSFFLRE
EPEAQRFTSASTPGVVLNSVLRFSKLKRGGAEQRRMAAEERRNANAATCFDNGTVICISWSFETERECPSISSSPPN
HGCLTHAFVRYLKHSAAEKSKPSYSTALCAMSAWLSSRGGNLPQFSSTHKVSPDKPISLL

>CcMCP4 XP_005710904.1 unnamed protein product [Chondrus crispus]
MSNPGVFPDGTLDPEYSRRINANNASKPRGHLYPSSAYPPQPQPYPPGYRPPHGYSPYAQPQNPYPPQPFHPPPPPP
PHPPQHPYAPQQPYPAHPPQPSYNPHGPPALAQSASHMTSPFRPSGRRKALLVGINYRGTSSALRGCVRDVTFVHHL
LVSKFGFRKKDFVVLTDESVNIPGVRKGPPTRRVILDSLKWLVSGSRSGDSLWFSFSGHGAQVRDVSGDESDGFDET
IVPVDHKRAGHIIDDELYEIVRHVARGARLTVLLDACHSGTGLDLPYQHDVFGTGSGQRISGGKGMSLTSGLLNVAG
ALLNGSASGALNAGFNMATGGKKKKKTPGPDPNAGEVLLFSGCKDNQTSADTSKLTGGLPTGAMTFALIETLEHSTV
GDWRNYNYRQLLQTMRQKLRAAKMTQVPQFSTSHPFDLSTSFML

>Ppurpureum1 evm.model.contig_3552.1
MWSSSYAPGSAPQQPGTFYGAQPPPPPQQXXXXXXXXXXXXXXXXXXXXXXXXXXXXXXXXXXXXXXXXXXXXXXXX
XXXXXXXXXXXXXXXXXXXXXXXXXXXXXXXXXXXXPYQQYPPEQPILASMVSSNALGTKKLSINYYQDSRLPASAX
XXXXXXXXXXXXXXXXXXXXXXXXXPVQHPGYGPAAPKAPATGRRKAVLIGINYTGTRNQLKGCVNDVRFIKHLLQT
RFGFKDEDFFILTDEDPKISGVRRAAPTRYNIIDALRWLIGGVTAGDSLFFHFSGHGSQVRDTNGDEEDGYDETILP
VDFQRAGQIVDDELHAILVKPLPAGARLTALIDACHSGTGLDLPYVMDVDQYDPSAVAASGYGRGAQQRGGGNERGL
IGAMIGAGLMASASYGAPPPNKRKKYQKKVPLAPACAGDAVLFSGCDDHQTSADTSGLSGGTSTGAMTYCFVEAIEH
GSVSDWHQYTYGSLLSTMRSKLLKKGYKQRPQLSSGKPMSASTPFRI

>Ppurpureum2 evm.model.contig_2187.3
MVAKRAVLVACNYPGTNAELKGCISDAKIHKSILIEKKGFKESDIKVLIDDEDSELTSYAPPTGENIKKALTELCES
AQPGDFLFFSFSGHGTQVPGGAEEEDGKNEALCPTDMNLLVDDDIRDIVNKTKKGVTLTLVCDCCHSGGMIDHSSIL
IDSDKKADPDAPPVEFRSRELPISGVAERLSQLLGKTVDPNGTAVRGAIHEKYGAAASKFAVGMFENYTGKKMGTEE
KKKLTGMITSCFSALGVGGGGGGGSDHSNAAPAVSGGSAGGPPKKVGHMVHVDEEMGILITGCRAHETSADVRENGQ
AFGALSKNLHLYLETKPDATFYEVVHGARDRLADKGFTQNPCLECAKDNAALPYICPNV

>AaMC1 AA0026G00590
ALLVGCNYPGSSAELRGCVNDVLRMRALLLGQGFPEQQIVILRDDRGGQQRPTRRAITEGLRWLAAGAGRGDSLFFH
FSGHGSQERDRTGDEADGYDETIVPCDYKSAGQITDDELHAILVRPLPDGARLTSIMDCCHSGTGLDLPYCFTPGRG
WQTDDVPCFSRGDVQLFSGCEDDQCSADTYANAAAGGAMTNAFLKALAENPMPLYPDFLTALHRELRRKGFKQKPQL
SSSQRFDLNQRVFSLTEGFIPNSNQTIGRRPGMRRKKSRRRGRGAGVGMETMLAAGIGAAVLSSLF

>AtMC1 AT1G02170
MYPPPPSSIYAPPMLVNCSGCRTPLQLPSGARSIRCALCQAVTHIADPRTAPPPQPSSAPSPPPQIHAPPGQLPHPH
GRKRAVICGISYRFSRHELKGCINDAKCMRHLLINKFKFSPDSILMLTEEETDPYRIPTKQNMRMALYWLVQGCTAG

DSLVFHYSGHGSRQRNYNGDEVDGYDETLCPLDFETQGMIVDDEINATIVRPLPHGVKLHSIIDACHSGTVLDLPFL
CRMNRAGQYVWEDHRPRSGLWKGTAGGEAISISGCDDDQTSADTSALSKITSTGAMTFCFIQAIERSAQGTTYGSLL
NSMRTTIRNTGNDGGGSGGVVTTVLSMLLTGGSAIGGLRQEPQLTACQTFDVYAKPFTL

>AtMC2 AT4G25110
MLLLVDCSSCRTPLHLPPGATRIRCAICHAFTLIAPEPRLQSHASASPFPFPNSSPAPSTFIYPPPTPSPYTHAPHA
PSPFNHAPPDSYPFTHAPPASSPFNHAPPGPPPPVHGQKRAVIVGVSYKNTKDELKGCINDANCMKFMLMKRFQFPE
SCILMLTEEEADPMRWPTKNNITMAMHWLVLSCKPGDSLVFHFSGHGNNQMDDNGDEVDGFDETLLPVDHRTSGVIV
DDEINATIVRPLPYGVKLHAIVDACHSGTVMDLPYLCRMDRLGNYEWEDHRPKTGMWKGTSGGEVFSFTGCDDDQTS
ADTPQLSGSAWTGAMTYAFIQAIERGHGMTYGSLLNAMRSTVHEIFDKNKGRELVEVGGADFLSTLLGLLILGASPP
DEEEEVNQAPQKTQEPQLSANEAFAVYEKPFSL

>AtMC3 AT5G64240
MASRREVRCRCGRRMWVQPDARTVQCSTCHTVTQLYSLVDIARGANRIIHGFQQLLRQHQPQHHEQQQQMMAQPPP
RLLEPLPSPFGKKRAVLCGVNYKGKSYSLKGCISDAKSMRSLLVQQMGFPIDSILMLTEDEASPQRIPTKRNIRKAM
RWLVEGNRARDSLVFHFSGHGSQQNDYNGDEIDGQDEALCPLDHETEGKIIDDEINRILVRPLVHGAKLHAVIDACN
SGTVLDLPFICRMERNGSYEWEDHRSVRAYKGTDGGAAFCFSACDDDESSGYTPVFTGKNTGAMTYSFIKAVKTAGP
APTYGHLLNLMCSAIREAQSRLAFNGDYTSSDASAEPLLTSSEEFDVYATKFVL

>AtMC4 AT1G79340
MTKKAVLIGINYPGTKAELRGCVNDVRRMYKCLVERYGFSEENITVLIDTDESSTQPTGKNIRRALADLVESADSGD
VLVVHYSGHGTRLPAETGEDDDTGFDECIVPCDMNLITDDDFRDLVDKVPPGCRMTIISDSCHSGGLIDEAKEQIGE
STKKEAEDEDESEESSSRFGFRKFLRSKVEGAIESRGFHIGGNKKDEDEAEEIETKEIELEDGETIHAKDKSLPLQT
LIDILKQQTGNDNIEVGKIRPSLFDAFGDDSSPKVKKFMKVILGKLQAGNGEEGGLMGMLGKLASGFLEGKLNDEDY
VKPAMQTHVGSKEEVYAGGSRGSVPLPDSGILISGCQTDQTSADATPAGKPTEAYGAMSNSIQTILEETDGEISNRE
MVTRARKALKKQGFTQQPGLYCHDGYANAPFIC

>AtMC5 T1G79330
MAKKAVLIGINYPGTKAELRGCVNDVRRVHKSLVDRFGFSERNITELIDTDESSTKPTGKNIRRALLNLVESAKPGD
VLVVHYSGHGTRLPAETGEDDDTGYDECIVPCDMNLITDDEFRDLVEKVPKEAHITIISDSCHSGGLIDEAKEQIGE
STKKKPKKESGGSSGLGIKGFVREAVEEALESKGIAIPHHKDEKDENKTKELKLEDGAKVHVVNKSLPLQTLIDILK
QNTGNNDIEVGKIRPTLFNVFGEDASPKVKKFMKVILTKLQEGKTEGGILGMIGKLAQEFLKHKLNDDEEYVKPAMK
THVGNKQEVYAGASNGSLADNGILISGCQTDQTSADASPQGHPEMAYGAFTNAVQIILEETKGMITYKELVLKARKL
LKKQGFSQRPGLYCSDSFVNAPFIC

>AtMC6 AT1G79320
MAKKALLIGINYVGTKAELRGCVNDVRRMRISLVERYGFSEENIKMLIDTDSSSIKPTGKNIRQALLDLVEPAKSGD
VLFVHYSGHGTRLPAETGEDDDTGYDECIVPSDMNLITDDDFRDLVDMVPKDCPITIISDSCHSGGLIDEAKEQIGE
STKKKKDSGDSSTINKETEAEIIEVGNRSLPLETLIDMLKQETGNDDIEVGKIRTTLFDMFGDDSSPKVKKFMNVIL
SNLQETTTTIQTVSDEVLGSVENLAQEFLEQKLSDDVKPAIQDVYAGAINGALPDNGILISGCQTDQTSSDASPPGH
PELAYGALTNAIQIIIGETKGKISNKDLVLKARKLLRKQGFDQRPGLYCNDAYVNARFIC

>AtMC7 AT1G79310
MAKRALLIGINYPGTTEELQGCVNDVHRMHKCLVDRFGFAEEDITVLIDTDESYTQPTGKNIRQALSELIKPAKSGD
VLFVHYSGHGTRVPPETGEEDDTGFDECIVPSDLNPIPDDDFRDLVEQVPEGCQITIVSDSCHSGGLIDEAKEQIGE
STTTKPNRESKVSSFEFEFKNCLHSIFVKLLAFCGIGSSHVETREIVEVGEGDEVVRSRYLPLERFIELLKQQTGQD
NIEIGKIRPTLFDVFGEDSSPKIKKFMKVILTKLRKTNDQSTLLGKIEESARGYIEETLNDEHYMKPAMQAQVKSDR

EIYGGRSSNGLFPDRGILLSGCQTDETSADVKKKGEAFGAFSNAIQMVLSETDHKDKITNKEMVLRAREILKKQMFI
QRPGLYCNDRFVNAPFIC

>AtMC8 AT1G16420
MAKKALLIGINYPGTAVELRGCVNDVHRMQKCLIELYGFANKDIVIMIDTDKSCIQPTGKNICDELDNLIASGQSGD
FLVFHYSGHGTRIPPGIEDSEDPTGFDECITPCDMNLIKDQQFREMVSRVKEGCQLTIISDSCHSGGLIQEVKEQIG
ESHMKPVDKVKEQIEESHMKQPKLGIASYFLNIVMNLLATCGVSKSQRDRGGGEESFRGEIELEKDETLDIKTRYLP
FESYLSLLKEQTGQTNIEPVRIRQTLLKLFGEDPSPNRQRGLSDLGNCEVDAGDSGASRLNAVTDNGILLSGCQTDQ
RSEDVYVTRTGKAYGAFSDAIQMILSAPRKDKKKITNKELVSEARVFLKKRGYSQRPGLYCHDRFVDKPFICY

>AtMC9 AT5G04200
MDQQGMVKKRLAVLVGCNYPNTRNELHGCINDVLAMKETILSRFGFKQDDIEVLTDEPESKVKPTGANIKAALRRMV
DKAQAGSGDILFFHYSGHGTRIPSVKSAHPFKQDEAIVPCDFNLITDVDFRELVNQLPKGTSFTMISDSCHSGGLID
KEKEQIGPSSVSSNISPAIETTNKTITSRALPFKAVLDHLSSLTGITTSDIGTHLLELFGRDAGLKFRLPAMDLMDL
LETMTAREKHVDSGILMSGCQADETSADVGVGNGKAYGAFSNAIQRVLNENEGAMKNKQLVMMARDVLERLGFHQHP
CLYCSDQNADATFLSQP

>CvMC1 CNC64A_020G01030
GRKKAFICGINYFGTSAKLNGCINDAKCMEYLLKSKFGFKQENILMMTDDCPDPMRRPTRANMFQGFRWLTMDMRPG
DSLVFHYSGHGSQTRDYSGEETDGMNETLCPMDFRQAGEIVDDELNRCLINPLPTGVKLHCIIDACHSGSVMDLPFQ
AHVRGGYAQWEASYHFTRAHKGTAGGFAVQFGASKDSQTAADT

>CrMC1 CR12G07090
MYGYPPPAYGAPPAGYGAPAAPSPYGAAPPYGAPPAPAYGAYGAPQPAYGAPPAPAYGGYGAPPAPAYGAQPAYGTP
YGGQAQPSAYTYGQPSANPQQRPATAPAPAYMPPTTYAPAPTNGGRRRRALLVGCGYPGTREALNGCLNDVNCIKFC
LMNRFGFTEQQILILRDDTRQPDFISTKANIFRGIQWLMTDQQPGDSLFFHFSGHGSQQYDRNGDEEDGYDETICPT
DFRRAGQIVDDELNRMMVQPLMPNVTLHAVIDACHSGTALDLPYRAKVDHSGRWYWKGRARYDKCTRGGTAFQFGAC
KDSQVAADTNKLSGKAYTGAATFSFIEAIEKYGVQQTYGVLLGHMMQTLRAMNGGMVSSGATGILASLLLGSSISSG
QEPVLSCDKQVDLYASRLNI

>CrMC2 CR03G07540
MPGKKAVLIGCNYPGTNAALRGCINDVWGMKEILITYYGFTDADLTILIDTDKSYLQPTGKNIKAKITEMVSAAQDG
DVLFLHFSGHGTQIPSADGDEKDGKDEAICPTDMNLICDDDLRVLLKPLETKPGVKFTFIADCCHSGTLLDHESVQI
SGPKSGAPPPPAIDMGALAGFLGALGQPDGRDLKNRALPFSELCGMLSQLLGGVPVDARTVRSSMGTIFGADSSAKI
QQFIGMYQALTAGTKGAAAGGAGGILQMLCACLAPPADQNGPSGTPANAQSGPGAHYAAPAAAGNLAEPDLKVNYTP
GAKPPANEQLGADVGILITGCQSNETSADACPSGNPDKAHGALSNAIQTVIKQQQQSPGQPITYRNLVIAVREMLT
KTGFAQNPCLECSNKNADTPFIVC

>CsMC1 CV04G04400
APAHQQPAPGQTYNPTGRKKALLCACNYRGSSSELRGCINDAHCLRHLLTSRFNFRDSDIVMLTDDSPNPQAWPTRA
NMLYQMQLLTWNAQPGDSLVFHFSGHGTQIRDQYGDESDGLNETICPCDFKTAGYIVDDEMNRLLVNPLPHGVRLHA
IIDACHSGSALDLEFKCKVKDTGVRWKNEYTRRTSIYKGTAGGEALQIGAARDKQTAADTATMSGSVSTGAATFAFI
QAIERQGTHITYLQLLYSMNQALEQLHASTGNRPPKLPPKVGGLFGGLVSKVVNGALDMAGLSGQTPVICSNVPFDL

>CsMC2 CV10G02150
PPPLQDGYGAPPPQQPGYGEPPAQQGRKRALICACNYAGTDNALNGCINDAKCMQYLLKTRFGFKEEDITMLTDDQN
DPAKWPTGNNMRAHMRRLVGDAQTGDSLIFHFSGHGSQTADWSGDEDDGYNETLCPCDFKQGGQIVDDELNQLLVNP

LRPGVRLHAIIDACHSGSVLDMEYRAEFHNGMPVWTNEFSKRPSIYKGTAGGEAFQFGAARDSQTAADTSQLSGNVS
TGAATFAFIQVIIWDIVVCLDSRVLSMEGIRSEHHLNCF

>CsMC3 CV05G02260
MGHCASKSQFDREVEEVRRERPDAAATVYSSQRGTPMKDLGLVNGKYSKHSIPAEPETPQRDTIPARPGTPSVSEIF
QEPLRPSPLPTSKALPGVMLSGRLQLPYAAPYVPPPSLPPPEYAHLAASGAWQPGRRRALLVAANYSRSADGSARLR
GCINDVHCLKHLLTSKFGFQDNNIVLLHDEQPHGDYWPTKDNILAAVRWLLEDCQPLDSLVFAFSGHGSLDTLCDEH
GRDGILPSDFLEAGPIYEDELYEGLVARLVKGSRLHCFVDTCRGIFALGLPSCEDERGLDSGFGASLRPQPDGEVVM
LSSTLSEDEDLDMDSTDYSHYASTGAVTFSLIQAVEQGQAATYNVLLRAMRYSLKNGPQHFPKVPELSASQDFDLNR
PFLL

>EsMC1 ES0009G00290
MEDDGTTINGTMIDGPEQDNSGDPPGPDTRAEDMSDDDFDTEIADGIKADVRMFSGCQDQQTSADVHDVTKFGVPKS
DGGAGGACTNALLVNVKDDEPDSWMSLLKGMRTTLSTKKFKQIPQMATSKKLDIHSPFSLAGGEGGKHRALLIGINY
VGDKSAELKGCHNDVAQMKDYIVEHGYSGEGEDLKIVMDDGEHTAPTRANIIEAIEWFVEGAAPGDSLFMHYSGHGG
SVEDNDNNEKDKRDETMIPVDYRVSGHIKDDELLAELVLPLPEGVVLSVVMDCCHSGSILDLPYTFDADEGALQLVD
DGGSAVVHKKAKFNMSKMKKAQKAKLGSPYMKAIRAKAAAAAAAKAAAAEAAAAETAATEAASEDQDQDQDQDQDQD
KDQNQDQEESPQPSGSGTAFKVDSKVAVEAAKAKELAAAKAASQNQDQDQGQEQERKQPANGSTGGATKDQPPSEDV
APSATVAPKFQLSIETEVVKAMESGGYVTPDPVAFKSFTSCEAAMEEDGKDDCDLSVFVVTPSSVDEESGHEHAAGI
SMLWVDSESSTGYSIKHLTKGDARFDGKRAVYIEATKGEGEEPFLVAVFAPEDFKPASVGGNTRVAPESAEFVVWVC
DKLAHSRDETLNWTACGSFVGTKVKRTSATTSATGERLVIVGALDEHNEGRVFYGTSLGNEDLQMSTFPKPRAMAAS
NQQVSTVQASALEKDGETFAIAVALPAAIPKETAAAGDSSDTSDDETEIRPSMLYAQSLNDPGFYAGRIEIPGDTRC
FMTTPAYGLKTDVYVAGSKKAMWYDDRSWDGSTESAGVCVNAVDFTSPAKEMRTMLDKQYIRHVFILLENGKLLYTS
QDPEEGSKFDEPIDILPNVEAFSCHIDSIGNVHVNNINKNGNLMHHMMDQGTGLWMQKEVMVSGRDAREHLLSVVDL
KVTSLKKTAGKKTAKDLMDDAEADLPTISLSCGSGAMFRINGKGHLMHKDKIVNVQPNMNGSVRIGQFVDSFSVPHV
AIRMEGLDRPLEIMCGNILTKKLEKMDASALKDAKSRDAMGDDAESLIDDPDDGDLNDFVDAVAQLAKGFEVLSPTE
FPKKNDVDGIFRLTGGAFNDKNKFVAFVDEGPAFEEEEVDDGPPSPGYESTTRQIGGGVESIGGELKEMASPQRPTS
VFRGDSALRGRGPVVRRFTKHRCAPDPFGMGHIGVERVRRVECRNMGNLAKKARENADEAIYETKKHAAESLKSKLS
NAERAQEQATEYAAEATKWAGEAKQAAEAVRWTQAAVEAAEAAVKASEEVEMKASQDIGEMKAAQAFGARKASQDSQ
VDIILGVEGPEEEAARAMEAAARAVEAAGVAQQKSEQAKAIERLQGSDGQLVDAGDFEAGGFLSAEYSRGRFFGPVA
KKLWLAAEAKRAGARPGRDSSYLPVRARHGQSPAWPGRSPGYPGRSPAIGVRYRRTAASTPAAHSDVVEAVEAAGQA
VEAAAQAMERAEQAKEALGEDTGEPERKGDDPDDNNPREYGSAEPSNKGFFDSIANELRELADEAKREAEKEAAQAG
HGPPGHTSSGLFGSEAMEGETTTRRPIINSTAECLTEPRWEEARKKAERTALEAARLVKAAQTAAFELKAVEEAEAA
EALDSEEDEGEDDIDGDDGAYGSTEHTSRGIGGWVKKTTKKAKSGVQSAANKTKKAAQTAARKAEEAAKQAKREAER
IAKDAARKAEAAALQAKREAEKAAKAVAQAAEQTKKAGGLVANEVADKAKIAADVAKQAAEGAGKLAEDAGNEVGDV
AGDIKDGVQDAAGTVKDGIVNAADKVKDVAKDTVEVVGDVTMKVVEVVEEVVVEVWEHTPEGLKKVARKVSKVVKKG
VDFVVKIGKVVYHFVETKLEQLIKALDWLWDKIKTGIMKAIEWLANFFKYKEILKTAEILEAQVLAGMDEFIKSIPT
QGDIKEYFNEIRQMLQKKTGLDLDAQDMSKEDTAQDDREIDPKENFVQHHAESGTFHESAGPADEEEDSSDDSDDDD
SGKDSMADKLQYLYGKFKEGLNAFMSALAEGFLDVLEASTKAMMDPLKNAANAVRAWLADGGTQYIPVISEIYKLIT
GRKLKLAKALFFMLAIPVTYASKALLGKWPSELGLIDPAMDTNSSTMAIVEEDVQSSGTGAPAHLTRSLPGDNSSSE
FVYTSRSGEKESDKGSETKKKVWWALLKVASIVMSGIVIPFQGDGKFAQLIGFDAVKRQRCSSSSILRPIATAYKTT
WLERTSCSFSRVLYGQRPVVPRRCKHRKYSPFFFWVRVEMPLSNRMHVCANHLTAVLGCVHCLLFRIAAVVDVSKVP
GQRRFPRGHRVFKIISLAKVIQKLLDRGASMVYVGDYFDKTKNRENKKKTVLKKGDKGLFWFTFALEVGGTVADAAL
GFSEQGHPHAGGLEHNTPQGGLTPVIVYDACEGIAEIAKGARLMWLAIEFGEGGFQQGTMVFQGVHGVFKGASQLAK
AYSKFLKVPPNKPEYKIMVILFAAHMKYRTNVWPLGIASVAVQLIGADKADTEDQEQTNDAYWTACHAYL

>EsMC2 ES0024G00620

MNSMGVKQGGEERAPAAAAAAAPAAGGGHGQHAAGGGGGGGGKGGKKRGHRAKDIPDEEFDAEIAGGIRADVRMFSG
CEDVQTSADVHDVAKFGLPDATGAGGACTNAVLANVKDSKPDSWMSLLKGMRSTLKEKKFKQIPQLATSKKMDIHSP
FDLSGGDESGNHKALLIGINYTGGKGELKGCHNDVKQMREYITTHGYPADGANLKIVSDDGEHEEPSKENILKAIKW
LVHGAKAGDSLFMHYSGHGGSVKDNTGDEEDNKDETMIPVDYMKSGQIKDDEILKELVMPLPEGVVLSVVMDCCHSG
SILDLPYSFDAKDGALELVEAGGSGVMQKKKNFNVKKKMHKAQGKH

>EsMC3 ES0394G00050
MEDDGNTTNGVMIDAPEQDSNDHGSQDPPSPDTRAEDMSDDDFDTEIADEIKADVRMFSGCQDKQTSADVHDVTKFG
VPNSDGGAGGACTNALLVNVKDDEPDSWMSLLKGMRTTLSTKKFKQIPQMATSKKLDIHSPFSLAGGEGGKHRALLI
GINYVGDPSVELKGCHNDVAQMKDYIVEHGYPAEEGEDLKIVMDDGEHTAPTRANIIEAIEWLVEGAAPGDSLFMHY
SGHGGSVEDTDNNEKDKRDETMIPTDYSMSGHIKDDELLSELVLPLPEGVVLSVVVDCCHSGSILDLPYTFDADEGA
LQLVDDGGSGVVLKKAKFNMTKIRKARKLNLRGPYMKAIRAKAAAAAAAKAAAAKAASEKQQQEQEQTPQPVGGSST
AFKVGSKVAAAAAKARASAAAKAASQQEQEQEKPAANGGTADTAKDQPPSEDVAPSSTVMQAPTFKLSIETEVIKSM
ETGGYITPDPTAFKSFTSCEADMEEDGQDDCDLSVFVVTPSVDEESGHEHAAGISMLWVDSESSTGYSMKHLTKGDA
RFDGKRAVYIEATKGEGEEPFLVAVFAPEDFKPASAGGGSGVAPESTEFVVWVCDKLAHSRNETLNWTACGSIVGTK
VKRTSATTSTSGERLVIVEALDVKNEARVFYGTSLGNDDLQMSMFPTPRAMRASNQQVSTVQASTLEKDGETFAIAM
ALPATIPKKTPVAGDSSDTSDDETEIRPSMLYAQSLNDPGFYAGSIEIPGDTRCFMTTPAYGLKTDVYVAGTKKVIW
YDDRAWDGSTEKAGVSLNSVDFTSPAKEMRTMIDKQFIRHVFILLENGKLLYTSQDPEEGSIFDEPVDILPNVEAFS
CHADSIGNVHVNNINKDGNLMHHMMDQGTSLWMQKEVMVAGGAAREHLISIVDLKVTALEKTSGKKTANDLMQAAEA
EADLPTISLSCGSGAMFRINGKGHLMHKDKKVEVRPNMSGLVRIGQFVDSFSVPHVTIRMPGLDRPLEIMCGNILTK
KLEKMDASALKGAKSRDAMGDDAESLIDDPDDGDLNDFVDAVAQLAKGFKVLSPTEFPKKNDVDGIFRLTGRAFNEE
NQFVAFVDEGPAFEEEEVGDGPPSPGGAPSPRQGGAGAEPIGGELKEMATPQRRTSVFRGDSALHGRRPVVRRFTKH
RCAPDPFGMGHLGVERVRRVECRNMGNLAKKARENADEAIYETKKHAAESLKAKLSNAERAQEQATEYAAEATKWAG
EAKQAAEAVRWTQAAVEAAEAAVKASEEVEMKASQDIGEMKAAQAFGARKASQESQVDIILGVEGPDEEAARAMEAA
ARAVEAAGVAQQTSEQAKAMERGQGSDGQLVDVGDFEAGGFLSAEYSSGRFFGPVAKKLWLAAEAKRAGARPGRDSS
YLPVRAGHGQSPAWPGRSPGYPGRSPAIAARYRRPAASTPAHSDVVEAVEAAGQAVEAAAQAMERAEQAKEALGEDT
GEPERRDDAGDLNPREYGSAESSNKGFFDSIAKELRELADEAKREAEKEAAQAAHGPPGYTSSGLFDTMDAGIPTRR
PITDTTAECLKEPKWEAVRQEAEKTAMEATRLVKAAQTAAFELKAVEEVEVAEALDSEEDEEEDHIDGDYGAYGSTE
YTSRKFGDRIKKATKKVKSGVQNAAKKTKQAAQTAARKAEEAAKQAKREAERIAKDAARKAEAAALKAKREAEKSAK
AVAQAAKEVKKAGELVAKEVADKAQIAAAAAKQAAEGAAKLAENAVNEVGDVAGDIKDGVEDAADKVKDGVVHAADK
VKDVAKDAAEVVGDVTMKMVDVVEEVVEEVWEHTPEGLKKVARKVAKVVKKGVDFVVKIGKVVYHFVETKLEQLVKA
LDWLWDKIKTGIMKAIEWLASFFKYKEILKTAEILEAGAIYGIDQFIATIPSEDAVKEFFNDLRLMLERKTGLDLDA
QDMSTEDTTPDDREVDPKENFVQHHSTCGTFEGAGTPADAKDDSKDQEKASEDSDDDDPGMDTVAEKLNYLYEKLKE
GLNAFMSALAEGIVDLLEGGTQLMVKILKFVAKKFRDWLADGGPIYIPIISEIYKLITGRKLKLAKALFFIFAIPVT
YMAKAVLGKWPSQMVSVDQLTGAKGNTLSIAGQAPDSTDAAMIDITKIPAGDRNFLTNRFKKSHPIFKIISYGRALQ
KILNLASTMVYVGDYFTGPKTPLKRKDKAFFSFLTMFDAAGVLVSLKVDGGQASEPGAGGLVNMNATEGGQTPNNAT
VPAIIFDMADGIAEIAKGSRLIALANELDEGGRQERTMKFQGSHGIFKGASQMATAFGKQIVKSPADPATKVAVIIV
AAGLKWITKVPLGMASVVLQLEGSAEGKDEDKDLKNNAYWIACFAYL

>EsMC4 ES0414G00090
MGGFMDSIKASCTKMAKQQINEAMAGDKKESASGGGSAATGTRAPEDAEAYAKKVAEAIKADVRMFSGCMDSQTSAD
VKDVSKFGLPDADGAGGACTNAMLLTLSDEHSDSWLGLLKGMQSVLKTKRFSQVPQLSTSREIDVNDKFSLRNTAGG
GSTKCLLIGINYIGQQGELAGCHNDVDMMKKYITTHGYSMDPADCKVLMDDNVHGMPDHKGVIEGFRWLTADAKAGD
SLFMHYSGHGGSVKDTSGDEADNMDETLVPVDYKSSGQITDDEILKELVMVLPEGVTLTVVMDCCHSGSILDLPYAL
KADEGTISAVEAGEVSSTISANPGFDFAKRAHGGGGGVDIEELPRVVYYPIFVTS

>FcMC1 FC007G01280

MDAKIQDAIPAKFVMLSGCEDKQTSADVGNVGQFSLPDPGGKAGGACTSSLLKVLNENQGREMSWIDCLHRMRSVLK
SKGFDQIPQLSSSRMLDVNHQFEIVPQESIQARGARRAVLIGINYVGQQGELSGCHNDVKNIKKYLIGKEGFLEKDM
LILMDDGRGHDPTRQNIMQAFDRITQYSKAGDVVFIHYSGHGGRVVDTSGDEDDGYDETLIPLDFKKSGQIVDDEIY
ERLVKKMPANVTVVCLMDSCHSGTALDLPYEINATESKMHANKGFNMGMLQNPAMIAGCCLCLFPLLMSLMDD

>FcMC2 FC007G03890
MNDQSNPSIITWADLLRKMKNEINDIEYVQAPIISSTRKFDLNTPFSLVPESFDKSTGKKRSLLIGCNYHGTEGAEL
KASHDDIRSMKDYIVNVHGFPETDDMMTILLDDKEHKSPTFTNIVEAFKSLSEQSQPGDSVFIQFAGHGGRILDSPI
NNNVESYDEIIAPSDYNKSGIIRDTLIYKTLLAPMRYGVHVTVIIDCCDTGMMLDLPYSWS

>FcMC3 FC008G00950
EAQRAFENDKPDSMRKDVRMISGCDDTQTSADVSNVNSFQLPDPAGRAGGACTSTLLNILYKDGQTPEDTMSFTEVL
DKMRINLKENGYSQIPQLTSLNPIDITTDFTLVPSDATGGVRRAVMIGINYVGHEQGELSGCHNDVLNMKKYIMNEH
GFDEDNIVVLMDDGQHTSPTKSNMINAYKQIVADSEDGDTIFLHYSGHGTKVRDESGDEEDGYDEALCPLDYNESGL
LVDDDLFAILIEPLQQGVHMVSLMDCCHSGTILDLPYIFKPIPNEDGSMPTSMKLDDTINLDGLIEQFG

>FcMC4 FC059G00280
MLGQLGKKYAGDVANKVEELTLKNLNQRYPQQDTDNSNSSTSSSNNKVLSNSGNKTTEKLHKISGRRRAVFIGINYI
GQKNELKGCINDVINIKNFFRSHYHIDDIMILTDDKRAEPETNYVPTRKNILSAFRWLIKGAKSGDSLLLHYSGHGS
KVKNLDGTEASGYDQTLVPVDHQKSGQIIDDDVHDVLCRKLKKGVRLTAIFDCCHSESIMDLPFTYNINGNLEIIEN
DKNQSIATLVAAGTRFLLDGNKKETKQIFKTEITNLVQSALGNETKQDSAKAKVMETNQTQADVIMFSGCKDDQTSA
DTKINGMSSGAMSYALIHTLKKHRKGKKQQILTYTELLREMRRTLEGKYTQVPQLSAGRKLVLDQPFRV

>FcMC5 FC073G00110
FDEQAEEAIPADVRMISGCHDEQTSADVNNVGSFNLPDPAGRSGGACTSAILQVVYKEHQNSSKHLTFMDVFLQTRD
VIKSGGFEQIPQLSSSRRIDINQPFDMMSTNKSKQGTRYAVLIGINYTSHKQGQLSGCHNDVQNIRKYIMDVGNVQS
KNITILMDDGGKHTDPTRGNIMNALTDLTRKCQAGDIAFVHYSGHGGRVKDETGQEESGYNSTLVPVDFHIAGQIID
DELYRHLVCAMPRGTTLTCLMDCCHSGSVLDLPFNFVADGEQTEM

>MrcMC1 MRCC299_04G00220
MPSASKPPTGASNPTPKPRDAQKEKDYERKKQRVKEAHRKKRVRALMKSAPGEPVKLHYDTDCRSPATEAHVVMFSG
VSSESKGEIAGSSGMGWYGMPRRAGKGGAGGACANALLHYLKESDRTKPPDRSWLTTMVGMQKWLSKEGYTTVPRLS
AGNNNFDFKRPFSILNPQCVNSQRLSGRTRAVLIGCNYPGTESALDGAWADVSKMKRYIASVGFSNDGDSLMVLRDD
PNGKSGELQPTKENILEALHWLALGAAEGDSLLLHFSGHGVRVNRPKASETDDETVVAEDGLVPCDYKTEGPILDRE
VQEILVSRLPKGCSLVMFLDCCRGGSAVELPYNFKLTPKQYAFEKERMAVGWPVRRNPLAALVDRTVNMVPNNCKRA
LRIGAKTADATCETIGGVLQAAADSFLPKLIKREYQRGKRKIHKLTGVKVQFEPHDERTHRFLF

>OsMC1 OS01G58580
MGRKRALLVGINYPGTKAELKGCHNDVDRMHRCLVDRFGFDEDDIRVLLDRDSSGTQPTGANIRRALAQLVGDARPG
DFLFFHYSGHGTRLPAETGQNDDTGYDECIVPSDMNLITDQDFRELVQKVPNGCLFTIVSDSCHSGGLLDSAKEQIG
NSTRQNQTQSREREEPSDSGSGFRSFLKETVRDVFESEGIHLPRSRHSQSHYGGEDQYETYAQPTDGHTKNRSLPLS
TLIEMLKEKTGKDDIDVGSIRMTLFNIFGDDASPKVKKFMKVMLGKFQQGQSGEQGGLMGMVGSLAQEFLKVKLEGN
EEEAFKPAIEQEVHSVDEVYAGTKTWAPNNGILISGCQSNQTSADATTPQGSSYGALSNAIQTILADKRGNVSNKDL
VMKARSLLAKQGYTQQPGLYCSDDHVHVSFIC

>OsMC2 OS03G27120

MDHFGGRALGFGGGGGCGAVRCRHCSASLPAMPGARVIQCAQCYGVTRVGGRGRRRHPNPVEPWRPAVPMPVAGGGF
FPGSRGKKRAVLIGITYAGMRRRGSQLMRGPVNDVKCMRYLLCERFGFPNDCVLILTDEEKDPCRLATKENIRMAMN
WLVQGCSSGDSLVFHFSGIGVQVPDDDGDEVDGYDEAICPMDSFSQGPILDDEINEAIVRPLVHGAKLHAVVDAEHS
STVLDLPFLCCLSSRSGGWQWEDHRPPTGAYKGSSGGQAMLFSGCSDGNNKHSLLPEASTVGAMTHSFIKAVECEPR
ATYGSLLTTMRSIMRDGGVTCNLQGPIGAPIRKVANFSGIQEPNLSCSEMFDIYRKPFVL

>OsMC3 OS03G27170
MGCNCLVGKGGNTRPAATTSARPSSSRCAHCGAGLAVPRPGPGGAAAVTTVRCALCHRMTCVDRRGGRDLGGGGGGG
ALEASSSSWAPAEASFLRRDAPSGYPFVPGRKRALLVGVSYKGSSYELEGTVNDVDCMRRLLGESFGFPADSILVLT
EELGEGDPSRSPTRANLLAAMRWLVEGCDAGDSLVFHFSGHGVQKLDVNGDEVDGYNEALCPVDFERSGKILDDEIN
ETIVRPLVAGAKLHAIVDTCHSGTILDLPFLCRLSRTGYWQWENHCRRPELAKGTSGGLAISISGCSDDQKSADSSG
FSSEQAAAAAAIGAMTYSFIRAVESEPGTTYGRLLAAMRATIREGQQGSSVRRLLPGRLGSFVRKMIPSGGVQEPQL
CASEVFDIYRKPFLL

>OsMC4 OS03G27190
MAVVSGGKRALLVGVSYKGDTSRELTGAAEDVKNMNSLLKKFLFPEESIHMLTEELGAKDPLKAPTRENIMKEMRWL
VEGCRAGDSLVFHFSGHGRQRKDDNGDEVDGRDEELCPVDYKVSGNILDDDINDAIVKPLTQGVKLHAIIDTCHSGT
MLDLPYLCRFNRMGLCSRYKWVGQTRWRLSPKKEWAMVPVGGHAISISGCKDYQNSLEPDNTAGGGVMTWSFLEAVG
SRRTMTYGELLDSMRAKVHHRLQQSSSGKCLVTGCLGSLAAKCLPCCFLSVQEPQLCSSKEFNVYEEQFIL

>OsMC5 OS03G27210
MASARPPRGTAWCGGCGAYLAVPPGARSVRCALCRAVTRVERRGHHGGHGGALGFIKGLISAFAPPPPLTPSAGAAA
AASYYPRVSGKKRALLVGISYAATGYELKGTVNDVNCMSFLLRERFAFPADCILVLTQENGDPYRVPTRANLLAAMR
WLVEGCSAGDSLVLHFSGHGVQKLDVDGDEADGYDEALCPVDFERAGVILDDEINETIVRPLVAGVKLHAIVDTCHS
GTILDLPFLCRLSRTGYWQWENHCRRPELAKGTSGGLAISISGCGDSQTSSDTTAFSGGAATGAMTYSFIKAVETEP
GTTYGRLLSAMRATIRGGGGEVGIPGPLGAFFRRVITFSCAQEPQLCASEPFDIYRKPFLL

>OsMC6 OS05G41660
MGRKRAVLVGINYAGTEGELKGCLNDVARMRRCLVDRFGFDEADIRVLADADPSTPQPTGANIRLELERLVGDARPG
DTLFFHYSGHGLQLPIETGGDDDDTGYDECIVPCDMNLIKDQDFTELVQKVPDGCLFTMVSDSCHSGGLIDKTKEQI
GSSTKQSKIQQRERELRRQQAPSPGTCSCASLLQIALRHLPRRGGQRIIGSRSRDGVGEDQPPRSQAELLAADATRA
GIKNRSLPLSTFVEMLRERTGKDDVGVGSIRTTLFHHFGDDASPKIRRLVNAMLGRRHGSATASEEHPDKAKPERVD
GEGEAAAAKQGAPETRPLPRNGVLISGCQTDETSADATTPEGVSYGALSDAIQSVLAEERRGKVTNMELVRRARELL
AKQGYTQQPGLYCRDKHANVAFIC

>OsMC7 OS05G41670
MGGRKRALLVGINYPGTKAELKGCHNDVARMRRALVDRFGFDEADIRVLADADRSAPQPTGANIRRELARLVGDARP
GDFLFFHYSGHGTRLPAETGQDDDTGYDECIVPSDMNLITDQDFTELVQKVPDDCLFTIVSDSCHSGGLLDKTKEQI
GHSTKQNQAQQIKREERSDSGTGGFRSFLKETLKETVRDAFESRGVHIPHQSSRRNDDEDEEPHMGSSSHGGDRIKN
RSLPLSTLIEMLKEKTGKDDIDVGSIRMTLFSLFGDDASPKIKKFMKVMLTKLQEGQHGGVMGLVGALAQEFMKAKL
EGNQEADALEPAMKQEVHSVHEAYAGTTARVSNGVLISGCQTDQTSADATTPKGVSYGALSNAIQTILSEKSGRVTN
KELVLRARELLSKQGYTQQPGLYCSDKHTSVAFIC

>OsMC8 OS11G04010
MERGQKKKLATLVGCNYAGTPNELQGCINDVAAMRDALVARFGFAPADVTVLTDDRGSPVLPTGANIKRALADMVAR
AAPGDVLFFHYSGHGTLVPPVKGRRHGHGECDEAIVPCDFNLITDVDFRRLVDRVPRGASFTMVSDSCHSGGLIDLE
KEQIGPSVLAAGAAPAAAAASTTATRATAARFLPYAAVVEHLSGVSGVDAAHHVADHLLALFGADASAKFHRDAEQP

VRTDDDGILLSGCQTDETSADVPGDDEVAAGGKACGAFSNAIQTVLASHPAPVSNRQLVSMARAVLSDQGFEQHPCL
YCSDGNAETPFLWQEEDKKKTVTAAAEQAMTAL

>PpMC1 PP00016G01500
MGGQRRIKCSSCQVETLVTLSPGTRSIICEHCLAVIQVTPVEAPSIPSKPDTYSPRHVLPYWEGSKRAVICGISYGG
TSIELKGCLNDAKCMSYLLMSKFHFPESAILVLTEDQVDPRRQPTKYNIMQALEWLVQGCQAGDSLVFHFSGHGSQQ
PNYIGEELDGFDETLIPVDFMTAGQIVDDDINTTIVRPLPTGVDLHAIVDACHSGTVLDLPFLYRYSGHEAFVWEDH
RPATGTWKGTAGGNVYSFSGCDDHQTVFDTMNLGRSTSTGEMTYCFIQAIERGYGTTYGSLLNAMRSAIHMAENQLV
TSLIDMLLKGGSYEGGDTPEPQLTSSRCFDVSTPFRL

>PpMC2 PP00043G01330
MVRRALLVGCNYPGEPCELKGSANDVDRMHVLLTKKFGFKPTEILVLVDIDPRSRQPTGANIRKSLRKLVDGAEPGD
VLFFHFSGHGTQVPPDRGRRDETGYEECIVPSDMNLLTDDDFRELVDRIPPGCNFTFIADACHSGGLIDNEKEQIGD
RYSMGEGGPLGGRPRPPLRPEAGGGGLTDLVAEGIEAFSGAGGGKQNSFMDAGVRYGGYLDSGDYGYSGKPHHTPLK
NKSLPLNILTSILSQRTGRSVKPGNIYQNLYDLFGEDASPTVKVFVRILLNKLEGEDGGRSHGLLGKIGGLASSLLK
TKIDNDPSGGYPEDRQGEELRRHRRRQRRNYDAGILISACEPHESSADANPTNDPRDAYGALSNAIQTVIGESRGYL
TNRQVVMAARRLLNEQGYKQHPCLYCSDRNADAYFLQPMPM

>PpMC3 PP00043G01350
MSKRALLVGCNYPGTKVQLHGCANDVRRMKALLIDRFGFDERDILVMLDTDPALPQPTGANIRKCLDKLIENTQPGD
CLVFHYSGHGTQVPAESGQEDDTGAEEAIVPTDMNLLTDDDFRELVNKIPVGVTFTFLSDSCHSGGLIDSTKEQIGH
TVKDVEEGEEKRGFRGFLSKAKAKYDSHKGKRKEEERPDTQNFDFESQYMEETGHQVKNKNLDINSLTEILSQRTGH
EVQVGNIRTTLFDMFGDDASPKVKTFVSVILNQLQSGAGEGGFMGMVSGMAGQFLKSKLESDSPDHISSLMAAAGSA
HPSNPRSAYAGVRPSASHRVREDAGILVSGCQHNETSADATPGGDHSQSYGAFSNALIGVLAKHDGPITNRDLVLQI
RESLAKSGFKQHPCLFCTDQNADAHFICVD

>PpMC4 PP00065G00020
MLVNCGRCRTPLVLPPGAMSIQCALCRHVTPIAYDRAAPSHSTPQISDPYRNSSHAPQYTPTPPNVHGRKKAVLVGI
NYFNSRHMLKGCINDSNCMRHMLTTKFGFPAASILTLTEEQPSAVMKPTRYNMHMAMVWLIQGCQAGDSLVFHYSGH
GSQQRDYSGEEADGFNETLCPVDFETAGMIVDDEINDTIVKPLPHGVRLHAIIDACHSGTVLDLPFLCRFNRYGQFT
WEDHRPANRRWKGTSGGQAYSFSGCDDSQTSADTSALSKITSTGAMTFCFIQAIERGHAQTYGSLLCAMREAIRTTK
VNSGMGTGTITSLMEMLVSGGSLTGGLTQEPQLTANEAFDINSPFHL

>PpMC5 PP00165G00360
MSKRALLVGCNYPGTKCELHGCANDVRRMKELLMNRFGFDEIDILVMLDTDPSLPQPTGANIRKSLAQLIQSTEAGD
CLVFHYSGHGTQVPAESGEQDDTGADEAIVPTDMNLLTDDDFRELVNQIPVGVTFTFLSDSCHSGGLIDSAKEQIGN
TGISAFSGKRDVEEGEEEEGGFRGLLSNMRSRFGSGNRDNDEERPDLQNFDFESQYLEETSQQVKNRNLDINTLSEM
LSEQAGHPVEVGNIRTTLFDMFGDDASPKVKVFVNILLSRIQSGGEEGGFMGMLSSAAGHYLKSKLDSESPDEAANY
MAAAGSVHPSTARAAYGGVRPSASHRAREDAGILLSGCQHNETSADATPAGDHSQSYGAFSNALIGVLAETEGPISN
RELVLKIRESLASSGFKQHPCLYCTDENADAHFICVTD

>PpMC6 PP00373G00150
MPTKKALLVGINYEGQPHHALRGCWKDVERMGECLVSRYGFPKESICTLVDRPGTSPDLMPTGEIIRRKLEELTRDL
KWGDCIVFHFSGHGLQMPPEGEPDETGMKEAVVPVDANMITDDDFRILVDKIPDGVFFTFIADCCHSGGLIAHCEQQ
VGSHAPEDRYAAPNFGNSHEGFRGSYNGNLETVQPGAWPYGGANLGQSSGRYDANLATSKYTSKRSMPVHAVTQMLS
ARAGHTVEPGNICANLYDLFGDKSSVTCKEIVHTVFNGLRSKGGSKREILKRISSKSIDFLSAKLHSSSLRDSNYQQ

QTATRDLTDATPENLSHIPKRPDRCILITACQSDETASEHRNEIHGAFTKTLLDIVDEHKGPLDNHRLVYECRQRLA
RKPYGQHPCLYSTPAQAHHVFICY

>PtMCA-a PTI_02G05190
MGNADSKATTSLAEAETVPTGIGRKESCLRSFNDSFEETKNTAADPIHPISGRSDRKETAQARDDEISSHITDDQVH
VNLAMADLMAYLQVVANNSSHLPLTRRDDPDLDRIEGSLASEEYGKKSAAFIPSDVRVIAGVFTRYGRVWDLPTSEE
YNAVDGNHEPGRSYGGACCNALLKVLYDAANDHVDAFHAVAATSASIFDDDDEEEDLYAGMGQSYKSGCSLEYTNPY
SVTITWCDLLRRMKAEMKDIEHVQVPKISTSRKIDLSKPFSLIPSNFDPINGQKRSLLIGCNYSDIPEAQLKASHDD
VRSIKDYIVNVHGFPEACGLMTVLMDDKNHKKPTFLNIVEAFKALSEEAQPGDAIFIQFSGHGGRVLDSHIDTEAES
YDEVLVPCDYLQSGLIRDTLIFKTLLAPMRYGVTVTILIDCCDNGMVLELPYSWSTKGDRRSSQPKLALNEDFSFKR
FLKVVRTLYESSSFTQLGRTVDIALNPMNATFKSTTITNDESSVLFEQPEKAVNAASRSSILDNFSLACSTKTLSTP
RGDGDGGIVNSTGLSVMKHYSLTKQSLLQQVMNCTLLSPEVDEDFSFDETYQTRTEDHNTFDEEDRSVGYNSSFRSC
VTEYEEESRRRGRRRRR

>PtMCA-IIIa PTI_16G01290 (plus manual correction)
MASFDEKIKQAIPAEFHMISGSHDSQTSADVYNTGKFQLPNPAGRAGGACTSALLQVLYNNGVAAGELSWVQCLRQM
RTALNGMGFDQVPQLTSSRLIDVNTPMAIVPPTATGRKRAVLIGINYTGQQGQLSGCHNDVKNIIKFLTKVHGFNET
EMLILMDDGQHHSPTKKNIEDAFTRITQYSQAGDVVFVHYSGHGGRVRDLDGDEDDGFDETLIPVDFKRAGQIIDDD
ILKILVKPMRQGVTVTVLMDCCHSGTVLDLPYRFSADDSKMRIDQGNANSLFGKLGDMDVAQVACCACLAYVVADML
LN

>PtMCA-b PTI_08G02260
MGYEQLPQLSSSRWIDVNRPLTIVPKGSGRRRAIVVGINYVGQKGELKACHNDANNVLKYLIEAQGFDPSQILILMD
DGKHTEPTRRNIEDAFVRMTQYSQPGDVVWVSFSGHGGRAVDISGDEDDGYDETLIPLDFMKHGQIIDDDILDMFVK
PMKKGVNVTGDFRSRDDFPWKVLMDCCHSGTVLDLPYTYSSGDQKMRTEKNFDFGDGKNATNAKKQKEMEKAERKAA
GENSDSDDEGDKKKKKKVKEKEEDPNEGLYDQSPIPPPQPRPPTRDLPPPPTPPNQCCRIL

>PtMCA-IIIb PTI_16G02000
MSNYLERAEELIPAEVRMISGCRDEQTSADVSNVASFSLPDPAGSAGGACTSAMLKVLYANHKAPQKDLSFQEVLMK
MRGILSQGRYTQIPQLSSSRPLDIHQSFNIVPANFTGTRRAVMIGINYTGQQGQLSGCHNDVKNMIEFIKDIHGFED
ENITILMDDGAHTEPTYKNILAAYHELVSSAKAGDAIFCHYSGHGGKVRDDDGDEADGYDETLVPVDFNAAGQIRDD
DIFSALIGPMPAGVTLTSVMDCCHSGTVLDLPYVFKADGEQNQMAPPPDFDFSKLAAMFQAYMVQQGANGQGGIDPN
DAIAIVAKECCNIL

>PtMCA-IIIc PTI_16G02030 (plus manual correction)
MGFLRRQLREQFEKKKPEALQADIRMISGCQDVQTSADVSNVASFQLPDPAGNAGGACTSTLLNVLYKDHQTPEDTM
SFVELLNKMRENLEAKGFSQVPQLTASHPIDVNVDFDLVPPAATGTRRALLIGINYVGHEQGVLRGCHNDVKNMVEY
IKAVHGFEDENITILMDDGEHTAPTHANMIAAYKKIVALSKADDALFCHFSGHGAKIRDDDRGEEDDGYDETLVPID
YHENGMIRDDDLYDILIKPLVQGVHLVCLMDCCHSGTVLDLPYVYKADGNFTEMEIDENFDFKKLLGKFGIDDFDKF
GGEALGKINGDALGKVGKDALGKLNKFFG

>TpMCA-IIIa TP20G01470
MGGDDDNNNEGHTMTKEEFEATKPDELRKDIRMISGCADHQTSADVSNVSSFQLPDPAGRAGGALTSTLLKVLYADE
QKTDFTEVMERLRGHLKGRYSQIPQLSSMNPIDVETKFDLVPDSATGTRRAVMIGINYIGDNPGELSGCHNDVLNMK
KYIMDVHGFEEDNIVVLMDDGEHTEPTHDNIMNAYKKVIADAEDGDAIFLHYSGHGTKLRDDDFGEEKDGYDEALCP
RDFASAGMIRDDDLYDILVKGCPDGVHMVSLMDCCHSGSIMDLPYIFKGDGSQTEMILDPDMNIDAFIEQITGKLVE
FIKAKMAAGF

>TpMCA-I TP10G02670
MRRIPNTPRGSIGIPPNGRHKALIIGINYTGTRAALKGCVNDAKSMQQLLMRNGFGDDGSHMLLLTDERSRGREYQP
NATNIMKAFAWFMKDVQKGDVLFFHFSGHGGQVPDKTGHEADGFNETIVPLDYERAGQISDDVLWGSLVYPMPEGCR
LIALMDMCHSGTGLDLPFDYNVDTRRWKEDVNPAHSPGDVVLFSGCEDAQTSAD

>TpMCA-IIIb TP02G04700
MTDKKRSSKSGKASSSIRTPPPPPKRSPSSGGRSKSGRSIKSSADATPTLSSSATRGTKKNGDAEPRREQSRSKSRS
RSKSRGAHDRDGRRSRSKSRPKQQQNKQEQQLVVHSSEARPKQRKSKSSKSLSNKSKSSKSNNGSSARSAPSPSSKS
KGNKAVASEFNVKKRQQKFANQAKKMIPAEVFMISGSHDAQTSADVSNINSSFALPNPAGKAGGACTAALMQVLYDA
YDDRREDSLSWVDVLREMRDVLDSKNYDQIPQLTSSRMIDVHDPFVITPKSFSKKNNTQRAVLIGINYVGQKGQLSG
CHNDVLNVAKYLKEVQGFRKENMTILMDDGNHKSPTKSAILSAYKKIVKESKEGDVVFCHYSGHGGRIKDDNGDEDD
GHDETLIPVDFEKAGQIRDDDLLKILVHPMAAGVRMTCCMDCCHSGTVLDLPYRFTADGDVEEMEMNERVNFTDALW
TGMSIAVGVAAGNAVASAVTSAVAPEMMILGGGPNAIQ

>TpMCA-a TP05G02130
MPSNMSWADLLRKMKTEMQGVGFSQAPALTSSYKFDLNTPFSLIPPNFKAGINKKRALLIGCNYRKTPDAGLKASHD
DVRSMKDFLVNVYGFPETSDLMTVLMDDKHHQHPTHENITEAFKRLAEKSQPGDAVVVLFSGHGCRVLDSPIDSEAE
SYDEALVPSDYNVSGNIRDTLVFKTLLAPMKKGVTMTCIIDCCHTGMMMDLPYIWSSKNDKGEQLPKMSLNNEFSFV
RFLKVVKTLYESSVFTKIGKTVGSELDKQLPAKDDETVTETVGSLVTIPENEPPKREASLFQLLSCQNPQCLATKVI
NCTLHEPEYSDDDTIPKDNTMDEFYDDVSHDSYSTGSGR

>TpMCA-c TP18G00030
MTIVENPTGVKRAVLIGINYVGQQGQLSGCHNDVRNIQDFLQRVHGFQQHNMTVLMDDGRHKEPTYAKIMAAFDWIV
KESMAGDTVWIHYSGHGGRVADQDGDEDDGYDETLIPVDFQRKGQIRDDDLLKHLVKPMRKGVVVTALMDCCHSGTV
LDLPY

>VcMC2 VC0004G01350
MPSKRAVLIGCNYPGTNAALRGCINDVWGMKAILEEFFGFGPSDITILIDTDPQYLKPTGKNMKAKISEMVAAAQDG
DVCVLHFSGHGTQIPSYGGDEKDGKDEAICPTDMNVICDDDLRALLKPLEAKPGVKFTFIADCCHSGTLLDHETVQI
SGPKDGAPRPPQIDMGVLSSLFGTLGQPDGRDFKNRALPFNDLCGMLSELLGGAPVDARNVRSNLGTLFAGDASAKV
QQFMQVFQMFTAGQKEGGGAGGVPAMGLLQLLCACLAPSADQGSGGPTGQPANANSGPGATYAVGNLNEPDLKINLP
TPGTKPPGDQQLSSDVGILITGCQSHETSADACPSGNPDKAHGALSNAIQTVVRTHHQQNPGQPLTYRNLVISVREL
LAKTGFAQNPCLECSDTNADTPFIMH

>Pn1 jgi|Psemu1|257123|estExt_Genewise1Plus.C_2100062
MTDARIKNAIPASFVMISGCEDKQTSADVSNVGAFSLPDPKGKAGGACTSSLLKVLNESGGREMSWIDLLHRMRSVL
TSKGFDQIPQLSSSRMMDVNNRFEIVPSWAQGRRRAVLIGINYEGQQGELSGCHNDAKNIQSYLINEQGFRESDMIV
LMDDGVNNPPTKRNIIDALDRVVEYSQAGDVVFIHYSGHGGRQRDADGDEEDGYDETLIPVDFKSAGQIVDDDLYAH
LVLKMREGVAVVALMDCCHSGTALDLPYNINATESEMHATEGFNFGLLDNAQMAMGCCFLLMSLLSMFD

>Pn2 jgi|Psemu1|188031|e_gw1.73.141.1
MFRRKNRDLRHGEDVATSISNNNNNNNDFDKQAQYLVPAEVRMISGCHSLETSADVSNIHSIVGRGKLPSPEGRAGGA
CTTALLSIFITFQKLLLELRRRLAQTGVSQIPQLTSSRPLELEETPFSLRGTPREGRGGTATATATQRALLVGINYY
GQNGQLSGCINDVANVKKYLCNHCGFEEKHVLLLIDDGRNHHPTRDNIIRALRRLVEHSKPGDSVYFHYSGHGGLLD
PNYWNRFKAGVSKKEYDETLYPVDHARAGQIRDFNLFHHFVKPMAAGVTVTCVMDCCHSGSVLDLPYSYR

```
>Pn3 jgi|Psemu1|188378|e_gw1.76.57.1
MFGTLGKKYAAQVAEKVEQLALQALNERNPQTVQNEKTTEQLHKISGRRKAVFIGINYKGQKGELRGCINDVKNIKN
FFQTHYQLDDVLVLTDDKQAEPETKYPPTRANILNAFKWLTKGAKPGDSLLLHYSGHGGKVKNHDGTEASGYDQTLI
PLDHQKSGHILDDDVHDALCRKLPKGVRLTAIFDCCHSETIMDLPYVYNIDGNLDIVLNDKNESVATLVSAGTRFLL
DGNKKKAAKVFQAELTNLVKGAVLGGNPEADSARRKQNIENNETSADVIMFSGCKDDQTSADATIKGEATGAMSFAL
VKTLKAHHKDSKRITYTELLREMRQVLEGKYTQVPQMSAGRKLMLDQPIQI

>Pn4 jgi|Psemu1|320764|estExt_fgenesh1_pm.C_7970004
MKDENRDDILQRRNSIIKDDQVLVNLAMADLMAYLQVVANNSNNLPLTRRDDPELTRMVTNLSSEVYARKSAAFIPA
DVRVIAGSFLKYGNVWDLPTSEEYVASDGAQEPGRSYGGACSNSMLKVLYDAASEAADVGNIDKDACDALFDDDDDE
SLSTLPLTRNNTFASLDMNGQSNPSTITWAELLRKMKTEIDEIEYVQAPTIGATRKFDLNQPFSLVPESFDKSTGKK
RSLLIGCNYNGTEGAELKASHDDICSMKDYIVNVHGFPESDDMMTVLLDDDQHRPPTFMNIVEAFKSLSEESQPGDA
VFIQFSGHGGRILDSPINNSVESYDEILVPSDYSKSGVIRDTLIFKTLLAPMRYGVCVTIVLDSCDTGMVVDLPYSW
STRSDKAGSVAKMKQSENFSFVRFLKVVKTLYESSTFTQLGRTVGSALGEQPTVDESSDEEADEEEEEGEKTLSTIE
RTKTEEMQEGSVLGLCTPRGRAADSTYSRSENNDRDAFSLIEKMLGCNFLVHDLDDDFSDGETFENTTYNTFDDTHT
MTNASTFDSLSDGEYRRSSRRRRSRRN

>Pn5 jgi|Psemu1|320057|estExt_fgenesh1_pm.C_3800004
MATTYDEQIEEEIPGTFFVFSILDAEEDEERNAVVSSLEDYELPGNVVQGETSSGGVCAYALLNVLKQNKNGAGKKK
KKPPKKTWADALEAMQAEIRNERGLDTYTPTLSTSRPTDLWSEPMQIASSSSEGVKRALLIGLHYENDGEDDGDDDD
DDDDDAEDAKLSSCHDDIRKMRQYLIHEEGFEPQNILVAMDDNGRHHEPTKEFILECVVRLCEISESGDSIFFHFSG
HGGRLLDEEEDDDCEIPHELLAPSDYRKGEILTNDELYSSFVREVPAGVHVVAIIDTCHPSPSGCAMELPYVCEAGD
DEVRDSGGFRSARAIMATVTAAAGTATVASKKKKKGKDVEKKGKQKKKTVDSDNSDDESENGETEDENDEEESSQSE
KGKTEKKPNKKKQKKDEEEEEAEEMEVPNDMEESS
QSEKGKPEKKSKKKKKKKGDEEPPPKEEEASEEQEEDEKPKKKKKEDKKKKKSKKQKDVSNSSDDVGSEGDDDPPEQ
APKKKKKEKEDAPKKKKKTKKK

>EhMC1 jgi|Emihu1|43172|gw1.322.10.1
TRASLNGNRRSVLIGINYSGLSSPSELRGCVNDVKRMRPFVERLGFPSNEGSQMVLLDDGSTPLAPTMANIRGAIAW
LVEGAAAGDALFFQYSGHGGRIQGSDGFHETLCPLDMASEGQLLDTELFETLVAPLPSGCRLTVILDSCHSAGALNL
PFLFTGTEGNLKSALAGEAVQMAMSRNWLRDVIAITGCRSDQTSADVGNVDLQFDLQPTARSGAGGALTSAFIEALD
GREAAPSYLELLEAIRLKLAEEGFTQVPQLASSLLVDLTSAFSML

>EhMC2 jgi|Emihu1|202860|gm1.1700383
MGAQPSLFDVHAPALDPRDVTQVFELLDKDGSGRLAGSEVTKMFAAMCGRPVKVHGAKSSYSLNEFIEAVEDMGRRQ
PFLNITGNLVKYVRENKEKMKPVDAFSDERIEAVSTFLDKDKSGAIEPDELLRFLQYLHIPLFTIFGVVNSSGVIKS
NAQLQRLLRKLAKQCPQLEIEQKIIKISGDIHAGDASKPPQYQEATASAEPPEPAAGRRKKALLIGINYTGSRMPLR
GCINDVKSQYNVLTTKFGYSSSEIRLMTDDTNDSSKVPNKKNMMAALKWLVEGVKPGDELFFHYSGHGSQCPDRSGR
EPDGKNECLCPTDCTKGFPEYVITDDQMQNFFSQIPEGAKITCLFDCCHSATMGDLCVNRELGLEEDIVPSTKQVWT
LSGCQDNQTSADAVIGGVPRGAFSWALMGALEDNNYKLKYDDLLAQTRHKLRRRMGEGSGDDSHLFRSAYLETSWCG
SSWTALQLGGRYFCIDMRP

>Ehux1-EhMC5  metacaspase-scaffold16
MELAAGAGVAAAAAAGGAAYLRSRRGPASGKEGTTKWQWRAAAVKERRALCICCSYPGTQWALPGCVPDQEAMVKTL
IRNSYAVTFLADHTPAWGQPKPSALSNRRNILAELEALCRWLHAAPGRQGWVSYSGHGTQTADRGGDEADGLDEAMV
PCDFKMAGLLTDDKLRTTIRFGAGSSLMFFFDCCHSGTILDLPYTLRHTLSGPEYPIETEAGAIFCISASKDDQSAY
```

ETAEGGVCTRAFLGAYTPSVSPAATLRLMRRYAERKGMPQTIMMASNRPFTEADASFVEAEPEGGGGFCAAFHRCQN
RLGCSKGRSKAGDFGGENPAARSLELNRGEASGRGPKLHPSCPDPLSTTKGRAYVAARRAKLAAPPP

>Ehux2-EhMC8 metacaspase-scaffold276
MSAAVMSLQPHVFTRYEWLPKSTSGTSTKHRALCIACAYPGTSYELHGTVKDQEALKKGLEDNGYSVTFLSDADDKH
AVPTKANILKEIANLCDWLNEEKGRMGWISYAGHGTQTKATVAGEEPDGKDEAMVPTDYPTAGLLTDNELNQTMLKS
HKVQNGCAMIIFMDCCHSASILDIPYALETEPVGPKYVEDKSIGDIFGVSAAADDKVAYETAAGGVCTRAFIEAVRK
LGKGALPAEILLEMRQYAKDQNMDQLITMHSNRQFTFGDFQIMKGVDVVKEAAPPVSAPPAPAPATAPSPAAEPPGH
AGYNPAAHTSHVYTAPATSKPDLTKVKGGEELNRPPAQAPPTAPATSGKKAGGKGKAGTKPATQPQSSCCVVC

>Ehux3-EhMC6 scaffold120 440238|estExtDG_fgenesh_newKGs_kg.C_1200066
MTDQSAAGPTTNFNYLKTGAGKTERKALCICCSYPGTQLALPGCVRDQAAMVDMLPKQGYDVTFLTDGDNYHEKPTK
ANIVREMKKLCAWLSEKEGRQGWISYSGHGAQVPDDEIQKVGYSEEKDGMDEAMVNIDYQSVGLLTDDELKEVLVFS
KGSSLMVFMDCCHSGTILDLPYRLTHEATGPAYDVIPDCGTIFCVSAALDEQCAYETSEGGVCTRAFLGAYRKGQPP
GQMLQKMRAYAQAKKMPQQITMNSNRPFTDADGSFIDADPASGICESLLNCLTGETSATRSIEIGNKQDKGQIPPSK
PCTAAGKILPQPTKDYVKSIREKNGYVAQL

>Ehux6-EhMC3 scaffold111_jgi|Emihu1|223690|gm1.11100063
MPNSFTGWMWESKAATAGDRKVVTKALCVCCSYPSNTEVKGLPKWASTGKHALPGCVGDQMRMKDMLKKEGVKVRFL
TDHKKLSKEKDYSTKKNIVAELKKLVKWLDGAKEDGPVRQAWFVYSGHGVINPKRPYSLLPEDAPDPGGHGFVMLES
IVPCDYFTTSELLSEVELEETLQGFNENGALLAFMDCCHSGTILGLPWDLELDESGEMVGCRAPHPEAIIERYSKCG
RILCISAALDAEVAYETKAGGVCTNAFLESHNKGDPPAKVLIAMNQYARDHYMKQTINMSSNVEFTDADAAVIAGGI
PHWSRSLGKIMSAPAALARSLAPKPQPVVERAVAFEKHPSAGAVSDFLYATALARHAGECGERALEIASQGASKAAR
LASTGKAPEEAADEANTYARGLIDDGVARRRGL

>EhMC7-9 correct65 452661 471262|estExtDG_fgeneshEH_pg.C_9100002
MGGCSSSQKTTAEPEQQRTPRRRALLVGMDYRGAPAGWPIINGTRNDVKVFDTLLREHWGFSEEDIVTVTDPAEQST
ESLLAALTAFKASLRPGDTAVIFYSGHGKQVFDANGDEVTRKGESAPDIMDEALCTTNGTLLDDDLTVLMQGIAASA
AQLFWFCDACYSGGFVDQGSGSSLPENVVLIASSLETQKSEDALRIETDKETGKMAMFYQGTGTRWLKAAVDGGGLP
PRATHQQLFDALAAERAKDKRTSVQNPFIVASATARQLTLWM

>Amphiprora1 CAMPEP_0186511138
MGFLKKAFRDVFEDKKPKELKADIRMISGCEDAQTSADVSNVASFCLPDPAGRAGGACTSTLLNLLYKDHKDTRDDM
SFVEVLTKVCIVGSQASAPLWMMDDSLRVGVAVFRRHNNRCEKIWAAVAFPKFPNXSASSPIDISTTFDLVPDGATG
KRRALLIGINYIGHSQGVLSGCHNDVLNMVEYIKEVHGFEEDSITVLLDDGEHDAPTKENILAAYKKMVAEAEPGDS
LFCHYSGHGCKIRDDDRGEEEDGFDEALVPVDYQTAGLIRDDDLFDIIIKPMPDGANLFCLFDCCHSGTILDLPYLY
KADGTMEVMEIDESFDFKKLLGKFGTLVDGFFD

>Amphiprora2 CAMPEP_0186511756
XAIPAEIHMISGSHDAQTSADVFNTGQFQLPDPAGRAGGACTSALLKTLYRLEEQGGANGKVSWVQLLRTMRGELRQ
MGYDQVPQLTSSRLIDVNKPMYMVPPDFKPAAGRKRAILIGINYVGQQGQLSGCHNDVKNIKKYLTEVQGFNESEML
ILMDDGNHHPPTRKNIIDAFKRIGEYSNAGDVVFVHYSGHGGRVRDTSGDEDDGYDETLIPVDFQRAGQIVDDDILK
ILVKPIKAGVYVTVLMDCCHSGTVLDLPYRFSADDSQMRIDQGFNMNSMLGKLGGAADVAACACLGLCVAQLLADIL
AG

>Amphiprora4 CAMPEP_0186484142

XMRFRSSSRRHGQNGAGETNTSQKGSAQQFSQQVQHLIPAEVRMISGCHSEQTSADVVESTSRDFSLLPNPKGKAGG
ACTSALLDILYEHHHRRSSLSFQQLLLQLRSALAQRGFDQIPQLTSSRPLEMQQTPFSCISPYEGNPQTPHQQNCRR
ALLVGINYRGQSGELSGCQNDVYRMRDYLCQVQGYLPSNVVLLVDDGKAHYPNRKNIISVLQQLVLQSTSGDSVFFH
YSGHGGLLEPSLCNNAWKRSVEDYDEILYPLDHKQAGHIRDYSLFRHFVKPMPAGVTVTCVMDCCHSGGVLDLPYSY
KATPGGTIAMERSMGSLNNLALLYLLAGGRLPGGGLFHNVSDSIQGVTGESVESLQGAGMTEEDAQDGITDFTGDNG
GDVVAESIENDITADDLGDDDIAPGDLGENDIGADDLGENDVGTEDIARTGDFPEPMPPPDPLLEPPVFDTNGNPIS
DTGLDGPIGAPPIFDSNGDQYGGGDAVYDTNNVPGYDDNRMAGFAGNSDLGFMQASPYGYDQGGRAEYEYGGTDYGG
MGDNGEDADCDDCGGCISELLQNLADDE

>Amphiprora7 CAMPEP_0186516142
XTLRDTKDQHRIQTSIMPTREEVLAKAEEYIPATVRMISGCRDEQTSADVSNVASFKLPDPAGRAGGACTSSLLQIL
YADHKKPEVDLSFQETLLAMRKVLSAKGFSQIPQLSSSRPLDINQTFDLVPPEHVSGTRRAVVVAINYVGQQGELRG
CHNDALNMIEYIKDVHGFPEENITVLMDDGQHTEPTRDNILAAYRKVVRDSEPGDVVFTHYSGHGGKLRDQDGDEKD
GYDETLVPVDYASSGQIRDDDLFKYLVAPMAAGVVMTCLMDCCHSGTVLDLPFEFVADGEQTEMHVKPDFDPQTLIN
LFQAFQTFSNGECSPETLMEQAQMLKGCNIL

>Amphiprora8 CAMNT_0029300213_6
YTNISYCFACVVTTTTTTRSIHNMGSGNFDDKVHKCIPAEFFMISSSQDSEISNEAYNTGSFELPDPAGKAGGACTS
AILEVFYSRGHEVGQGMSWVDVLKDMHEQLKKQKFDQVPQLSSSRWIDVNQPMFIVPPKSGVRRAMLIGINYVGQKG
QLKACHNDVENVKDYLIKAQGFRESEMLILKDDGRHEMPTKQNIMDGFVRLTQYSQPGDVVFVSFSGHGGRVVDTSG
DEDDGFDESLIPVDFQEVGQIVDDDILDIFVKKMKGGVTCTVVMDCCHSGSVMDLPYYFSADGKEMETEKNFQFGSG
GTDGEVPKKHKRTFDKHGKAVKVHEKEEQDKAEKKAPKRTVHEAPIKKIKPRAPPRDRKPPPPPPPPQR

>Acoffeaeformis1 CAMPEP_0186537396
MSVEEQAEEAIPAVVRMFSGCEDRQTSADVSNVGSFQLPDPAGRAGGALTSALLNTTYADHANTGADLTFKETLITA
REKLREMGFEQVPQLSCSRATDLDQPFSIIPPDFEGTRRAVMIGINYVGHDPGELSGCHNDVFNMAEYIKDCHGFTD
DDIVYLLDDGEHTPPTAANIIEAFQTLASQAQPGDACFLHYSGHGCSIPDQDGDEDDGKDEALCPVDYAENGILRDD
HVLQMLVAPMPRGVTLTCIMDCCHSGTILDLPFLFLADGSQEEMEADPDFEFGPFLTMVSSFAAAGLEGLKQLHAEG
KARRKKRRQWLKSRLGF

>Acoffeaeformis2 CAMPEP_0186537718
MAEDLIRAEVRMISGCRDSQTSADVSNVAEFQLPDPAGRAGGACTSAMLQVLYADHQTPEDDLSMQEVLLQMRDILR
SSGYTQIPQLSSSRCLDIHTPFRLVPEDFSGTRRAVVIAINYVGQQGELRGCQNDAHNMIEYIKNVHGFEDENILIL
MDDGIHPDPTYENIITAYRQLVASCEPGDAVFCHYSGHGGKLVDEDGDEEDGYDETLVPVDYQSAGQIRDDTLYKEL
VGALPEGVMMTSVMDCCHSGTVLDLPYVFVADGEEEEMHENPGFDMNAMAGFASAAGIAMALASGADPIEVAMQACC
AIL

>Acoffeaeformis3 CAMPEP_0186529544
MGRFLKKLAKLKDKVEDRIADFREEEPAARAAFEDAKPEAMKNDIRMISGCEDAQTSADVSNVNSFSLPDPAGRAGG
ACTSTLLNILYADEKVPEDDLSYTQVLEKMRANLKKKGFSQIPQLTASNPIDVNTDFDLVPPAATGTRRAVMIGINY
VGHDPGELRGCHNDVLNMKKYIMAVHGFAEENIQVLMDDGEHTPPTKENILAAYKKVIAETQSGDAVFLHYSGHGTK
LRDDDWKEEEDGYDEALVPVDYQEGAGMIRDDDLYEILVKPLAHGAHMVSLMDCCHSGTILDLPYIFKADGTDQEMH
LDPTLNLDGLIQSFGGHVTNLMSKFKFFK

>Acoffeaeformis4 CAMPEP_0186534122
MSSFNEKVINTIPSQVFMISSSSDKQTSDEAYNAGAFNLPNPNGKQGGACISSLLQVLYERGHEKGSMTWISCLEDM
WQQLKNLGFEQVPQLSSSRWIDVYRPMYIVPPNSGKRRAMIIGINYVGQQGQLKACHNDADNIKDYLINAQGFREEE

MLVLKDDGKHMMPTKQNIMDGFTRLTQYSQPGDVVFVSFSGHGGRVVDTSGDEEDGYDESIIPVDFRENGQIIDDDL
LDLFVKRLKGGVFCVVVMDCCHSGTVMDLPYAFSANRKKMAIERNFPFDDDDKTRKRAFKNQKHAERRGDASMAGDD
KTVSTTRTKPKKKQRPKPSIREMPIEPVPPRPPRRSVPPPPPPPKQCCTIM

>Acoffeaeformis5 CAMPEP_018654023
MSLEQAQQAIPAVVRMFSGCEDKQTSADVSNVASFKLPDPAGRAGGALTSALLNVTYADHKDTGKDLTFKETLYAVR
TSLQGKGFEQTPQLSSSRPTDLEEHFAILPKNFAGKRRAVMIGINYTGDNPGELRGCHNDVHNMKEYIKNCHGFTDA
DITLLLDDGKNTAPTAANILAAFKKLASEAKAGDACFVHYSGHGCSIRDDDGDEADGMDEALCPVDYKKSGVLRDDD
VLEKLIAPLPRGVTLTCIMDCCHSGTILDLPYLFLADGMQEQMQPDPDFDFGPLITMVSSFAKLGFEGLKKLHKQGK
VRRKKERQWLKNRLGL

>Acoffeaeformis6 CAMPEP_0186520996
MSSIDERIKQAIPAEFHMISGSADSQTSADVQNTGKFQLPDPAXXXXXXXXXXXXXXXXXXXXXXXXXXXXXXXXXX
XXXXXXXXXXXXXXRAGGACTSALLQVWYRDGHATGEKTWVQCLREMRSNLLQMGFDQVPQLTSSRMIDVNKIMHIV
PPGSTGRRRAVLIGINYVGQQGQLSGCHNDVNNIKKYLIAAQGFKESEMLILMDDGRHHAPTRKNIEDAFRRITEYS
NAGDVVFVHYSGHGGRVRDTSGDEEDGYDETLIPVDFRSAGQIVDDNILDMLVKPMRAGVTVTVLMDCCHSGTVLDL
PYRFSADDSQMRLDQGMNMDNFLGKLDPGSVALCACLGFMMMNMLDF

>Acoffeaeformis7 CAMPEP_0186541908
MGGDFEGKAHELIPADVRMISGCQDAQTSADVGNVATFSLPDPAGRAGGACTSALLKVLYQQDGTESFSFVDVLMRM
RTVLRSGSYPQIPQLSCSRKMDVQEKFTLVPESSSTPYGSKRALLIGINYVGQQGELRGCHNDVGNIKRYIMDVHGF
QEEDIVVLMDDGNHELPTYDNIIAAFRRLVNDTESGDCAFFHYSGHGGRLPDDNGDEEDGYDETLIPVDYQSAGQIR
DDLLYSDLVGRMPEGSTLTCLMDCCHSGSVLDLPYTFQADGEQQEMGENPKANMGRLQAMAVGFLVRKIFGTGPAAQ
MVMSLATSGLAMAQSGGSSGGQKTGGANNVLLPLLX

>Aglacialis1 CAMPEP_0199875840
MGFLSRMKKRIEERFDEKQGAVIPADVRMISGCQDSQTSADVWNVQSFGLPDPAGRSGGACTSALLKVLYADHRKLD
DDLTFVEVLDKMREILSEGEYTQIPQLSSSRAMDLELPFHLVSQQEDATDKRRALLIGINYVGHESGVLSGCHNDAL
NMKEYIMDVHGFEEENITLLLDDGEHDPPTKENILNAYKKLVEESEKGDSVFCHYSGHGGSTRDDDRGEEEDGKDET
LIPVDYKTAGIIRDDDLFDTLVHPMKEGVTLTCLMDCCHSGTILDLPYTFKADGSGNVDMSIDDDFNFDKLFKKIGE
VFD

>Aglacialis2 CAMPEP_0199894872
MPSIEEAIPANVRMISGCRDEQTSADVSNVATFSLPDPAGRAGGACTSGLLKILYADKHKPDDDLSFQEVLLNLRDV
MSSGRFTQIPQLTSSRPLDVHTPFTLVPDECTGTKRALLIGINYIGQQGELSGCHNDVLNMVEYLKDVWGFEEHNIT
ILLDDGEHTNPTRSNILSAYQQIVAEAESGDAVYCHYSGHGGRLRDDDGDEEDGYDETLVPLDYQRAGQIRDDDLFN
ILVGPMKAGVVLTCVMDCCHSGTVLDLPYCFVADGEQEEMTLKPDFDMEKLMGLAQALMAASNGDPMALAQACCTIL

>Aglacialis3 CAMPEP_0199871010
MVDFGAFDSQAIEAIPADVHMISGSHDKQTSADVSNVGAFQLPDPAGKPGGACTSALLKVLYKDKHAAAPMSWVDTL
QRMRSILRGMGYDQIPQLTSSRMIDVKSTMYIVPPGSTGRRRAVLVGINYIGQQGQLSGCHNDVHNIKDYLINVQGF
KEQDMLILMDDGRSHEPTRENILKSLKRVTEYSEPGDVVFFHYSGHGGQVRDTSGDEEDGYDETLIPVDFKRSGQII
DDLLSDVLVKSLKKGVTLTALMDCCHSGTVLDLPYRFDADGSDAMIRDEKMDFGKFLDSASEVVGCLLCLYCILGLV
SDF

>Caffinis1 CAMPEP_0187013496

MPPQLEKVKREIRFQDQDTTIPPGGEYDLATADSPSENLLFDEIVEKVYPAEILMIGGCRDSQISVEVDAPGASLAS
MLSSGGETHHSKEVSINLSQLRNTHFRLPNNIDGRSSGVCTKAFLSCLQGNHATVSWFQLLKSMRAQIHLKGYNQDP
QLSSSRLIEVDQPFEIVRNKNGTKRAVMIGVNYTGQKGELSGCHDDVESITSYLIESHGFEKENMTVLMDDGIHEPP
TYRNIMVACRAIVRESKPGDTVFFHYSGHGGRIQDINKDEADGFDETIIPSDYFRAGQITDDDLNKTLVKPMKKGVY
VVALMDCCHSGTVLDLPYVCTEGSHMTRQTQLNLDLKKTKFCGFFYSCIRAN

>Caffinis2 CAMPEP_0187019438
MTSEQAQNAIRSEFRMISGCEDKQTSADVSNVGSFQLPDPAGRAGGACTSTLLNVLYRDKVRPDDKSFKELLLEMRE
ILQSKDFTQIPQLSSSRNLDVDTKFDITPDGFHGTKRAVMIGINYVGQEGELAGCHNDVLNMKEYLMDIHEFEEENM
TILMDDGVHPDPTRDNIMYAYRKIVAESQAGDVVYLHYSGHGGKLRDDGNDEEDGYDETLIPLDYQSAGQIRDDDLL
RTLVIPMQEGVFVTSVMDCCHSGTVLDLPYNYQGEGDSMQEQDFKYDAAFSILGALATGNTSAAASVAMDECCTVS

>Caffinis3 CAMNT_0029934239_2
HKSTLLIFHNSHKRLTSASNLYFIYNKKRKNIKTKMSYPGNRRPQNRPAQPQKQNSVGFGNQPTQFDEMAIKAFPAE
FQMISGCHDSQTSADVSNLNSQFSLPNPQGKAGGACTAALLSVLYDSHRNGTFSSMSWVTLLRSMRQNLLNKGFDQV
PQLTSSRLIDVNKPLKLVNDDANASYGTRRAVMIGINYTGQQGQLSGCHNDVKNMIEYLKSYQGFEDRNMTVLMDDG
YHHDPTYYNIMDAYRTVVRESRPGDTVFLHYSGHGGRVRDTSGDEDDGFDETLIPVDYQRSGQIVDDDLCTQLVKAM
PQGVLVTSLMDCCHSGTVLDLPYRFTADGDAMVRQNEFDFGDIAVVALAACCLFDLLCCLCSALGGMVDGFDM

>Ccurvisetus1 CAMPEP_0187040390
MDAEQQIEFDTKAKEQIPASIRMISGCRDSQTAADVTDLGSFSLPSPDGRSGGASTAVFLKTLYDAKNDGTLSSLNW
VDLLQAMKEKLDGKGYSQVPQLSSSSMMNIKEPVKFVNDDPNGTKRAVLVGINYVGLPGQLSACHYDVKKVKEYLIT
ELGFEEKNMIILMDDDHHTRPTKYAILTAYRRLVEESKAGDTVYCHFSGHGSRVVDTNGDEDDGYDETLIPVDFYRA
GQIVDDDLLKLFVKPMAKGVLMTCMFDCCHSGTVLDLPYYFNADDDEKKMQRLSDFKFDKKKYLWCRLSVAFRFVYS
PVSGSSYVVNSLEKHVMKIFVSDTALPGILYL

>Ccurvisetus2 CAMPEP_0187047062
SADVSNVGSFNLPDPAGRAGGACTSALLNVLYADKKKPDDDLSFKQVLLSMRDMLGSKNFTQIPQLSSSRELDVDTK
FDITPDGFSGTKRAVLIGINYVGQQGELAGCHNDVLNMKEYLMDVHEFEEDNMHVLLDDGVHEDPTYDNIMNAYRQL
VSSSVEGDCVYIHYSGHGGKVRDRDGDEADGYDETLIPLDYMNAGQIKDDDLLRTLVIPMQRGVFVTSVMDCCHSGT
VLDLPYVFKADGEHDQMEHDEDYDFDPMMSILGDAGMIGTGLAVAAAVADSECCTIS

>Ccurvisetus4 CAMPEP_0187051206
LITTKGKMNDFDQKAEQAIPADVHMISGCHDSQTSADVSNVGNFQLPNPQGRAGGACTAALLQTLYDAHRNGSLPTL
SWVTLLRSMRTNLLNKGFDQVPQLTSSRMIDVEKPVQFVNDSNNYGTKRAVLIGINYVGQNGELSGCHNDVKNMKEY
LMNVHGFPESNMTVLMDDGYHLNPTRYNITNAYQNLVRQSMPGDSVFCHYSGHGGRVRDTSGDEDDGFDETLIPVDF
QSSGQIIDDDLFKNLVQPLPKGVLMTCLMDCCHSGTVLDLPYRFTADGDVSAGMQRIDGFDFDELLMIGLICCCLPD
LLYCFGDLFGCLYGLLDF

>Cdebilis1 CAMPEP_0200872066
MIYQQRYLSILRHRIRRPSLKEAFVATAIFSCLLLLLVPFLHKASDDNILETQEKGAPTQTVNYVSSLVVGYPSTSP
PYNSTVEFIISPKDPSCIEDPGHLFYYPFAEEADDPDPTIYNCTWLANTRRPNLICDYEIFNTGFENLQPAREVCPV
TCDSCYLLHPSAVPTPTPSFSSHPSSEPSVSARPTLLPSTEPSGSPTSIPTQRPTACPEDPDAIFFFRTELEDATKP
IFHTCKELSEFKYPENTCKFLTQSWTEEIEEDQKPISINYYYPAKDICRNTCSTCVIESPNPSNGPSISPSRTPSKF
PTQNPSEVPTIPGIPSPRRTPNPSITPSKNPSLRPSNSPTGTPKIVEFDLTMTFVKDPNDSAPNEDFNEEPLTEVVT
ETLMSSFENQKDGNFEVVSRVKSQESFGERRYLRLQSSGSQSQTILSRRSGSNRSRQLQVSGQRVIFLIVVENNSGR
SLKSIKNASENTFESEESQRKFLKELRNRDSSFVIYNGVVVSTDEVKPSLTPTPRVATVKKRKAWIIVLSVVLGVSI

LLVLILLGLLYLFKRKKDEVKKIEGNDKIDTVMPPPPSITDLSIPPDPPKFPPTNSNSDNENAFLISNDSPKQEHNT
ETSDNGEIEQANSSTTEATGDGESALDSDSGMQPNADSIQPSEPIGPSVSDTSNAQKDRDKNVGMRERGYNSYSEYS
SQDDYSSDDSYEDSLSGSDFRYGDNSEESVTRSDESLVSLKCTVHMISGCRDTQTSADVDEVGKFCLPNPLGKSGGA
CTAALLQTLYDFHGSEKTRSLTCSWAGILLSMRENLRSQGYSQVPQLTSSKPINVNDTFHISNPWYKYGTKRAVLIG
INYVGQEGELSGCHNDVIRIKDYLINFQGFEERHITLLMDDGININPTKGRIIRAYRRIVKVSKAGDTVFCHYSGHG
GRTKDLDGDESDGLDETLIPMDYQSAGQIIDDDLFKELVQPMSEGVHATCLMDCCHSGTVLDLPYHFTAESDASKGI
QRGRAPHSSYREIRQNTQKKPEIIPLPAKNMNKPRVGKVKPSPGTGHEFDDVFRIDHACISPELHLPPMISEGKIAI
TVPSGPLGFATVDNPTNGIRASEVFAMKQSSILAGKIRNGDRLIALDGIDTSSLTAAEVTKLIISRSSKSRDMVFHR
SDRGKPTDMRTIDEGSVSSHGINDYTCKTGTSELTKLIDGVAEHEAVSVISVASEDNESVCSMISC

>Cdebilis2 CAMPEP_0200886990
MSDFDQKAEQAIPADIRMISGCQDSQTSADVSNVGNFQLPNPQGKSGGACTAALLQTLYKVHREGAEISWVDLLRAM
RDNLLEKGFDQIPQLTSSRMIKVESPLKIVNDTDVYGTGTKRAVLIGINYTGQQGELSGCHNDVKNMKEYLITVHGF
EEQNMLILMDDNYHPNPTRMNVLNAYRNLVRESKPGDTAFCHYSGHGGSVKDYSGDEEDGFDETLIPVDFQRSGQII
DDDLFKELVRPMSTGVLMTCLMDCCHSGTVLDLPYRFTADGDVSQGMQRISGFNFDDLLVVGFVCCCLPDLLGCLFE
ILG

>Cdebilis3 CAMPEP_0200879288
MFSEKAEGAIPAEFRMISGCQDRQTSADVSNVASFRLPDPAGHAGGACTSAILNVLYADKHKPDDDLTFKDVLMKMR
DMLDTKGFAQIPQLSASRDIDVDTKFDITPDDFSGTKRAVLIGINYVGQDGELAGCHNDVLNMKEYLMDVHEFEEDN
IHVLLDDGVHEDPTSENIIAAYRRIVAESVDGDCVYCHYSGHGGKVRDDNGDEADGYDETLVPLDFQEAGQIRDDDL
LKTLVIPMRKGVFVTSVMDCCHSGTVLDLPYTFKADGEQESMQHDESFNFDPMMGILANAALGGAAAVVVAANECCT
IS

>Cdebilis4 CAMNT_0047068781_6
KQQFFHIIYSISSSSIMSEFDKKAEENIPAEIRMISGCKDEQTSADVGNVTSFGLPDPAGRAGGACTSAILKVLYAD
KKRPDKDLSFKDVLLKMRDVLDDKRFEQIPQLTSSRNLDVDTKFDITPDDFNGTKRAVLIGINYVGQNGELAGCQND
ALNMKEYLKDVHDFKDENIQLLLDDGTNTNPTKRNILKAYAKIAEESDAGDVVYLHYAGHGGHKSDRGGDEKDGRDE
TLIPVDFQKWGVGEILDDDIYQKLVLKLKKGVFATSVMDCCHSGTVLDLPYKFVGDGKQEGGMNANEGFNFGTAKGG
CIIL

>Cneogracile1 CAMPEP_0200990868
MISGCEDKQTSADVSNVASFKLPDPAGRAGGACTSAILNVLYADKKKPDGDLSFKDVLLQMRGMLDGKGFDQIPQLS
ASRNLDVDSKFDITPDNFSGTKRAVMIGINYVGQDGELAGCHNDVLNMKEYLMDVHEFEEDNMMILMDDGEHVEPNQ
ANILSAYRRVVALSQPGDVVYLHYSGHGGKLRDDNNDEEDGYDETLIPLDYNSAGQIRDDDLLRILVLPMQAGVYVT
SVMDCCHSGTVLDLPYNFKGDGEQESMNESENFDFSTPMGILSTLTSNASLMNSVASAAEECCTIS

>Cneogracile2 CAMPEP_0200992636
MSNYPGNRRPQAEKQQSIQYSSNATGYDRQAEETFPAEFHMISGCHDSQTSADVSNLNTQFSLPNPQGRAGGACTAS
LLSVLYEEHRKGSIDSLSWVNLLRNMRQDLLNKGFDQVPQLTSSRLIDVNKPLELINKSSDAAYGTKRAVMIGINYR
GQQGELSGCHNDVKNMVEYLKNKQGFQESNMTILMDDGYHTNPTYSNIMNAYRTLVRTSQKGDTVFIHYSGHGGRVR
DLNGDEDDGFDETLIPVDFKRAGQIVDDDLCKELVKAMPRGVLVTSLMDCCHSGTVLDLPYRFTADGEAMVRQDQFD
FGDVIGGVVAMCCCFEIMLND

>Cneogracile3 CAMNT_0047211831_3
IMDQSTDNNDEHANDTSHNHESNNAMFHLISACDDSQTAADVSDINSQFSLPNSQGKSGGACTAALLNALYAAHENG
TIDSMSWVGLLKEMRKDLIKKEYDQIPQLTSSHRIEVDKPFKIVNDSPDSMSGTKRAIMIGINYVGQQGELSGCHTD

VKNMRKYLVEVYGFKKSDIIVLMDDGIHHNPTKHNIMKAYRKIVRDSLPGDTVFLHFSGHGGRQVDLNGDEDDGYDE
TLIPVDFQEAGQIVDDDLWVELVKAMPQRVLVTSLMDCCHSGTVLDLPYQYAADEDNVVSSGDCSDNSTGSLQDDQS
PETPETAAASSTVSEDARLYELITSKNWKRAEEFLDDKSTFNEEEKLENLQYQDSNGYTPLVKAVLKKSAPVSLIKT
MINLGKRETLEVTNDSDWNVLHWAAYSNNVSLEVFKLILEKSNRDVIYAKNNYDKTPLEILLNRNKAAFIDKTLALL
VKEQLLSDQKTTVDRLCTSWSTIKTLAWAHHLPESEKDIVLERPLFKAILNSTFICPRYIFISMMDLYMQIALVLVF
SFFLRNAIIQSDTSPFLSLIILVVCFVWFLIREMTELMLTYLKNYFLEPTNVLDIIQFVLLAGSIVIIFASGIDDDL
NPLSTIDHAVFISATCVAWLKLLFVVGNLYYNGAVFANGVITIIGKIVPFLFTSAVLLLMFSHMYLMWGAGHYYFCS
QFFKSDDEWTCNLPSSYHETFSMFLGLNSEVQVSTFAISYVYGFFISILMMNILIAIICYSYAKVLESGDRTFWRNR
LKFIAECQCFFNMFCCAERHNRIRNESTRYGRIDSEIITNFDLNVDQDGDPQQDNGPQVHILKNDRISFGTHDWSIY
NKKIGANDKREFFGWWFETSVTSTPSLRIRLKYFFSRALWEDIICPGQEFENILLGIQYNKSGRGLRLFFVRLVCYV
LIPFSLLIIIILMFCFGSVSFGLLWHRKMREHLFAEDTNKFKSEQVEARDTLKEEIFIELQEYETNINDSLEQLVRKH
EEKIGKQFAILSKKQKMNFKKQNEHEYRFDVIDEHLSDIKKMLSEMGNK

>Cpennatum1 CAMPEP_0200351432
MSFQEQAEKLIAAEVRMISGCADSQTSADVSNVKEFELPDPAGKAGGACTSALLNILYNDGVAAGEMSWVDMLRRMR
VELQGDYTQVPQLSSSRQFDLSNTFEFAKTGYGGTKRAVLIGINYVGQSGQLSGCHNDVLNIQKYIMDVHGFQEENI
EVLMDDGYHTSPTRHNITVAYQRIAEVSQSGDAVFCHYSGHGGKLPDDNGDEDDGYDETLIPVDYEHAGQMRDDELL
QRLVLPLRAGVTMFSLMDCCHSGTILDLPYRFRADGDTEEFQEESGYNFGGYMGLLGESAETVFCCFMLLKFVWDMV
M

>Cpennatum2 CAMPEP_0200351262
MPFSFNGAFQNLKDRFDDDDEDPSYEERATNMIPATVTMLSGCKDCQTSADVYDVKSFGLPDPAGKAGGACTSALLK
LLYHEEQVPDDDLTYVEVLNGMRDNLDNDYTQVPQLSSSRPFDLETPFVFAEGIGTRRALLIGINYGGQNGELSGCH
NDVINIKGYIEKVHGFSEDNMTILMDDAEHEEPTRKNILKGFAELVKSCEAGDSVFVHYSGHGGSIKDDDSGEEEDG
MDECLCPVDYALNGVIRDDDVYSTLVAGMPGGARVTCLMDCCHSGSILDLPYLYTAGSEECCLKENEKFKFGKLFQL
LGNLAEDFLDLF

>Cpennatum4 CAMPEP_0200356122
XTQKPDAMASTGNFHQQAVELIPATVRMISGCHSEQTSADVSNVASVAQLPSPAGRAGGACTSALLDILHRTPPGSL
SFQDLLGQLRQNLSNSGFDQIPQLTSSRSLDVGRTPFHLVGGSGQRRAVLVGINYRGQSGELSGCHNDVFNMKKYIM
DVHGFQERNILVLVDDTRHPIPTRQKIIAALEALVANSVAGDNVYFHYSGHGGLLEPEANLFKLGGGDTYDETLYPL
DHTRAGQIRDYSLFNRFVKPMAAGVTVTCVMDCCHSGSVLDLPYSFQPTPGGGIRSVQNFDALSNLAFLYVLAGGLL
PSAGFGDIAANIGDQVGGDISDYQGAGIEAEAQDDEAYEGDGPDFGDFADGDGAPDDFGPGGVPDDPGGYEMGGGDR
GFGGGDAGEGYDTGDGDRGFGGFGMRTRDVDGDDEGPGFGGFDMAGGDGGFGMAGGDMGGDDDGPGVDCDCLIDSIN
SLLETTGEEE

>Cpennatum5 CAMPEP_0200357564
MTFQEQAEKLIAAEVRMISGCADCQTSADVSNVKEFELPDPAGKAGGACTSALLNILYNDGVAAAEMSWVDMLRRMR
VELQGDYTQVPQLSSSRQFDLSNTFEFAKTGYGGTKRAVLIGINYVGQSGQLSGCHNDVKNIKRYIMDVHGFQEENI
EVLMDDGHHTSPDRRNIMEAYARIAEESQPGDAVFCHYSGHGGKLPDDNGDEDDGYDETLIPVDYEHAGQIRDDELL
KKLVLPLQAGVTMFSLMDCCHSGTILDLPYRFRADGDTEEFQEEGGYNFGSYMDLLGDSGATVFCCFMILQWVMSML
W

>Cpennatum7 CAMNT_0046377579_4
KSEGYSQASKKREVINSRYPCVSTLLLIPSNRSFSLTIQPINIPL*PRQIFNMPISFNDQFRKLKNRFDDDDEDPSL
EERATVMIPANVIMISGCKDAQTSMDVFDVGDFGIPNPAGKAGGACTSALLKLLYNDEVVPDDDLTYVEVLNGIRDN
LDDDYTQVPQLSSSRPFNLEKPFVLAEGEGTRRALLIGINYVGMDGELDGCHNDVLNMKSYIEKVHGFKNSNISILM

DDGKNRHANPTKKRILRNFKKLVRECEEGDSVFVHYSGHGVSLRDKDRNEKDGMDECLCPLDYTAKGYIRDDDIYST
LVAAMPAGVRMTCIMDCCHSGSILDLPYAYKAKTRKNGSEQSKKLKTGYAKSSVFEENNKFKFDKLFKCLGNLTEDF
LDASDDSDVSD

>Cpennatum8 CAMNT_0046377197_3
LPTQKSDTMASTDNFHQQAVDLIPATVRMISGCHSEQTSADVSNVASVAHLPSPAGRAGGACTSALLDILHRAPPGG
LSFQDLLGRLRQHLAGAGFEQVPQLTSSRSLDVGRTPFHLVGGGGQRRAVLVGINYRGQSGELSGCHNDVFNMKKYI
MDVHGFHERNIIVLVDDTSHPVPTRHRIIAALEALVSNSVAGDNVYFHYSGHGGLLAPEANLFKLGGGGDDGYDETL
YPLDHTRAGQIRDYSLFNRFVRPMAAGVTVTCVMDCCHSGSVLDLPYSFQPTPGGGIRSVRNFDSLSNLAFLYVLAG
GLLPPSGFGDVAAHIGDRVGGDMSDYTGAGIAAEAQDDEAYEGGDPDVGDFADGDGAPDDFGPGGVPEDPGGYEMGG
GDRGFGGAETGGGEAGEGYGMGEGDPGFGGFGMGTRDVEGDDGGPGFGGFGMAGGEMGGDDGGFGMADLMGGDDNDP
GVDCDCLIDSINTLLETTGDEE

>Dbrightwellii4-1 CAMPEP_0193954124
MGWLVKKIREFKEEQKAHLIKAEVRMISGCDDSQTSADVSNVKMFELPDPAGKAGGACTSAYLSVLYKDKKASGAEL
TFVEVLQKNEGSSPEKKYTQIPQLSSSKPIDLNEPFQLVPDTATGTKRALLIGINYTGMQPGELTGCHNDVKNVKEY
LMNEHSFKEENITVMLDDNVHESPTKANIISGYKKLTKESQPGDAVFCHFSGHGSQIKDDDWFEEEDGLDETLVPVD
FKENGQIRDDDIFDILVVPMKEGVTLTCFFDCCHSGTVMDLPFTYRADSDSSQMVLDKKLDFGKVFREVGDFFNKKE
TNAK

>Dbrightwellii4-2 CAMPEP_0193979726
XQAIPAEVRMISGCADAQTSADVSNVNQFELPDPAGRSGGACTSALLKVLYTNKKKPDTDLSFRDVLQRVRDILAEG
KYAQIPQLSSSRPFDVKTHFALVPKGKEHGTRRAVLIGINYTGMNPGALTGCHNDVHNIKHYIMDVWGFKEEHITVL
LDDGKHKPPTRANILGAYAALAAKCQPGDVAFCHYSGHGGMLPDDNGDEDDGFDETLIPLDFKRTGQIRDDDLFKIL
VVPMKAGVTLTCLFDCCHSGTVLDLPYNFQADDHEFTEEKNYKFTPLLSIAAELKANCINCTNPADPAANCTVS

>Dbrightwellii4-3 CAMPEP_0193978290
XFPLKDKNIHFLSIGTCKMGNDNSREAESTPEVAEFRMISGCQDSQTSADVSNVNEFELPDPAGRAGGACTSALLKV
LYHDEKTPEDTYSFVEVLKMMRDELSEGEYTQVPQLTSSTIIDLNEPFDLVPEKSTGTRRAVLVGINYVGMNGELSG
CHNDVYNIKKYIMDVHGFEEENIVMLVDDGECEMPTRENMMNAYKWLVESSEAGDAVFTHFSGHGGQLRDQDGDEAD
GFDETLIPVDYLENGQIRDDDLYDTLVKPMKVGVVVTSLMDCCHSGTVLDLPYTFRADSDKFEMEMEEGFDLGKLLG
KIGA

>Dbrightwellii4-4 CAMPEP_0193955922
MADFDTKAKAAIRAEVRMISGCHDAQTSADVSNVGSFQLPDPAGRAGGACTSALLNVLYRDHQASEDLSWVDTLRQM
REILSSKDFEQIPQLTSSRYIDVNETMQIVKGHGAKRALLIGINYVGQNGELSGCHNDVLNIKEYLMDVLGFEEDNI
MVLMDDGIHHEPTRDSILSGYRRLVAESVAGDTVFCHYSGHGGRLVDDDGDEDDGYDETLIPVDFEHAGQIRDDVLF
NEFVRPMAEGVTVTCLMDCCHSGTVLDLPYRFVADGDDVQMRADESVDFEGFLGPAFWTAGIIAASSLGGGGGGVGE
IFGAIMIAAGVYVGLSWIPVSVYVGVYVTV

>Dbrightwellii4-5 CAMPEP_0193981564
LTFQDLLLQLRSSLSNSGFDQIPQLTSSRPLDVQQCPFHLVGGTGARRALLVGINYLGQNGELSGCFNDVYNIKKYL
QNHHGFAPKDIVVLMDDGKHSFPTRQRIIQELKKLVSCSIGGDSVFFHYSGHGGLLDADGFNLFKAANPNAKQYDET
LYPVDHGTSGQIRDFSLYSHFVQPMKEGVFVTCLMDCCHSGSVLDLPYSFQPTSGGGIRTGRNLDTLSNLAFLYVLA
GGMLPSHGFENVTNHIENNVDGTLDDYQGTCVEQLEMDDVACEEAQFDDPEGVGYGEGIDDDDVEYGHDYDDFGGDD
DTEERFVGNGDGDDVGEYMNDPDNPVMGSPGFDGGPPPPFEDDAMDGPLSRPPMEYDQAPPDIVQGTFVDDGDRGGE
FVGDDFAEDEAVNYFPPQEEVQGGFGEGYDDAGGDDAEIDCGCLGDLMSALMEDEE

>Dbrightwellii4-6 CAMNT_0038558635_2
ITPSITSSMFSYPGQKKRRPKPRPTGPPPPLSDLSRWFDYYDTDRGGSLDREEVSAALLHTPPFNHRGADSVRETVY
SVWAAFDHDGSGSIDKREFCAPGGLGESLLAYTTQHHQPPPPKPAHTYPPQTTHTRPPQTYQPYQPATNTTQSNDPD
IYVPAYGSNNQTHQETHQHNSPAPSAPPVYSSGSSNTWNCQNCTFANSNTDPRCQMCNAAAPAGIAAHNHHQPAPAP
YHQPAPAPYHQPAPAPYQQPNHHHQQPPSYQQPTHHQQPTRHQHQTQQSPQYQTMRINIPAGTVPGQRLKIKTPGGH
EHMVTIPERHQWHSQHPPVFDVQVPVAPAVVVQAAPIHNPTPSGGHYAPAPPPKPAQTPWQSWESFRGHTFQQVPMA
TTNVPPHEISHHSSNIPASGRRKALLIGINYKGTRAALRGCVNDAKNIKNLLMENGFRDDPTHMVMLVDEGARNSNY
LPTRANIMRGFQWLVQGVSEGDALFFHFSGHGSQVPDKSGFEEDGLNETILPVDYQKGQISDDVIWSSIVYPLPSGV
KLTVIMDCCHSGTGLDLPFDYMLDSKRWVEDTNPAHSKGHVVSFSGCEDSQTSADTIEKYQAGGAMTQSFISAYRQN
PMSTYPEFMAAIHRRLKQRGFKQRPQLTSSQAFDVKNTVFSFTEGFQPNKNQVIGRIQRKKIRPGSTGDRRQSGVND
LLFGGLMVGGALAFADALFG

>Dbrightwellii4-7 CAMNT_0038593613_3
ALGLRNNSKRISHIKANILLDSLTKHPPTKPTHTTSLPQAHQSAYSHQGVNVSTGSAPQLQTSHQSEHNHQHHTMMM
SVPTHTMRPPHTYHSTHSHQGVNVSTGSAQQLQTYQSHQSEHNHQHHTMMMSVPTHTMRPPQTYHSTYSHQGVNVSS
GSAPRIQNYQSHQHQQIRPYHTVAMSTKDYPGSQVHTVTTPKIDEAHSQNAAGDVVEVHAAPSVATQNHQQQPTFNQ
HPAHQKHQSHQNHQYHAMTMSAPQASAPELPPKDPTVSQDHKVTTPKIDEAQSQNNTSVDVEVPAAPSVATQNHQPQ
STSNQQPSHQKHQSQQNHHHAMAMSAPQASAPGQTPKIDIPERPDHIAAAFERIKLQNAATYDENPNTEGQGSLVPD
RSDEHSTVAPPPVLLRPNQTPWQSWESFRGYPFKEVPLATTDIPSNEVYNDASNVIASGRRKALLIGINYAESPAPL
QGCVNDAKNIKNLLIENGFTDDPTHMVMLTDEEQNSKYLPTKANIRRGFQWLTQGVSEGDVLFLHFSGHGSQVSDEA
GMEEDGLNETILPMDYQKGQITDDMIWSSVVYPLPSGVKLTVVMDCCHSGTGLDLPFDYEPYTGRWVEETNPAHSKG
HVVLFASCEDAETSADAIGKYQAGGAMTQSFILAYKENPMPKYPEFMAAIHGHLIDRHFKQRPQLTSSQELDTKNSR
FSLIEGIQRNKNRTIGRIQRKKIRPGSTGDRRNFLFR

>Espinifer1 CAMPEP_0200425734
PTATTLAPPLTDPYSWFDYYDQDNSGTLDQDEVVSGLLNAFYQKQSSTADEQHGGTNQSGNDAAPPSVDTIRESIKA
IWPAFDIDGDGQIDREEFCSPGGMAESLLAATTGGIPPGQAETTDRGFFSSHHHASGGGGYPGARPHHSSGGGGYPA
ARPQQQHQAYVPTAKPVASSGMMAGVYNPHAHQQQQQQPAPTAPPPPRPAAATVQVVQPVTYKTIRVQIPPGMGPGQ
KLKVLTDAAGKEAAIVTIPDRAKWRANARTGQPFFDIRVAEKPKPAPTPVVVQGHSVGSPGRHSAGASVSSVTSSMA
AMSTGAAAGPAAVTFKAWERFGGHSYHAPSLGMRRVPHLPITSMSNVHASGRRRALLIGINYKGTRAALRGCINDAN
NMERLLVQHGFPRDSCHMVKLVDDSRNRNYLPTRANITKACQWLLQGVSKGDVLFFHYSGHGAQVPDRSGMESDGYN
ETILPMDYRSGQITDDALWNSLIAPLPDGTRLTSVMDCCHSGTGLDLPYDVDIRTGRWKEDVNPAHAQGDAILFSGC
EDAQTSADTMDKFQAGGAMTQSFIKAYQDNPMATYPEFMASIHRHLKRRGFHQRPQLTASQKFDVRSRIFSFTDGIE
PNHNPQIGRMKRRHVRPAKAGYKKNNMNDLLFSAGGAIALAGLANLFLDG

>Espinifer2 CAMPEP_0200438730
XLFAAMKQERKSISLVTAAAVSLALNIALARATTLTDTSWRSDLGVDSQTWSLAMRASGRLALLDLHDLYVKEPSKL
QSVRLPPFRGGGAGEDGDGSERNKDDDVLYNPRLSLSTAWSASSAQLPAPSQSATKAAIRECAESSQSVSPANDLQT
TPGGASAGSCGQDMQQVCAGSRRTNTVSTTTRASGQARALVVVWASWNILNGALLTLTPKDSYDFAAYLVEAIGAVR
VSHGLQLFLAAGANISAQKVMGVGILVRLVFLAASFALGTYETLQTQSKAPFLLSAGVMSMAAFSLLSNKGRPMLMA
NLFSTTTCIKGMNMIFRPFASNRLFGMGFTDGELYWILHSSEKSSHFSSLGQSDCDFFFXIPLLNVIGTKIPGKYRS
LGFHLVISSVFMNALSHGMEPIRAAGLSAATWLALLAELTFVSGGYRRLSDVPRVYLFFLASALVCSIVFLTDTPRK
HVDSSSVGDALSVSGWRSDEEGASQEILWKQEDVYPLMYERDDNNTSDVVRSIRIIXLAYSTSLTGTILLELSLRDG
RKPWCCECGKSGAALGRSAPMIDSTDSIDSTDSIDSHHDDGRQEDKTRHQQRSGALLLLCAAAAAARSAAAAAAAAA
ARGESYMYMSCRELRRSRGKILFASYFPQRRSGRGPKIRAFHLIYGFAKLPSLIVLRSRLIRVLPCRVAEGGCCGHQ
TLTEVANKNNRNAEAEETLEAAEKAIPAEVRMISGCQDSQTSADVSNVASFSLPDPAGRAGGACTSALLKVLYADHH

RTEEDLSFQETLLKMREILSSGRYTQIPQLTSSRPMNIQESFDLVPPDCSGERRALLIGINYIGQQGELSGCHNDVM
NIKEYIIDVHGFPEENITILLDDGEHRDPTRENILDAYKALVSASKAGDACFCHYSGHGGKLRDDDGDEADGYDETL
VPLDYQRAGQIRDDDIFNTLIGPMPKGVTLTCIMDCCHSGTVLDLPYVFKADGESEEMTADPDFDFGPLLQLAVSLF
SDGIQKEDFLKIGSSLLSGVFS

>Espinifer3 CAMPEP_0200433120
MTKKSNSSSTDVDPSAIIPATVHMISGCEDRQTSADVTNVDAFSLPDPAGRAGGACTSALLKVLYYISRSKTPLSFV
DVLMNMRGILSEESFSQIPQLTSSRLLNVSDAFYIVPPTCTGTKRALLIGINYVGQSGELSGCHNDVLNIKEYLIDV
LGFKEKNITVLMDDNESERPTGSNIIDAFKDLASLSKSGDAVYFHYSGHGGKLRDLNGDEKDGYDETVIPVDYDTYG
QIRDDTLYKKLIGAMRSGVNLTCVMDCCHSGTVLDLPYKFVANGSTTEMALDDDANLNLLQEFAADLELEGRWLLPC
IGVLLLMALIAIVLWLFLGRA
>Espinifer4 CAMPEP_0200482898
MGDFLAEAEKAIPAEVHMISGCMDSQTSADVSNVASFSLPDPAGKAGGALTSSLLKVLYADHRKPDGDLSFQEVLMK
VRGVLGGSYTQIPQLSSSRPMDINTPFEIVPDGCTGTKRALLIGINYVGQQGQLSGCHNDVLNMVEYLKDVWGFPPQ
NITVLLDDGSHVNPTRSNILNAYRTLVAESQPGDAVYCHYSGHGGKLRDDDGDEKDGYDETLVPLDYQSAGQIRDDD
LFNTLVGPMKKGVQATCIMDCCHSGSILDLPYVFKADGEQTEMAPSPDFDMGPLLALAGALASGASITEALSQQDPQ
QLMAMAQQCCTIL

>Espinifer5 CAMPEP_0200484576
MAELDRFMNGLASPSPNHLANRSVAASAGNADSGAGDDDTKAATIVLGGANLVGTIPTSGPIDVDEVSQSSLVEEIN
APSGAASSGILLARDGDKKTTQEAEKDASVAAAVAPIVATLSMLSASKSRGGTEASMKKSKSQKKVTIVEQIDEKSL
NDTNENAVDSETIPPRNSNMSVCSSHHTDDASTVVSLQFKVEKKKKKRKTSQNPASISREASSTASSTVMVDNTAGG
KGRSNGGKKTVFRSLKKKLSNKKVPSASSVVSSVSVVGGGPDATIRILSGCSHSNASDIVPNTSSLPYPSGTTGGAC
ASSLLQVLYDAEGGKAKAAGASKKKHDGATSKLSYVDILVKMRSDLTTRGYDQIPQLAASHPVDIAAPFRLKSGKGT
ARALLVGINYAEEENQQSSSHNDVKNMHRYLTEVAGFDADNVTMLMDDGDCPLPSKANIIETWKELAEDSKAGDSVF
FHFSGKGSETIAPVDYENAGQICDGDMYNAFVKKIPKGVTVTCLMDSCHSGTVLELPYVFKADGTDIRSKTMMMNQG
FNSSKFFFGDTFNQLIGESRNGFSTPPRRSSNGRKVASSPSTPTTNSSACDDDLGDDAVTMVSI

>Fkerguelensis5 CAMPEP_0188144146
MSSTLAQAEEYIPANVRMISGCQDSQTSADVSNVATFQLPDPAGRAGGACTAAMLKTLYNDEDNPAMDLSFQDTLLQ
MRDVLSEGKYTQIPQLSSSRPLDIHTKFDLVPDSGFSGTKRSVLIGINYVGQDGELAGCHNDVGNMTRYIQQVHGFT
ETHMTVLMDDGSHQAPTYDNIMSAFRQLVQDTQEGDAVFVHYSGHGGKVADQDGDEADGYDETLVPVDFQGAGQIRD
DDIFKVLIGPMPRNVTMTCVMDCCHSGTVLDLPYTFVADGTQEEMMETPGFDFGTLMKLYQSFQTFKQG

>Fkerguelensis8 CAMPEP_0188102320
MGRFMKKLRQRLEDKIDTVTTSITDGTLSMPEGYPGIAPSQQHEAQRSFESNKPESMRKDVRMISGCEDSQTSADVS
NVRQFQLPDAPAGGGGACTSTLLNILYKDEQIVEDTYSFTEILEQMRINLGKSGYTQIPQLTSLNPIDVTTDFQLIP
EDASGTRRAVMIGINYVGHAQGQLSGCHNDVLNMKKYIMDVHGFEEENIVVLMDDGIHSSPTASNMIQAYQQIVADS
EDGDAIFLHYSGHGTKLRDDNGDEDDGYDEALVPVDFNEGGGMIRDDDLFDILIKPLAQNVHMVSLMDCCHSGTILD
LPYIFAPKLNTDGSGSYSMPQEMTLDETIDLDGLVEQFGGQAMGLLVNFLQKSLGQN

>Fkerguelensis9 CAMPEP_0188100640
MGRFSINNLQTSVERIEKKLLLEKFQKKLLHSKKEDHENAQRDFEERKPDTMKKSVVLISGCEDRKTSTMSNIDMFT
LPDSAGRCGGIVTAALLNVLYWNDDKQKLDEVKQQIQTRTNEIEKSNQDTSERAASYRKVAEDTLPLLQLLVNDGGN
LVDRMEEIIKPTEDEDDTEEDVQEMRKQMDYILSYDLFVNLDKAKKIIYKAFNTSSPLFVSGNIAGCAQIYREAAEK
NILPLLPSNDDLQNKLQEVIASKYDDPAEETRAFREQFDHILSYNEWTDSDKKFEEALDNAKQCIVEAIDNGATYLD
DEDYDSIASIHRKSAEEKILPLLSLNSDLQSRLESVIATEYSDTCVEAAAFLEPFNYILDWTENKEKKLKCMSKNNC

SKEEAQHLTYVDVIDKLRSSIKNSGHDQKPQLTSLTLIDINTEFKLVPDNFKGKRRAVMVGISYDGGLIGCHNDVIN
MKKYLIERCDFKEYYVDVLMDDGVHTLPTKSNIMNALLRVVNDCNDGDAVFFFFSGHGTEIVDENGDEKDGYDECLV
PCDYLEYENFKDGVISDDDLFDNIISELPSDVHMVALMDCCHSGTILDLPYILKANEDGSMPETMVLDDTIDLKSLI
KQYGGRRKLDLLLNSVSKLFIEKEMPPMHVNPVKLGRVLLLADEDAYRHSFARFEGDELNGLIDEKLPRRGKECKVV
TVWGDQTITALFDDGTRFDFPFESVEAQLHVMEMPEAESNEGEHTPTVIFKLHSSLNSVIDIYEVRDGTMVGSISKE
DEDSGTLHMDTHYGHSFRFCTHEESTTLSQQMDMAKSLISTAISIGAPAYNTGDIPECARIYKETALQIAPMLPQNL
QTGLEATIQQTFKNANEEAWAFRKNFDSIMSFVPSSSFKIYYIDQCNGPHQTCVVTSDDDEESNNDEEDSDDDGEEG
GEDEEKSDDDDEESDDDEEDGDDNGEEGGEDEKESVDDDEVSDDDGEEDGEEDGEEDGEDDEESENDDKESL

>Fkerguelensis10 CAMPEP_0188069114
MNTTNNRFLSYDECQQFERNKPGELRDADIWMISGCDDHQTSADVSNVASFQLPESNGKAGAGGACTATLLQVLYEE
EETPHDDYTFTQVLEKMRQRLRSKGFTQIPQLSSTKPIDMSTEFKIVPDDLPGTSRAVLIGINYRGQKGELKGCHND
VFNLYNYIQDWYGFQDENVTVLADDGDHQLPTKQNILAAYHRIVRQSRSGDSIFLHYSGHGTKVPDLNGDEDDGYDE
ALVPLDFDRSGVILDDELYEIFVKGLPSNVHVVALMDCCHSGTVLDLPYIFKADGNFSKMEIDRGSLSASKLFGQLG
GMALKIVMGGSNGGGSGQLVEGFIKHFLR

>Fkerguelensis15 CAMPEP_0188088198
XTLQSIICIMFQQGRERRQNRRGDRRTDRQERRGGNNSRSPARPATTTTTTRATTTTTSTKGRSTAARETTTTATVN
DEGLSFDEQAEQAIPADVRMISGCHDVQTSADVSNVSDFALPDPAGRAGGACTSALLQVIYNEHHLDPTYHQLSFMD
VFLQTRDVIDGMGYPQVPQLSSSRRIDIHQPFNMMKNDSDTGPSGGTRYAVLIGINYTSHKQGQLSGCHNDVGNIKK
YIMDVGKIKAHNITTLMDDAGISIPPTKRNIMKAFDELTRKCQPGDCAFVHYSGHGSRQKDYTGQEESGYNSTLVPI
DFNEAGQIVDDELYEHLVLKMPRGSTLTCLMDCCHSGTVLDLPFNFVADGEQTEMVEMENFPFLQLLQCVGKALQEA
GVSQLRDLRDKDKREQVKAALVENGVGEQLAAARAGAAANAGGAGAGSGQRGGVAGGTVRDNIKSRQERRENRRKFL
R

>Fkerguelensis17 CAMNT_0031427971
TMDEEKIRNAIPANFVMLSGSEDKQTSADVGNVGQFSLPDPAGKAGGACTSALLKVVNENQGRQMSWIDLLHRMRNV
LKDKGFDQIPQLSSSRMIDVNNQFEIVPRQSINEQGARRALLIGINYVGQQGELSGCHNDVKNIKKYLIRKEGFQEK
DMLILMDDGRQHAPTRQNIMEAFDRITQYSKAGDVVFIHYSGHGGKIRDTSGDEADGYDETLIPLDFKQAGQIVDDE
IYSRFVTKMPGNVTVVVVMDCCHSGTVLDLPYEINATQSKMSLNKGFNMGLLNNATVAIGCCLCILSMLDI

>Npunctata1 CAMPEP_0199328580
MGFLNEQQRRAFEEEKPQALRADIWMVSGCEDRQTSADVSNVATFQLPEPSGNAGGACSATLLNVLYEDEKNPLDRF
TFTQVMEKMRGILKRKGFTQIPQLSSTRPIDMNDKFCLVPDDLPGNRRAVLIGINYRGQKGELRGCHNDVFNMYNYI
QDYYGFQDEDITVLVDDADHPPPTKANIIAAYQSVVAKSQPGDAIFLHYSGHGTKLPDDNGDEDDGYDEALVPLDFK
SAGMIRDDYLYDIIVKALPSGVHVVAVMDCCHSGTVLDLPYVFKANGEYRRMEIEDKFDFNKIFRIFGGGGKMGGAM
MTKVGMKMAKGMLKQFLR

>Npunctata2 CAMPEP_0199330992
MESRINDAIPAKFVMISGSEDKQTSADVYNVGSFQLPDPAGKAGGACTSTLLKVLHDNGNRPMSWIDLLHKMRDVLR
QKGYDQIPQLSSSRMIDVSHKFEIVPTETIERNGARRAILIGINYQGQKGQLSGCHNDANNIKNYLINNQGFLEKDM
LILMDDGRHHQPTRANIMNAFDRIVQYSKAGDVVFIHYSGHGGRVVDTSGDEADGYDETLIPVDFQRAGQIVDDDLY
KHLVKRMPAGVHVTVLMDCCHSGTALDLPYEIDATQSKMSFNKGFNMGLLDDPGAMCCLAICAACLFDDIIGGIMSG
LAD

>Npunctata3 CAMPEP_0199330036

MEDAIEQAKKTIPAEIHMISGCRDEQTSADVSNVNSFQLPDPAGRAGGALTSALLNVTYKDEEDTGKDLSFQETLLR
VRDQLKGKGFSQIPQLSSSRPMDISKKFDIVPDNMHGTRRAVMIGINYVGDNPGELRGCHHDVLNMKKYIMDVHGFQ
EENITVLMDDGEHIPPTRENILGAYRKIVAESESGDVVFCHYSGHGGKLRDDNGDEEDGFDETLVPRDYQTAGQIRD
DALYDTLVAPMKAGVFATFVMDCCHSGTVLDLPFNFVADGEQEEMKLQEGFDFSPLLAFAAAYMASQQAGDDPVSGL
LSACGACLIQ

>Npunctata4 CAMPEP_0199338742
LPSPAGRSGGACTCALLSILYDAEQNKKPITFQKVLLELRRRLSESGMTQIPQLTGSRPLDLHETPFSFVNPRENSI
NTRRALLVGINYFGQNGQLSGCINDVNNVKKYLCQYEGYLEKNILVLTDDGRNPHNPTREKIIRALRQLVAQSVSGD
SVYFHYSGHGGLLDPDYWNRFKAGKLHKEYDETLYPVDHERAGQIRDFSLFNHFVKPMSAGVTVTCVMDCCHSGSVL
DLPYSYRPTSAGTIRMRQSMDTLTNLAFLYILAGGMLPHHGGLFDSVTQNLQDVTGVSVDSLLGTGVEEMTFDVEGL
GDYGDHMIQDNGGPSDITVDALDNQYQGQDRAFDEFGQGDVPDVGIGHTFSRNFGIGESGFVDSDTGGDGSDLNGTD
VDCGENDIDCADCGDIGDILGNILEDL

>Npunctata6 CAMPEP_0199343924
MGRFLRKLRERRERRRIGNQEEAQRAFEEEKPDAMRKDVRMISGCEDKQTSADVSNVHSFQLPDPAGRAGGACTSTL
LRILYKDETIPEDTLSFTQVLEKMREDLAEQGYTQIPQLTANNPIDVEADFDLVPSTATGTRRAVMIGINYIGDDPG
ELRGCHNDVLNMKKYIMEVHGFEEDNIVVLMDDGEHDPPTRDNILAAYEKVVADSEAGDAIFLHYSGHGTKLRDDDG
DEDDGYDEALVPRDYNEAGMIRDDDLYDILIKPLADGVHMVSLMDCCHSGTVLDLPYIFKADGGATPNVMQLDDTID
LDGLIQQFGGHAIGFLKNFLEQQG

>Palata1 CAMPEP_0200176934
LERDELISGVIRAAPKNDAANIADAVYALWSSWDVDGDGRISVDEFCRKGGLGEEIVRNLNLEVNDKGDTRVNFYGP
PAVNPAFHNLQPYTNTASTAASQQAVPSQPATWNCLACTFIXFIESNILQPCVFFLLKDVNKHNQCFPLILSLLCLG
HSHVNSILGVCTTDVPTAVPSQPATWNCLACTFINLSSQTSCKACGTPQVTTPPTIHHAFSPPSAISQPYPHGSSQV
PSSPHAGHKTFRVKVPPGMKPGQKVKVSTGPTTSDAVVVIPPRHAWSNNDHSNGRWEPSTFEVTVTMGDNQENIYQS
PNATNHTYTHHSPNHTANHTNHTYTNHSTNHTSHIPNRINNSYHHIPERFHDGRYGSGDVPWMPFHQFQSQKYKPPP
TKMISTPLTSNHSPINASGRRKALLIGINYTNTRASLRGCVNDAMKIKGLLVENGFTDDAAHMTLLIDETPEPHAGG
GGAVSSYLPTKANIVKGLQWLVSGSRKGDVLFFHFSGHGAQVPDATGQEADGLNETILPLDYQKAGMISDDTIWGSM
VYPLAEGVRLTALMDCCHSGTGLDLPFECQLKRNLSSVNNTDYQWVEDINPAHSRGDVVLLSGCEDAQTSADAFDRH
RKEAGGAMTTAFIDAYRAHPAGSTYPEILNSISEQLRRKNFAQRPQLTTSQRFDVRRRIFSLVEGIEPNGNAQIGRL
KRRHFKPAKNVTAIDPRLNELLFGGALAIGLASILFD

>Palata2 CAMPEP_0200175712
MTEITPYDYYDVVTPGTADERDYIGETYQQSDVISYWHGLGRGGNTDDVSSLNSEGIQEYINGALIISILLLIFIVI
WGGIIIFLRIYDDCIPGKIKFLSGWRFENTSSTSLMPFIIRITFIFCAICVYIFTMIYVQKGIHGFEDATKIAQAQN
TRFRDVVEEADRVVITMLANGEEASGLKDRIKRDIPYLCPKRNISNLGTDFLFTLNKYTNSLDQLGDFYIDQRWKPA
EMWVERLLNITVKFDDTIDEINLPLFAIHWIPLIVLTTILLIATLLSIYDNGREDLKIRLAPWFQWCLKKIVFPLFT
LWSIGTFFLAAAVAFGLVVNSDLCAGGPGAGSPDFPFMRLVDSSGLEGTRTGGAVYKFIKYYSRGCDKRFRDKGSTF
YEGNLTRALSQTEYITDFILDKGTIEMSDDCGNDVTGLMVALDELRTHMQYLKVGTKRIRDVLRCKRLNNIYTELFY
NGLCYHSVNALAWSYCSLLAIGFFSMLMVTLRSSILPSYEYSWSEEFDIDGDNVSDDQTMEEDSYELESNTMAFPAI
VRMISGCQDSQTSADVSNVASFSLPDPAGRAGGACTSALLKVLYSLIDSSCEISWEDLLIKMRENLNDDGYKQIPQL
TSSRPTDLKSNFELVPSDGSATGAKRALLIAINYVGQKGELTGCINDALNMKKYIMDVWGFEEENITMIMDDGEHME
PTRENILNAYNDLVIQSEAGDSIFCHYSGHGGKLRDDNGDEEDGYDETLVPVDYARSGQIRDDDLYEILVGCMPRGV
YMTCLMDCCHSGTVLDLPYKFVAPERRRK

>Palata3 CAMPEP_0200178272

MSPSTLCCRKNAFSVADTENAIRSTVRMISGCMDSQTSADVSNVSQFQLPDPAGRAGGACTSAFLRVMYNEQQRPSY
DLTFAEVLMGMRDVLSNGSYSQIPQLTSSHALDINEPFQLHSLTSFRGKKHALLIGINYVGQSGELSGCHNDVWNIR
SYLIDVHGFEERYITILMDDGSHTSPTRENIMSAFRMLVKSSVGGDSAFCHYSGHGGKIRDNNNDESDGYDETLVPL
DHYASGQIRDDDVYDALVGPMPSNVTLTCLMDCCHSGTVLDLPYKFKPDNTATDKMVLDPNFNDAKLVRFARKLFGL
ATETGIKRNDTFVDKEGQLWRKAEDGEPLPFKLYKHQLIQRDIEEHNNENIIKKIEDMGIKVYIVSHNSSDLLQCID
DVAEEVSNFSIKTNGRKRCCFLILTEEGRLYNLALGGNVGKTINGLAGQWEALLNSFSGLEATIAIAVLARSFGCAE
VGKGYELRPGYLSLILGYILSEEMGLSWQNVAMFVRGIPRISQLNPWIYPE

>Palata4 CAMPEP_0200144054
MGLFSRLREKLESLDDEKVVIKADVRMISGCMDAQTSADVSNVDDFQLPDPAGKPGGACTSALLKVLYDEEKTPTDD
LTFSEVLEQMRTVLDEEYEQIPQLSSSHALDVSTPFTVVGGEGTKRALLIGINYIGQNGELSGCHNDALRIKKYLMD
VYGYEEENFTYLMDNGVYDDPTYDNILAAFDDIVAKSEAGDSVFVHYSGHGGFLADDSCDEEDGKDETLIPVNYTEA
GQIRDDTVFNRLVLPMADDVTLTCLMDCCHSGTILDLPYKFKAGMGESVMSLQPGFRFDNFFRKIGLSDEE

>Palata5 CAMPEP_0200147548
MSAFGEQAQKVIPSEFRMISGCHDAQTSADVSNVGAFQLPNPCGKAGGACTAALLKVLYEMNERRETWSWVDVLRKM
RTNLDGMGYEQVPQLTSSRMISVTDSMEIVPATQQGVRRAVLIGINYVGQSGELSGCHNDVFNVKDYLVNVLGFENR
HITLLLDDGLNTSPTRENITYAYRDLVNRSAPGDVAFCHYSGHGGFVDDTSGDEEDGRDETLIPVDFQSAGQITDDE
LLKDLVHPMKAGVLTTCLMDCCHSGTVMDLPYRFTADGDNIQMRENPSSNMLEYGDLIATFAGAYALTGLLGAGGGM
GGMGGGSPNNTGEEEEADCCTCIMDLVNSFL

>Palata6 CAMPEP_0200160162
MSSTLEQAEKYIPANVRMISGCKDSQTSADVSNVASFSLPDPAGRAGGACTAAMLKTLYNDEEDRGVDLSFQDTLLQ
MRKVLDQGSYTQIPQLSSSRKLDIHTKFDLVPDDGFEGTKRAVMIGINYVGQQGELAGCHNDVGNMKRYIQKVHGFT
DEHMTVLMDDGQHEEPTYGNIMAAFRQLVENTKEGDAVFVHYSGHGGKLRDDDGDEADGYDETLVPLDYQEAGQIRD
DDIFSTLIGPMPRNVTMTCVMDCCHSGTVLDLPYSFVADGSQDEMTETPGFDFGTLIKLFQSFQNGGGGGISPEVMQ
GIQKIASKCFSMFS

>Paustralis1 CAMPEP_0199647600
MPLRLNRKDRSGRENAAAVSQQNNPSHSKNAATSKNNFDEQVEYLIPAQVRMISGCHSLETSADVSNIHSIAGKGKL
PSPEGRAGGACTTALLSILYDAQKRKNDNHYSGDGSTTFQQLLLELRRRLAQTGMSQVSQLTSSRPLELEETPFSLR
SIPATNQQQQQQQHGGGGTQRALLVGINYYGQRGQLSGCINDVLNVKKYLCNYQGFLEKHVLLLIDDGRNHHPTRD
NIIRALQRLVKQSKPGDSVYFHYSGHGGLLDPNYWNRYKAGVSNKKYDETLYPVDHLKAGQIRDFNLFNHFVKPMAA
GVTVTCVMDCCHSGSVLDLPYSYRPTSDGTIRMRQSMDSLTNLAFLYILAGGMLPDLGFESIAQNLENATGETMDNL
QGIGVEELSSDIGGYTNDYTNDGDVVTGATCDNTDLTGIAGDDRDICSSNGDFAYEGDEITGEDITYDIDSGGFDVD
GGTNLGGAQDGFLADDYNPDGDCEGNECDGCDVIGEILNTLFEDS

>Paustralis2 CAMPEP_0199652308
MDAKIESAIPANFVMISGCEDKQTSADVGNVSTFALPDPQGKAGGACTSSLLKVLNENQGGSMSWIDLLHRMRSVLK
SKGFDQVPQLCSSRMIDVNNQFEIVPEEAQGCRRAVLIGINYEGQQGELSGCHNDVKNINKFLRKEQGFHQSDMMIL
MDDGSSTPPTRRNIMDAFDQVVQNSTAGDVVFIHYSGHGGNVPDESGDEEDGYDETLIPVDFQSAGQIIDDELYERL
VTKMPAGVTVVVLMDCCHSGTALDLPYQINATQSQMSLSEGFNFSLLDNAAGVIGCCLCLMSILDMVM

>Paustralis3 CAMNT_0045498567
FQKLIPYTMGRFFKKFTQHVQNQVKAAMGDQSDAQAAFEDQKPDSMRNKDVRMISGCEDAQTSADVSNVANFCLPDP
AGRAGGACTSTLLNILYKDDQVPEDTLSFTQVLEQMRSDLGSGGYSQIPQLTSLNKIDVETDFELVPSTATGVRRAV
MIGINYVGHSPGELRGCHNDVLNMKKYIMDVHGFEEDNIVILMDDGIHESPTKENMVAAYKQIVADSEEGDAIFLHY

SGHGTKLVDKSGDEDDGYDEALVPLDFQEVGMIMDDDLFDILIAPLQDGVHMVSLMDCCHSGTILDLPYIFKPNQDG
SMPLGMMLDESIDLDGLLQQFGGQAVGILLNFMQKACC

>Pfradulenta1 CAMPEP_0199827318
MGRFFKKFRQNIREKIEEAIGDAIGADGGGFIGNQEEAQNEFEDKKPDSMRGKDIRMISGCEDSQTSADVFNVVDFQ
LPDPAGRAGGACTSALLRILYKDEHIPEDTLSFTQVLELMREDLSEEGYRQIPQLTSLNKIDVETDFELVPHTATGV
RRAVMIGINYVGQDGELRGCHNDVLNMKKYLMDVHGFEEENIVILMDDGVHDEPTRENMIEAYQQIVDESEDGDAVF
LHYSGHGTKLVDGSGDEEDGYDEALVPLDYDEAGMLLDDDLFEILIEPLAEGVHMVSLMDCCHSGTILDLPYIFKPN
DDGSMPHAMKLDDTINLDGLVEQFGGQALGLLVNFVAESF

>Pfradulenta2 CAMPEP_0199811974
MDAKMRNAIPARFVMISGSEDKQTSADVGNVGTFSLPDPAGKAGGACTSTLLKVLNENGGQPMSWIDLLQKMRSVLK
YKGFDQIPQLSSSRVIDVNNQFEIVPESSGGRRRAVLIGINYEGQQGELSGCHNDVLNIKNYLVREEGFQEQDMIIL
MDDGQHNLPTRKNILDAFDRIVQYSKAGDVVFIHYSGHGGRVYDSSGDEADGYDETLIPLDYQSAGQIVDDDLYRQL
VTRMPEGVTVVVLMDCCHSGTALDLPYEINATESEMRTNPGFNMALLDAGAVVSCCLCLNVLFELLASLDGGDGGDR
GDRYDSGE

>Pfradulenta3 CAMPEP_0199786516
MPSLFNRKNRHGGGLGSDNFDATTTGTDNNFDNQVRQLIPAEVIMISGCHSLETSADVSNVKTIDGHLPSPEGRAGG
ACTTALLSILYESLKTTDRNNSPREISFQQLLLRLRGRLAQSGMSQIPQLTSSRPLELEERPFSLKSNNPNGGTRRA
LLVGINYYGQSGQLSGCINDVLNVKKYLCNHHGFLEKHVLLLIDDGRNHKPTRENIIRALRRLVEHSKAGDSVYFHY
SGHGGLLDPDYWNRYKAGKKDKQYDETLYPVDHARTGQIRDFSLFNHFVKPMTAGVTVTCVMDCCHSGSVLDLPYSY
RPTEDGTIRMRQSMDSLTNLSFLYILAGGMLPDFGFESIAENLQSATGEAMDALQGTGVEEFSSDLDGGINGYGLGN
GDDVDDAMNYTSGLETGNVDTGFDDSNVDYGGNGVDDFDLQNTDIAENAYGDGDRGDAFFGAFRDTYFADETVGDAD
CGDVNCGDADCNDGCDVVGAILGALMDSDS

>Pfradulenta4 CAMNT_0045629199
RQQQQQKQQQQQRQQQQVLRRTDQQNTSRLSGKRLSCGTLANKKATKATTRTTQHSHTRHEIQATIGANKQTNMFGK
LGKQYAAQVAEKVEAFALEKLNERNPQSTVTEETTEKLKSISGNRKALFIGINYKGQRGELKGCVNDVVNIKKFFRT
HYTLNDTLVLTDDKRAEPETDHAPTRKNILNAFKWLVKGAKPGDSLLLHYSGHGGKSKNHDGTEASGFDQTLIPVDY
EKAGQILDDDVHDVLCKSLPKGVRLTAIFDCCHSESIMDLPFVYNVNGNLEIVENDRNQSIATIMATGTRFLLDGNR
KKAGQALKNEITSMLSNVMAGGQDPKAQEARKKVIASNQTKADIIMFSGCKDMQTSADTSQQGEAGGAMSFALIKTL
EKHHRDKKDLTYTELLREMRQVLEGKYTQVPQLSA
GRRLVLDHPFRV

>Sdohrnii1 CAMPEP_0192149910
XISGCHDAQTSADANITSFQLPNPAGRAGGACTAALLQVLYDRPSIAAGNDNTSWVQVLRRMRENLDEQGFEQVPQL
SSSRIIDVNERMTIVNDNPSGVKRAVLIGINYVGQQGELSGCHNDVKNISSYLQQVQGFRPQNMITLMDDGVHDNPT
YDRILQAFQWVVNESQAGDTVWIHYSGHGGRVEDDNGDEDDGYDETLIPVDFQRKGQIRDDDLLRYLVKPMREGVLM
TCLMDCCHSGTVLDLPYRFIADGDHYQMERNDDFDMDHLIEVAKIAATVAASLGGMAQSVIQSSGLVEEIDECCVIL

>Sdohrnii2 CAMPEP_0192135274
MGFFNKLKEAALKEAKKKIMEELGLGGDDDETKPTMTTEEFEKTKPDELRKDIRMISGCQDRQTSADVSNVSSFQLP
DPAGKAGGACTSTLLNILYADEHVPEDDLSFVEVLEEMRIKLKSGGYSQIPQLSSMNPIDVNTKFDLVPDTATGTRR
AVMIGINYIGDDPGELSGCWNDVLNMKKYIIDVHGFEEENIVILMDDGIHTEPTKANIIDAYKTIIAQAEENDAIFL
HYSGHGTKLRDDDGDESDGYDEALVPRDFQTSGMIRDDDLYELLFKNLPDGVHMTSLFDCCHSGTILDLPYLFKGDG
SQTEMTLDPNMNLDAFIEKLTGKLMEFLQAKMAASLSL

>Sdohrnii3 CAMPEP_0192159136
RGKPPPPPPRRQSSSTAGQSKKADATIKRSSSTREARPVKSNANSKSSRRDSQSRSKSRPRDDSRNSKPRDKSRSRS
KSRPRSSPRNNENDTQQRRSKSKSSSSRSKPPQSKGNRSKSSPRSGTLHGKRSSSSSNKGNKAQAESFNAKKRQQKF
ASQAKKAIPTNVRMISGSHDAQTSADVGNINAQFELPNPAGRAGGACTAALLQVLYDAHDRGDINMSWVDALREMRE
ILHEKGFDQIPQLSSSRMIDVCDPFVITPESFDPGFNKQRAVLIGINYKGQKGQLSGCHNDVHNVTQYLKEVQGFKD
ENITILMDDGMHKPPTKSAIISAFKRLVKQTKEGDVVFCHYSGHGGRLPDDNGDEDDGYDETLIPLDFQKSGHIRDD
DLLKILIHPMPAKVTMTCLMDSCHSGTVLDLPYRFVGDGDHVEMEMNDRVNFTEALWTGLSVAAGVVAGNAIADAVN
VAPEMMILGGGPNAIHEHX

>Smarinoi1 CAMPEP_0192232734
MGFFNKLKEAALKEAKKKIMEELGLGGDDDETKPTMTTEEFERTKPDELRKDIRMISGCQDRQTSADVSNVASFQLP
DPAGEAGGACTSTLLNILYADEQVPEDDLSFVEVLEEMRIKLKSGGYSQIPQLSSMNPIDVNSKFDLVPDTATGTKR
AVMIGINYVGDDPGELSGCWNDVLNMKKYIQDVHGFEEENIVILMDDGIHTEPTKANIIDAYKTIIAQAEENDAIFL
HYSGHGTKLSDDDGDESDGYDEALVPRDFQTNGMIRDDDLYELLLKNLPDGVHMTSLMDCCHSGTILDLPYLFKGDG
SQTEMTLDPNMNLDAFIEKLTGKLMEFLQAKMAASLSL

>Smarinoi2 CAMPEP_0192224422
XQIPSEVRMISGCHDAQTSADANITSFQLPNPAGRAGGACTAALLQVLYDRPSIAAGNDNTSWVEVLRRMRENLDEQ
GFEQVPQLSSSRIIDVNERMTIVNDNPSGVKRAVLVGINYVGQQGELSGCHNDVKNISSYLQQVQGFRPQNMITLMD
DGVHDNPTYDRILQAFQWVVNESQAGDTVWIHYSGHGGRVEDDNGDEDDGYDETLIPVDFQRKGQIRDDDLLRYLVK
PMREAVLMTCLMDCCHSGTVLDLPYRFIADGDHYQMERNDDFDMDHLIEVAKIAATVAASLGGMAQSVIQSSGLVEE
IDECCVIL

>Smarinoi3 CAMNT_0036352755
PRSSPRNNENDTQQGRSKSKSSSSRIKPPQSKGTRSKSSPRSGTPHGKSSSSSSSNKGNKAQAESFNAKKRQQKFAS
QAKKAIPTNIRMISGSHDMQTSADVGNINAQFELPNPAGRAGGACTAALLQVLYDAHDRGDVNMSWVDALREMREIL
HEKGFDQIPQLSSSRMIDVCDPFVITPESFDPGFNKQRAVLIGINYKGQKGQLSGCHNDVHNVTQYLKEVQGFKDEN
ITILMDDGMHKPPTKSAIISAFKRLVKQTKEGDVVFCQYSGHGGRLPDDNGDEDDGYDETLIPLDFQKSGHIRDDDL
LKILIHPMPAKVTMTCLMDSCHSGTVLDLPYRFVGDGDHVEMEMNDRVNFTEALWTGLSVAAGVVAGNAIADAVNVA
PEMMILGGGPNAIHEHSDTACCAVS

>Smenzelii1 CAMPEP_0192261006
XRCRLNIYQGEKDDSPATASSYTCIPLQCRRRGDHQAEESIRRLHHPEGSLKTQVKAGLIQHSRDPHQREERQDQPS
LMLTTKPDEIIEVGANPDQEMIILVTQNRGTSLDPGANRDPDPLLEKIMNTIHKRDGGANQNLLRPGANTXAKSNRS
KSNPRSSAPQAKSSSSSKGNKAQAESFNAKKRQQKFASQAKKAIPANVRMISGSNDVQTSADVGNINSQFELPNPAG
RAGGACTAAILQVLYDANDRGDVDMSWVDALREMREILHEKGFDQIPQLTSSSMINVSDPFDITPQSFDPSLNKQRA
LLIGINYKGQTGQLSGCHNDVHNVSQYLQEVQGFKEDNITMLMDDGMHKPPTKSAIISAFKRLVKQTKEGDVVFFHY
SGHGGRLPDDNGDEDDGFDETLIPLDFKKSGQIRDDDLLKILVHPMPANVSMTCLMDCCHSGTVLDLPYRFVGDGDH
VEMEINDRVNFTEALWTGLSVAAGVVAGNATADVAPEMMILGGGPNAIHEYS

>Smenzelii2 CAMPEP_0192280530
MGFFNKLKEAAVKEAKKKIMEELGLGDEKDDDTKPTMTTEEFENTKPDELRKDIRMISGCQDCQTSADVSNVSSFQL
PDPAGRAGGACTSTLLNILYADEHVPDDDLSFTEVLEQMRVKLRRGGYSQIPQLSSMNPIDVSTKFDLVPDTATGTK
RAVMIGINYVGDSPGELSGCWNDVLNMKKYIQDVHGFEEENIVILMDDGNHTEPTKANIIDAYKTIIGQAEENDAIF
LHYSGHGTKLRDNDGDEGDGYDEALVPRDFQTSGMILDDDLYDLLIKDLPDGVHMTCLMDCCHSGTIMDLPYLFKGD
GSQTEMILDPNMNLDAFIEKLTGKLMEYLQAKMAASLSL

>Smenzelii3 CAMNT_0036412763
ADCSDIVAEEPIKQYSVLSRNKMPSFAEKVASQIPSEVRMISGCHDAQTSADANITTFQLPNPAGRAGGACTAALLQ
VLYDRPSIASANDNTSWVEVLRRMRGNLEQQGFEQVPQLSSSRIIDVNEPMTIVNDNPSGVKRAVLIGINYVGQQGE
LAGCHNDVKNISKYLQQVQGFRPQNIITLMDDGIHDNPTYDKIMQAFQWVVNESQAGDTVWILYSGHGGRVPDDNGD
EDDGYDETLIPIDFQRKGQIRDDDLLRYLVKPMRQGVLMTCLMDCCHSGTVLDLPYRFIADGDHYKMERNDDFDMDH
LIAVAQMAAAVASSFDVGSVGRMAQSVIQSTGLVEEIDECCVIL

>Skcos11238
QTQYQTANNGSSYATQQQQHHTSYVTQPMPTSAPVXPTTSTQPLQQPVVATAYASGGGGTSAPPVVATAYVPSSTNA
TTASSATAAPILATAYIPANELDDGHTRPSAPPMNPSYNPNYSATTGNNNFHTHQDEFWECSVCTFPNRQSETHCKG
CGNAQPGGNSSSYNSKPQAPSAVQSSAAPSYSTMNDINSHMASISLGTGTYPNGINMMSSSAPPPSSTSGTMKVHIP
AGMRSGQKLKVRSPAGDEVVKTIPNQSEWSYEIDGRPYFRLQFGPSQTAAASTASPAMSAASTMKVHIPNGMGPXQX
IKVRSPTGSEVVKTIPNQSEWSYEIDGRXFFRMAFGDEXNATNYSYSTTLSTPPPPHSTTWREFHTRASSRYNPPPI
GMKPVQHVPRGVSSITPNGRHKSLLIGINYTGTRAALRGCINDAKNMQTLLLKNGFPNDGSHMLMLTDERHRGSEYQ
PNASNIMKAMSWLMKDAQKGDVLFFHFSGHGGQVPDKTGHEADGFNETLIPLDHTRAGQISDDVLWGSLVYNLPEGA
RLTALMDMCHSGTGLDLPYDYNVNTRRWTEDVNPAHSRGDVVLFSGCEDSQTSADVQGYSGXGGAMTLAFTKAYQQC
SSSTYHEFLSVVKXELRKKRHSQRPQLTSSQQFDASSRIFSLGHDNGGITSMIEPNHNPQVGRQKRRHVRPARQGFG
RAGGGNDLFGLGIAAVGAALFADALF

>Skcos12022
GVEVLRRMRENLDEQGFEQXPQLSSSRIIDVNERMTIVNDNPSGVKRAVLXGINYVGQQGELSGCHIDVKNISSYLQ
QVQGFRP*NMITLMDDGVHDNPTYDRILQAFQWVVNESXAGDTVWIHYSGHGGRVEDDNGDEDDGYDETLIPVDFQR
KGQIRDDDLLRYLVKPMREGLLMTCLMDCCHSGTVLDLPYRFIADGDHYQMERNDDFDMDHLIEVAKIAATVTASLG
GMAQSVIQSSGLVEEIDECCVIL

>Skcos13618
GELWRRNFAINATVHKSSKFTVMSAKNNDLEEYEVEKVVDHRYRKTAFPDGSHDEYLIKWKGYPSSENTWEPESNLN
PSALAEARTLKWTTEQFENTKSDELRKDXLCISACNDXATAADVHNVKKXFKLPDAAGKFGGHGGGACCSSLLSILY
ADEKVLEEDLSFKEIIWKMRKTLIKDPYRQIPQMSSMNPXDVNTKFDLFPDTATGTKRAVMIGVNYVGDEDNELSGS
HNDVLNMKRßYIQEVRGFEEENIVILMDDGKHTNPTKKNIIHACKKVIRQAEENDAILFLYSGHGTRVEDDNGDERD
GFDEAIVPRDFEENGFILDDDLYEILIKDLPKGVSMFSLFDCCHSATIMDLPYLFKGDEMQTEMTLDPKFNLDAFIE
KITGNLKEFVQAKMAAKQKKKAAGSKRKVKPEPSKEKKKKLEQEHE

>Skcos16234
SRGKPPPPPPPRRQSSSAAGQSRKAELTIKSSSSTRGEARSVKSHANSKSRRDNQSRSKSRPRDDSRNSKPRDKSRS
RSKSRPRSSPRNNENDTQQRRSKSKSSSSRSKPPQSKGTRSKSSPRSGTPHGKSSSSSSSNKGNKAQAESFNAKKRQ
QKFASQAKKAIPTNIRMISGSHDMQTSADVGNINAQFELPNPAGRAGGACTAALLQVLYDAHDRGDVNMSWVDALRE
MREILHEKGFDQIPQLSSSRMIDVCDPFVITPESFDPGFNKQRAVLIGINYKGQKGXLSGCHNDVHNVTXYLKEVQG
FKDENITILMDDGMHKPPTKXAIISAFKRLVKQTKEGDVVFCQYSGHGGRXPDDNGDEDXGYDETLIPLDFQKSGHI
RDDDLLKILIHPMPAKVTMTCLMDSCHSGTVLDLPYRFVGDGDHVEMEMNDRVNFTEALWTGLSVAAGVVAGNAIAD
AVNVAPEMMILGGG

>Skcos4616
LAKMGNQESVPSPVGDEEIPMDASPQVGKHSGSKLHSPQYMEGVPEPNKKSSNRSKSKDKRSSRERPERGEAREDEN
RDSSVEQHHNKRISPASTDSPSMSYANNFATLPPRGNKRITDDQVHLDLPMAELMAYLQMVANHSSNLPLTRRDDPD
LGRTVSSLTADEYAFKCAAFIPSKIRILGGQFGKYGKVWDLPTSEEFNAKSSTREPGISYGGACSNALLKAIYDTES

EINNVASPHMVDAANLFEDDDDQTVNTAGFSIDQNSKSFDKLSFDDSNATSLTWAQLLHKMKGEMHGMGFNQVPAIT
SSYKFDLNKPFSLVPPEFKVGVNKKRTLLIGCNYRKTRDAQLKACHDDVTSIKDFLVNVYGFPESPDLMTILMDDKK
HKSPTHKNITEAFKRLAEQSQPGDAVFVLFTGHGCRIMDSPIDATAESYDEALLPSDYEESGIIRDTLMFKTLFAPM
KKGVTVTCIMDCAHTGVMIDLPYLWTSKDSKKEEQQAKMSLNNDFSFVRFLKVVKTLYESSVFTRIGKTVGAELAKQ
LSAADDETAVDSVASLETMPENDINDKDNAGILCSIAKLITCQPDEYSDEEHFDDTGSYDSR

>Tnitzschioides1 CAMPEP_0200188848
MGRFFKKIFGSSGAGSKISDQQQARDAFEEQKPAGLRKDIRMISGCEDAQTSADVSNVASFALPDPAGRAGGACTST
LLRILYKDDTVPEDDLSYTQVLTQMRDDLAAQGFTQIPQLTASNPIDVNTDFDLVPATATGTRRAVMVGINYVGHNP
GELRGCHNDVLNMKKYIIAVHGFEEENIHVLMDDGEHEEPTRANLIAAYEKIVAETESGDAIFLHYSGHGTKLRDDD
GDEDDGYDEALVPLDFKETGMIRDDDLFDIVCKPLADGAHMVSLMDCCHSGTILDLPYIFKADGSSTQMQLDDSIDL
DGLLQQFGGAAISFLQNFLG

>Tnitzschioides2 CAMPEP_0200208386
MSDFDAQVQKLIPAEVNMISGSHDSQTSADVYNVSQFQLPDPKGRAGGACTSAVLKVLYNDSQEPSEQLTWVTLLRR
MRSVLNSMGFDQIPQLTSSRMIDVNKPMYIVPPNANGMKRAILIGINYTGQRGQLSGCHNDVDNISEYLQKVHGFEA
TNMLRLVDNGVDHAPTHANIMSAFSRIGDYSKAGDVVFLHYSGHGSRVPDRDGDEDDGYDETLVPVDFERSGQIVDD
DILRKLVKPLAAGVTMTCLMDCCHSGTVLDLPYRFTADGDVW

>Tnitzschioides3 CAMPEP_0200209970
MGDLLEKAKRAIPADVRMYSGCRDEQTSADVSNVTSFSLPDPQGRAGGACTATMLQILYADEKKLEEDLTFQEVLLK
MRDVLKEKGFSQIPQLSASRPIDIKEKFDLVPDNIAGTKRAVMIGINYVGDNPGELRGCHNDVFNMKKYIQDVHGFE
DENITILMDDGNHTVPTRENILAAYSKIVEESQPGDAVFCHYSGHGGKLKDDNGDEADGYDETLVPVDYKTAGQIRD
DLLYKNLVGGFKEGVFCTFVMDCCHSGSVLDLPFQFQADGESDSMEAPADFDFGPLLSLAASLAGGQNIGTDPGKLL
AMCGSCIVS

>Tnitzschioides4 CAMNT_0046182537
ALPYNPHYQPSPTAVQPVTAQPVMAQPVAAQSFNAQSVTTPQPVMAQPVTAQSFNAQSVTTPLPVMAHPITAQPSNA
HSVTTPLPVMAHPVTAQPVGGQPAIAQPVMAQPVMAQPATAQPMSTQQQPVVAQAFVPPQYQQPQLVAPVPAQVYNP
YAVPSLPPPSLSNTSAWFDYYDKDRSGSLSKSEIMQGLLYTFGAKTPNEQYSLQGTVDSIWSIFDTDRNGTLDKREF
CAPGGFGESLQAAMASNGKATATTPHQPVVSSTGNSWTCTKCTFANSMADSYCKMCQAIRVGGGGATNNAIAPAPGV
YVANSSSAAATTIYGGAATNNTITSVPGVYVPGSSAAAATTTTNSWNCTKCTFANATSQSHCRMCQFPRNGTTMNAS
APVNTKIRVGIPQDSSPGQRIKIQTPAGKSEIAIIPPRSEWVFLNTGQPAFDYSIATTSTTTTASAVSVPAPTNTTI
RVGIPQGTNAGQRIKIKTPAGGSEIVTVPQQSQWVYLNTGQAAFDYELPTATSASAVHVQSSSQHPWQEYEQITKSS
YQSPAPLGVMKTVPISIGSMIQPSGRRRALLIGINYTGTRAALRGCINDAKNMRNVLIRKHAFPGDTCHMVMLTDEP
SRGRNYQPTYSNIRRGLQWLLQGVSQGDVLFFHFSGHGAQVPDKTGHEADGLNETILPLDYENKQITDDELWGSIVY
PLPAGARLTALMDCCHSGTGLDLPFEYMYKQNKHNKYNQSSWMEDLNPAHSQGDVVLISGCKDDQTSADAFQQGTAG
GAMTQAFLTAYEQHPYCTYPELLQAIHQALKQRRFTQRPQLTASQPFNTQERIFSLVEGIEPNHQQSIGRLKKKHIR
PGRTGGGGGGGNAGNMLLGAAGIFGALALGDALFDF

>Tantarctica1 CAMPEP_0200089382
MSYQDNIDAEIPAEVRMLSGCHDTQTSADANITTFELPDPAGRRGGACTAALLQVLYNDNDGDPQDCSDQSWVEVLR
AMRNNLAAEGFTQVPQLSSSRMIEINDPMQIVNNPQGTKRAVLIGINYVGHDPGELSGCHNDVANISKYLKGCLGFE
QDNMMVLMDDHRHEEPTYRNIMRAFDWIVSESQPGDTVWIHYSGHGGRLEDQDGDEEDGYDETLCPIDFQTAGQIRD
DDLLKHLVKPMKKGVLMTCLMDCCHSGTVLDLPYNFIADGEHAGMERNENFDLGNVMGMLGAVAGAAMASGAVGEIA
DECCVIL

>Tantarctica2 CAMPEP_0200100074
MGFFNNIKAAAIEKVKEEIFASLGLDGGDDDGDGGQMTTEQFEKSKPDELRKDIRMISGCQDKQTSADVSNVSSFQL
PDPAGNAGGACTSTLLKILYADEQVPEEDLSFTEVLEQMREHLSTEGYTQIPQLSSMNPIDVNHKFDLVPDTATGTR
RAVMIGINYVGDSPGELSGCWNDVLNMKNYIMDVHGFEEENIVILMDDGENIEPNYENIIDAYKAVVSQSEDGDAIF
LHYSGHGTKLRDDNNEEADGYDEALCPRDFQSSGMIRDDDLYEILVKELRDGVHMTSLMDCCHSGSIMDLPYIFKGD
GSQTEMILDPEMNMDAFIEQITGKLMEFLMKKLGL

>Tantarctica4 CAMPEP_0200125594
MTEEKRPSKKSSSHHRSNPPPPPPRRQNSSGGGKERSKRENNGGGDAGRSVGTKSMATKASVRSSRTHETNHTNRSS
RSKSRPRESPGKSRSRSKSRPREAPRESRSTLDIKSKPSDEKSRSRSKSRPRQSSSKSQPQDDTRSSSNKQSKSPKQ
KSKKSPKPKHNSNTTKSKSHNNGNGTRSSLKKQPSVKKREKFGNFAHKAIPAAVRMISGSHDAQTSADVSNISSQFQ
LPNPAGRSGGACTAALLQVLYQSHEYGKDDISWVDVLRQMRDVLDEKGFEQIPQLTSSRMIDVHDPFAITPTSFSHK
KNTQRAVLIGINYTGQSGELSGCHNDVHNVAKYLMEVQGFKKENVTILMDDGAHKPPTKAGIVNAYKRLVKESKEGD
VAFCHYSGHGGRLPDDNGDEEDGYDETLIPVDYDKKGQIRDDDLLKILVHPLQEGVTMTCLMDCCHSGTVLDLPYRF
TSDGDLEEMEINDRTNFKDALWTGMGLGVGMAAGNAIGNAVVSAVTPDPEMMILGGGPNAIHEYSDTDCCVIS

>Tgravida1 CAMPEP_0200700714
MSGEKRSSKKKTSVQSIRTHPPPPPPPRPNRQSSSSSGIGKEKSKRENGGSGRSVGTKVSKSTVRSSRTRESSRTNR
STRSKSRTRDAQSSRSRSKSRPRETPREGRSQSGRSTGSRSKSKTTGDRSRSRSRARPQDGSRSSPTKQQSKNSSNP
KSISSSKSKSQKNGSRSAPKKQTSTKKREKFASNAQQAIPASVRMISGSHDSQTSADVSNINSQFQLPNNAGRSGGA
CTAALLQVLYQSHEYGHDDISWVDVLRQMRDVLNEKGFEQIPQLTSSRMIDVQDRFAITPVDGSFSQKNNTKRAVLI
GINYTGQKGELSGCHNDVKNVARYLSEVQGFKKENVTILMDDGNHKPPTKTAIISAYKKLVKESKKGDVVFCHYSGH
GGRLPDDNGDEEDGYDETLIPVDFEKRGQIRDDDLLKILVSPMSEGVTMTCLMDCCHSGTVLDLPYRFTSDGDLEEM
EMNERCNFKDGLWSGMGLALGMAAGSAVVDVLTPPPEMMILGGGPNAIHEYSDSDCCVIS

>Tgravida2 CAMPEP_0200710618
MGFFNTLKNQAIAAAKEGMLSSLGVDADGSDGEQMTKSQYEESKPDELRKDIRMISGCQDKQTSADVSNVEQFELPD
PAGQAGGACTSTLLNILYADEKVPEEDLSFAEVVSTMREQLSEGSYTQVPQLSSMNPIDLNAKFDLVPDTATGTRRA
VMIGINYVGDRPGELSGCHNDVHNMKKYITDVHGFDEENIRILMDDGENEAPTRSNIIAAYKAVVDEAEEGDAIFLH
YSGHGTKLRDQNGDEADGYDEALCPRDFQQAGMIRDDDLYDILVKALPDGVHMVSLMDCCHSGSIMDLPYIFKGDGS
QTEMELDPEMNLDAFIEGISGKLVDFLKKSLGL

>Tminiscula1 CAMPEP_0201031444
MSYQDRIDAEIPADVHMISGCHDTQTSADANITQFELPDPAGRRGGACTAALLRVFYNDGDDDPQDFQGQSWVDVLR
RMRENLAAEGFSQVPQLSSSRMIDVESPMRILNSPSGTKRAVLIGINYVGHNPGELSGCHNDVRNITKYLKGGLGFE
GSNMRVLMDDGRHEQPTYRNIMRAFDWIVNVSQPGDTVWIHYSGHGGRLPDQDGDEEDGYDETLCPIDFNTAGQIRD
DDLLKHLVKPMREGVLMTCLMDCCHSGTVLDLPYNFIADGEHYGMERNENFDLSNLMDAAMGVAAAAAAAGVAGDIV
DECCVIL

>Tminiscula2 CAMPEP_0201033658
XAGGACTSAILQVIYKEEQPSCENLTFMDVFLQTREVIKSNGFEQIPQLSSSRCIDVNQPFDLMTNKYGNEGTRYAV
LIGINYTSHRRGRLRGCHNDVHNICKYIMDVGNVEESNITILLDDGTSTPPTRENIMQALDELTQKCQPGDTAFVHY
SGHGGRVKDETGEDPTGFNSTLVPLDFNKRGVGHILDKELYEHLVCAMPSGTSLTCLMDCCHSGTVLDLPYNFVADG
EQTEMTPVKDFPFVKLIMLRQALREAGVERLMDLRDSDKREQVKAAMAEKLEALARLGHNFDVRADFACGAKEEWRQ
ERHEAGGNVFGGVYVLCDCNX

>Tminiscula3 CAMPEP_0201021774

XELEGEATNTQYHQSIAPRIVTMGFFNKIKEAAIEKAKQEMLAQLGMSGDDEEGGQQMTTEEFERSKPDELRKDIRM
ISGCEDRQTSADVSNVSSFQLPDPAGRAGGACTSTLLKILYADERVPEEDLSFTEVLEQMRAHLSSGGYTQIPQLSS
MNPIDVSHKFDLVPDTATGTKRAVMIGINYVGDNPGELSGCWNDVLNMKKYIMDVHGFEEDDIVILMDDGEHIEPTA
ENIINAYKTVVAEAEEGDAIFLHYSGHGTKLKDDDHNEEADGYDEALVPRDYQSTGMIRDDDLYEILVKGCPDGVHM
ISLMDCCHSGTILDLPYIFKGDGSQTEMILDPELNLDSFIEMLTGKLMDFMMKKLGLGGD

>Tminiscula4 CAMPEP_0201050048
XSWARKGDSRSRNRSNHPAKIAVILLRRRDGATAVAAGGREIKEMVRVGQWGLATLHPCRDRLERARRRKFTIAPIT
IVGAEANPNRENRQETAALEANLDPEKRINPKTKTEAVAERIAGTEEGIGRQRIMTKLTNGETRVAAVANLDPDNPN
LNRKTKVTAMLAXSPQKQQSKKPSKHNNSNNTRSNSQRQHRSNGTRSSPAKPKKQPSSKKRQKFADAAQKAIPATVR
MISGSHDAQTSADVSNINSQFQLPSPAGKSGGACTAALLQVLYQHYENDEDEDDLSWVDVLRRMRNILDDKGFEQIP
QLTSSRFIDVRDPFVITPTDGSFNYKHNTQRAVLIGINYTGQSGELSGCHNDVHNVARYLTEVQGFRKENVTILMDD
GMHKAPTKSAILGAYKRLVKESREGDVVFCHYSGHGGRLPDDNGDEEDGYDETLIPVDYDKKGQIRDDDLLKILIHP
MPKGVTMTCLMDCCHSGTVLDLPYRFTADGDVEEMEMNERCDFKDALWSGFGLAVGMAAGNAVANAAVSALTPDPPA
PEMMILGGGPNAIHEYSDTDCCAIS

>Toceanica1 CAMPEP_0192891684
MGFFSSLKKAACDAAKQKIYEELGISQSDAQPQSGQMSREDYEANKPDELRKDVRMLSGCADHQTSADVSSVSKFKL
PDPAGSAGGACTSTLLKILYADEENPETQLSFTEVLETMREDLKGNRYSQIPQLSSMNPIDVSDTFDLVPPEATGTK
RAVMIGINYVGDSPGELSGCWNDVLNMKRYIMQVHGFDEENIVILMDDGEHTAPTFRNIIDAYKIVISQAEEGDSIF
LHYSGHGTKMKDDDGDEEDGYDEALCPRDYASAGLIRDDDLYDILVKELPDGVHMFSLMDCCHSGSIMDLPYVFKGG
IDSEMHLDPEINLDAFIQQISGKLKEYIERRLRERWG

>Toceanica2 CAMPEP_0192912886
XDDGALRTPHGGDDGKVLARAFYDRATDEEXPMSLAICEPYPAENKVKVLYSQKAGCYDVSKRLASQLLSSYRLTQA
LSIESFLLTGCRKMPDRQNSRRQKSTSGRRRKEDDAAKQKSTHHRRRSSSRKRQPSTSERRSKSRGRKKTQSRDTRK
SARTDVRSRSKSQPRTGVGSKSKSARTEKTKPQDASSSRSQKSRSGRNSQGGRKRSSGAKGRRAVGPNKTPSAKRRS
HFAEEANKAIPALVTMISGCHDAQTSADVANLSSQFKLPNPAGKSGGACTAALLEVLHCSSSDDEREMSWVDVLRRM
RIVLNKKGFDQCPQLTCSRMINVKDRFFITPPDFNEVNNTKRAVLIGINYTGTPGELRGCHNDCLNVARFLREQGFR
DENVTMLLDDNKHRPSTKAAILSAYKKLVRESKPGDVVFCHYSGHGGRLPDDNGDEDDGWDETLIPVDFKTAGQIRD
DDLFKFLVHPMPANVTMTCLMDCCHSGTVLDLPYRFTADGDLDEMQINDRCDFKSALYHGLVLGAGLAVGDVVASTV
ISGAVNAFTKPSDLDTAGMVLGGGHSMDSDMIVATSFEX

>Toceanica3 CAMPEP_0192912014
MSSFQSRIEEEIPADFHMISGCDDHQTSADVHNISSFQLPDPQGRAGGACTAALLQVLYRGDSPQDQSSSWVDVLRQ
MRRNLAAMGYEQHPQLSSSRLIDVNSPMRITNGHGGTRRAVLIGINYVGQQGQLSGCHNDVRNIAKYLSSMGFQQHN
MTILMDDGMHEEPTYRNIMEAFKWIVQESQPGDTVWIHYSGHGGSVEDDDGDEEDGRDETLIPLDFQRAGQIRDDDL
LKYLVKPMKRGVLVTCLMDCCHSGTVLDLPYNFYADGRQTSMRRNEDFDMGHLMEIAAFGVTMAMASDLAGMAAGAA
GFASAASGGGAEEILDECCVIL

>Trotula1 = throt11193 = GS1 CAMPEP_0192965622
MLRERARAAARQRMDDFVDETFGPPSPQADDRDVEITDDDAPTSDPEFDQRAEEAIPANVRMISGCHDTQTSADVSN
VDTFQLPDAAGMAGGACTSAILQVVYKEEEPSSEDLTFMDVFLQTRDVIESKGFDQIPQLSSSRCIDVQQPFDLMSN
EYGNEGTRYAVLIGINYTSHRRGRLRGCHNDVHNICKYIMDVGKVEESNITILLDDGEATEPTRANIMEALDELCQK
CEPDDTAFVHYSGHGGRVKDETGQDPTGFNSTLCPVDFDQPGVGQILDKELYEHLVCAMPAGTSLTCLMDCCHSGTV
LDLPYNFIADGEQTEMEPNQDFPFLKLLAMRQALREAGVETFRDLFDEEKREQLKASYDAAYNESMEGLGRDIGGGE
NFGRAARRERRQNRRANRRKR

>Trotula2 = throt1069 = GS4 CAMPEP_0192960494
MGFFNTLKNQAIAAAKEGMLSSLGVDADGTDGEQMTKSQYEESKPDELRKDIRMISGCQDKQTSADVSNVEQFELPD
PAGQAGGACTSTLLRILYADEKVPEEDLSFAEVVSTMREQLAEGNYTQVPQLSSMNPIDLNAKFDLVPESATGTRRA
VMIGINYVGDRPGELSGCHNDVHNMKKYIMDVHGFEEENIRTLMDDGENEAPTRSNIIAAYKAVIAEAEEGDAIFLH
YSGHGTKLRDQNGDEADGYDEALCPRDFQQAGMIRDDDLYDILVKGLPNGVHMVSLMDCCHSGSIMDLPYIFKGDGS
QTEMELDPEMNLDAFIECISGKLVEFLKKSLGL

>Trotula3 = throt10365 = GS3 CAMPEP_0192967966
MSYQDQIASQIPAEVRMLSGCHDTQTSADANITSFELPDPAGRRGGACTAALLQVLYDDRNGDPQDFQGQSWVDVLR
AMRNNLAAEGFSQVPQLSSSRMIEVNDPMTIINEGGGGTKWAVLIGINYVGQNGELSGCHNDVHNITKFLTNSQGFE
QENMMTLLDDGHHKEPTYRNIMDAFSWITRAAQPGDTVWIHYSGHGGRVEDQDGDEDDGYDETLIPVDFQSAGQIRD
DDLLKYLVKPMREGVLMTCLMDCCHSGTVLDLPYNFIADGEHLGMEQNEKFDLGGLMEVAAGVAAAAAAAGVADDII
DECCTIL

>Trotula4 = throt16314 = GS2 CAMPEP_0192949914
FVVPLVVVPPSHHPTATTFSNTNSNTSTANVPPINIIMSGEKRSSKKTSVQSKSIRDNPPPPPPPRPKRQSSSSSGI
GREKSKRENGGSGRSVGTKSIADTKVSKSTVDRHELVSRTAPIAAPAANPEREMPKAVAQGANLDPEKHRERAVPKV
AVPLAVGANPKLEEIGAAAVAEPGHKTAAXSSPKKQQSKKSSNSKNDSSTKSKSHKNGTRSSPTKQPSSRKREKFAS
NAQQAIPASVRMISGSHDSQTSADVSNINSQFQLPNNAGRSGGACTAALLQVLYQSHEYGHDDISWVDVLRQMRDVL
NEKGFEQIPQLTSSRMIDVHDRFAITPADGSFSQRNNTKRAVLIGINYTGQKGELSGCHNDVNNVARYLSEVQGFKK
ENVTILMDDGNHKPPTKSAIISAYKKLVKESKKGDVVFCHYSGHGGRLPDDNGDEEDGYDETLIPVDFEKRGQIRDD
DLLKILVGPMPEGVTMTCLMDCCHSGTVLDLPYRFTSDGDLEEMEMNERCNFKDGLWSGMGLALGMAAGSAVVDVLT
PQPEMMILGGGPNAIHEYSDSDCCVIS

>Throt14982
EERNRERRSRNGGEGSSHHQHGHNNNNNNNNNNSRSTNNRSDRDNGVGRSISPLHSSVGSGNTSGNASGSAFGSHSD
LSRVMPGGGATSPGLSSIASTKIADDTVQLDLPMADLMAYLQVVANNSSNLPLTRRDDPELGRTVSSLTAEEYAFKC
AAFVPSNVRILGGQFGKYGRVWDLPTSEEFDVTDGTREPGISHGGSCCNALLKAMYDTESEVNNIASPHTVDAKDLF
DDDDDETVDTAGYTVDRTIASFDTLVLNDGSNASAMSWAQLLRKMKPEMQGVGFNQVPAVTSSYKFDLNEPFSLVPP
DFKKGVNQKRALLIGCNYRRMPDAELKACHDDVRSVKDFLVNVYGFPESPELMTVLMDDKKHKHPTHSNMTQAFKKL
AERSQPGDAVFVLFTGHGCRVLDSPIDESAESYDEALVPADYEETGIIRDTLFFKTLLAPMKKGVTLTCIVDCCHTG
VMIDLPYLWTTKGDKGEVLPKMSLNNDFSFVRFLKVVKTLYESSVFTRIGKTVGTELDKQLPGRDDETVIETVGSLE
TMPENEQPEKKKTFFEKLCSPATLAQSIINCTLQAEDHQYSDDEATLGRNNTLDEEHSQYSYDS

>Throt6337
KIKEDHDSRQIIASNFSLLTSDPSETTNDRTAPTPTSAANDPLQSSSNFHLNPVSDISTMFGANGKKYADVIASKLE
KAVGDHLSSKAPQSTESATERMGKVTGKRRALFIGINYFGQKGELRGCINDVHNIQGFLTSNFRIDEVMVLTDDQKD
PRYIPTRQNILNAFKWLRNGAKAGDSLILHYSGHGGSVKDTDGDEEDGMDETLIPVDYQKAGQIVDDEVHTVLCRGL
PKGVRLTAIMDCCHSESMLDLPYIYTINGDLQIIETSKQEGIVTLIGAGTRFLLDGNKKNAVSNFKTGLGLLMSGGG
GGNSEAREKTIKTRSTEADVIQISGCRDSQTSADAQIAGQATGAMSHALISCLKKNKNQTYTQLLQSMRGTLEGKYT
QIPMMSAGRKLVMDHPFTI

>Throt7051
FTTLDYKSAGGSKVKISQHKMGFFKKTGALSSYPARRNRLQNEQNHQNPTAANPPSNYGAHNPTPMPASVYQTNGMS
VAPAAATTTTTAAVAAPYNAVAYGAFPGNNAPVQATVYIPGQGASTAPTPVSTAAPVQATAYVPGTVSSIPPAAATA
TAQVHTAAPVQATVYNPNNDSTPAANPSFLGAQQRASFNNGNGNYNANESSFWECSVCTFPNLRTEPNCKGCGAVIP

PGMLYSAASSATAQQPPTQQRPPQDAYNNITSQMNSMGLEGAKIGGAGTTSAGAPSSAANTGAGVMRVHIPLNMTTG
QKIKVRSPDGKEVVQTIPPQSQWHYDGTQPFFRMQFGGSPAPPTTAYGRDDGNNHNTNAPTMAHDVPPMHTTAWRDF
CHAPGAGHYSSPPLALQSVPSTPIGSGMPMRPNGRHKCLIIGINYTGSRAQLRGCINDAKNMQGLLRRNGYPDDGSH
MLLLTDERNRGREYQPNKEMITKAFAWLMKDVRKGDVLFFHFSGHGGQQRDTTGMEVDGYNETLIPVDYDRKGQISD
DVLWGSLVYRLPEGARITALMDMCHSGTGLDLPYDYNVDTRSWTEDINPAHSAGDVVLFSGCEDAQTSADVAGAGRQ
AGGAMTQAFMGAYNTCNNDAATYHEFLTAVKKELRKKKFSQRPQLTSSQRFDAKSRVFALGYASGGCGGGGGSIPSV
IEPNHNPKIGRMKRQNIRRQRHGLGGGGGNNFFGMMAGAAGAALFADALF

>Tweissflogii1010-1 CAMPEP_0193034156
MGFFNKLKNAASKAIKEDLLKSMGIDDADDGGMTRDEFEANKPDELRKDIRMISGCADRQTSADVSNVSSFQLPDPA
GRAGGACTSTLLKVLYADERVPEEDLSFTQVLEQMRRHLKEGGYSQIPQLTSMNPIDVEAKFDLVPETATGVRRAVM
IGINYVGDEPGELSGCWNDVLNMKKYIMDVHGFEEDNIVVLMDDGENTEPTRENIIEAYRTVVAHSEEGDAIFLHYS
GHGTKLRDDDSDEADGYDEALVPRDFQENGMIRDDDLYEILVKDLADGVHLVSLMDCCHSGTIMDLPYIFKGDGEMG
EMILDPDMNLDAFIEQITGKLIEFLQSRFS

>Tweissflogii1010-2 = 36-1 CAMNT_0037344015
IAHNILINRYFLIGVFQSLQTSFQRIMAGEPAKRSKSSRDDAPPKRSASSASHPKHSSSSSRQRSKSDADRHDKSSS
SKNGGDLKRSQSSRHGQPKSKHPKSKSPKPKPQDGGSKSKPSSNKKSTNKDSQKKSASASKKKSKPVENYGTKVVAT
NGSANKAAAAAMNEKKKAQKFNKDAQKAIPAQVRMISGSHDAQTSADVSNINSQFQLPDPAGKSGGACTASLLEVLY
DIHNNGLDGKVSWVDLLRDMRDVLEDKGYEQIPQLTSSRMIDVSHPFAITPSSFDSSKNKKRAVLIGINYTGQSGEL
SGCHNDVKNISKYLREVQGFKKENVTILMDDGQHKEPTKANIIASYKKLVKDCKSGDVVFCHYSGHGGRLPDDNGDE
DDGHDETLIPVDFEKNGQIRDDDLLNMLVNPMPKGVTMTCLMDCCHSGTVLDLPYRFTADGDLEEMEMNERVDFSDE
FWSGFFATAAVAAVASKVDPPAEMMILGGGPNAIHEYSDTACCVIA

>Tweissflogii1010-3 = 36-2 CAMNT_0037378133
YVLINYCSITFSNHIFQTLSTMSFADKVAESIPAEVRMISGCHDVQTSADANITAFELPDPAGRRGGACTAALLQVL
YDETNGAQDNSADMSWVEVLRAMRENLAAKGYSQVPQLSSSRIIDVNEKMDIVKDPEGTKRAVLIGINYVGQQGELS
GCHNDVKNINSYLQEVLGFERENMKVLMDDGMHEEPTFENIVRAFKWVVKESAPGDTVWIHYSGHGGRLEDQDGDED
DGYDETLIPIDFQSEGQIRDDDLLKYLVQPMCEGVTMTCLMDCCHSGTVLDLPYRFIADGDHVEMERNENFEFKDFL
TAAVGVAAVAADIAGAAGALDECCTIL

>Txantarctica1 CAMPEP_0200975774
MEEAIENAKKAIPAEVRMISGCRDEQTSADVSNVASFQLPDPDGSAGGACTSALLNVLYADHKKLEEDLSFQQVLLK
VRDVLKEKGFAQIPQLSSSRPIDIKNTFDLVPNHVTGTRRAVMIGINYVGDNPGELAGCHNDVGNMKDYIQDVHDFD
EENITLLMDDGEHISPTRDNILAAYRRIVEESQPGDAVFCHYSGHGGKLRDDNGDEADGYDETLVPVDYATEGQIRD
DLLFKNLVGGFREGVFCTFVMDCCHSGSVLDLPFQFKADGENDSMGVSSGFDFNSLLSMAASMNDTNMFADIASNPQ
KLLALCGSCTIS

>Txantarctica2 CAMPEP_0200957050
MSDDKCDDKLCGIDMDGICLPSKDKDNKLVQAEEGSCRQSPKSASDMEDEAETRDINETENEDTATEDKVRKDAATA
YSDNSGTDNKNRSIQADVFMLSGCEDSQTSADVSNVSSFQLPDPNGRAGGACTSALLKVLYSNQAAVTDLTFVKVLT
KMRTVLQSGGFSQNPQLTSSQEMDVNQQFYIVPPRCQGTKRAVLIGINYTGQSGELSGCQNDCINIKDYIMNVWGFE
EENIVILMDDGNHTNPTRSNILEAYKNLVTNSEDGDAAFCHYSGHGGRVRDDDRGEEADGYDETLIPVDYDTSGFIR
DDDLYSNLVCAMSQGVTLVSLMDCCHSATVLDLPFKYGADTTDTNQIGSFVTFGRGKIIIIICIIAVVITIVVLVAL
YLA

>Txantarctica3 CAMPEP_0200971514

MGRFFKKIFSGVREALSDKDGARNEFEDKKPDALRNEIRMISGCEDSQTSADVSNVSGSGFSLPDPAGRAGGACTST
LLSVLYADEQVPEDDLSFTQVLNAMRVNLAEQGFTQIPQLTSSNPIDVNADFDLVPANATGTRRAVMIGINYVGDDP
GELRGCHNDVLNMKKYIMHVHGFEEDNITILMDDGEHDSPTKENILEAYKKVIEETEESDAIFLHYSGHGTKVKDRD
GDEADGYDEALVPVDYQEEGLINDDDLYDILCKPLAKGATMVSLMDCCHSGTILDLPYIFKADGSMPEGMQLDESLD
FDGLIQSFGGHVIGFLTNFLK

>Txantarctica4 CAMPEP_0200971326
MSDFDAQVAKLIPAEVHMISGSADSQTSADVYNVSQFQLPDPQGKAGGACTSAILQVLYRDDQDYSNELTWVALLRK
MREVLNGMGYDQVPQLTSSRLIDVNEPMYIVKPGTDGGVKRAILVGINYTGQQGELSGCHNDVENISGYLQKVHGFP
PDQMLRFLDNGVDHSPTRANLMSAFERIAAYSEPGDTVFMHYSGHGSRVKDYNGDEDDGFDETLVPVDFESNGQIVD
DDLLDKFVKPLKSGVNMTCLMDCCHSGTVLDLPYRFTADGDVMVRDEGFGGLMDSPENVLALCCCLALLADFL

>Txantarctica5 CAMNT_0047144487
QPHNNNIITPTAPFNSTQTAAAPPTRPPPSLSNKSAWFDYYDRDYSGKLSRDEIIRGAVETLSVPYGSKKYNDISNT
IHSVWVVFDTDRSGSIERHEFCGPDGFGESLQQAMMTMNNSQSTTTTQQSKKKVRVGIPSGYGPGKQVMVPSGGKTE
IVTIPDRSEWIYLNTGQATFDIEIPTSSHYSAVQQQAKPQKPTTMTKTVRVGIPKGSNPGSKVNVPTSDGKTNIVTI
PDRSKWIYLNTGQPAFDIQVPTAQQPQAAHYSAGGGTDITTSASSYHTTSSSSASLPPWKNYNDMIPASYQPPPLGM
KSVSVSPQSNLSFVKTSSRRRALIIGINYTGDKRAALRGCINDAKNMKNLLLRNQFPNDGSHMVVLVDDSTSSNNHR
PTHSNIFKAMQWLMQGVRGGDVLFFHFSGHGAQVPDRTGHEADGLNETILPLDYKKGGQITDDEMWGSLVYPLPAGA
RLTALMDCCHSGTGLDLPFEYQKGKKTTNRYGMTSTSSSIRNANWIEDINPAHSQGDVVLFSGCQDDQTSADTFSTN
TEAGGAMTQSFISAFESNPYSTYPEFLSAIQRSLRQRRFSQIPQLTSSQAFSAEERIFSFVDGIAPNRNPHIGRIKN
KHVRPGRTSDRGRGIGP

>Txantarctica6 CAMNT_0047161225
FTLGGNNNKRALLVGINYIGQNGELRGCHNDVYNVKQYLVSYHGYRERDIQILVDDYNNRSIFPTRQKIISALQYLV
QSSVAGDAVYFHYSGHGGLLEPNAFRSSSKKDYDETLFPLDFDQSGQIRDYSLYQNFVQPMPAGVVVTCVMDCCHSG
SVLDLPYSFQPTHEVGDRSLIPMGTNTMDLSNLAFLYILMGGTLPVGGLFDSVTDTLQSSLGDGFLEDYQGTGMGNE
EMMDQFTEGGEDTAAPYGEDEDIARQGENFNYYDDNDGGGEILAPPADAQENFGYNNNNNYEENQNNYEENQNYYDV
GGDEGEEATNLPDCGSCIADVLNDLLSEGGEEY

>Hsinensis1 GAUA01013815.1
MSSFYENAEKTIAAQVRMISGCEDVQTSADVSNVSSFQLPDPAGRAGGACTSTLLKILYADKQAPDEDMSFQDLLLK
MREVLDEKGFTQIPQLSSSRPMDVATKFDLTPEDFSGVKRAVLIGINYVGQQGELAGCHNDVLNIKEYIMDVHGFEE
ENITVLMDDGVYSDPTRENILNAYKELVASSEAGDVAFIHYSGHGGKLRDDDGDEADGYDETLVPLDYMSAGQIRDD
DLLTTLVIPMAAGVFMTSIMDCCHSGTVLDLPYNFKADGEQTEMQESEGFDFSPMLALLLA

>Hsinensis3 GAUA01014143.1
MSNFDQKVEQMIPAEVRMISGCADSQTSADVSNVSNFKLPDPQGRAGGACTAALLQVLYRDESAPAQDLSFVSVLRQ
MRQILDAKGFSQVPQLTSSRIIDVQNKFTIVNPKEATMGGTRRAVLIGINYVGQQGQLSGCHNDVKNMKKYLMQVHG
FEEHNMTILMDDGYHREPTYRNIVNAYKDVVRQSLPGDTVFCHYSGHGGSVRDASGDEEDGYDETLIPVDFQTAGQI
LDDYLLENLVKPLSRDVLMTCLMDCCHSGTVLDLPYRFTADGD

>Hsinensis4 GAUA01006240.1
VGTQVADDQIHINLAMADLMAYLQVVASNSSNLPLTRRDDPELGRTVSTLTAEEYATKSAAFIPSDVRVIGGSFLKY
GRVWDLPTSEEFTVSDGAQEPGLSYGGACCNSFLKVLYDIENEESVIMGQSEYMDSNNLFDDDDENISVSNDSCTSL
DLGNDEGSGVTWTQLLRKMKEEMREVGFSQVPTITSSRKFDLNQPFSFFPENFDPTQNKKLSLLIGCNYNAVTDAQI
KASHDDVRSVKDYIVNVHGFPEKKGAMTILLDDKEHSPPTHSNIIQAFKHLAEESKPGDAVFIQFSGHGGRVLDTRL

DSEGECYDEVLIPSDFTTKGLIRDTLMFKTLLAPMRDGVTVTILLDCCDTGVMLDLPYAWTTKNDRLDIAPPKLSLN
DNFSFVRFLKVVKTMYEMSTFTQLGRTVGSALN

>Fsolaris1 Scaf102 GAX16243.1
MGFLSRALKETFEQEKPQQLRGDIRMISGCLDSQTSADVSNVQSFQLPDPAGEAGGACTSALLNIVYKDHHNTAADL
SFTEVLTQMRSMLKAKRYSQVPQMSASREIDVNTKFDLVPDNATGTKRAVLVGINYIGHSQGVLSGCHNDVKNMVDY
IKDVHGFEEQNITVIMDDGEHPEPTKANIVAAYKKLIEESQPGDCLFCHYSGHGAKIKDDEQGEEKDGYDEVLVPVD
YEQAGMIRDDDLFDILIRPMQQGVSLFCLMDCCHSGTVMDLPYIFKADGIFENMEIDENFDFEKLFKKVGEVAVREL
GNYVKNKYLKDVL

>Fsolaris2 Scaf183 GAX22319.1
MTDYAERIEHAIPAEFHMISGSHDMQTSADVFNTSQFELPNPAGKAGGACTSALLQVLYKDNHAASSQMSWVECLRK
MRSELNRMGYDQIPQLTSSRLIDVNKPMTIVPPGSTGHRRAILIGINYIGQKGQLSGCHNDAMNIKKYLINVHGFKE
SEMLILMDDNKHHPPTRRNIEDAFQRITQYSQAGDVVFVHYSGHGSRIPDLDGDESDGYDETLVPVDFKSAGQIVDD
EILKLLVKPMKAGVTCTVLMDCCHSGTVLDLPYRFSADDSQMRLDPRMNLEKYLGKFDVQTVLCLALLACCLADLLN

>Fsolaris3 Scaf132 GAX18784.1
MRGNPTISDREFDQRVKQVIRSHFHMISSSADHQQSEEAYHAGKFQLPNPAGKAGGACTSAFLQAMYQRGEGANWVE
TLQEMHDILQGMGYAQTPQLTSSRLIDVRKPLQIVPAGSGRRRALLIGINYVGQEGQLTACHNDVHNIREFLTEVYG
FKESEMLILMDDARHNPPTRRNIEDAMIRLTRYSQPNDVAFVSFSGHGGNTRDTSGDEADGMDETLIPVDFRTEGHI
VDDDILRLLVKPMMTDVHVTVLMDCCHSGTVLDLPYKFGANDTRVQRESGFNLDQVHESQLTVGSYHAPGTVLSSWS
LHGDKEKPPKEEKKSPKKSKKKKKAATFQPKKKKNKDMTKVEENEQQEVGPKMVGGQAALPVRRASKKSAAPAPGDK
KCSVM

>Fsolaris4 Scaf78 GAX14314.1
MGNSDSKPEVIEIPLPGAVETLQQGDQGEVDHIDPIEASTSRNESRDERDTQDNELPVTKIRDDQVHVNLAMADLMA
YLQVVANNSNQLPVTRRDDPELDRIVSSLSSEEYARKSAAFLPADIRVIGGTFTRYGRVWDLPTSEEYTATDGALEP
GRSYGGACCNTLLKVLYDSANDAAGAAQSEAAAESLFDDDDDETDTDPLPKSPKSGISLDFGGQSSVSINWIDLLRR
MKVEFKEIGYAQAPKVTTSRKIDLNKPFSLTPENFNPEKNKKRSLLVGCNYHELKDAELKASHDDIRSMKDYIVNVH
NFPEGKEYMTVLLDDGEHPPPTFMNIVEAFKALSEESQPGDVVFVQFSGHGGRVLGDPSDQVGTYDEVVVPSDYKSS
GLIRDTLMFKTLLAPMRYGVTVTIIIDCCDNGMVLELPYCWGTMHDKKESIAKVSMNKDFSFVRFLKVVKTLYESSV
FTQLGKTVGSALNPLSPLEDVVSSRREKEKNSEKRNTKASKERGDTIFDALAHACTTVRFDDRVICRTGKTDNSVEE
ESKKASSDAGSLIEKVLNCTMMERDEDFSDDDSFKSNSYDVGSNSFDSLTEEDEPHRSHRRRRR

>symbA1-2 GAKY01119793,GAKY01119787,GAKY01119790
MGNAGCCSSSDGNPQFEILAAPRDIVGRKKALLVGLNYRNTRAELHGCINDVNNMQQVLVKQYGFKLEDILMLNEDQ
DKSQWPYKKVIIEGLQWLYKDAKAGDLLFFHYSGHGSQYQLDKKGMPADCICPLDCLDKKWPEAVILDTEIHQELYD
PLPKGCKAVCIFDCCHSATVANLCETMVVQEGPMTAARKEKMVKALTDQKNKAAATVRFYQDINSGKLDL
>symbA1-5
GAKY01160523,GAKY01160524,GAKY01160525,GAKY01160526,GAKY01133546,GAKY1133547,GAKY01133551,GAKY1133554
MLAPPERDEFGKLPLEDTTEAPPEDDDGLLAPEETDGDHLDEIPEAPREVGSYQHQQHHYSGHHQYGHHAPYVSQLA
SYGSHGHHGPYALASYGSPGMYGQGADGSYGSYGMYSADGFQQLYQPYSGYAYHLSGLGMAPMAPQPSQHVQISSGQ
AGQMVTSPSHQAAPSKPAKPETAAKLHVTGQRRAVTVGVNYIGTENELRGCINDSDTFITLLTEDFGFSVSDIRQLR
DDHPQRMPTRKNISAALQWLVKGAAAGDHLLFHYSGHGSQRRDSSGDEADGKDETIVPCDFNRSGMIADDELRRMLV
DGLPKGCRLTVIMDCCHSGTGMDLSYKAKVLHDGSLTIRAVSSRQRHPTGAEVVLLSGCRDVQTSADISGGVAGNKA
AGAMTTAFRKVISKKRDISYHHLLLEMRSFLKQHSFDQVPQLCMEFHLNFEEPFLPEADPPVAQPPAPLRSTQRRAL
TIGINYFSLTPGQGRLSGCINDSETMIAVLKETFKFEDGQICRLRDDRTNLMPTKANILSAMRWLTQGVGSGDELFL
HYSGHGGRAADRSGDEITGQDDTLIPCDFQTAGQISDDELHSLLVEHLPEACRLWVLFDCCHSGTALDLAFKVTASA

ALXLDSRRLRCTKMRSRHRRPSGRPATPANGEVVMISGCKDDQTSADIQSGSSAHKAAGAMTTAFRHVVSPTISCED
LLLQMRQFLRRNSFAQVPQMSSDKYLQLDSAFTNYQTKRXXXXXXXXXXGRCAYGISATACRQRDGRGHTHSSTRGT
DSRTAAQVAFANGACQSAVSCRMQHGDAATVAPSKSRRAASGLDESKLVRPLTSSSSEHLAVQRDRKS

>symbA1-6
GAKY01019324,GAKY01019325,GAKY01019326,GAKY01019327,GAKY01019328,GAKY01019329,GAKY01019330,GAKY01019331,GAKY01019332
SSDLAPLAASAMGKVCSVLSGRPAPADICAVIKEAEHRAEEWDCWAFGDSRDVSPKAAKQEPLTGARRSLLIGCNYA
GTSNELHGCANDVRRMIPVLAKLGFPSDGGSQKVLLDEEGVDGPKPTHANMLEGLDWLVAEAQPGDALLHYSGHGGR
EPAEEGGYHETLVPLDFETAGMLRDTELFERLVKRLPEGCRLTCILDSCHSAGALNLPYIFVGTEEELRKAVAGEAV
RMALAMRWKSDLEKWRQGSSKELFGDVSSLGKNLWKMYQDGQSGYVTDKETTKGVAVGEVVAITGCRSDQTSADVGD
VSTFGLKQVSGSAGGALTSALVEALEGQEELTYAELLERIRQELARKGFSQVPQF

>symbA1-8 GAKY01116216
MANFMDELATGSRRTWRDLCKKETPTVGSDGMLKGIWREAVIAAVACEKAGKAGRFADGPAGQVLDYLLERGFNKFF
TNAEQTEEAAHAGRIRPSMAPSGAGAAIDSWSGGGKPHEYRPGKDDGKKPGSGDLQIRCGNCGNVYGVAVPPGTAPG
ATVQARCPHCGTTNQAAMPHGGGSPSSPMKFTGGGAPPRPSGRQKALLIGVNYFGTRAELRGCINDVHNLFRLLTET
YGWQAHNIRTLTDDGRGGGMPTRHNITQHLRWLAEDAMPGDVLFFSFSGHGAQKEDPQGFEEDGMNETILPVDFEHA
GMMTDDEVSDYIVKPLPEGVRLTSVMDCCHSGSGFDLPFTWDPRRGMWREAVNPFLCRGDVLMFSGCEDDDTSSDAA
SMYAAPGGAMTTAFCDVLRRNPRPIYPELLQLLHRHLSMGGFSQRPVLSSSQQFSLDRPFSFDDIHPNMNSQLGRIF
RQRFPPRPRPMSGPLADMLGPLGMLAGGLVVGALAGEALEGGVGLLGALFG
>symbA1-9 GAKY01183850,GAKY01183751,GAKY01183753
KRLKPSVWARETMGAGDSKCNCGLCHCCGDGPLEPWRDSGGCIRMLIIALDYDYAPSAELTCTKDARTMYRMAGRAG
VDDITVITDKAGVGDPSFPTRSFVLRHMRQVAKRCEEGDWFVWFWAGHGVNVPDFNGDEKDGFDQAFVTPDVTGRLT
ESAVLIDDEFAMALDTFVPDGVRILCINDCCHSGTICDIDSFMYKHDIYSISASQDNEEAEDMGEGGVLSTALRRAV
RTLSVEYGSQEFSIMDVFQRCKRFAVRLTGEQNINLQYSGPKPSVVAWPLCFPWWTYLQKAGLMKVDIQEFAEDGLD
SDEDWTVPVAGSPATTSPFALTPGAGVRTNG

>symbA1-10 GAKY01049302,GAKY01049303,GAKY01049304,GAKY01049305
MGKRALCVGVNYPGQEYQLYGCVNDCLDWERMLKEAYEFEETRVLIDQYPDGTPTESGAQLPTRANILAQLGGWLVA
GAQPGDVLVFVFAGHGCQVRTNERELEEALVPGDYRDSSALILRDEVHALMARLPSGCYITMILDCCHGAHMLDVPC
SVDTSTGPFTVHQTTARPQEVSRTHEAWMQAYVDHARSRPRFIPTVTASGRTKRSPEGAGAHVGRMTLNPGVTAFCL
AAARPFENARDANIKTYQCGVLTFCIHQALQDLQYRCTFEQLLERAAWKMEDIRNKYMRMMDQTIQMSFCPNSAPSA
VVVFDARYAPVAQHRISQLAQQQNMSPPENLRSPGAPSPEPVRQASTEFVPSPGYREQPDTNAVGGEVVGVGIVYVQ
LFSCSDLRPQGMYGQCDPYVKMTCGGVTHQSTVKRNSTHPTWGDEENKFTFKASDCQVEMLFIEVRNAGRDELIGRV
DVPLRSLPSMTWEERRQFLQNGGEVEFRVALYPERAAANLRPQPGLGMGLGGPMGPMEQQPPPAGTGYPEKPRPPSP
PARDLGPSLMGEYDLRAPMERRPMDEEPLLENIFGKPNLFAAMPDLMSQLSTNAPTGFHEVLPAIPQVGGLSTPALG
GLGTPVSGLPAPATAMPAPAAPATSRLGVASYTPPPAACFTPPSLLPTAEVGRTASRGLSLEGYYSTGGMAAAKMNT
ASTAVPTPQVPGSYTYSTPSPFTYSQAQPGQSALGSNLNAASYPYSSSGTPFTGYSQQIISGQTSQSALGTDLASYT
YTAPSYTTSAAPASLTSYTPQGQIIGTTAQKALNADLSAGAYTYTSAPTALFTGYSQPGQSTGAYGARLATPSYQPP
YQVLS

>symbA1-11 GAKY01048077,GAKY01048080
MSGWSPAAPRPFSPAAAGAAAASGASGASAMGSWRFGPEVRHVEAPASEKVNQLRRSFTSLQAEMQLSFQRQDGEVA
SEPFGAFLPNSGREGVLAPVAGGSMALGSSSSLASTVPGGAADVSPSNAKSETGELDVWLKEKAQKIKDHVTEMANA
QRRAEDVGRSLQDHLDQLAELRRMLRQAQADLAEELAKAQELERRRDALRQEQADLQSQIVLREQEFQELRSNRDDV
DRQLSRALAEQGLQKNNLEASSKSFQGQAEILAKDIDQSKRQVLELRSSLAGRKAVLEEKEKSLKMEETVRVEKEMV
QREQLSAELDQTRTSLAEAKAVMEVSKRRFDEMEEKGRETTKRLEMAREELHGLRAEDGESPVRSTVEDAKAILAQK
TAKLNQQTLQVEKLEKELQDNREQLEKQSLRMAEVHAEATEFEMKSKCLDERKLQLQQDLEDKRLLVQQSLAEKDEQ

QRRLAEARESCARDLAVLKDEEQRVAEARTRYAQTADELERDVAAKQHELQTAKSHAEEIQKVCSELEKTRSSRRSL
LQDRTAALQAAQARAQDLEEELAGTEAELATAATELQRLEKEEVERATEIEDARQKLSEVQMEIETMQARAQTSRVD
DLEATSRALAESLQKAGQERTAAREKQDALKVELHESESDLKKAEAEVASLTGELEKAQSEAESLTEQVNNLLQSSS
GDKSKAETLQKSVEANNKELDATRGLLVTRQLEVEALHQVLDNENQSHRQELEALEAAAEASTQSSQELAQQLRELE
RRRDEAADALAAAESHKLAVEQEIQKVIAETERVTKETYTSLSLLKAQTPQTYAQGAEQKPDDPEAVKLRQLKRQVE
AEAQRVANLRSDLAAREVEVERQASIAAALAPAGGVSELEGSLQQMNMELVRAQESARKSRRQEEDLRNDLAKLKER
TAATRDKLLYQEDHLKLQGETLAKLESQKAKSEGDVETLTLGLHKLDQENQYLQEAIKHLMQQVEAKHGGSLDGWAG
ESCEAQVAREVARYVAEIEQWKRQTEQVRRQKTGEVTRQYQEHEVTVQTLNQRIDKLQKEIIQCDRCAKSWEEEKSR
RTNSAPTSPTVLPGLVIFQRPLPSHHERRKALLIGVNYSNSHAPLKGCINDVWNMQCLLRYTLRYTPEQLQLLVDGA
DGRSRPDRAPTKANILAGLQWLIEDVRPGDHLLLVFSGYGAQHPRTPGSNKCESYLVPSDFAADLPSDFFEVVASAC
KSPAQPNVIDQTAKMAAGAAPGGPEEIERQAAAAQKALGNSKASYRLISMLEVHDFLSQLPQRCRVSLLLDACYTIL
PGAGPESNSPATFRKVDRGRVEYDKLRNFMSRPRFLELPPLPVQHTPPHLPRSNSFLPCTLHCFSGCRLKEWCAEFP
IEGSVQGAFSWAFLKALAQGHFHIGVYQFQQLVSNILLNLKGYFRHIDQQPVLQLSAAASPQDVVLWT

>symbA1-12 GAKY01032711
MGSVGSQICCFLRRACDPEQRVEMRSLAAQPVPPTPQPLTGYAPYAGPGQLATGVAVATPIPYASAAPVVACATPVE
MSPVCHNGFSCTPHAESIPVTGMAVSSRSVPQTLSQAQVQLPGRRKSLLVGINYFGTEAELSGCIADVKRMQPFLEQ
LGFPSDQQCQMVLLDAPGWPHYRRPTLANMRQAIRWLVHDVRTGDALFFHYSGHGGREPSTSGADGYVETLCPEDYD
EEGMLLDTELFETLVRPLPSGCRLTCLMDCCHSGGVLNLPYLFTGTETHWDFWKMPLAWAWGCGAFGRNTRPPRART
KRGSLPTKLAMSVSLWARLWQSPAAVLIRRVQMWVTSKRSSTSSRPGAGRC

>symbA1-13 GAKY01017080
LGVVHEVLSRSVRAMGNTGCCASSDGKPIYEALSAGPPGGRKKALLVGINYRNMGSELRGCINDTINMQEMLVKQYG
FKLEDIRMINEDQEKSSWPFKKVIFEGMEWLYQDARSGDTLVFHYSGHGSQYSADEKAMPADCICPLDLIIDRKWPD
SVILDTEIHEKLYDPLPPGCKAICVFDCCHSATVANLCETMVVKEGPITASKKEKLIKELTRQKDNVAAEVRFYREV
NSGQLDIKKKSREEMIQLLEKHAFPKRGPYGPYSDVSTLNYNYLLSTSISAFFEDSVKAAEARLAAKEKAIAKVQSM
QPGEGAEIYIGNLEQDQQTAYGKREAAKDIRLRFIPQPGMDSGDSETAKPKLLGAGRGYRDVLGQAKYKDHQLWVFS
GCQDEETSEDAHVDGTFQGAFTWALIKALKSDGFREAYSKLLIQIKTYLEGGGFKQVPALSTTHKMYLDCGFLGEEL

>symbA1-14 GAKY01067445
MELLRCVAEICCGESPQKPRPLVQPGPGVTGAPAPPSQVALTGRRRSLLVGINYFGTQNELHGCVSDVQRMLPLLDK
LGFPSDAESRRVLVDAPDWPQHLHPTLANMRQGIAWLTQDAQPGDSLLFHYSGHGGRMPRSDGRSEWHETLCPVDMD
AEGMLLDSELFETLVRPLPSGCRLTCILDSCHSGGVLDLPFIFVGTQENLASALAGEAAQMVMSKNWLQDWQAWQES
DDPAMLLSDAASMGMDLWSLWGKYSETREANEQGFRTDEAENEGVAVGEVVAFTGCASEQTSADVGDVGAQFQLQPT
LGAANAGGHLLLEGAGRAGGALTSAFIEAMQTEGEETYVQLLEHLRQRLDSAGFSQVPQLASSLVLDLNTPFSVDKI
TPPPQPGQMPSKSMDRDMPGYGGYGCTDQNQGSVFSDMLTGVQNMLMAEEATELMEEAGDLLMDNVAPDEDF

>symbA1-15 GAKY01124971
HISGASLAQAAGSEASGSDIRRVAMGAANDKLASADDRFWQASAKDAPEMWSHVPATYVERVVPEQESLVDLQLPPC
KVISHTVQMAADLEHRPGMLRTEARPAESAGSFNVGGTSNAKDLTPVQISRPLADTPQVHSQGFEPQSALTQKSEQP
SHWTCSACEEPNKAVREQCNNCNAPRPPGSSATEVDTKAAEPGRKKCLLIGINYYGSQNELHGCVDDVHRMLPVLEQ
LGFPHAEDDSSCRILLDSPEWRMDRRPTLANIRAGISWLVAGAQAGDTFFLHYSGHGGRMPRADGRGDWHETLCPVD
MDEAGMLLDSELFASLVQPLPSGCRLTCILDACHSAGALDLPYIFMGTPENIKKALAGEAVQMAMSKSWLRDLERWE
VAEDPTAFLADVASMGMGLWDMWRRYKTATSSNQNGFCADEPNNIGRAVGEVIAITGCASDQTSADVGDVHSEFDLK
PSQGGGHLRVSRTSAGGALTSAFIEAMDASQGRVTYLNLLEHLRGRLADAGYSQVPQLASSLLLDLKQCFSLATSAQ
QGSTSAEDAPTWQGQRSGMGGMGGALGGVGAGGCGGGPWTSHPAPGCYPDSALPNAGVVEPANTAPGLFSFFGGNQA
PYRDEATTGYPTGQTDDDPDACGEDDEYGSENEADDYFD

>symbA1-16 GAKY01140159
MQLEITVKQAVGLKHDGRHCVEFLLDDHAPQRTRWVDDLYDTQGLVVQPKNFQSSRCTITLKADKSAYKDSFLHLTL
FHQRNVRDDKRAAEAHLPFSGMANRFKGWVSLEHKDKYGGKLYVEANLKEPAPAPPAPAPPRPKAAAPVLDDSELAE
QQAILASIQASQRRSQTGQALPARQSSQNSIQSQSQSPSASPSWPWPDSTSTSQAARPPSWPDWPGTAAPAALPPVR
EPVEADPVLPDWLPWPRFKAALQAGRLEALSRSLEPQRAFPSGEPKATGRRKALLIGLNYPGTSAQLRNGINDVERV
AAVLGRLGFPEEWMLKLTDDAPRHGAPEVAPTKSNCIAAMQWLTHNVAPGDVLFFHFSGHATQDDAEGSDALCPSDF
MDAGFISSEKVHETLMRGLPAHVRLTMLLDCCIPCLCPELPLLYDLEQDRWVVGSSASSSADVVCFSAAGSEMTLEE
LNQLRMAEHGFVTSAFLQAMQQLAQQRKGPVTYQELVSEASQHLRPLAHRALQLSVTQMFDPNVRTFRFFDAIKSKV
R

>PfMC1 XP_001350260.1 PF13_0289
MEKIYVKIYELSGLEDKDNFSCYIKIYWQNKKYKSCILQKNPYKFNEIFLLPIDIKNNVKDEKNNILSIEVWSSGIL
NNNKIAYTFFELDHIRRERISSEKINLIDVVKKCTLQISVHIINNNQDILFCNIKDIFGNNKNDKEIHDAILKYGGN
ERHIIKELRKEKEIGQYNNIYFNDYVNVLNTDPSQNYIYNDMPKITPNNIYNNMNNDQTNHTYLKAPNSLYNNENTI
YSSNVHYSTYMNNSPTYKNSNNMNHVTNMYASNDLHNSNHFKPHSNAYSTINYDNNNYIYPQNHTNIYNRASPGSDQ
TLYFSPCNQKKALLIGINYYGTKYELNGCTNDTLRMKDLLVTKYKFYDSSNNIVRLIDNEANPNYRPTRRNILSALM
WLTRDNKPGDILFFLFSGHGSQEKDHNHIEKDGYNESILPSDFETEGVIIDDELHKYLIQPLNEGVKLIAVVDSCNS
GSSIDLAYKYKLKSKKWKEDKNPFHVICDVTQFSGCKDKEVSYEVNTGQIAPGGSLVTAMVQILKNNMNTPSIITYE
YLLHNIHAHVKQHSNQTVTFMSSQKFNMNRLFDFEHIIKNKNNQLGQIINKYIEKNKSKNKNKLKHELKNLFFF

>PfMC2 XP_001348537.2 PF14_0363
MNSMNTYSPLYSSYENINIYKKNRKFEEDNYETSTANISCDSKGRILVKESENDRNESIQKKQMLSSMDNRKTNKSE
KHNISSYYKMRKKASNHLKDKEKLNNNIIYAKNPHANRYDKYYSLNMNIMNNNMNNMNNMNNVNNMNNVNNMNNVNNM
NNMNNVNNMNNMNNVNNMNNVKSMYNNNNNSNVYYRISRQNINGKERLLNRYNIMRPQRSADMLIRNYLYNDLPYEI
NQQNLNKNINDNNAGSFDNLNKTVFENYNKNILDEYNRNIMQYYNSNLKNYRNSSFDNIYTRYNNNNNNNNVYSNNN
VYSNNNIYNNNNNNDNINNSFQFSKQEGLMKIYSNNNRNMNDYVVKTSNITRAADNTCAQEQINIPTYNKTVLLKSS
PNTENTFNKTLSNILKEQPKRSSMFSFNSSDFFKDFGKLRKNFVGKKKNKINKDIVINGNIFNNKIIDNNTSNAKSE
GNSTLHKIYNFIFPSSVKKLQKKKQKLQVIEDKEKNSNRAVVMPLKNKIIYINDANITNDKFKHSFENYNYHQTNIP
FVSVTNYMNKDSTKNVRSFSSFDVKERISPRVIRNGNINNNKNNNINNNNNNINNNNNNINNNNNNINNNNNINNNN
NINNNNNINNNNNINNNNINNINNINNDFVNDSNINKTVNIHNNNNNLNNYSNTMKSVAKNTQKLSAQKNSLVNDKH
HTYHEKLITNIYQFTGGQSQKSCNIKNQNVKEEKINNTQISYINNTAYNDYTNLPNVETYVLSEKSNNLENKEDNNI
NEKFKNDNNVDKKNNDENNIIKHNVNQNNNEVIVMNNQTEEQINDNKFIKPLNNITGIDPKKKNIYNTVMHINDKMD
LNKHDYNYNYNNIIPHNYINSSDKMTNNSLHINKKADDIFQNMISNNDTNVPCVYYVNKYINNIFQNNNPSNVTNYIN
TINMNKAPITIDENSNKLDNNINYENVTKNMIYNNYTNNNVLYITKEKSDNKLNNSTNHLNDLKNNYYVINPSLLYI
HNNNNNIEWNKQYTNNFSKPNFLDKFFYEKKVESIQPHHLNQYYYNNQNVKYNPSHINILENKYVNQFTNQNENSLY
NFTTHHNNIKNNNIDNILLLDKAIRNQSIYYNNNKGDDETKSAEHTTNPSINLLKNNYEDKNKSNYVHSIVSITDKD
NSIYAKNKYAEVVTKNNEKREDAIHKKDEQYIFNNNDNNNDNIHNNNIHNNIHNNIHNNIHNNIHNNIHNNIHNNIH
NNIHNNIHNNIHNNIDVSSRENFFQHDIQNTNQINNKTILDITQNEKPNATNEKCLDINNKEKKKKKIKSVHYDMDE
ILSLKCKVYDELDTCLKCINNLTKNKKLYEQLKKYKHKLDKDTKTKKLYDECTSEKTGSYYTMKDSYISSLLCNEYK
LNHILNKSNNKVSDDKHILSYNHHHNKNNNINDINNIKHISKFVNKDNEKTLNNINILNHNNKDPNSGVFLPSDNTE
SVIYNMNRNRKLCDNNFNVSKHVLYKDSKGTDSTRTNFYDDYSMSYVKSNVDNVEKKEKIKNNINISDKYFLSETDS
PYIGKKKALMITLNYNGLLEGCVNDTVDMCDHLMQRFGFNDFILLNDCNLCYRNFVTQKANKKNILSNLHNFIVNSN
NGDILFFYFCGYSIKLIDSKFTENYNFALLPQDHSKNNYIYSNEIFNIIKKLQGGKQLCIIFDTTYTSYFVPVPTSI
TYNKNMNTTEIYKYNNFSSNQKYLKSLKTFGKIRDRNVDSIFVENIKKPLLYEIYKKENDTNTNDKIILVPSIFFFS
PDCNDRNDFEFSIKNKVRGLLTYCLGKAIELLKNDFSYHDLFVAASQILIDIKKEYNLKYVKFKLSFLNEYSPDDIK
FLSHESLFLKKKLQLDEPLWKPSLKLNNLNQYIQDICNMDERKMLKSSKKKCLLIFIKDIKFYTYKNIDTKNEYFVS

CFIKNKNVNILCVRRNNTKEQRIVQDKIFFLEYITLNVTHMENANIYVELFKKKKKNYFVARSIFNIRNVNGKFSLS
DEKKNIIGIIDLNIKCVS

>PfMC3 XP_001348333.1 PF14_0160
MVYNNGSLRGRKKIEEKYIPMNRKSTSLKDPSRTKNNNYIKTLDTYINEEDSPTKILSKGKKNKNENIKKRINEKDN
DTDREDAASLNVDYNKNVMKKYNTRGTFIEKVSSSERNIEHNNNIIRKLGSKDYSKARVQTMTTNIGNHNALKKINP
KVPSITSRTKSYTKVMNENKYNNNNSNNSNNNNINSMYYSKKLNNKKYSTEHTQSIDNNNITSKRLNNKKTATDKAQ
STSRYCISNSVKKSYSTKGYNTDRSQTMSRYTLQNKKDSNTNTNRKGYSTHRGQIIQDNSNINNQAEKKKRNMSLKK
KYIKESSNSTITGSNEKKNSNNILKKNSTCDLNYLNKEYNNNNNMHEHAENVYNINDNEGCSSMQIINDEDVQKNVP
EFYTINLGSETKNMGENYIFKNRDKKIKPNIYNERNNMDLMEHKLNDDMSYNFADHIKDIECYINQLNESNKCNIYK
ECDPSKLISPVYDNVNDNILENNQQLNEYEKHNDILTTSYLKYYSHTNNNNHYYNDEDVHLDLGKTNEGFSNVVKNM
DNIPWNETNKKESFSNIKKKIDHTDDRHIAYVDNSSMKHTMNDKDQYVKNVPYNNTMEEVLLLNEKRGNILGSLISK
NHDAKRATSNNNNINNNKYYYYDNKYSEHGGDSVSSNIGYPHGYKNNNIYNYVNNIKDLSSPLYCNTKTKRAHKFHT
DNIMSDQLNKYEDNNKNDGKFFSGNISSKQKGKNNLTNYTTHSSEYRIKSIESNLTKDYRKSLTYNDIPNFLDYNIN
NTNVQNSDFGDSHYINRNVSYKNSMNSNDDIQNSNNNNNNNNNNNNMVNNPNEYLVYTNEKHYVRINNKLYEKTRDNT
YIEVDSNSNFDIINDINSSHNSPLDNIKDNTLYHNTNKKEYSDYVSSYSNINKYEDINLPNNIHVSKDGKYLFKNYS
VSQNVSDDNSVQDSFFSRTERSNSQNRYDKNGVNHIEQNYYNNSNRRDDIYYYHNQLKKLERLTQQNIKSGKYQRNI
PQNDDNDHNDDDSEIKEVKKDKTKIDMNKHLHLNDKKIFNQVKRENTGVHMNASKTIKNMKEVTYRNINNDTNYNNN
NVKINYLYSDELGKNKNRDTPLKTKQHGDSLDNKTELKKKIYNDIINNDIINNNIINNDIIKSGAINNNIVNNNLIN
NYNADNRNVSNSIFTQHINNINNNFYNSDDERINIIDNNKRMDMTKDESDRFRNNYFVELEKRKKNLGKTNNTTPIS
LSKNETINNMMINKRNFFNNYISDKVRDKILKREISCSVNTVCSSKTNNKTNSNYSSNNNNDNNNNNNNSNPFILDN
SIRKSNIIITTSNNAKLINNTIISKGINNNILNINSNKNKSLNSAKINALGLLSSRNDLNYIMNNNRTSNNHINQHL
LYNNRNYSVIKIPNVHKEKRSSKIPHNINIKSNLNLEPNIKTYTNIKTPINIKAYTNIKAPTNIKAPINIKASTNIK
APTNVKAPTNVKAPTNVKAPTNVNAPTNVNAPTNANAPTYVNTPTNIKRQEFSNVTLNYKGISKFDINEDKNTTIIP
PLHRQSNSNFPFKNTKLNLNSDMYTEKELIKCKSINNSDIYNYLRNTYNKTPSKFYRTVSYRNMSLKKNKSMLENRK
RKTEKKEENLMSTFNYTSEVDSKYIVKKAVVVGCNYVSEERSRLYGSVNDAYVFCRALVKYFDFLPENILLLTDSLP
SNAYIYEDFDINRKKYINVDEEENIKNNEPLKKKNIFNLFNTNALYTTLKKTNEEELNCNSCKDVEIKNVDISSEKM
NFNLWPTRVNILKAVNWLVRDSIPFGSYVFYFAGKSVQVDNMSGWEGEGYDEAFLCSDPFNKISEHNVITAVQLKDL
LLSINESAQMTIILDCSGGQTILDPAGTENSLSYIKGCKQKGIWPITNPTNKVHKAIYDITILNNTSMKKYFCRSRY
SKLIEVESTSAMIDPLLQSISSLPVAPKAYCLCAATWEQISIEGLFPIIEFARVSQLKKPESYKTGEGHYQNMNKKN
IRAEKSNRNGPNGLINKNNNLSKKTNYEKNFNFTLNMMKMLFSNTNNNENKLDEKEKINRLGFNENDSDYIDDNYNS
DDNNNNNYYYNYDGEKGFKNFQENLNNVGQKDVIKNKKFKDNYILVSHGVFTYCLIEAIIEFKEKELKYNILEKKNE
QFIPMTLKNLINVIQQKMQNIKYNKLKKINQKPEFTIHPGANATNNNYFVHYSKNIHFQNYKCNFINADLSPFLNVN
KAWEEINRTTLRNRKSLSLSSTLINTASSKYFTQKNEQFKNSYSLKY
>GtMC1 jgi|Guith1|57223|gw1.16.206.1
GRKKALLIGINYAGTRNALRGCINDVENMQQLLRKEGFRREEMVILTDDGRGDAMPTRNEILRACQWLVAGAGLGDV
LFFHFSGHGSQQRDDSGMESDGYNETIVPCDMQQIVDDELWNNLVFPLPSGVRLTAVMDCCHSGTGLDLPFTWKHNR
WLEDENPSHSCGDVQLFSGCQDDQTSSDGDVEKFKIGGAMTNAFIRAYNAQPFQTYPEFLSRLKSNLRSAGFGQVPQ
LSSSQAFDVNEKVFSLVEGIVPNTNVTIGRLQRRKIRPK

>GtMC2 jgi|Guith1|156638|fgenesh2_kg.347_#_2_#_2724_1_CCFI_CCFN_EXTA_EXTB
MDCFGCCPKELQAEAEKVATNEAQNAVSNATRAAPNMQNTMGAGGGNSALSQALNAVGGMQGLMGMVQSLASGQTPS
LPSTSNATDFKQKAAQALPAEVHMFSGCRDEQTSADVYDTSSFGLPADSGPGGAGGACTNSIMLALSENPNPTWIDL
LNRMRTILKEKGFTQVPQLSSSKEISLDGNFQLAAASSSTKALLIGINYVGQKGELRGCHNDVLQMKDYILKNGYDP
ASMRVLMDDGSNMNPTRANILDSIKWLVKDAKSGDCLFMHYSGHGGSMKDDNGDEADGMDETMVPVDYTSTGQIRDD
IIFQELVAPLPQGVKLTVIMDCCHSGTILDLPFSFTANDQNTQNYTSGMTLPMNGNFNWKKIFDIGMSMLKKYGMGG
AMGGAQSGMSANV

>GtMC3 jgi|Guith1|101222|au.6_g2324
MKAEARKSDQAVLFVDTACDDDKHAQKEAIHMIELVLKEETFTDTFSKRNSALSDQSKFRSKKFVDSLTQLSFESDL
IFLEHPRVFGLCMGLNEYKGELKPLETAVQDARELHDKLCSLPQSHSVLSLNPRSPSEMRKYLRKNLKQLMPASPEL
VIFFYAGHGFYEPRKKDQMLVPVDVEEVKGLSTHEINDQMLSIKDLFKIFRDFENDISLFHPMVLVIIDACREKIEG
VTEELPQDQEDKKLPQNWSLLLTCGKGKVASDNSIFFKALLDPREGMFACNRPLEGVLQSCCRTCKGRQPCISMNTY
TIPEDFCLVRDGEALRDIKMSIAGLNKAAKEIASSQLSDTDTSETETFVLAYSPEDAKSFRDRTKDALKYFLSQSKQ
RADNPRWRICSIVLMYFLQYEDTRFCTRRLLGYFYTYMEDLIAGPELVNDVLEYISRNKILSPSFGAWKKSQTNRSI
SDDKLLIAIIDYTVAKELRRVISDTEELNDALELWKKAIDPCFDSSKSWEDGMDRIERHLQKQAALECGQNLLFYFY
VLEKAVAVNSYVMILKMTRLSSVFLRHCLLEYNNKKEGEGINKLEGWRTWVSSTGWIVRRDGYRADWNHCKERWCED
MRKGFQQLEKFERPRGFQRFQDFQKDGSVVSAFMENGCDTSNKRRKLGIEYPSSSTSVRGELCESPVSGPSRQEGSD
GRGDQEDISILQFHMQYARRYDVKDFLMDRDFWSGLKWYSQDIDCYYYFRIVHTLAHFVYDNQVDIAVDTENPNEEI
DSTFAHPPPSYRAYRPAISDSPFLFGAKDLLEDDDFWANINQLIEDEIFEEHHRQMVLQIAKYVDFHKVDLYVSMDN
DSVDSDDSDEFVDIPKSNSQSQF

>GtMC4 jgi|Guith1|102616|au.10_g3718
MPRQHPRIHALCIGAGEYQYIDKLDAPKDARAMHRKFEETPLCKCFLNVDPTSKEDMQKQLQELAESSYGRPPQVTF
IFYAGHGCQLTSGPLAMLPCNFKAAEEETMMEEGTFTLDELLDILYESHWSWNKIPVVFVMDACRESIQGHSLALEA
AEPRRPARTSIFCSCSRGQLASDESSFFQDLLDPEHGIFAINQPLFDGLMFALDQSRARVTEQWARSISPEDVPKKF
CIKEDKTAKADVDQIEAAGRRGIETSQQRTSPDAATVAAYSPRASEKIEHLQSSLVQVCKGALNSFAAGTSPGKAMF
VFLSLFLRYEETEFGESWLIPCLHGLLSQGVKEVSEMINSHLMKNTITSSSLKQWIAEKCPGIDQSKLVFAALDFSV
RTELQRCSNDNPDMARRESEGWVTNVANVCYTDAITVNVAMDMANEYLSSRLAALLSIQVFERIVETGSYVVFMRMS
PLTSACLSQFLAEQVRKRLHGETGADQVFRTWISSSGWLSSTSEHRELRQYLDEDEEKHLMEILRGVGQLQHFVDLT
RVEGQGALSLLKKQDATSSSPPLVNLIELVLKGDETSKWSRFWRGVAAIWTHAQERGTSVEAVIMRLNERGICLDMF
DNTCLVRENELKEQLESSERAASILRMWWARPEEIKQGGDWLRERQQRSEGAQVGRTDLEEIFKAGMDVSRLVMRRR
KREIRDERDSQEMKMNKMCQVNLWVAGEKQGQALRDQPQSADVTAICSFSGSFDSFPRVIEGMQESVLTYSSPSKTS
LLLRVMLREVLALGFPPDLSELWAELERLQLAAGKELDFVGMCGNILYDVIVKSSQDARAWIQRNIFGMTAEELTSA
LRHHASNNSKHELLSGCFLSVNLTHFWLRKNLILDSITQLEDALEVFLFKFEDQTNRKRAVEEYLKKILVVLDACRL
SLSLRSIETELLHQESCVVVLKMPAVFGKMLLYLMENDCEINGSLNQQRLDRIVSDWLTPTHKLVSVREWFAALSSS
LPNVGDVSSRCERGRQGLKEQLGNMMTAVADQVMVGMIREGLGLLQEQDEVFTILRMFILDEQKLDERQHEKCVMIK
SSTSRRPRIATRGMRSAPAAPASSDKEWALLTLGELHDFCGSSSYVLQVIVEESDDDETETRRLQAVEVGETYRRYR
VTRDFVVSVSLVNFGSSDVVLQTFYKSDEGEAEDEGPITLKPWAHTSLENLQTKLDEGQERDTIILEDRRQATTLRL
FFEA

>GtMC5 jgi|Guith1|111492|au.52_g12594
MQDYLHKRFSALVEPFFNAHGWTSSVGAGPEPLVVRLTRGVQDKYIRLVSIIDLEEVGGGRQGGGGGGGDPLLQRLL
CFQDDLWPDDQLYLELKTSETWRDRSREDVIHGRARNVVAAIPRAIEKRESSLSATSDAAILFDGAQASQQLMQMIR
AVLDDNRERLLKRNSALSSQTGYPYQSSGSFYESEMVFLEHARVFGLCMGLSEYDGEFQPLAAAEKDAMKLHQTLCS
FKNCHSALCSNPRTLEDMKGFLRSNLTSLEEACPEQVLFFFAGHAIFEPTCKDQLLVPTALEAVRSLSSTQLLESML
SITELFRQFRDFERNVALGTPRVLVIVDACREKVECKKENLRAESDFQGMPTFWTLVQTCSNGQVAFDDSAFFQAFL
DPCEGMFACNQSLQRALLQCCSSTNTNNPKCVLTNVQNIPEDFCVISDGKDLRVVMSSVIETQKAARYVTSNASWRT
DASGADTCVLAYSAQDSVLIRQRASQALKHFVSKKEQDIPRWSFCSMVIMLFLQYDDTRFSSKTMLGHLLAKVKNGN
VGPELCEDIQEYLRHGRITSPSFEAWKEQSGLNFQDNKLLIAILDFTVTKEIRKVMPDPQEQRDALLIWEIRVVKCY
KSSTSWEKGMDLIEEHLRHVTVIDDGEDNLFYCQVLEKAADAGSFAMILKMTKLSSVFLSCCLLEYNMQHNVSELDG
LSAWVSSTGWIVREDGYNTDWTVSNALWSNELTRTFQTLERCEVGMGPRSDQTSLQQDPHNQEQRARTNSSAFYQSP
SRQNNVDRLDEITIDEMAARTQTKADGSTTPKVI

>GtMC6 jgi|Guith1|116566|au.97_g17668
MEGYVQKRFHSLLKTFSDLQAWSSSPVSQTERLLDKLYAGMQRKTTRLISIIDLEGQDSLLERLLCFREEERPDDEL
CLHVMTSESWGARSKDEAVSGRATGGSLTRARMKAIERRRGRGSDSSTRQETRKADQAVLLGEGGGGDDEGARELVR
RVMELALEGGRADESFSRRNRSVEVSLPELRGQSDGAELRGRLDLPGASSVYGLCMGLNDYQGELKPLARAVAAATE
LHEKLCSLPQCHSVLSSNPTLSAMKRYLRRSLRELEGACPELVIFFYAGHGIYESRSKDQMLIPTDVEEVRGLSFRE
MGEMMLSMEEVFKFFRDFEQDVSGYHPRVLVVIDACRDEIEGVSEGLPQDPEVKALPKHWTLLLTCGKGKTASDESK
LFESLLDAETGMFACNRPLDSVLLDCARRCKESQPCVLMNIYDIPEGFCLVSDGEALRDIRSSTAALHAAKDVVSSY
PPLTDTSKADTYVLAYSPEDAKSFRDRAMEAVKYFLGEQRAG

>GtMC8 jgi|Guith1|117476|au.111_g18578
MRGTRHVKALCIGADAYTHIAPLVEAVKDARRIHRELEDSPGCSSSFLANPTTRQSMLDMLSSLAMDCQTKRPELML
VFYAGHAIQLSSGEIAMLTCDVVRPEVSEETRGSRVTVGDALSAMAAGAENVEGLPPLLVIIDACRDEAQGRERIES
ERTNRTRSMKVSLFLSCSRGQRADDESAFLRDLLDKEHGMFARNMRLKQAIEHAMLESLRREQVAVSFCTVFIPEGL
CIRPDPTVPRDIQQIQQGARRVAESSRRLEDPTAGPTSPGPAAEVVAGYSPAAAGTGADAFRSSILGLLQESLGSLL
EESPRWRVCSMFVLVFLRYEGTRYGTQATRRYYQTRSKKRLGPDLIRAVNGHMSRNEIRSEELEQWVQETYETSTRG
KTAVAAIDFAVREEIRRSLSHMPEEARSELRLWEEDVRLRDFYEGDMTEEEALDEAEEYLRDKMTADQDGRLSPLLA
VHVLSRVVETGSFVTFLKMTRLSCACLSVALAKRVRATLDEESVGGDEVGFVVLKTLEGGGALTVLGREQEVDSHVS
EELKKLSSRPPAASEFTMMSLGGKRLGDPDDIRSDDAQSVAGSDALSQASTALHSDSRSSDARSVAGSESALSQAST
ASVSSASSREGQQRFLSVLLDKPELSEALKPFDNILPGDTWEDVRGKVKYEDVVKVGKALMKEAELIAGDFKTLMRS
KEVEKLLESSERPTDVKDFERKINAKWGPGDKQDGPYKIWAKIRTRRELYLNEFSSTEKRQKHLIVAMAYLSTFNWC
LTSKPGKPFEYALPSDQKAL

>GtMC9 jgi|Guith1|117482|au.111_g18584
MRGTGRVKALCIGADAYTHIAPLVEAVKDARRMHRELEDSSGCSSSFLANPTTRQSMLDMLSSLAMDCQTKRPELML
VFYAGHAIQLSSGEIAMLTCDVVRPEVSEETRGSRVTVGDVLSAMAAGAENVEALPPLIVIIDACRDEVQGRERIES
EITNRTRSMKVSLFLSCSRGQRADDESAFLRDLLDKEDGMFARNMRLKQAIQHAMLESRRREQVAVSFCTELIPEDL
CIRPEPTVLRDIQQIQQGARRVAESSRRLEDPTAGPTSPGPAAEVVAGYSPAAAGTGADAFRSSILGLLQESLGSLL
EESPRWRVCSMFVLVFLRYEGTRYGTQVTRRYYQTRSKKRLGPDLIRAVNGHMSRNEIRSEELEQWVQETYGTSTRS
KTAVAAIDFAVREEIRRSLSHMPEEARSELRLWEEDVRLRDFYEGDMTEEAALNEAEGYLKNKMTADQDGRLSPLLA
VHVLSRVVETGSFVTFLKMTRLSCACLSVALAKRVRVAGQGYHEHGELEERLEKEEAEQLRALLAGVGGDEEGFVAM
SKLEGGGALTVWGREQEVDSQVGEELKKLSVVPVREIKTASRLRVHQNVMRSESALSQASTASVSSAFARERHQRIL
SALLDKPELSDALEPFDNISPGDTWENVRGKVKYEDVVRVGKALMKEAELIAGDFKTLMRSKQVEKLLGTSVERPTD
VKDFERKINARWGPGDIERGPYKIWAKISNRPECFLSRLERYLNEFLSTEERQADLIVAMAYLSTFNWCLTSKPGKP
IEYALPSDQKAS

>GtMC10 jgi|Guith1|146838|fgenesh2_pg.136_#_4
MGENACRIKTLCIGIDAYKHIPTLDNAIRDATDVHNQLESSPGCQANLLVNPSTRTAITSQISSLVRDRKTRGLELI
LVFFAGHGMQLSKGDIALLTSDIVRAEESRETKQSMITVADMLEALRDGAAQNSDRIPPLLVIIDACRDSKRSAWMG
MERLEHEGSNLTQRMRVSLCLSCSRGQQASDESAFLKDLLDSELGMFAQNRRLKLAIEYAVRMSYDREQGKYGQYAR
TLCTELIPDKLCIRNDKTVAADLQQITMGVESLIQSEGMQPDSTDSGVQVVAAYSPAATRTGADVFRESLLQLVRKS
LFSLQDKGPRWKICSMFLLVFLSNKGTTYKKQGDRGFFHAFAKKKLGPELIKKVEKAMVADEIWSEKLERWIREEYG
EIDRKKMVTAAIDFTVREEIRSSLGHMEEEADTEISWWEGEVCLADHYSKGEKEQEEALDLAEEYLKDKITADQEGG
LSPLLAVHVLTRVVETGSCVAFLKMTRLSSACMSVMLAKSVREVLGGNGKEGLFRTWISSSGWLSGPREYGEMEERM
EAKEVEMLRTLLLTVGGGVSKFIDWKKLEQPDRKSLEDSKPNITMEKVDPVKKVTYREEKVPAKTSKKLSADSAGVQ
ETQLAGYFQSLPADLATGRLERNLDSSREALFANIHFDTFVYLARSMLSVKMEIEESFDGAGIGISVGVDGETSIWM
EKICQDLKVDLKRLEAHECAFYSDQSLEHQSMMTDIWFFPQLVTALQKRLTEKEIDVLDCLDEAKESENKALRKREL
LEGAAIMAHALGMNQKEKNDLIGLALRMRGKEQVYRCMTKSTQQRTERWTWQSLNADGSFRFVEVFVGSEGEGPSVP

ADSKSSRIQMIRINEGQELEDRTIGFLIIMRQTADIELLGKELNLLSEAEQRQEVSDRPCRIELQGAGDCKGHKSKI
EIIQQDLEQLTPAEFEKRVKEAFESEEFTKPCRVRGIERIVKIEILDDGFGGKKTFDRQDTGYFQAISERYNKQRSN
QDPELGLQLVIKMRVEASVLPSSEDILETLRPLDASEIETDARALGRVFPEQKAVAGRILNRLKFVLQEEKMSLRDF
EKLDFAILVPNFLTLKQQTEARMLETLFSDMTSDRECNKVVAHQAPAAEQLRKKILSISDKTLFVLISDESHWALNK
GSPQDLIINDPNLVKKKNFIVLQVSATPYPNLTLHSRIPEKYVKFEEDDPYKPVQEAKERRVGKFDEELHVIKWKLR
SRRYRTIYFRFEDFLRTLPSYVLDECILPGEEASADDQAREDVRNQLIRRENNLISTLMESVKVSRKKKEEEEAVRE
HVWLADFILSMLYFHKVRWDRSTGRLKSVADDLHNITLKDLEKLKLEEEYMTIANQCKSSGARQACAKLRERVKETL
KEERSGSAARDCIAEVFYIKLKSEAKEESVEVNDDDSFMEADDYFQFNETDRIVKDLLSNRTHNGGLYGHMKVVRMF
SIKLADQVCEELKRIRKSLFGDSVFAVIEDYGGSELYDCIETQFRDQPLTYDKDKAPRSINSLIQGRARGKKLTSLN
YKDLEGLPCILFLIEKGRMGDTFPHSFDCLDLRVRSSDNTTTLIQEMGRLCRYPTLREEHRFETSEEAVQVAKEKRW
FEGKGRPIVVYANAADDEVDLSQGLDGKRLIGIAWSLSSWTQKSILQVVERGKTLSPGRRGEEDWYTLDRNVDRSAQ
ASMAEDLKGCMLIRFPASTMNRRAVPWGDVQCLKITESRRKDDETKIRVQDISQVKKYNNFGPDSTYIIVRDSASLA
WLASRHSCPHGGVLKELEHDLPSALVSSSLYDTLMKAVETAEYCSKEIWECIRLSPQLHTHMKRKDDRGQLVISYQK
NDLHDYRNRHVAVTRAKAEGEGSKRVKVNHCDWCQDHTKRRQHQLRFLLHAECQIGKTGAYLHLLNLLRREVGLRAD
SEYACIPQIRPEDYPIKVEFQTRLAWEMPFWRDLAGQKLGLFKMKEGKYHKMVIFQRLRLLAKCLRDSRGRSSWQQL
YCNGLLAPASSQNAEGECIQVKEKVIRLLKKVSKSNSSPIEIISEGAEVKLKILDFERLRDIVEWDREDSMPSIVAD
MRKTGLKTRSGETLLQSNQDKSELSLSALGSKTDLQNIAWEGRETTAGTSSGLATGQVKNDFPARCLCLHTLPEKTK
VKLLRLSPIMRGVRIARLFGDDERRWPRISFPFGEEQVSRYFSLRDGRVNGAFELQGEKAIRNWVMTASYKRDAAGS
IQTGRAAFLDRSSAFKEGDRDVPVRQMLLVRPDDGEGDGQFSSYVSEVGSEYIVVALSHEMILSEAVSELGFRSEQF
EHQMPDDGKLRLTPQDGGIGYARLFAQLFAHLLGLERVWMIDDNVLDCYQLDLSKMFASDPPLLHPPIACSFATIML
QMQDMISDAPKSSEEICRHHRKQGKGQACPKTEGFFDHESSMTTKSCQAVRGAAAKAAAMERGVKSTPDISTIQDVR
GNTDGYAVIGMRRDVNVFMKSSVPFKITHSVYSFFLLNVKSTIGKGVLFPPKKVWEDVDFNNLCEEAGLAVLKVNRF
FHHKQHRSLHDRLEVDGQPEACEVVCRVHVNGRQIFNVACGSEMSTLSDVYETIVKRLKRLYPQSDIKKVTCRIQGG
DISFAYKESYRDIKELEGQEMCEAVYLDVLFPYEARERMQVDEAIVTTSTSKASIPFLVSLVESQRNIFYYPPRYEM
DESEGDQPFFAEVPAGRKWNEFSDTNLRNVKLKEINVMWDDVEEYKRFFNIVRNLLRDKECIRKINLILPAKAYETM
KEKIDDKIKNLRIGKMTKTWTTVLPCKEDASQEDDVDMIEASQSDNAESTMDFVVICCSSEEEKKAAQDKGEGQRAE
ADEAKQQEAKQQEAKRQEAKQQEAKRQAEEESSDSSKKPKTEEPEKRKSSEPPVGESPAKKGKAAEKQGDLWRTEKW
KRKFQEGTVRVGDNISYRPAKQEAREGKVIEGGKIEEKGRTFADVLEFFRSGGDEAPVAKLRSVYINRDGKNIISVH
DLLKQEKD

>GtMC11 jgi|Guith1|146844|fgenesh2_pg.136_#_10
MGENTCRIKTLCIGIDAYEHISTLDNAIRDATDVHNQLESSPGCQATLLVNPSTRTAITSQISSLVRDRKTRGLELI
LVFFAGHGMQLSKGDIALLTSDIVRAEESEETKQSMITVADMLEALRDGAAQNSDRIPPLLVIIDACRNSKRSAWMG
MERLEHEGSSLIQRMRVSLCLSCSRGQQASDESAFLEDLLDSEQGMFAQNRRLKLAIEYAVRMSYDREQGKYGQYAR
TLCTELIPDKLCLRNDKTVAADLQQIRMGVESLIQSEGMQPDSTDSGVQVVAAYSPAATRTGADVFRESLLQLVRKS
LFSLQDKGPRWKICSMFLLVFLSNKGTTYKKQGDRGFFHAFAKQMVGPELIKTVGKAMVADEIWSEKLERWIRERYG
EIDRKKMVTAAIDFTVREEIRSSLRHMEEEANAEIKWWTRNDYLADHYSKGEKEQEEALDLAEEYLKDKITADQEGR
LSPLLAVHVLSRVVETGSCVAFLKMTRLSSACMSVMLAKSVREVLGGNGKEGLFRTWISSSGWLSGPREYGEMEERM
EAKEVEMLRTLLLTVGGGVSKFIDWKKLEQPDRKSLEDSKPNIKMEKVDPVKKVTYREEKVPAKTSKKLSADSAGVQ
ETPLAGSMLSVKMEIEESFDGAGIGITVGVDEETSIWMKKICQDLKIDLKRLEAHECAFYSDLSLEHQSMMTDISFF
PQLVTALQKRLTKNKEINVLGCINKAKESENNDSQKRELLEGAAMMARALGMNQREKNDLISQKLPSRGKEQIYDCM
TKSTQKQTERWTWQSLNADGSFRFVEVFVGSEGEGPSVPADSKPSRIQMIRINEGQRLEDRTIGFLIIMRQTADIEL
LEKELNLLSEAEQRQEVSDRPCRIELQGAGGCKGHKSAIEIIQQDLEQLTPEEFEKRVKEAFESEEFTKPCRVRGIE
RIVKIEILDDGFGGRKTFDRQDTGYFQAISERYNKQRSNQDPELGLQLVIKMRVEASVLPSSEDVLETLRPLDASEI
ETDARALGRVFPEQKAVAGRILNRLKFVLQEEKMSLRDFEKLDFAILVPNFLTLKQQTEARMLETLFSDMTSDRECN
KVVAHQAPAAEQLRKKILSISDKTLFVLISDESHWALNKGSPQDLIINDPNLMEKKNFIVLQVSATPYPNLTLHSRI
PEKYVKFEEDDPYKPVQEAKEQKTGEFDEELHVIKWKLRSRRYRTIYFRFEDFLRTLPSYVLDECILPGEEASADDQ

TREDVRNQLIRRENNLISTLMESVKVSRKKKEEEEAVREHVWLADFILSMLYFHKVRWDRSTGRLKSVADDLHNITL
KDLEKLKLEEEYMTIANKCKSSGARQACAKLRERVKETLKEERSGSAARDCIAEVFYIKLKSEAKEESVEVNDDDSF
MEADDYFQFNETDRIVKDLLSQRKHNGGLYGHMKVVGMFSIKLADEVCEELKRIRKSLFGDSVFAVIEDYGGSELYD
CIETQFRDEPLTYDKDKAPRSINSLIQGRARGKKLTSLNYKDLEGLPCILVLIEKGRMGDTFPHSFDCLDLRVRSSD
NTTTLIQEMGRLCRYPTLREEHCFETSEEAVQVAKEKRWFEGKGRPIVVYANAADDEVDLSQGLDGKRLIGIAWSLS
SWTQKSILQVVERGKTLSPGRRGEEDWYTLDRNVDRSAQASMAEDLKGCMLIRFPTSTFKIGGSPSWGDVQCLKITE
SRRQDDETKIRVQDSSQVKKCPDSTYIIVRDSASLAWLASRHNCPHGGVLKELEHDLPSALVSSSLYDTLMKAVETA
EYCSKEIWECIRLSPQLHTHMKRRDDRGQLVISYQKNELHDYRNQHVAVTRAKAEGEGSKRVRVNHCDWCQDHTKRR
QHQLRFLLHAECQIGKTGAYLHLLNFLRREVGLRADSEYACIPQIRPEDYPIKVEFQTRLAWEMPFWRDLAGQKLGL
FKMKEGKYHKMVIFQRLRLLAKCLRDSRGRSSWQQLYCKALLAPASSQNAEGECIQVKEKVIRLLKKVWKSDSSPIE
IISEGAEVKLRILDVERLRDIVEWDREDSMPSIVADMMKAGLKTRSGETLLQSNQDKSELSLSALGSKTDLQNIAWE
GRETTAGTSQGLATGQVKNDFPARCLCLHTLPEKTKVKLLRLSPMMRGVRIARLFGDDERRWPRISFPFGEEQVSRY
FSLKDGRVNGAVELQGEKAIRNWVMTASYKRDAAGSIQTGRAAFLDRSSAFKEGDRDVPVRQMLLVRPDDSEGDGQF
SSYVSEVGSEYIVVALSHEMILSEAVSELGFRFEQFERQMPDDGKLRLTPQDGGIGYARLFAQLFAHLLGLERVWMI
DDNVLDCYQLDLSKMFASDPPLLHPPVACSFATIMLQMQDMISDAPKSSEEICRQHHKQGKGQACPKTDGFFDHESS
MIPKSCQAVRGAAAKAAAMERGVKSTSDISTIQDVRGNTDGYAVIGMRRDVNVFMNSSVPFKITHSVYSFFLLNVKS
TIGKGVLFPPKKVWEDVDFNNLCEEAGLAVLKVNRFFHHKQHRSLHDRLEVDDQPEACEVVCRVHVNGRQIFNVACG
SDMSTLSDVYETIVKRLKRLYPQSDIKKVTCRIQGGDISFAYKESYRDIKELEGQEMCEAVYLDVLFPYEARERMQV
DEAIVTTSTSKASIPFLVSLVESQRNIFYFPSRRALDESEGDQPFFAEVPAGRKWNELSDKSLDRVKLEELNIMWDD
VEAFKPFFNIVRNLLRDKECIRKINLILPAKAYESMKEKIDDKIKNLRIGNMTKTWTTVLPCKEDASQEDDVDMIEA
SQSDNAESTMDFVVICCSSEEEKKAAQDKGEGLRAEADEAKQQEADEAKQQEAKQQEAKPQAEEESSDSSKKPKTEE
PEKRKSSEPPVGESPAKKGKAAEKQGDLWRIEKWKRKFQEGAVRVGDNISYRPAKQEAREGKVIEGGKIEEKGKTFA
DVLEFFRSGVDDGDASGSAPGQKLRSVYINRDGKNFISVHDLLKQEKD

>GtMC12 jgi|Guith1|163584|estExt_fgenesh2_pg.C_400124
MSHAGVTLTSVIDFRYRDEGNQVQDRGLGPFLERMYAFRVGNNNLERYSLHVHTLKKWSEREQHVWDRARRITAVNI
RRCARRDETGYFKDVELYGLPVLPCSPHLRRHWRNYLDRSQDLEWDCTKLRVYHTETESKQETIRNVRGGLKQKEEE
EFDLVLQERLEVFALCIGVGGYEMLPPLPQALRDSHDFLHALRRMPRCRGVEISDPTENDLLYLLEKHLEVIAKFPP
SWILVTFSGHVLKEGKSGELCFLLKDFDASWLDRLDEEPEGAVTLGDIVKLWTKWFEHPRPPVLLLVDGCRTMVPDQ
PQLHAVDLPTGWSMCMSRRTERGETIQEASYMHSMMMDQENGMFAGGERLRGVLTRIRDQAPAGIAVEESWKEDPRL
PEMFSFSDIEEFPAAVLQDNDGDIREEEEQETGAGAGAGAGAGAGAGAGAGAGAGAGAGAGAGAGAGFSEILKGS
AVMKKKGVDRRMRRYDPESALKFYEKAVQLEPNDVNLLDAAGELCVEVGRSDDAVKLFRRSIELRPEENSCKYMNLG
QLMAGAESLELFLKGLALMRQELGNVAEEKGEEAKKLRGRLCSAHCSVAELYMTDLCDEENAEAGILKLLIELEQLD
EAEEFGNLLLQVDDQYVETWYLLALIHFHRGAHSAAHHCTLSAIKILEQEGVEDEDQASRLQELHEEIEKNLRDGND
NVAMEDEEGDEDGDWMTDDEEEAHP

>GtMC13 jgi|Guith1|144452|fgenesh2_pg.87_#_38
MEAAEEEEAEVRAIAKLLEVYCKGSNGKYDAYTDKKLVDTCQRELGEEMVETRTGGPKGRLLRAFADKLNKFDKFSG
KTRTEALCNLAEILNKKVGPQRQPTPERQKLALIIGISNYDKEALPNCTSDARCISEAFKSRGFETYLEFDTQDQES
FWKVYEDFASKIRPPCTVLFYVAAHGAQFHTDVLVFPIIPFRTKDAEKAISMQAMINKISTRLDPDSDVVMLLDTCR
VNFSQRGHQDLEQLATLPQTWILYATKESYPVCDGPDGGNSPFTRAVLRTLKELGPDVEFYKFATSVNKYVRDCGMQ
RSELKADMTKELFI

>GtMC14 jgi|Guith1|149022|fgenesh2_pg.268_#_3
MDETREGGGSRWDGSHLQVQLVAIEPGVTDKRKCNVRLNPPAAVLLTGTFLDPCQPCVLGRLEVSYLILTSPSLSDL
PGLALTTRCIDVCVDRRSLKSEQRGQEQTLDQDQTQEQGQTLDQEQEGRGRLEGEGKEQDCQVDGEIYEENGFTSFQ
VGGGDSQGEPAASRGVVRNEEGRGADEEQGEHGQGAPGEGPVSTRAAASPDRIEGTPPTRSRRSSGVSSSAEKSTGR

AKKSPATPGLSPDGGLDEIRQGLAKAELKEKEVEEVIEKMKSLGVRGKKDIKFVRELIDIKTLDLTEMSKLRVEKYL
KRFARKKKPASPGEEESLFRRSLKQFDVHLNVQSCMFSSRMAEDFPGWNLQEEKSIEDRQRTSVNELYERLKSCDDN
LEVDFLYLLTVSWCLSRCDPVLGFNEFPYTCGGSLKREEWRIEREVWFLLRATSEGVPPTYIVFHLLFAGMWRESRE
ERDCSVVGKVRMRSSTKVRSKKHYVKCTDTTKWDSAGGGLDLRSSSMARWRWREGAEGEESMAKVRGWLVRAEQEAP
FAQAVTVGPCWNLAGTRRRAALIVCNQAYRRARCFPELYNPVPDGRQLGEVLEELGWKVEFRVDLNLEELVGAVRCF
RDAIGDNESAAMLAFVGHGVEVHGKLFLLPTDIQLEVDNYFQREKDLAADLARCSLSFDAVQSELRGRNGTCPTLFV
LDCCRSSNNSRSPIFQPSTTVQNLELKNSCIIYSTQSGQVALEGAPGDGGPFMSAFAGELRKEEAELNQVLIETRKI
VMNSTGGAQMAPNQSLLLEQFFFCPPEIAACL

>TbMCA-I MCA2_TRYB2
MCSLITQLCDAGQLADYVGLGWLNAVSSQPYLVQALGLQPPPRRVDVDAAFRDAKGLHGHQPWVATPLPGQTVRALF
IGINYYGTSAALSGCCNDVKQMLATLQKKGLPINEAVILVDEDNFPGRTDQPTRDNIVRYMAWLVKDAKPGDVLFFH
YSGHGTQCKSRGDSDEKYDQCIAPVDFQKSGCIVDDDIHKLLFSRLPEKVRLTAVFDCCHSGSIMDLPFTYVCSGGE
QASGTPHMKRIREGNDVLGDVMMISGCADEQTSADVKNTATFGTGSTGAGGAATQCITCMLMNNQSLSYGKLLIETR
DMLKRKGFKQVPQLSASKAIDLDQTFSLTEMFSVDRSIQ

Supplementary Data 2 Alignment of the p20 domains of diatoms MCs, used to generate the phylogenetic tree in Figure 5.

Species name and MC number as dissipated in the methods, number of amino-acids in each alignment in Parentheses. Similar amino acids are in blue, identical amino acids are in gray.

```
                              10        20        30        40        50
                      ....|....|....|....|....|....|....|....|....|....|
PtMCA-IIIb(154)       -RRAVMIGINYTGQQ-GQLSGCHNDVKNMIEFIKDIHGFEDE--NITILM
Espinifer2(154)       -RRALLIGINYIGQQ-GELSGCHNDVMNIKEYIIDVHGFPEE--NITILL
Aglacialis2(154)      -KRALLIGINYIGQQ-GELSGCHNDVLNMVEYLKDVWGFEEH--NITILL
Espinifer4(154)       -KRALLIGINYVGQQ-GQLSGCHNDVLNMVEYLKDVWGFPPQ--NITVLL
Caffinis2(154)        -KRAVMIGINYVGQE-GELAGCHNDVLNMKEYLMDIHEFEEE--NMTILM
Cneogracile1(154)     -KRAVMIGINYVGQD-GELAGCHNDVLNMKEYLMDVHEFEED--NMMILM
Ccurvisetus2(154)     -KRAVLIGINYVGQQ-GELAGCHNDVLNMKEYLMDVHEFEED--NMHVLL
Cdebilis3(154)        -KRAVLIGINYVGQD-GELAGCHNDVLNMKEYLMDVHEFEED--NIHVLL
Hsinensis1(154)       -KRAVLIGINYVGQQ-GELAGCHNDVLNIKEYIMDVHGFEEE--NITVLM
Amphiprora7(154)      -RRAVVVAINYVGQQ-GELRGCHNDALNMIEYIKDVHGFPEE--NITVLM
Acoffeaeformis2(154)  -RRAVVIAINYVGQQ-GELRGCQNDAHNMIEYIKNVHGFEDE--NILILM
Tnitzschioides3(155)  -KRAVMIGINYVGDNPGELRGCHNDVFNMKKYIQDVHGFEDE--NITILM
Txantarctica1(155)    -RRAVMIGINYVGDNPGELAGCHNDVGNMKDYIQDVHDFDEE--NITLLM
Npunctata3(155)       -RRAVMIGINYVGDNPGELRGCHHDVLNMKKYIMDVHGFQEE--NITVLM
Palata2(154)          -KRALLIAINYVGQK-GELTGCINDALNMKKYIMDVWGFEEE--NITMIM
Fkerguelensis5(154)   -KRSVLIGINYVGQD-GELAGCHNDVGNMTRYIQQVHGFTET--HMTVLM
Palata6(154)          -KRAVMIGINYVGQQ-GELAGCHNDVGNMKRYIQKVHGFTDE--HMTVLM
Acoffeaeformis7(154)  -KRALLIGINYVGQQ-GELRGCHNDVGNIKRYIMDVHGFQEE--DIVVLM
Palata3(154)          -KHALLIGINYVGQS-GELSGCHNDVWNIRSYLIDVHGFEER--YITILM
Palata4(154)          -KRALLIGINYIGQN-GELSGCHNDALRIKKYLMDVYGYEEE--NFTYLM
Amphiprora8(154)      -RRAMLIGINYVGQK-GQLKACHNDVENVKDYLIKAQGFRES--EMLILK
Acoffeaeformis4(154)  -RRAMIIGINYVGQQ-GQLKACHNDADNIKDYLINAQGFREE--EMLVLK
PtMCA-b(166)          -RRAIVVGINYVGQK-GELKACHNDANNVLKYLIEAQGFDPS--QILILM
Fsolaris3(155)        RRRALLIGINYVGQE-GQLTACHNDVHNIREFLTEVYGFKES--EMLILM
Amphiprora2(154)      -KRAILIGINYVGQQ-GQLSGCHNDVKNIKKYLTEVQGFNES--EMLILM
Acoffeaeformis6(154)  -RRAVLIGINYVGQQ-GQLSGCHNDVNNIKKYLIAAQGFKES--EMLILM
PtMCA-IIIa(154)       -KRAVLIGINYTGQQ-GQLSGCHNDVKNIIKFLTKVHGFNET--EMLILM
Fsolaris2(154)        -RRAILIGINYIGQK-GQLSGCHNDAMNIKKYLINVHGFKES--EMLILM
Aglacialis3(154)      -RRAVLVGINYIGQQ-GQLSGCHNDVHNIKDYLINVQGFKEQ--DMLILM
Fkerguelensis17(154)  -RRALLIGINYVGQQ-GELSGCHNDVKNIKKYLIRKEGFQEK--DMLILM
Pfradulenta2(154)     -RRAVLIGINYEGQQ-GELSGCHNDVLNIKNYLVREEGFQEQ--DMIILM
Npunctata2(154)       -RRAILIGINYQGQK-GQLSGCHNDANNIKNYLINNQGFLEK--DMLILM
Paustralis2(154)      -RRAVLIGINYEGQQ-GELSGCHNDVKNINKFLRKEQGFHQS--DMMILM
Tnitzschioides2(154)  -KRAILIGINYTGQR-GQLSGCHNDVDNISEYLQKVHGFEAT--NMLRLV
Txantarctica4(154)    -KRAILVGINYTGQQ-GELSGCHNDVENISGYLQKVHGFPPD--QMLRFL
Tantarctica1(155)     -KRAVLIGINYVGHDPGELSGCHNDVANISKYLKGCLGFEQD--NMMVLM
Tminiscula1(155)      -KRAVLIGINYVGHNPGELSGCHNDVRNITKYLKGGLGFEGS--NMRVLM
Trotula3(154)         -KWAVLIGINYVGQN-GELSGCHNDVHNITKFLTNSQGFEQE--NMMTLL
Toceanica3(153)       -RRAVLIGINYVGQQ-GQLSGCHNDVRNIAKYLS-SMGFQQH--NMTILM
TpMCA-c(149)          -KRAVLIGINYVGQQ-GQLSGCHNDVRNIQDFLQRVHGFQQH--NMTVLM
Sdohrnii1(154)        -KRAVLIGINYVGQQ-GELSGCHNDVKNISSYLQQVQGFRPQ--NMITLM
Smarinoi2(154)        -KRAVLVGINYVGQQ-GELSGCHNDVKNISSYLQQVQGFRPQ--NMITLM
```

```
Skcos12022(153)         -KRAVLXGINYVGQQ-GELSGCHIDVKNISSYLQQVQGFRP---NMITLM
Smenzelii3(154)         -KRAVLIGINYVGQQ-GELAGCHNDVKNISKYLQQVQGFRPQ--NIITLM
Tweissflogii1010-3(154)-KRAVLIGINYVGQQ-GELSGCHNDVKNINSYLQEVLGFERE--NMKVLM
Caffinis3(154)          -RRAVMIGINYTGQQ-GQLSGCHNDVKNMIEYLKSYQGFEDR--NMTVLM
Cneogracile2(154)       -KRAVMIGINYRGQQ-GELSGCHNDVKNMVEYLKNKQGFQES--NMTILM
Cneogracile3(154)       -KRAIMIGINYVGQQ-GELSGCHTDVKNMRKYLVEVYGFKKS--DIIVLM
Caffinis1(154)          -KRAVMIGVNYTGQK-GELSGCHDDVESITSYLIESHGFEKE--NMTVLM
Ccurvisetus4(154)       -KRAVLIGINYVGQN-GELSGCHNDVKNMKEYLMNVHGFPES--NMTVLM
Cdebilis2(154)          -KRAVLIGINYTGQQ-GELSGCHNDVKNMKEYLITVHGFEEQ--NMLILM
Hsinensis3(154)         -RRAVLIGINYVGQQ-GQLSGCHNDVKNMKKYLMQVHGFEEH--NMTILM
Cdebilis1(154)          -KRAVLIGINYVGQE-GELSGCHNDVIRIKDYLINFQGFEER--HITLLM
Palata5(154)            -RRAVLIGINYVGQS-GELSGCHNDVFNVKDYLVNVLGFENR--HITLLL
Ccurvisetus1(154)       -KRAVLVGINYVGLP-GQLSACHYDVKKVKEYLITELGFEEK--NMIILM
Dbrightwellii4-4(154)   -KRALLIGINYVGQN-GELSGCHNDVLNIKEYLMDVLGFEED--NIMVLM
Tantarctica4(153)       --RAVLIGINYTGQS-GELSGCHNDVHNVAKYLMEVQGFKKE--NVTILM
Tminiscula4(153)        --RAVLIGINYTGQS-GELSGCHNDVHNVARYLTEVQGFRKE--NVTILM
Tgravida1(154)          -KRAVLIGINYTGQK-GELSGCHNDVKNVARYLSEVQGFKKE--NVTILM
Trotula4(154)           -KRAVLIGINYTGQK-GELSGCHNDVNNVARYLSEVQGFKKE--NVTILM
TpMCA-IIIb(153)         --RAVLIGINYVGQK-GQLSGCHNDVLNVAKYLKEVQGFRKE--NMTILM
Sdohrnii3(153)          --RAVLIGINYKGQK-GQLSGCHNDVHNVTQYLKEVQGFKDE--NITILM
Smarinoi3(153)          --RAVLIGINYKGQK-GQLSGCHNDVHNVTQYLKEVQGFKDE--NITILM
Skcos16234(153)         --RAVLIGINYKGQK-GXLSGCHNDVHNVTXYLKEVQGFKDE--NITILM
Smenzelii1(153)         --RALLIGINYKGQT-GQLSGCHNDVHNVSQYLQEVQGFKED--NITMLM
Tweissflogii1010-2(154)-KRAVLIGINYTGQS-GELSGCHNDVKNISKYLREVQGFKKE--NVTILM
Toceanica2(153)         -KRAVLIGINYTGTP-GELRGCHNDCLNVARFLRE-QGFRDE--NVTMLL
Dbrightwellii4-1(156)   -KRALLIGINYTGMQPGELTGCHNDVKNVKEYLMNEHSFKEE--NITVML
Dbrightwellii4-2(155)   -RRAVLIGINYTGMNPGALTGCHNDVHNIKHYIMDVWGFKEE--HITVLL
Cpennatum1(154)         -KRAVLIGINYVGQS-GQLSGCHNDVLNIQKYIMDVHGFQEE--NIEVLM
Cpennatum5(154)         -KRAVLIGINYVGQS-GQLSGCHNDVKNIKRYIMDVHGFQEE--NIEVLM
Cdebilis4(156)          -KRAVLIGINYVGQN-GELAGCQNDALNMKEYLKDVHDFKDE--NIQLLL
Espinifer3(154)         -KRALLIGINYVGQS-GELSGCHNDVLNIKEYLIDVLGFKEK--NITVLM
Amphiprora1(156)        -RRALLIGINYIGHSQGVLSGCHNDVLNMVEYIKEVHGFEED--SITVLL
Fsolaris1(156)          -KRAVLVGINYIGHSQGVLSGCHNDVKNMVDYIKDVHGFEEQ--NITVIM
PtMCA-IIIc(156)         -RRALLIGINYVGHEQGVLRGCHNDVKNMVEYIKAVHGFEDE--NITILM
Aglacialis1(156)        -RRALLIGINYVGHESGVLSGCHNDALNMKEYIMDVHGFEEE--NITLLL
Acoffeaeformis1(155)    -RRAVMIGINYVGHDPGELSGCHNDVFNMAEYIKDCHGFTDD--DIVYLL
Acoffeaeformis5(155)    -RRAVMIGINYTGDNPGELRGCHNDVHNMKEYIKNCHGFTDA--DITLLL
Cpennatum2(155)         -RRALLIGINYGGQN-GELSGCHNDVINIKGYIEKVHGFSED--NMTILM
Cpennatum7(156)         -RRALLIGINYVGMD-GELDGCHNDVLNMKSYIEKVHGFKNS--NISILM
Tantarctica2(155)       -RRAVMIGINYVGDSPGELSGCWNDVLNMKNYIMDVHGFEEE--NIVILM
Tweissflogii1010-1(155)-RRAVMIGINYVGDEPGELSGCWNDVLNMKKYIMDVHGFEED--NIVVLM
Tminiscula3(156)        -KRAVMIGINYVGDNPGELSGCWNDVLNMKKYIMDVHGFEED--DIVILM
TpMCA-IIIa(156)         -RRAVMIGINYIGDNPGELSGCHNDVLNMKKYIMDVHGFEED--NIVVLM
Tgravida2(155)          -RRAVMIGINYVGDRPGELSGCHNDVHNMKKYITDVHGFDEE--NIRILM
Trotula2(155)           -RRAVMIGINYVGDRPGELSGCHNDVHNMKKYIMDVHGFEEE--NIRTLM
Toceanica1(155)         -KRAVMIGINYVGDSPGELSGCWNDVLNMKRYIMQVHGFDEE--NIVILM
Sdohrnii2(155)          -RRAVMIGINYIGDDPGELSGCWNDVLNMKKYIIDVHGFEEE--NIVILM
Smarinoi1(155)          -KRAVMIGINYVGDDPGELSGCWNDVLNMKKYIQDVHGFEEE--NIVILM
Smenzelii2(155)         -KRAVMIGINYVGDSPGELSGCWNDVLNMKKYIQDVHGFEEE--NIVILM
Skcos13618(155)         -KRAVMIGVNYVGDEDNELSGSHNDVLNMKRYIQEVRGFEEE--NIVILM
```

```
Acoffeaeformis3(157)    -RRAVMIGINYVGHDPGELRGCHNDVLNMKKYIMAVHGFAEE--NIQVLM
Txantarctica3(155)      -RRAVMIGINYVGDDPGELRGCHNDVLNMKKYIMHVHGFEED--NITILM
Npunctata6(155)         -RRAVMIGINYIGDDPGELRGCHNDVLNMKKYIMEVHGFEED--NIVVLM
Tnitzschioides1(155)    -RRAVMVGINYVGHNPGELRGCHNDVLNMKKYIIAVHGFEEE--NIHVLM
Fkerguelensis8(156)     -RRAVMIGINYVGHAQGQLSGCHNDVLNMKKYIMDVHGFEEE--NIVVLM
Paustralis3(155)        -RRAVMIGINYVGHSPGELRGCHNDVLNMKKYIMDVHGFEED--NIVILM
Pfradulenta1(154)       -RRAVMIGINYVGQD-GELRGCHNDVLNMKKYLMDVHGFEEE--NIVILM
Fkerguelensis10(153)    --RAVLIGINYRGQK-GELKGCHNDVFNLYNYIQDWYGFQDE--NVTVLA
Npunctata1(154)         -RRAVLIGINYRGQK-GELRGCHNDVFNMYNYIQDYYGFQDE--DITVLV
Fkerguelensis9(156)     -RRAVMVGISYDG---G-LIGCHNDVINMKKYLIERCDFKEY--YVDVLM
Dbrightwellii4-3(154)   -RRAVLVGINYVGMN-GELSGCHNDVYNIKKYIMDVHGFEEE--NIVMLV
Txantarctica2(155)      -KRAVLIGINYTGQS-GELSGCQNDCINIKDYIMNVWGFEEE--NIVILM
Tminiscula2(157)        -RYAVLIGINYTSHRRGRLRGCHNDVHNICKYIMDVGNVEES--NITILL
Trotula1(157)           -RYAVLIGINYTSHRRGRLRGCHNDVHNICKYIMDVGKVEES--NITILL
Fkerguelensis15(156)    -RYAVLIGINYTSHKQGQLSGCHNDVGNIKKYIMDVGKIKAH--NITTLM
Paustralis1(159)        --RALLVGINYYGQR-GQLSGCINDVLNVKKYLCNYQGFLEK--HVLLLI
Pfradulenta3(160)       -RRALLVGINYYGQS-GQLSGCINDVLNVKKYLCNHHGFLEK--HVLLLI
Npunctata4(161)         -RRALLVGINYFGQN-GQLSGCINDVNNVKKYLCQYEGYLEK--NILVLT
Amphiprora4(159)        -RRALLVGINYRGQS-GELSGCQNDVYRMRDYLCQVQGYLPS--NVVLLV
Cpennatum4(158)         -RRAVLVGINYRGQS-GELSGCHNDVFNMKKYIMDVHGFQER--NILVLV
Cpennatum8(160)         -RRAVLVGINYRGQS-GELSGCHNDVFNMKKYIMDVHGFHER--NIIVLV
Txantarctica6(158)      -KRALLVGINYIGQN-GELRGCHNDVYNVKQYLVSYHGYRER--DIQILV
Dbrightwellii4-5(161)   -RRALLVGINYLGQN-GELSGCFNDVYNIKKYLQNHHGFAPK--DIVVLM
TpMCA-I(157)            --KALIIGINYTG-TRAALKGCVNDAKSMQQLLMR-NGFGDDGSHMLLLT
Skcos11238(157)         --KSLLIGINYTG-TRAALRGCINDAKNMQTLLLK-NGFPNDGSHMLMLT
Throt7051(157)          --KCLIIGINYTG-SRAQLRGCINDAKNMQGLLRR-NGYPDDGSHMLLLT
Dbrightwellii4-6(157)   -RKALLIGINYKG-TRAALRGCVNDAKNIKNLLME-NGFRDDPTHMVMLV
Dbrightwellii4-7(156)   -RKALLIGINYAE-SPAPLQGCVNDAKNIKNLLIE-NGFTDDPTHMVMLT
Palata1(166)            -RKALLIGINYTN-TRASLRGCVNDAMKIKGLLVE-NGFTDDAAHMTLLI
Tnitzschioides4(158)    -RRALLIGINYTG-TRAALRGCINDAKNMRNVLIRKHAFPGDTCHMVMLT
Txantarctica5(159)      -RRALIIGINYTGDKRAALRGCINDAKNMKNLLLR-NQFPNDGSHMVVLV
Espinifer1(156)         -RRALLIGINYKG-TRAALRGCINDANNMERLLVQ-HGFPRDSCHMVKLV
Pfradulenta4(157)       -RKALFIGINYKG-QRGELKGCVNDVVNIKKFFRT--HYTLN--DTLVLT
Throt6337(154)          -RRALFIGINYFG-QKGELRGCINDVHNIQGFLTS--NFRID--EVMVLT
Espinifer5(139)         --RALLVGINYAEEE-NQQSSSHNDVKNMHRYLTEVAGFDAD--NVTMLM
PtMCA-a(158)            -KRSLLIGCNYSDIPEAQLKASHDDVRSIKDYIVNVHGFPEACGLMTVLM
Fsolaris4(149)          ---------NYHELKDAELKASHDDIRSMKDYIVNVHNFPEGKEYMTVLL
Hsinensis4(159)         KKLSLLIGCNYNAVTDAQIKASHDDVRSVKDYIVNVHGFPEKKGAMTILL
Skcos4616(158)          -KRTLLIGCNYRKTRDAQLKACHDDVTSIKDFLVNVYGFPESPDLMTILM
Throt14982(158)         -KRALLIGCNYRRMPDAELKACHDDVRSVKDFLVNVYGFPESPELMTVLM
TpMCA-a(156)            -KRALLIGCNYRKTPDAGLKASHDDVRSMKDFLVNVYGFPETSDLMTVLM

                                  60        70        80        90        100
                        ....|....|....|....|....|....|....|....|....|....|
PtMCA-IIIb(154)         DDGA-----------HTEPTYKNILAAYHELVSSAKAGDAIFCHYSGHGG
Espinifer2(154)         DDGE-----------HRDPTRENILDAYKALVSASKAGDACFCHYSGHGG
Aglacialis2(154)        DDGE-----------HTNPTRSNILSAYQQIVAEAESGDAVYCHYSGHGG
Espinifer4(154)         DDGS-----------HVNPTRSNILNAYRTLVAESQPGDAVYCHYSGHGG
Caffinis2(154)          DDGV-----------HPDPTRDNIMYAYRKIVAESQAGDVVYLHYSGHGG
Cneogracile1(154)       DDGE-----------HVEPNQANILSAYRRVVALSQPGDVVYLHYSGHGG
```

Ccurvisetus2(154)	DDGV-----------HEDPTYDNIMNAYRQLVSSSVEGDCVYIHYSGHGG
Cdebilis3(154)	DDGV-----------HEDPTSENIIAAYRRIVAESVDGDCVYCHYSGHGG
Hsinensis1(154)	DDGV-----------YSDPTRENILNAYKELVASSEAGDVAFIHYSGHGG
Amphiprora7(154)	DDGQ-----------HTEPTRDNILAAYRKVVRDSEPGDVVFTHYSGHGG
Acoffeaeformis2(154)	DDGI-----------HPDPTYENIITAYRQLVASCEPGDAVFCHYSGHGG
Tnitzschioides3(155)	DDGN-----------HTVPTRENILAAYSKIVEESQPGDAVFCHYSGHGG
Txantarctica1(155)	DDGE-----------HISPTRDNILAAYRRIVEESQPGDAVFCHYSGHGG
Npunctata3(155)	DDGE-----------HIPPTRENILGAYRKIVAESESGDVVFCHYSGHGG
Palata2(154)	DDGE-----------HMEPTRENILNAYNDLVIQSEAGDSIFCHYSGHGG
Fkerguelensis5(154)	DDGS-----------HQAPTYDNIMSAFRQLVQDTQEGDAVFVHYSGHGG
Palata6(154)	DDGQ-----------HEEPTYGNIMAAFRQLVENTKEGDAVFVHYSGHGG
Acoffeaeformis7(154)	DDGN-----------HELPTYDNIIAAFRRLVNDTESGDCAFFHYSGHGG
Palata3(154)	DDGS-----------HTSPTRENIMSAFRMLVKSSVGGDSAFCHYSGHGG
Palata4(154)	DNGV-----------YDDPTYDNILAAFDDIVAKSEAGDSVFVHYSGHGG
Amphiprora8(154)	DDGR-----------HEMPTKQNIMDGFVRLTQYSQPGDVVFVSFSGHGG
Acoffeaeformis4(154)	DDGK-----------HMMPTKQNIMDGFTRLTQYSQPGDVVFVSFSGHGG
PtMCA-b(166)	DDGK-----------HTEPTRRNIEDAFVRMTQYSQPGDVVWVSFSGHGG
Fsolaris3(155)	DDAR-----------HNPPTRRNIEDAMIRLTRYSQPNDVAFVSFSGHGG
Amphiprora2(154)	DDGN-----------HHPPTRKNIIDAFKRIGEYSNAGDVVFVHYSGHGG
Acoffeaeformis6(154)	DDGR-----------HHAPTRKNIEDAFRRITEYSNAGDVVFVHYSGHGG
PtMCA-IIIa(154)	DDGQ-----------HHSPTKKNIEDAFTRITQYSQAGDVVFVHYSGHGG
Fsolaris2(154)	DDNK-----------HHPPTRRNIEDAFQRITQYSQAGDVVFVHYSGHGS
Aglacialis3(154)	DDGR-----------SHEPTRENILKSLKRVTEYSEPGDVVFFHYSGHGG
Fkerguelensis17(154)	DDGR-----------QHAPTRQNIMEAFDRITQYSKAGDVVFIHYSGHGG
Pfradulenta2(154)	DDGQ-----------HNLPTRKNILDAFDRIVQYSKAGDVVFIHYSGHGG
Npunctata2(154)	DDGR-----------HHQPTRANIMNAFDRIVQYSKAGDVVFIHYSGHGG
Paustralis2(154)	DDGS-----------STPPTRRNIMDAFDQVVQNSTAGDVVFIHYSGHGG
Tnitzschioides2(154)	DNGV-----------DHAPTHANIMSAFSRIGDYSKAGDVVFLHYSGHGS
Txantarctica4(154)	DNGV-----------DHSPTRANLMSAFERIAAYSEPGDTVFMHYSGHGS
Tantarctica1(155)	DDHR-----------HEEPTYRNIMRAFDWIVSESQPGDTVWIHYSGHGG
Tminiscula1(155)	DDGR-----------HEQPTYRNIMRAFDWIVNVSQPGDTVWIHYSGHGG
Trotula3(154)	DDGH-----------HKEPTYRNIMDAFSWITRAAQPGDTVWIHYSGHGG
Toceanica3(153)	DDGM-----------HEEPTYRNIMEAFKWIVQESQPGDTVWIHYSGHGG
TpMCA-c(149)	DDGR-----------HKEPTYAKIMAAFDWIVKESMAGDTVWIHYSGHGG
Sdohrnii1(154)	DDGV-----------HDNPTYDRILQAFQWVVNESQAGDTVWIHYSGHGG
Smarinoi2(154)	DDGV-----------HDNPTYDRILQAFQWVVNESQAGDTVWIHYSGHGG
Skcos12022(153)	DDGV-----------HDNPTYDRILQAFQWVVNESXAGDTVWIHYSGHGG
Smenzelii3(154)	DDGI-----------HDNPTYDKIMQAFQWVVNESQAGDTVWILYSGHGG
Tweissflogii1010-3(154)	DDGM-----------HEEPTFENIVRAFKWVVKESAPGDTVWIHYSGHGG
Caffinis3(154)	DDGY-----------HHDPTYYNIMDAYRTVVRESRPGDTVFLHYSGHGG
Cneogracile2(154)	DDGY-----------HTNPTYSNIMNAYRTLVRTSQKGDTVFIHYSGHGG
Cneogracile3(154)	DDGI-----------HHNPTKHNIMKAYRKIVRDSLPGDTVFLHFSGHGG
Caffinis1(154)	DDGI-----------HEPPTYRNIMVACRAIVRESKPGDTVFFHYSGHGG
Ccurvisetus4(154)	DDGY-----------HLNPTRYNITNAYQNLVRQSMPGDSVFCHYSGHGG
Cdebilis2(154)	DDNY-----------HPNPTRMNVLNAYRNLVRESKPGDTAFCHYSGHGG
Hsinensis3(154)	DDGY-----------HREPTYRNIVNAYKDVVRQSLPGDTVFCHYSGHGG
Cdebilis1(154)	DDGI-----------NINPTKGRIIRAYRRIVKVSKAGDTVFCHYSGHGG
Palata5(154)	DDGL-----------NTSPTRENITYAYRDLVNRSAPGDVAFCHYSGHGG
Ccurvisetus1(154)	DDDH-----------HTRPTKYAILTAYRRLVEESKAGDTVYCHFSGHGS
Dbrightwellii4-4(154)	DDGI-----------HHEPTRDSILSGYRRLVAESVAGDTVFCHYSGHGG

Tantarctica4(153) DDGA-----------HKPPTKAGIVNAYKRLVKESKEGDVAFCHYSGHGG
Tminiscula4(153) DDGM-----------HKAPTKSAILGAYKRLVKESREGDVVFCHYSGHGG
Tgravida1(154) DDGN-----------HKPPTKTAIISAYKKLVKESKKGDVVFCHYSGHGG
Trotula4(154) DDGN-----------HKPPTKSAIISAYKKLVKESKKGDVVFCHYSGHGG
TpMCA-IIIb(153) DDGN-----------HKSPTKSAILSAYKKIVKESKEGDVVFCHYSGHGG
Sdohrnii3(153) DDGM-----------HKPPTKSAIISAFKRLVKQTKEGDVVFCHYSGHGG
Smarinoi3(153) DDGM-----------HKPPTKSAIISAFKRLVKQTKEGDVVFCQYSGHGG
Skcos16234(153) DDGM-----------HKPPTKXAIISAFKRLVKQTKEGDVVFCQYSGHGG
Smenzelii1(153) DDGM-----------HKPPTKSAIISAFKRLVKQTKEGDVVFFHYSGHGG
Tweissflogii1010-2(154) DDGQ-----------HKEPTKANIIASYKKLVKDCKSGDVVFCHYSGHGG
Toceanica2(153) DDNK-----------HRSPTKAAILSAYKKLVRESKPGDVVFCHYSGHGG
Dbrightwellii4-1(156) DDNV-----------HESPTKANIISGYKKLTKESQPGDAVFCHFSGHGS
Dbrightwellii4-2(155) DDGK-----------HKPPTRANILGAYAALAAKCQPGDVAFCHYSGHGG
Cpennatum1(154) DDGY-----------HTSPTRHNITVAYQRIAEVSQSGDAVFCHYSGHGG
Cpennatum5(154) DDGH-----------HTSPDRRNIMEAYARIAEESQPGDAVFCHYSGHGG
Cdebilis4(156) DDGT-----------NTNPTKRNILKAYAKIAEESDAGDVVYLHYAGHGG
Espinifer3(154) DDNE-----------SERPTGSNIIDAFKDLASLSKSGDAVYFHYSGHGG
Amphiprora1(156) DDGE-----------HDAPTKENILAAYKKMVAEAEPGDSLFCHYSGHGC
Fsolaris1(156) DDGE-----------HPEPTKANIVAAYKKLIEESQPGDCLFCHYSGHGA
PtMCA-IIIc(156) DDGE-----------HTAPTHANMIAAYKKIVALSKADDALFCHFSGHGA
Aglacialis1(156) DDGE-----------HDPPTKENILNAYKKLVEESEKGDSVFCHYSGHGG
Acoffeaeformis1(155) DDGE-----------HTPPTAANIIEAFQTLASQAQPGDACFLHYSGHGC
Acoffeaeformis5(155) DDGK-----------NTAPTAANILAAFKKLASEAKAGDACFVHYSGHGC
Cpennatum2(155) DDAE-----------HEEPTRKNILKGFAELVKSCEAGDSVFVHYSGHGG
Cpennatum7(156) DDGKN---------RHANPTKKRILRNFKKLVRECEEGDSVFVHYSGHGV
Tantarctica2(155) DDGE-----------NIEPNYENIIDAYKAVVSQSEDGDAIFLHYSGHGT
Tweissflogii1010-1(155) DDGE-----------NTEPTRENIIEAYRTVVAHSEEGDAIFLHYSGHGT
Tminiscula3(156) DDGE-----------HIEPTAENIINAYKTVVAEAEEGDAIFLHYSGHGT
TpMCA-IIIa(156) DDGE-----------HTEPTHDNIMNAYKKVIADAEDGDAIFLHYSGHGT
Tgravida2(155) DDGE-----------NEAPTRSNIIAAYKAVVDEAEEGDAIFLHYSGHGT
Trotula2(155) DDGE-----------NEAPTRSNIIAAYKAVIAEAEEGDAIFLHYSGHGT
Toceanica1(155) DDGE-----------HTAPTFRNIIDAYKIVISQAEEGDSIFLHYSGHGT
Sdohrnii2(155) DDGI-----------HTEPTKANIIDAYKTIIAQAEENDAIFLHYSGHGT
Smarinoi1(155) DDGI-----------HTEPTKANIIDAYKTIIAQAEENDAIFLHYSGHGT
Smenzelii2(155) DDGN-----------HTEPTKANIIDAYKTIIGQAEENDAIFLHYSGHGT
Skcos13618(155) DDGK-----------HTNPTKKNIIHACKKVIRQAEENDAILFLYSGHGT
Acoffeaeformis3(157) DDGE-----------HTPPTKENILAAYKKVIAETQSGDAVFLHYSGHGT
Txantarctica3(155) DDGE-----------HDSPTKENILEAYKKVIEETEESDAIFLHYSGHGT
Npunctata6(155) DDGE-----------HDPPTRDNILAAYEKVVADSEAGDAIFLHYSGHGT
Tnitzschioides1(155) DDGE-----------HEEPTRANLIAAYEKIVAETESGDAIFLHYSGHGT
Fkerguelensis8(156) DDGI-----------HSSPTASNMIQAYQQIVADSEDGDAIFLHYSGHGT
Paustralis3(155) DDGI-----------HESPTKENMVAAYKQIVADSEEGDAIFLHYSGHGT
Pfradulenta1(154) DDGV-----------HDEPTRENMIEAYQQIVDESEDGDAVFLHYSGHGT
Fkerguelensis10(153) DDGD-----------HQLPTKQNILAAYHRIVRQSRSGDSIFLHYSGHGT
Npunctata1(154) DDAD-----------HPPPTKANIIAAYQSVVAKSQPGDAIFLHYSGHGT
Fkerguelensis9(156) DDGV-----------HTLPTKSNIMNALLRVVNDCNDGDAVFFFFSGHGT
Dbrightwellii4-3(154) DDGE-----------CEMPTRENMMNAYKWLVESSEAGDAVFTHFSGHGG
Txantarctica2(155) DDGN-----------HTNPTRSNILEAYKNLVTNSEDGDAAFCHYSGHGG
Tminiscula2(157) DD-G----------TSTPPTRENIMQALDELTQKCQPGDTAFVHYSGHGG
Trotula1(157) DD-G----------EATEPTRANIMEALDELCQKCEPDDTAFVHYSGHGG

```
Fkerguelensis15(156)   DDAG----------ISIPPTKRNIMKAFDELTRKCQPGDCAFVHYSGHGS
Paustralis1(159)       DDGRN-----------HHPTRDNIIRALQRLVKQSKPGDSVYFHYSGHGG
Pfradulenta3(160)      DDGRN-----------HKPTRENIIRALRRLVEHSKAGDSVYFHYSGHGG
Npunctata4(161)        DDGRN---------P-HNPTREKIIRALRQLVAQSVSGDSVYFHYSGHGG
Amphiprora4(159)       DDGKA-----------HYPNRKNIISVLQQLVLQSTSGDSVFFHYSGHGG
Cpennatum4(158)        DDTRH---------P--IPTRQKIIAALEALVANSVAGDNVYFHYSGHGG
Cpennatum8(160)        DDTSH---------P--VPTRHRIIAALEALVSNSVAGDNVYFHYSGHGG
Txantarctica6(158)     DDYNN---------RSIFPTRQKIISALQYLVQSSVAGDAVYFHYSGHGG
Dbrightwellii4-5(161)  DDGKH---------S--FPTRQRIIQELKKLVSCSIGGDSVFFHYSGHGG
TpMCA-I(157)           DER--------SRGREYQPNATNIMKAFAWFMKDVQKGDVLFFHFSGHGG
Skcos11238(157)        DER--------HRGSEYQPNASNIMKAMSWLMKDAQKGDVLFFHFSGHGG
Throt7051(157)         DER--------NRGREYQPNKEMITKAFAWLMKDVRKGDVLFFHFSGHGG
Dbrightwellii4-6(157)  DEG--------ARNSNYLPTRANIMRGFQWLVQGVSEGDALFFHFSGHGS
Dbrightwellii4-7(156)  DE---------EQNSKYLPTKANIRRGFQWLTQGVSEGDVLFLHFSGHGS
Palata1(166)           DETPEPHAGGGGAVSSYLPTKANIVKGLQWLVSGSRKGDVLFFHFSGHGA
Tnitzschioides4(158)   DEP--------SRGRNYQPTYSNIRRGLQWLLQGVSQGDVLFFHFSGHGA
Txantarctica5(159)     DDS--------TSSNNHRPTHSNIFKAMQWLMQGVRGGDVLFFHFSGHGA
Espinifer1(156)        DD---------SRNRNYLPTRANITKACQWLLQGVSKGDVLFFHYSGHGA
Pfradulenta4(157)      DDKRA------EPETDHAPTRKNILNAFKWLVKGAKPGDSLLLHYSGHGG
Throt6337(154)         DDQK---------DPRYIPTRQNILNAFKWLRNGAKAGDSLILHYSGHGG
Espinifer5(139)        DDGD-----------CPLPSKANIIETWKELAEDSKAGDSVFFHFSGKGS
PtMCA-a(158)           DDKN-----------HKKPTFLNIVEAFKALSEEAQPGDAIFIQFSGHGG
Fsolaris4(149)         DDGE-----------HPPPTFMNIVEAFKALSEESQPGDVVFVQFSGHGG
Hsinensis4(159)        DDKE-----------HSPPTHSNIIQAFKHLAEESKPGDAVFIQFSGHGG
Skcos4616(158)         DDKK-----------HKSPTHKNITEAFKRLAEQSQPGDAVFVLFTGHGC
Throt14982(158)        DDKK-----------HKHPTHSNMTQAFKKLAERSQPGDAVFVLFTGHGC
TpMCA-a(156)           DDKH-----------HQHPTHENITEAFKRLAEKSQPGDAVVVLFSGHGC

                                110       120       130       140       150
                       ....|....|....|....|....|....|....|....|....|....|
PtMCA-IIIb(154)        KVRDDDG-------DEADGYDETLVP---VDFNAA---GQIRDDDIFSAL
Espinifer2(154)        KLRDDDG-------DEADGYDETLVP---LDYQRA---GQIRDDDIFNTL
Aglacialis2(154)       RLRDDDG-------DEEDGYDETLVP---LDYQRA---GQIRDDDLFNIL
Espinifer4(154)        KLRDDDG-------DEKDGYDETLVP---LDYQSA---GQIRDDDLFNTL
Caffinis2(154)         KLRDDGN-------DEEDGYDETLIP---LDYQSA---GQIRDDDLLRTL
Cneogracile1(154)      KLRDDNN-------DEEDGYDETLIP---LDYNSA---GQIRDDDLLRIL
Ccurvisetus2(154)      KVRDRDG-------DEADGYDETLIP---LDYMNA---GQIKDDDLLRTL
Cdebilis3(154)         KVRDDNG-------DEADGYDETLVP---LDFQEA---GQIRDDDLLKTL
Hsinensis1(154)        KLRDDDG-------DEADGYDETLVP---LDYMSA---GQIRDDDLLTTL
Amphiprora7(154)       KLRDQDG-------DEKDGYDETLVP---VDYASS---GQIRDDDLFKYL
Acoffeaeformis2(154)   KLVDEDG-------DEEDGYDETLVP---VDYQSA---GQIRDDTLYKEL
Tnitzschioides3(155)   KLKDDNG-------DEADGYDETLVP---VDYKTA---GQIRDDLLYKNL
Txantarctica1(155)     KLRDDNG-------DEADGYDETLVP---VDYATE---GQIRDDLLFKNL
Npunctata3(155)        KLRDDNG-------DEEDGFDETLVP---RDYQTA---GQIRDDALYDTL
Palata2(154)           KLRDDNG-------DEEDGYDETLVP---VDYARS---GQIRDDDLYEIL
Fkerguelensis5(154)    KVADQDG-------DEADGYDETLVP---VDFQGA---GQIRDDDIFKVL
Palata6(154)           KLRDDDG-------DEADGYDETLVP---LDYQEA---GQIRDDDIFSTL
Acoffeaeformis7(154)   RLPDDNG-------DEEDGYDETLIP---VDYQSA---GQIRDDLLYSDL
Palata3(154)           KIRDNNN-------DESDGYDETLVP---LDHYAS---GQIRDDDVYDAL
Palata4(154)           FLADDSC-------DEEDGKDETLIP---VNYTEA---GQIRDDTVFNRL
```

```
Amphiprora8(154)        RVVDTSG-------DEDDGFDESLIP---VDFQEV---GQIVDDDILDIF
Acoffeaeformis4(154)    RVVDTSG-------DEEDGYDESIIP---VDFREN---GQIIDDDLLDLF
PtMCA-b(166)            RAVDISG-------DEDDGYDETLIP---LDFMKH---GQIIDDDILDMF
Fsolaris3(155)          NTRDTSG-------DEADGMDETLIP---VDFRTE---GHIVDDDILRLL
Amphiprora2(154)        RVRDTSG-------DEDDGYDETLIP---VDFQRA---GQIVDDDILKIL
Acoffeaeformis6(154)    RVRDTSG-------DEEDGYDETLIP---VDFRSA---GQIVDDNILDML
PtMCA-IIIa(154)         RVRDLDG-------DEDDGFDETLIP---VDFKRA---GQIIDDDILKIL
Fsolaris2(154)          RIPDLDG-------DESDGYDETLVP---VDFKSA---GQIVDDEILKLL
Aglacialis3(154)        QVRDTSG-------DEEDGYDETLIP---VDFKRS---GQIIDDLLSDVL
Fkerguelensis17(154)    KIRDTSG-------DEADGYDETLIP---LDFKQA---GQIVDDEIYSRF
Pfradulenta2(154)       RVYDSSG-------DEADGYDETLIP---LDYQSA---GQIVDDDLYRQL
Npunctata2(154)         RVVDTSG-------DEADGYDETLIP---VDFQRA---GQIVDDDLYKHL
Paustralis2(154)        NVPDESG-------DEEDGYDETLIP---VDFQSA---GQIIDDELYERL
Tnitzschioides2(154)    RVPDRDG-------DEDDGYDETLVP---VDFERS---GQIVDDDILRKL
Txantarctica4(154)      RVKDYNG-------DEDDGFDETLVP---VDFESN---GQIVDDDLLDKF
Tantarctica1(155)       RLEDQDG-------DEEDGYDETLCP---IDFQTA---GQIRDDDLLKHL
Tminiscula1(155)        RLPDQDG-------DEEDGYDETLCP---IDFNTA---GQIRDDDLLKHL
Trotula3(154)           RVEDQDG-------DEDDGYDETLIP---VDFQSA---GQIRDDDLLKYL
Toceanica3(153)         SVEDDDG-------DEEDGRDETLIP---LDFQRA---GQIRDDDLLKYL
TpMCA-c(149)            RVADQDG-------DEDDGYDETLIP---VDFQRK---GQIRDDDLLKHL
Sdohrnii1(154)          RVEDDNG-------DEDDGYDETLIP---VDFQRK---GQIRDDDLLRYL
Smarinoi2(154)          RVEDDNG-------DEDDGYDETLIP---VDFQRK---GQIRDDDLLRYL
Skcos12022(153)         RVEDDNG-------DEDDGYDETLIP---VDFQRK---GQIRDDDLLRYL
Smenzelii3(154)         RVPDDNG-------DEDDGYDETLIP---IDFQRK---GQIRDDDLLRYL
Tweissflogii1010-3(154) RLEDQDG-------DEDDGYDETLIP---IDFQSE---GQIRDDDLLKYL
Caffinis3(154)          RVRDTSG-------DEDDGFDETLIP---VDYQRS---GQIVDDDLCTQL
Cneogracile2(154)       RVRDLNG-------DEDDGFDETLIP---VDFKRA---GQIVDDDLCKEL
Cneogracile3(154)       RQVDLNG-------DEDDGYDETLIP---VDFQEA---GQIVDDDLWVEL
Caffinis1(154)          RIQDINK-------DEADGFDETIIP---SDYFRA---GQITDDDLNKTL
Ccurvisetus4(154)       RVRDTSG-------DEDDGFDETLIP---VDFQSS---GQIIDDDLFKNL
Cdebilis2(154)          SVKDYSG-------DEEDGFDETLIP---VDFQRS---GQIIDDDLFKEL
Hsinensis3(154)         SVRDASG-------DEEDGYDETLIP---VDFQTA---GQILDDYLLENL
Cdebilis1(154)          RTKDLDG-------DESDGLDETLIP---MDYQSA---GQIIDDDLFKEL
Palata5(154)            FVDDTSG-------DEEDGRDETLIP---VDFQSA---GQITDDELLKDL
Ccurvisetus1(154)       RVVDTNG-------DEDDGYDETLIP---VDFYRA---GQIVDDDLLKLF
Dbrightwellii4-4(154)   RLVDDDG-------DEDDGYDETLIP---VDFEHA---GQIRDDVLFNEF
Tantarctica4(153)       RLPDDNG-------DEEDGYDETLIP---VDYDKK---GQIRDDDLLKIL
Tminiscula4(153)        RLPDDNG-------DEEDGYDETLIP---VDYDKK---GQIRDDDLLKIL
Tgravida1(154)          RLPDDNG-------DEEDGYDETLIP---VDFEKR---GQIRDDDLLKIL
Trotula4(154)           RLPDDNG-------DEEDGYDETLIP---VDFEKR---GQIRDDDLLKIL
TpMCA-IIIb(153)         RIKDDNG-------DEDDGHDETLIP---VDFEKA---GQIRDDDLLKIL
Sdohrnii3(153)          RLPDDNG-------DEDDGYDETLIP---LDFQKS---GHIRDDDLLKIL
Smarinoi3(153)          RLPDDNG-------DEDDGYDETLIP---LDFQKS---GHIRDDDLLKIL
Skcos16234(153)         RXPDDNG-------DEDXGYDETLIP---LDFQKS---GHIRDDDLLKIL
Smenzelii1(153)         RLPDDNG-------DEDDGFDETLIP---LDFKKS---GQIRDDDLLKIL
Tweissflogii1010-2(154) RLPDDNG-------DEDDGHDETLIP---VDFEKN---GQIRDDDLLNML
Toceanica2(153)         RLPDDNG-------DEDDGWDETLIP---VDFKTA---GQIRDDDLFKFL
Dbrightwellii4-1(156)   QIKDDDWF------EEEDGLDETLVP---VDFKEN---GQIRDDDIFDIL
Dbrightwellii4-2(155)   MLPDDNG-------DEDDGFDETLIP---LDFKRT---GQIRDDDLFKIL
Cpennatum1(154)         KLPDDNG-------DEDDGYDETLIP---VDYEHA---GQMRDDELLQRL
```

```
Cpennatum5(154)          KLPDDNG-------DEDDGYDETLIP---VDYEHA---GQIRDDELLKKL
Cdebilis4(156)           HKSDRGG-------DEKDGRDETLIP---VDFQKWG-VGEILDDDIYQKL
Espinifer3(154)          KLRDLNG-------DEKDGYDETVIP---VDYDTY---GQIRDDTLYKKL
Amphiprora1(156)         KIRDDDRG------EEEDGFDEALVP---VDYQT---AGLIRDDDLFDII
Fsolaris1(156)           KIKDDEQG------EEKDGYDEVLVP---VDYEQ---AGMIRDDDLFDIL
PtMCA-IIIc(156)          KIRDDDRG------EEDDGYDETLVP---IDYHE---NGMIRDDDLYDIL
Aglacialis1(156)         STRDDDRG------EEEDGKDETLIP---VDYKT---AGIIRDDDLFDTL
Acoffeaeformis1(155)     SIPDQDG-------DEDDGKDEALCP---VDYAE---NGILRDDHVLQML
Acoffeaeformis5(155)     SIRDDDG-------DEADGMDEALCP---VDYKK---SGVLRDDDVLEKL
Cpennatum2(155)          SIKDDDSG------EEEDGMDECLCP---VDYAL---NGVIRDDDVYSTL
Cpennatum7(156)          SLRDKDR-------NEKDGMDECLCP---LDYTA---KGYIRDDDIYSTL
Tantarctica2(155)        KLRDDN-N------EEADGYDEALCP---RDFQS---SGMIRDDDLYEIL
Tweissflogii1010-1(155)  KLRDDD-S------DEADGYDEALVP---RDFQE---NGMIRDDDLYEIL
Tminiscula3(156)         KLKDDDHN------EEADGYDEALVP---RDYQS---TGMIRDDDLYEIL
TpMCA-IIIa(156)          KLRDDDFG------EEKDGYDEALCP---RDFAS---AGMIRDDDLYDIL
Tgravida2(155)           KLRDQN-G------DEADGYDEALCP---RDFQQ---AGMIRDDDLYDIL
Trotula2(155)            KLRDQN-G------DEADGYDEALCP---RDFQQ---AGMIRDDDLYDIL
Toceanica1(155)          KMKDDD-G------DEEDGYDEALCP---RDYAS---AGLIRDDDLYDIL
Sdohrnii2(155)           KLRDDD-G------DESDGYDEALVP---RDFQT---SGMIRDDDLYELL
Smarinoi1(155)           KLSDDD-G------DESDGYDEALVP---RDFQT---NGMIRDDDLYELL
Smenzelii2(155)          KLRDND-G------DEGDGYDEALVP---RDFQT---SGMILDDDLYDLL
Skcos13618(155)          RVEDDN-G------DERDGFDEAIVP---RDFEE---NGFILDDDLYEIL
Acoffeaeformis3(157)     KLRDDDWK------EEEDGYDEALVP---VDYQE--GAGMIRDDDLYEIL
Txantarctica3(155)       KVKDRDG-------DEADGYDEALVP---VDYQE---EGLINDDDLYDIL
Npunctata6(155)          KLRDDDG-------DEDDGYDEALVP---RDYNE---AGMIRDDDLYDIL
Tnitzschioides1(155)     KLRDDDG-------DEDDGYDEALVP---LDFKE---TGMIRDDDLFDIV
Fkerguelensis8(156)      KLRDDNG-------DEDDGYDEALVP---VDFNE--GGGMIRDDDLFDIL
Paustralis3(155)         KLVDKSG-------DEDDGYDEALVP---LDFQE--VG-MIMDDDLFDIL
Pfradulenta1(154)        KLVDGSG-------DEEDGYDEALVP---LDYDE--AG-MLLDDDLFEIL
Fkerguelensis10(153)     KVPDLN-G------DEDDGYDEALVP---LDFD--R-SGVILDDELYEIF
Npunctata1(154)          KLPDDN-G------DEDDGYDEALVP---LDFK--S-AGMIRDDYLYDII
Fkerguelensis9(156)      EIVDEN-G------DEKDGYDECLVPCDYLEYENFK-DGVISDDDLFDNI
Dbrightwellii4-3(154)    QLRDQD-G------DEADGFDETLIP---VDYLE---NGQIRDDDLYDTL
Txantarctica2(155)       RVRDDDRG------EEADGYDETLIP---VDYDT---SGFIRDDDLYSNL
Tminiscula2(157)         RVKDETG-------EDPTGFNSTLVP---LDFNKR-GVGHILDKELYEHL
Trotula1(157)            RVKDETG-------QDPTGFNSTLCP---VDFDQP-GVGQILDKELYEHL
Fkerguelensis15(156)     RQKDYTG-------QEESGYNSTLVP---IDFN---EAGQIVDDELYEHL
Paustralis1(159)         LLDPNYWN-RYKAGVSNKKYDETLYP---VDHLKA---GQIRDFNLFNHF
Pfradulenta3(160)        LLDPDYWN-RYKAGKKDKQYDETLYP---VDHART---GQIRDFSLFNHF
Npunctata4(161)          LLDPDYWN-RFKAGKLHKEYDETLYP---VDHERA---GQIRDFSLFNHF
Amphiprora4(159)         LLEPSLCNNAWKRSVED--YDEILYP---LDHKQA---GHIRDYSLFRHF
Cpennatum4(158)          LLEPEANL-FKLGGG--DTYDETLYP---LDHTRA---GQIRDYSLFNRF
Cpennatum8(160)          LLAPEANL-FKLGGGGDDGYDETLYP---LDHTRA---GQIRDYSLFNRF
Txantarctica6(158)       LLEPNA---FRSSSK--KDYDETLFP---LDFDQS---GQIRDYSLYQNF
Dbrightwellii4-5(161)    LLDADGFNLFKAANPNAKQYDETLYP---VDHGTS---GQIRDFSLYSHF
TpMCA-I(157)             QVPDKTG-------HEADGFNETIVP---LDYERA---GQISDDVLWGSL
Skcos11238(157)          QVPDKTG-------HEADGFNETLIP---LDHTRA---GQISDDVLWGSL
Throt7051(157)           QQRDTTG-------MEVDGYNETLIP---VDYDRK---GQISDDVLWGSL
Dbrightwellii4-6(157)    QVPDKSG-------FEEDGLNETILP---VDYQK----GQISDDVIWSSI
Dbrightwellii4-7(156)    QVSDEAG-------MEEDGLNETILP---MDYQK----GQITDDMIWSSV
```

```
Palata1(166)             QVPDATG-------QEADGLNETILP---LDYQKA---GMISDDTIWGSM
Tnitzschioides4(158)     QVPDKTG-------HEADGLNETILP---LDYENK----QITDDELWGSI
Txantarctica5(159)       QVPDRTG-------HEADGLNETILP---LDYKKG---GQITDDEMWGSL
Espinifer1(156)          QVPDRSG-------MESDGYNETILP---MDYRSG----QITDDALWNSL
Pfradulenta4(157)        KSKNHDG-------TEASGFDQTLIP---VDYEKA---GQILDDDVHDVL
Throt6337(154)           SVKDTDG-------DEEDGMDETLIP---VDYQKA---GQIVDDEVHTVL
Espinifer5(139)          ---------------------ETIAP---VDYENA---GQICDGDMYNAF
PtMCA-a(158)             RVLDSHIDT------EAESYDEVLVP---CDYLQS---GLIRDTLIFKTL
Fsolaris4(149)           RVLGDPSD-------QVGTYDEVVVP---SDYKSS---GLIRDTLMFKTL
Hsinensis4(159)          RVLDTRLDS------EGECYDEVLIP---SDFTTK---GLIRDTLMFKTL
Skcos4616(158)           RIMDSPIDA------TAESYDEALLP---SDYEES---GIIRDTLMFKTL
Throt14982(158)          RVLDSPIDE------SAESYDEALVP---ADYEET---GIIRDTLFFKTL
TpMCA-a(156)             RVLDSPSE--------AESYDEALVP---SDYNVS---GNIRDTLVFKTL

                                   160       170       180       190
                         ....|....|....|....|....|....|....|....|....
PtMCA-IIIb(154)          IGPMPAGVTLT------------SVMDCCHSGTVLDLPYVFKAD
Espinifer2(154)          IGPMPKGVTLT------------CIMDCCHSGTVLDLPYVFKAD
Aglacialis2(154)         VGPMKAGVVLT------------CVMDCCHSGTVLDLPYCFVAD
Espinifer4(154)          VGPMKKGVQAT------------CIMDCCHSGSILDLPYVFKAD
Caffinis2(154)           VIPMQEGVFVT------------SVMDCCHSGTVLDLPYNYQGE
Cneogracile1(154)        VLPMQAGVYVT------------SVMDCCHSGTVLDLPYNFKGD
Ccurvisetus2(154)        VIPMQRGVFVT------------SVMDCCHSGTVLDLPYVFKAD
Cdebilis3(154)           VIPMRKGVFVT------------SVMDCCHSGTVLDLPYTFKAD
Hsinensis1(154)          VIPMAAGVFMT------------SIMDCCHSGTVLDLPYNFKAD
Amphiprora7(154)         VAPMAAGVVMT------------CLMDCCHSGTVLDLPFEFVAD
Acoffeaeformis2(154)     VGALPEGVMMT------------SVMDCCHSGTVLDLPYVFVAD
Tnitzschioides3(155)     VGGFKEGVFCT------------FVMDCCHSGSVLDLPFQFQAD
Txantarctica1(155)       VGGFREGVFCT------------FVMDCCHSGSVLDLPFQFKAD
Npunctata3(155)          VAPMKAGVFAT------------FVMDCCHSGTVLDLPFNFVAD
Palata2(154)             VGCMPRGVYMT------------CLMDCCHSGTVLDLPYKFVAP
Fkerguelensis5(154)      IGPMPRNVTMT------------CVMDCCHSGTVLDLPYTFVAD
Palata6(154)             IGPMPRNVTMT------------CVMDCCHSGTVLDLPYSFVAD
Acoffeaeformis7(154)     VGRMPEGSTLT------------CLMDCCHSGSVLDLPYTFQAD
Palata3(154)             VGPMPSNVTLT------------CLMDCCHSGTVLDLPYKFKPD
Palata4(154)             VLPMADDVTLT------------CLMDCCHSGTILDLPYKFKAG
Amphiprora8(154)         VKKMKGGVTCT------------VVMDCCHSGSVMDLPYYFSAD
Acoffeaeformis4(154)     VKRLKGGVFCV------------VVMDCCHSGTVMDLPYAFSAN
PtMCA-b(166)             VKPMKKGVNVTGDFRSRDDFPWKVLMDCCHSGTVLDLPYTYSSG
Fsolaris3(155)           VKPMMTDVHVT------------VLMDCCHSGTVLDLPYKFGAN
Amphiprora2(154)         VKPIKAGVYVT------------VLMDCCHSGTVLDLPYRFSAD
Acoffeaeformis6(154)     VKPMRAGVTVT------------VLMDCCHSGTVLDLPYRFSAD
PtMCA-IIIa(154)          VKPMRQGVTVT------------VLMDCCHSGTVLDLPYRFSAD
Fsolaris2(154)           VKPMKAGVTCT------------VLMDCCHSGTVLDLPYRFSAD
Aglacialis3(154)         VKSLKKGVTLT------------ALMDCCHSGTVLDLPYRFDAD
Fkerguelensis17(154)     VTKMPGNVTVV------------VVMDCCHSGTVLDLPYEINAT
Pfradulenta2(154)        VTRMPEGVTVV------------VLMDCCHSGTALDLPYEINAT
Npunctata2(154)          VKRMPAGVHVT------------VLMDCCHSGTALDLPYEIDAT
Paustralis2(154)         VTKMPAGVTVV------------VLMDCCHSGTALDLPYQINAT
Tnitzschioides2(154)     VKPLAAGVTMT------------CLMDCCHSGTVLDLPYRFTAD
```

```
Txantarctica4(154)      VKPLKSGVNMT------------CLMDCCHSGTVLDLPYRFTAD
Tantarctica1(155)       VKPMKKGVLMT------------CLMDCCHSGTVLDLPYNFIAD
Tminiscula1(155)        VKPMREGVLMT------------CLMDCCHSGTVLDLPYNFIAD
Trotula3(154)           VKPMREGVLMT------------CLMDCCHSGTVLDLPYNFIAD
Toceanica3(153)         VKPMKRGVLVT------------CLMDCCHSGTVLDLPYNFYAD
TpMCA-c(149)            VKPMRKGVVVT------------ALMDCCHSGTVLDLPY-----
Sdohrnii1(154)          VKPMREGVLMT------------CLMDCCHSGTVLDLPYRFIAD
Smarinoi2(154)          VKPMREAVLMT------------CLMDCCHSGTVLDLPYRFIAD
Skcos12022(153)         VKPMREGLLMT------------CLMDCCHSGTVLDLPYRFIAD
Smenzelii3(154)         VKPMRQGVLMT------------CLMDCCHSGTVLDLPYRFIAD
Tweissflogii1010-3(154) VQPMCEGVTMT------------CLMDCCHSGTVLDLPYRFIAD
Caffinis3(154)          VKAMPQGVLVT------------SLMDCCHSGTVLDLPYRFTAD
Cneogracile2(154)       VKAMPRGVLVT------------SLMDCCHSGTVLDLPYRFTAD
Cneogracile3(154)       VKAMPQRVLVT------------SLMDCCHSGTVLDLPYQYAAD
Caffinis1(154)          VKPMKKGVYVV------------ALMDCCHSGTVLDLPYVCTEG
Ccurvisetus4(154)       VQPLPKGVLMT------------CLMDCCHSGTVLDLPYRFTAD
Cdebilis2(154)          VRPMSTGVLMT------------CLMDCCHSGTVLDLPYRFTAD
Hsinensis3(154)         VKPLSRDVLMT------------CLMDCCHSGTVLDLPYRFTAD
Cdebilis1(154)          VQPMSEGVHAT------------CLMDCCHSGTVLDLPYHFTAE
Palata5(154)            VHPMKAGVLTT------------CLMDCCHSGTVMDLPYRFTAD
Ccurvisetus1(154)       VKPMAKGVLMT------------CMFDCCHSGTVLDLPYYFNAD
Dbrightwellii4-4(154)   VRPMAEGVTVT------------CLMDCCHSGTVLDLPYRFVAD
Tantarctica4(153)       VHPLQEGVTMT------------CLMDCCHSGTVLDLPYRFTSD
Tminiscula4(153)        IHPMPKGVTMT------------CLMDCCHSGTVLDLPYRFTAD
Tgravida1(154)          VSPMSEGVTMT------------CLMDCCHSGTVLDLPYRFTSD
Trotula4(154)           VGPMPEGVTMT------------CLMDCCHSGTVLDLPYRFTSD
TpMCA-IIIb(153)         VHPMAAGVRMT------------CCMDCCHSGTVLDLPYRFTAD
Sdohrnii3(153)          IHPMPAKVTMT------------CLMDSCHSGTVLDLPYRFVGD
Smarinoi3(153)          IHPMPAKVTMT------------CLMDSCHSGTVLDLPYRFVGD
Skcos16234(153)         IHPMPAKVTMT------------CLMDSCHSGTVLDLPYRFVGD
Smenzelii1(153)         VHPMPANVSMT------------CLMDCCHSGTVLDLPYRFVGD
Tweissflogii1010-2(154) VNPMPKGVTMT------------CLMDCCHSGTVLDLPYRFTAD
Toceanica2(153)         VHPMPANVTMT------------CLMDCCHSGTVLDLPYRFTAD
Dbrightwellii4-1(156)   VVPMKEGVTLT------------CFFDCCHSGTVMDLPFTYRAD
Dbrightwellii4-2(155)   VVPMKAGVTLT------------CLFDCCHSGTVLDLPYNFQAD
Cpennatum1(154)         VLPLRAGVTMF------------SLMDCCHSGTILDLPYRFRAD
Cpennatum5(154)         VLPLQAGVTMF------------SLMDCCHSGTILDLPYRFRAD
Cdebilis4(156)          VLKLKKGVFAT------------SVMDCCHSGTVLDLPYKFVGD
Espinifer3(154)         IGAMRSGVNLT------------CVMDCCHSGTVLDLPYKFVAN
Amphiprora1(156)        IKPMPDGANLF------------CLFDCCHSGTILDLPYLYKAD
Fsolaris1(156)          IRPMQQGVSLF------------CLMDCCHSGTVMDLPYIFKAD
PtMCA-IIIc(156)         IKPLVQGVHLV------------CLMDCCHSGTVLDLPYVYKAD
Aglacialis1(156)        VHPMKEGVTLT------------CLMDCCHSGTILDLPYTFKAD
Acoffeaeformis1(155)    VAPMPRGVTLT------------CIMDCCHSGTILDLPFLFLAD
Acoffeaeformis5(155)    IAPLPRGVTLT------------CIMDCCHSGTILDLPYLFLAD
Cpennatum2(155)         VAGMPGGARVT------------CLMDCCHSGSILDLPYLYTAG
Cpennatum7(156)         VAAMPAGVRMT------------CIMDCCHSGSILDLPYAYKAK
Tantarctica2(155)       VKELRDGVHMT------------SLMDCCHSGSIMDLPYIFKGD
Tweissflogii1010-1(155) VKDLADGVHLV------------SLMDCCHSGTIMDLPYIFKGD
Tminiscula3(156)        VKGCPDGVHMI------------SLMDCCHSGTILDLPYIFKGD
```

TpMCA-IIIa(156)	VKGCPDGVHMV------------SLMDCCHSGSIMDLPYIFKGD
Tgravida2(155)	VKALPDGVHMV------------SLMDCCHSGSIMDLPYIFKGD
Trotula2(155)	VKGLPNGVHMV------------SLMDCCHSGSIMDLPYIFKGD
Toceanica1(155)	VKELPDGVHMF------------SLMDCCHSGSIMDLPYVFKGG
Sdohrnii2(155)	FKNLPDGVHMT------------SLFDCCHSGTILDLPYLFKGD
Smarinoi1(155)	LKNLPDGVHMT------------SLMDCCHSGTILDLPYLFKGD
Smenzelii2(155)	IKDLPDGVHMT------------CLMDCCHSGTIMDLPYLFKGD
Skcos13618(155)	IKDLPKGVSMF------------SLFDCCHSATIMDLPYLFKGD
Acoffeaeformis3(157)	VKPLAHGAHMV------------SLMDCCHSGTILDLPYIFKAD
Txantarctica3(155)	CKPLAKGATMV------------SLMDCCHSGTILDLPYIFKAD
Npunctata6(155)	IKPLADGVHMV------------SLMDCCHSGTVLDLPYIFKAD
Tnitzschioides1(155)	CKPLADGAHMV------------SLMDCCHSGTILDLPYIFKAD
Fkerguelensis8(156)	IKPLAQNVHMV------------SLMDCCHSGTILDLPYIFAPK
Paustralis3(155)	IAPLQDGVHMV------------SLMDCCHSGTILDLPYIFKPN
Pfradulenta1(154)	IEPLAEGVHMV------------SLMDCCHSGTILDLPYIFKPN
Fkerguelensis10(153)	VKGLPSNVHVV------------ALMDCCHSGTVLDLPYIFKAD
Npunctata1(154)	VKALPSGVHVV------------AVMDCCHSGTVLDLPYVFKAN
Fkerguelensis9(156)	ISELPSDVHMV------------ALMDCCHSGTILDLPYILKAN
Dbrightwellii4-3(154)	VKPMKVGVVVT------------SLMDCCHSGTVLDLPYTFRAD
Txantarctica2(155)	VCAMSQGVTLV------------SLMDCCHSATVLDLPFKYGAD
Tminiscula2(157)	VCAMPSGTSLT------------CLMDCCHSGTVLDLPYNFVAD
Trotula1(157)	VCAMPAGTSLT------------CLMDCCHSGTVLDLPYNFIAD
Fkerguelensis15(156)	VLKMPRGSTLT------------CLMDCCHSGTVLDLPFNFVAD
Paustralis1(159)	VKPMAAGVTVT------------CVMDCCHSGSVLDLPYSYRPT
Pfradulenta3(160)	VKPMTAGVTVT------------CVMDCCHSGSVLDLPYSYRPT
Npunctata4(161)	VKPMSAGVTVT------------CVMDCCHSGSVLDLPYSYRPT
Amphiprora4(159)	VKPMPAGVTVT------------CVMDCCHSGGVLDLPYSYKAT
Cpennatum4(158)	VKPMAAGVTVT------------CVMDCCHSGSVLDLPYSFQPT
Cpennatum8(160)	VRPMAAGVTVT------------CVMDCCHSGSVLDLPYSFQPT
Txantarctica6(158)	VQPMPAGVVVT------------CVMDCCHSGSVLDLPYSFQPT
Dbrightwellii4-5(161)	VQPMKEGVFVT------------CLMDCCHSGSVLDLPYSFQPT
TpMCA-I(157)	VYPMPEGCRLI------------ALMDMCHSGTGLDLPFDYNVD
Skcos11238(157)	VYNLPEGARLT------------ALMDMCHSGTGLDLPYDYNVN
Throt7051(157)	VYRLPEGARIT------------ALMDMCHSGTGLDLPYDYNVD
Dbrightwellii4-6(157)	VYPLPSGVKLT------------VIMDCCHSGTGLDLPFDYMLD
Dbrightwellii4-7(156)	VYPLPSGVKLT------------VVMDCCHSGTGLDLPFDYEPY
Palata1(166)	VYPLAEGVRLT------------ALMDCCHSGTGLDLPFECQLK
Tnitzschioides4(158)	VYPLPAGARLT------------ALMDCCHSGTGLDLPFEYMYK
Txantarctica5(159)	VYPLPAGARLT------------ALMDCCHSGTGLDLPFEYQKG
Espinifer1(156)	IAPLPDGTRLT------------SVMDCCHSGTGLDLPYDVDIR
Pfradulenta4(157)	CKSLPKGVRLT------------AIFDCCHSESIMDLPFVYNVN
Throt6337(154)	CRGLPKGVRLT------------AIMDCCHSESMLDLPYIYTIN
Espinifer5(139)	VKKIPKGVTVT------------CLMDSCHSGTVLELPYVFKAD
PtMCA-a(158)	LAPMRYGVTVT------------ILIDCCDNGMVLELPYSWSTK
Fsolaris4(149)	LAPMRYGVTVT------------IIIDCCDNGMVLELPYCWGTM
Hsinensis4(159)	LAPMRDGVTVT------------ILLDCCDTGVMLDLPYAWTTK
Skcos4616(158)	FAPMKKGVTVT------------CIMDCAHTGVMIDLPYLWTSK
Throt14982(158)	LAPMKKGVTLT------------CIVDCCHTGVMIDLPYLWTTK
TpMCA-a(156)	LAPMKKGVTMT------------CIIDCCHTGMMMDLPYIWSSK
